# Monograph of the Afrotropical species of *Scelio* Latreille (Hymenoptera, Platygastridae), egg parasitoids of acridid grasshoppers (Orthoptera, Acrididae)

**DOI:** 10.3897/zookeys.380.5755

**Published:** 2014-02-17

**Authors:** Matthew J. Yoder, Alejandro A. Valerio, Andrew Polaszek, Simon van Noort, Lubomír Masner, Norman F. Johnson

**Affiliations:** 1 Department of Entomology, The Ohio State University, 1315 Kinnear Road, Columbus, Ohio 43212, USA; 2 Dept of Life Sciences, Natural History Museum, London SW7 5BD, UK; 3 Iziko South African Museum, PO Box 61, Cape Town, 8000, South Africa; 4 Agriculture and Agri-Food Canada, K.W. Neatby Building, Ottawa, Ontario K1A 0C6, Canada; 5 Illinois Natural History Survey, 1816 South Oak Street, MC 652, Champaign, IL 61820

**Keywords:** Africa, Scelioninae, biodiversity informatics, locust, grasshopper, new species, taxonomy

## Abstract

The genus *Scelio* is a cosmopolitan and speciose group of solitary parasitoids of the eggs of short-horned grasshoppers (Orthoptera: Acrididae). A number of these hosts are important pests, including plague locusts of the genus *Schistocerca*. Species of *Scelio* are recognized as potentially important biological control agents, but this possibility has yet to be fully realized, in part because the species-level taxonomy is still incompletely developed. The species of the pulchripennis group have been recently revised. As a continuation of this effort, here we revise the Afrotropical species of *Scelio*, excluding the *pulchripennis* species group. Sixty two (62) species are treated, 48 of which are new. Species are classified into the following species groups: *ernstii* (12 species, 9 new), *howardi* (23 species, 19 new), *ipomeae* (6 species, 5 new), *irwini* (4 species, 3 new), *simoni* (3 new species) and *walkeri* (12 species, 9 new). Keys to species groups and to the species within each group are provided. New species described are: *S. albatus* Yoder, **sp. n.**, *S. aphares* Yoder, **sp. n.**, *S. apospastos* Yoder, **sp. n.**, *S. ardelio* Yoder, **sp. n.**, *S. aurantium* Yoder, **sp. n.**, *S. balo* Valerio & Yoder, **sp. n.**, *S. bayanga* Yoder, **sp. n.**, *S. bubulo* Yoder, **sp. n.**, *S. cano* Yoder, **sp. n.**, *S. clypeatus* Yoder, **sp. n.**, *S. concavus* Yoder, **sp. n.**, *S. copelandi* Yoder, **sp. n.**, *S. crepo* Yoder, **sp. n.**, *S. destico* Yoder, **sp. n.**, *S. dupondi* Yoder, **sp. n.**, *S. effervesco* Yoder, **sp. n.**, *S. erugatus* Yoder, **sp. n.**, *S. exophthalmus* Yoder, **sp. n.**, *S. fremo* Valerio & Yoder, **sp. n.**, *S. gemo* Yoder, **sp. n.**, *S. grunnio* Yoder, **sp. n.**, *S. harinhalai* Yoder, **sp. n.**, *S. igland* Yoder, **sp. n.**, *S. impostor* Yoder, **sp. n.**, *S. irwini* Yoder, **sp. n.**, *S. janseni* Yoder, **sp. n.**, *S. latro* Yoder, **sp. n.**, *S. memorabilis* Yoder, **sp. n.**, *S. modulus* Yoder, **sp. n.**, *S. mutio* Yoder, **sp. n.**, *S. ntchisii* Yoder, **sp. n.**, *S. parkeri* Yoder, **sp. n.**, *S. phaeoprora* Yoder, **sp. n.**, *S. pilosilatus* Yoder, **sp. n.**, *S. pipilo* Yoder, **sp. n.**, *S. quasiclypeatus* Yoder, **sp.** n., *S. retifrons* Yoder, **sp. n.**, *S. ructo* Yoder, **sp. n.**, *S. scomma* Yoder, **sp. n.**, *S. simoni* Yoder, **sp. n.**, *S. simonolus* Yoder, **sp. n.**, *S. somaliensis* Yoder, **sp. n.**, *S. susurro* Yoder, **sp. n.**, *S. tono* Yoder, **sp. n.**, *S. transtrum* Yoder, **sp. n.**, *S. tritus* Yoder, **sp. n.**, *S. ululo* Yoder, **sp. n.**, *S. vannoorti* Valerio & Yoder, **sp. n.** The following species are redescribed: *S. afer* Kieffer, *S. chapmani* Nixon, *S. howardi* Crawford, *S. ipomeae* Risbec, **stat. n.**, *S. mauritanicus* Risbec, *S. philippinensis* Ashmead, *S. remaudierei* Ferrière, *S. striatus* Priesner,*S. taylori* Nixon, and *S. zolotarevskyi* Ferrière. The genus *Lepidoscelio* Kieffer is treated as a junior synonym of *Scelio* Latreille, **syn. n.**; its type species, *Lepidoscelio fuscipennis* Kieffer, 1905 is transferred to *Scelio*, renamed *Scelio obscuripennis* Johnson, **nom. n.** (preoccupied by *Scelio fuscipennis* Ashmead, 1887), and redescribed. The following additional species are transferred from *Lepidoscelio* to *Scelio*: *S. cayennensis* (Risbec), **comb. n.**, *S. insularis* Ashmead, **rev. comb.**, *S. luteus* (Cameron), **comb. n.**, *S. thoracicus* Ashmead, **rev. comb.** Lectotypes are designated for *S. africanus* Risbec, *S. ipomeae* Risbec, *S. mauritanicus* Risbec, *S. remaudierei* Ferrière, *S. sudanensis* Ferrière, and *S. zolotarevskyi* Ferrière. *Scelio gaudens* Nixon is a junior synonym of *Scelio striatus* Priesner, **syn. n.**; *Scelio africanus* Risbec and *Scelio clarus* Fouts are both junior synonyms of *Scelio afer* Kieffer, **syn. n.**; *Scelio sudanensis* Ferrière and *Scelio cheops* Nixon are both junior synonyms of *Scelio zolotarevskyi* Ferrière, **syn. n.**; *Scelio cahirensis* Priesner is a junior synonym of *Scelio mauritanicus* Risbec, **syn. n.** The name *Scelio chapmanni* Nixon is an incorrect original spelling, requiring an emendation to *S. chapmani*. Digital versions of the identification keys are available at http://www.waspweb.org/Platygastroidea/Keys/index.htm

## Introduction

This paper is the second in a series that seeks to revise and update our knowledge of a majority of the world species of *Scelio* Latreille. Currently the genus encompasses 255 species considered to be valid, and we estimate that there may be a minimum of 500 species of *Scelio* worldwide. Dangerfield et al. (2001) summarized the existing literature pertaining to the biology and the ecology of species of this genus. Aside from the recent treatment of the *pulchripennis* species group (Yoder et al. 2009b), species of African *Scelio* have been largely neglected since the work of Nixon (1958). Nixon treated 19 species and also defined many of the species groups now recognized. His observations were particularly insightful, and he identified many important morphological character systems that we reuse and extend.

The geographical range of species treated in this work is restricted to the Afrotropics: sub-Saharan Africa and Madagascar. Surrounding islands (e.g. Seychelles) are presently excluded due to lack of available material. This circumscription largely excludes the species treated by Priesner (1951). Despite several efforts of the last decade we have been unable to locate many of Priesner’s *Scelio* types. However, we were able to review most of his species and here, where pertinent, provide discussion and propose synonymies.

This work is a product of the Platygastroidea Planetary Biodiversity Inventory, a project funded by the U.S. National Science Foundation (N.F. Johnson and A.D. Austin, University of Adelaide, principal investigators). One of the primary objectives of this project is to use biodiversity informatics tools to accelerate the taxonomic process and to make real-time collaboration possible among the narrow community of researchers with appropriate expertise. The contributions of the individual authors are: M.J. Yoder: project coordination, manuscript preparation, character definition, species concept development, species group concept development; key development, imaging, figures, databasing of specimen data; A. Valerio: species concept development, species group concept development, imaging, databasing of specimen data; A. Polaszek: species concept development; imaging; critical review; L. Masner: character development, development of collection, species group concept development; S. van Noort: collection development, imaging, interactive key development; N.F. Johnson: project coordination, species identification, critical review. The authorship of the new taxa reflects the contribution of each individual.

## Materials and methods

A total of 6,449 specimens have been databased and examined in this study. The following collections provided specimens for this study: AEIC, American Entomological Institute, Gainesville, FL^1^; AMNH, American Museum of Natural History, New York, NY^2^; BMNH, Natural History Museum, London, UK^3^; BPBM, Bishop Museum of Entomology, Honolulu, HI^4^; CASC, California Academy of Sciences, San Francisco, CA^5^; CNCI, Canadian National Collection of Insects, Ottawa, Canada^6^; EMEC, Essig Museum of Entomology, Berkeley, CA^7^; FMNH, Field Museum of Natural History, Chicago, IL^8^; INHS, Illinois Natural History Survey, Champaign, IL^9^; MCSN, Museo Civico di Storia Naturale Giacomo Doria, Genova, Italy^10^; MNHN, Muséum National d’Histoire Naturelle, Paris, France^11^; MZLU, Lund University, Lund Museum of Zoology, Lund, Sweden^12^; NMKE, National Museums of Kenya, Nairobi^13^; OSUC, C.A. Triplehorn Insect Collection, Columbus, OH^14^; RMCA, Musee Royal de l’Afrique Centrale, Tervuren, Belgium^15^; SAMC, Iziko Museums of Cape Town, South Africa^16^; SANC, South African National Collection of Insects, Pretoria, South Africa^17^; TAMU, Texas A&M University, College Station, TX^18^; UCDC, R.M. Bohart Museum of Entomology, Davis, CA^19^; UCRC, University of California, Riverside, CA^20^; USNM, National Museum of Natural History, Washington, DC^21^.

Descriptions are presented alphabetically first by species group then by species. Species descriptions, in particular the Material Examined sections, largely follow the method of Johnson et al. (2008) and Yoder et al. (2009a) using the database application vSysLab (Johnson 2010). Polymorphic characters are indicated in descriptive statements by listing the states observed separated by semicolons. In cases where multiple specimens were associated on the same slide (historically important collections whose specimens can not easily be remounted) letters are appended to unique identifiers. In the Material Examined the specimens studied are documented in an abbreviated format, using their individual identifiers. The numbers prefixed with a letters (e.g., OSUC, CASENT) are unique identifiers for the specimens. The label data for all specimens have been georeferenced and recorded in the Hymenoptera On-Line database, and details on the data associated with these specimens can be accessed at the following link, http://hol.osu.edu, and entering the identifier in the form. Note the space between the acronym and the number. Additionally the data for all specimens is included as supplemental data in the Darwin Core Archive format.

Species groups within Afrotropical *Scelio* are quite distinct, and therefore each group is treated separately. Individual species descriptions are derived from a matrix particular to the parent species group, that is, they are cross comparable only to species from the group to which they belong. Diagnoses are usable against all other species in that group, i.e. it is already assumed that the specimen has been diagnosed to group prior to applying a given species diagnosis. All descriptive statements pertain to females only unless otherwise noted. Males were associated with females where possible, but in some cases, particularly for *walkeri*-group species, association was prevented by strong sexual dimorphism. We use a phylogenetic species concept wherein species are defined by putative autapomorphies or unique combinations of characters. Species hypotheses remain to be tested quantitatively. After identification of specimens with the key(s) readers should always refer to the Comments and Diagnoses sections for additional information and confirmation as these sections include information on morphological variability.

Abbreviations and morphological terms used in text: A1, A2, A12: antennomere 1, 2, … 12; T1, T2, T7: metasomal tergite 1, 2,7; S1, S2, … S7: metasomal sternite 1, 2, … 7. Morphological terminology otherwise follows Masner (1980) and Mikó et al. (2007).

Several morphological characters are of broad importance to *Scelio* (and often platygastrid) taxonomy. To aid and simplify reference to these we introduce the following new terms: **villus** – the strip of pilosity immediately posterior to the propodeal spiracle (Figs 7–12, *vil*), bound ventrally by the dorsal margin of the metapleuron and dorsally by the propodeal plica; **oxter** – the sloped, more or less vertical area below the pronotal shoulder (Fig. 34, *o*); **antespiracular setal patch** – small patch of microtrichia anterior to the anterior thoracic spiracle.

Appendix 1 lists terms associated with identifiers in the Hymenoptera Anatomy Ontology (Yoder et al. 2010). Identifiers in the format HAO_XXXXXXX represent concepts in the HAO version <time stamp version> and are provided to enable readers to confirm their understanding of the concepts being referenced. To find out more about a given concept use the identifier as a search term at http://portal.hymao.org. The identifier can also be used as a URI (universal resource identifier) by appending the identifier to http://purl.obolibrary.org/obo/ (e.g. http://purl.obolibrary.org/obo/HAO_0000124). URLs in the format http://purl.org/net/hao/HAO_0123456 resolve to the HAO’s community-based resource that includes additional images, notes, and other metadata.

Images were taken with a JVC 3 CCD camera (model KY-575U) attached to a Leica Z16 APO with a Planapo 1.0× objective. Specimens were illuminated with a 4 channel LED dome light from Advanced Illumination, with light levels at maximum output. Figures were produced from stacks of images that vertically transected the specimen. These were combined automatically into a single image using either Auto-Montage Pro version 5.1 or Cartograph software packages. For those images produced with Auto-Montage the resulting image was in most cases manually edited using functions within that software package. Images were post-processed for contrast and light levels in Adobe® Photoshop® or similar software.

The electronic version of the paper contains hyperlinks to external resources. Insofar as possible the external information conforms to standards developed and maintained through the organization Biodiversity Information Standards (Taxonomic Database Working Group). All new species have been prospectively registered with Zoobank (Polaszek et al. 2005, www.zoobank.org), and other taxonomic names of *Scelio*, where appropriate, have been retrospectively registered. The external hyperlinks are explicitly cited in the end notes so that users of the printed version of this article have access to the same resources. Life sciences identifiers, LSIDs, may be resolved at the specified URLs or at http://lsid.tdwg.org

## Results and discussion

Dangerfield et al. (2001) reviewed the literature on hosts for the genus. Based on our revision it is clear that the identity of some species with broad host ranges may need to be revisited as previous species may represent cryptic species complexes. Specimen data suggest that hypotheses that species of *Scelio* are restricted to hosts in the family Acrididae (Orthoptera) are confirmed: we found no evidence that species attack hosts in other families or orders. The hosts recorded on specimen label data are summarized below:

Acridinae

*Acrida madecassa* (Brancsik): *Scelio afer*

*Acrida turrita* (Linnaeus): *Scelio remaudierei*

Cyrtacanthacridinae

*Cyrtacanthacris tatarica tatarica* (Linnaeus): *Scelio zolotarevskyi*

*Kraussaria angulifera* (Krauss): *Scelio howardi*

*Nomadacris* Uvarov: *Scelio zolotarevskyi*

*Nomadacris septemfasciata* (Serville): *Scelio howardi*, *Scelio zolotarevskyi*

*Schistocerca gregaria* (Forskål): *Scelio zolotarevskyi*

Eyprepocneminae

*Eyprepocnemis plorans plorans* (Charpentier): *Scelio howardi*, *Scelio remaudierei*, *Scelio zolotarevskyi*

*Eyprepocnemis smaragdipes* Bruner: *Scelio zolotarevskyi*

*Heteracris* Walker: *Scelio zolotarevskyi*

*Heteracris zolotarevskyi* Dirsh: *Scelio tritus*

Oedipodinae

*Acrotylus* Fieber: *Scelio zolotarevskyi*

*Acrotylus patruelis* (Herrich-Schaeffer): *Scelio zolotarevskyi*

*Gastrimargus africanus* (Saussure): *Scelio zolotarevskyi*

*Hilethera nigerica* (Uvarov): *Scelio remaudierei*

*Locusta* Linnaeus: *Scelio zolotarevskyi*

*Locusta migratoria* (Linnaeus): *Scelio ipomeae*

*Locusta cinerascens migratorioides* (Reiche & Fairmaire): *Scelio remaudierei*, *Scelio zolotarevskyi*

*Locusta cinerascens capito* Saussure: *Scelio zolotarevskyi*

*Oedaleus virgula* (Snellen van Vollenhoven): *Scelio zolotarevskyi*

Oxyinae

*Oxya* Serville: *Scelio philippinensis*

These host records are concentrated on those species of acridids of the greatest economic importance, undoubtedly a reflection more of the interest in plague locusts than in the biology of the parasitoids. The host records span five of the 16 subfamilies of Acrididae recorded from Africa and Madagascar in Orthoptera Species File (Eades et al., Accessed 6 Dec 2013). We have but a single rearing from the desert locust, *Schistocerca gregaria* (Forskål), despite the great attention paid to the biology and control of this pest. This contrasts with 9 collecting events and 65 specimens of *Scelio* from the New World that have been reared from other species of *Schistocerca*. This pattern is consistent with the greater number of species of *Schistocerca* in the New World, 36 species versus a single species in the Old World, as well as the conclusion of Song (2004) that *Schistocerca gregaria* originated in the Americas. The species recorded from the desert locust, *Scelio zolotarevskyi*, has been reared from the eggs of nine genera.

We define several new species group, and refine those proposed by Nixon (1958). In his key Nixon (1958) mentioned three groups, *pulchripennis*, *howardi* and *walkeri*. To these we add the *irwini*, *ipomeae*, and *simoni*-groups, and split the *ernstii*-group from the *howardi*-group of Nixon. Our goal in doing this is not to assert anything about the phylogenetic history of the genus, but to provide an initial structure for interpreting and understanding the diveristy of species and characters in *Scelio*. The key to species groups is robust, at least based on the specimens we have studied, and the description of each of the groups is provided to give a better understanding of the range of variation to be found.

Dangerfield et al. (2001), in an analysis of 61 predominantly Australian species based on morphological characters, found a very high degree of homoplasy and little internal phylogenetic structure. They did not formally circumscribe clades, but they did recover the *walkeri* species group as monophyletic (fig. 6.2, node 8, number is erroneously placed one branch too high). We have not quantitatively tested the presently defined species groups on a species-level basis, however we are relatively confident that they represent monophyletic units. Our species group definitions depend heavily on characters of posterior propodeum, features that were only cursorily treated in Dangerfield et al. (2001). Relationships among the species groups remain unclear.

**Figures 1–6.**
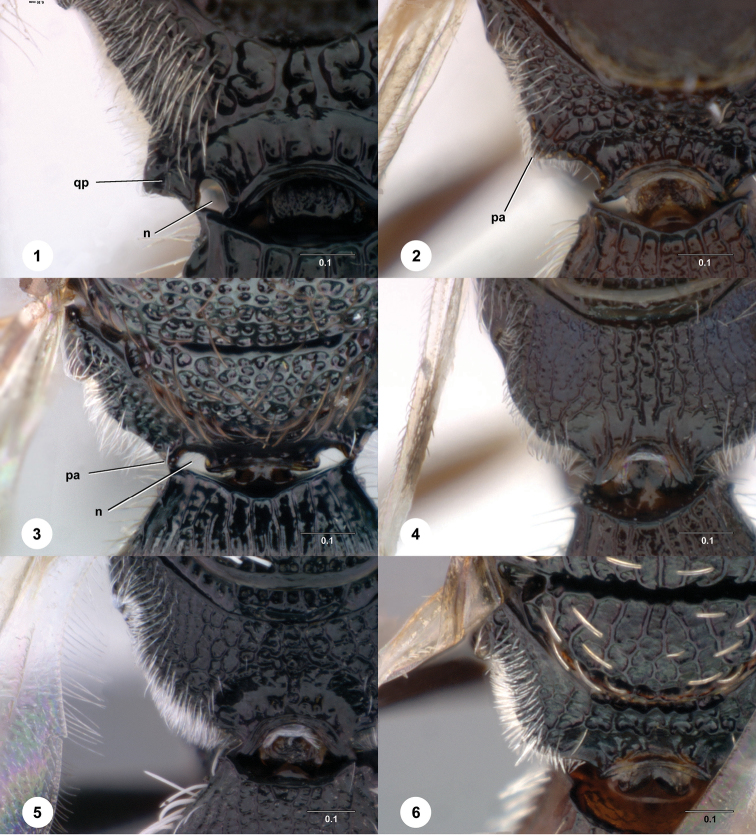
^114^ Female, propodeum, dorsal view. **1** *Scelio zolotarevskyi* Ferrière (OSUC 212373) **2** *Scelio aphares* sp. n. (OSUC 21364) **3** *Scelio simoni* sp. n. (OSUC 213634) **4** *Scelio nitens* Brues (OSUC 212091) **5** *Scelio tritus* sp. n. (CASENT 2134010) **6** *Scelio ipomeae* Risbec (OSUC 213627). *n*, notch; *pa*, propodeal angle; *qp*: quadrate plate. Scale bars in millimeters.

**Figures 7–12.**
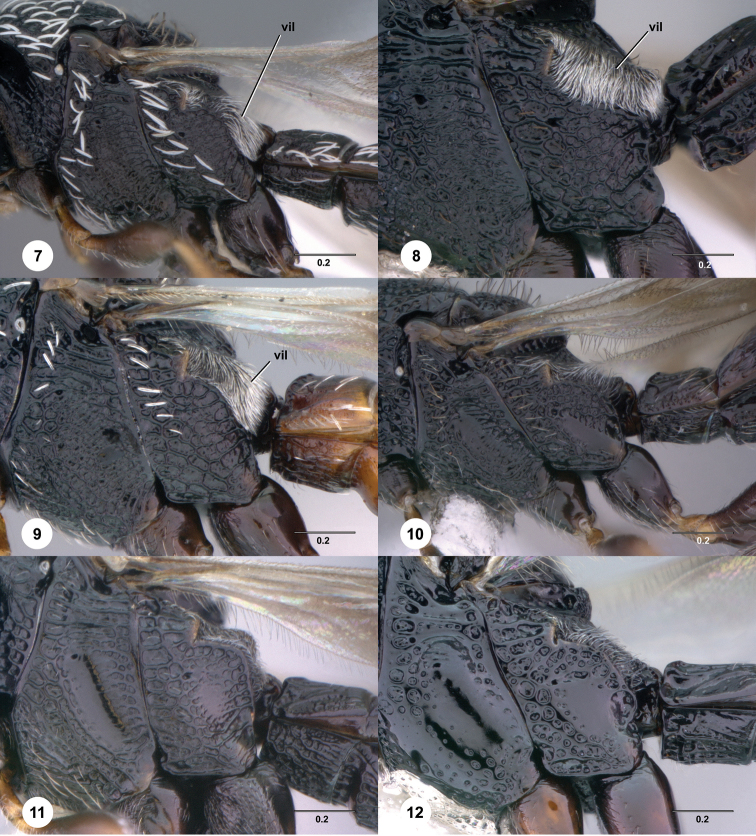
^115^ Female, metapleuron, lateral view. **7** *Scelio afer* (Kieffer) (OSUC 212366) **8** *Scelio taylori* Nixon (OSUC 213533) **9** *Scelio ipomeae* Risbec (OSUC 213040) **10** *Scelio simoni* sp. n. (OSUC 250874) **11** *Scelio parkeri* sp. n. (CASENT 2042035) **12** *Scelio effervesco* sp. n. (CASENT 2134281). *vil*, villus. Scale bars in millimeters.

**Figures 13–18.**
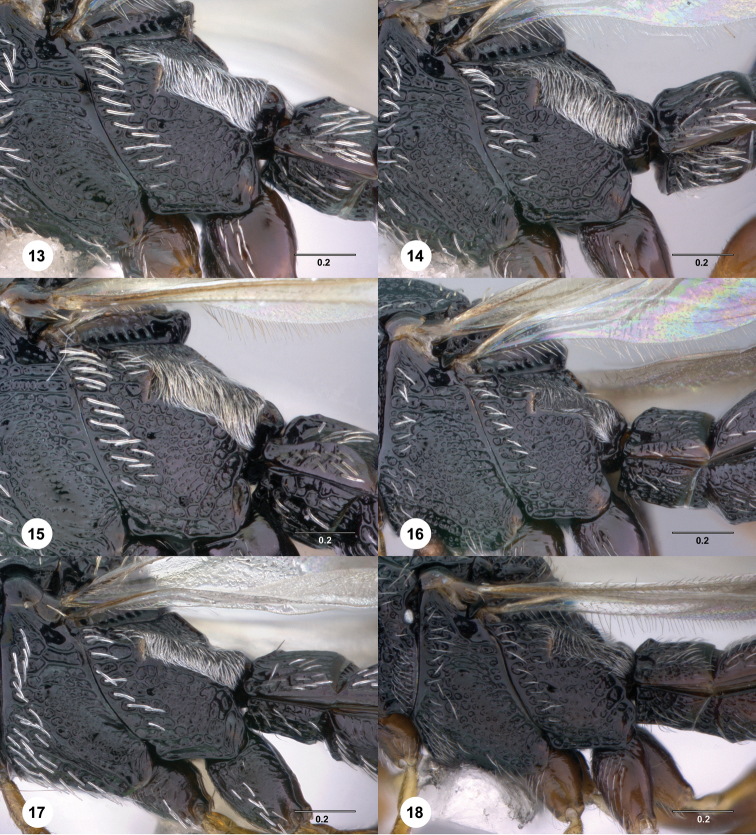
^116^ Female, metapleuron, lateral view. **13** *Scelio howardi* Crawford (OSUC 171121) **14** *Scelio zolotarevskyi* Ferrière (OSUC 164165) **15** *Scelio scomma* sp. n. (CASENT 2042253) **16** *Scelio igland* sp. n. (CASENT 2132900) **17** *Scelio destico* sp. n. (OSUC 214085) **18** *Scelio philippinensis* Ashmead (OSUC 213315). Scale bars in millimeters.

**Figures 19–24.**
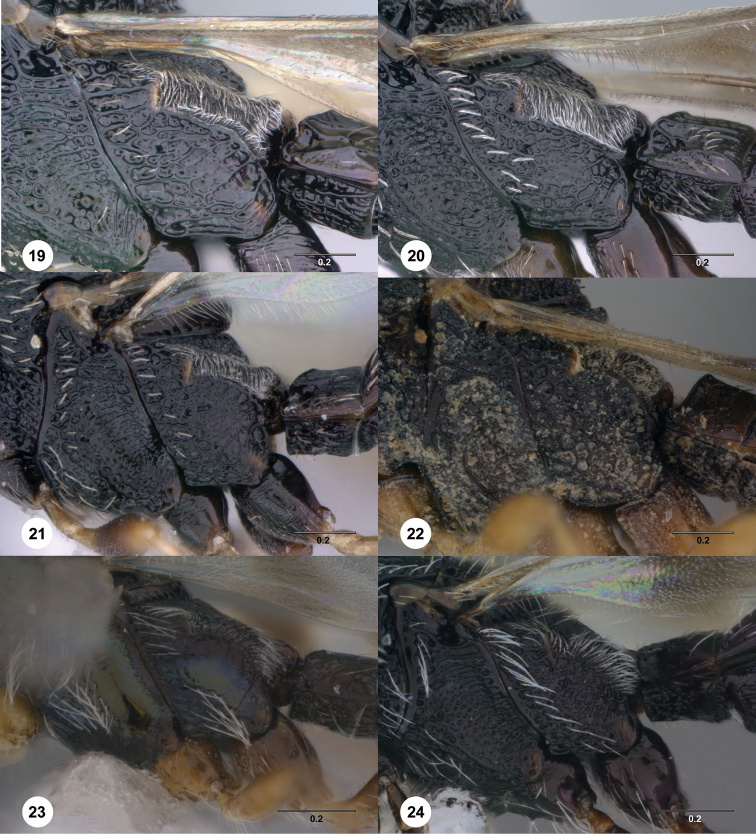
^117^ Female, metapleuron, lateral view. **19** *Scelio gemo* sp. n. (OSUC 212932) **20** *Scelio pipilo* sp. n. (OSUC 244025) **21** *Scelio latro* sp. n. (OSUC 250953) **22** *Scelio memorabilis* sp. n. (OSUC 244026) **23** *Scelio nitens* Brues (OSUC 212091) **24** *Scelio parapulchripennis* Yoder (CASENT 2042134). Scale bars in millimeters.

**Figures 25–30.**
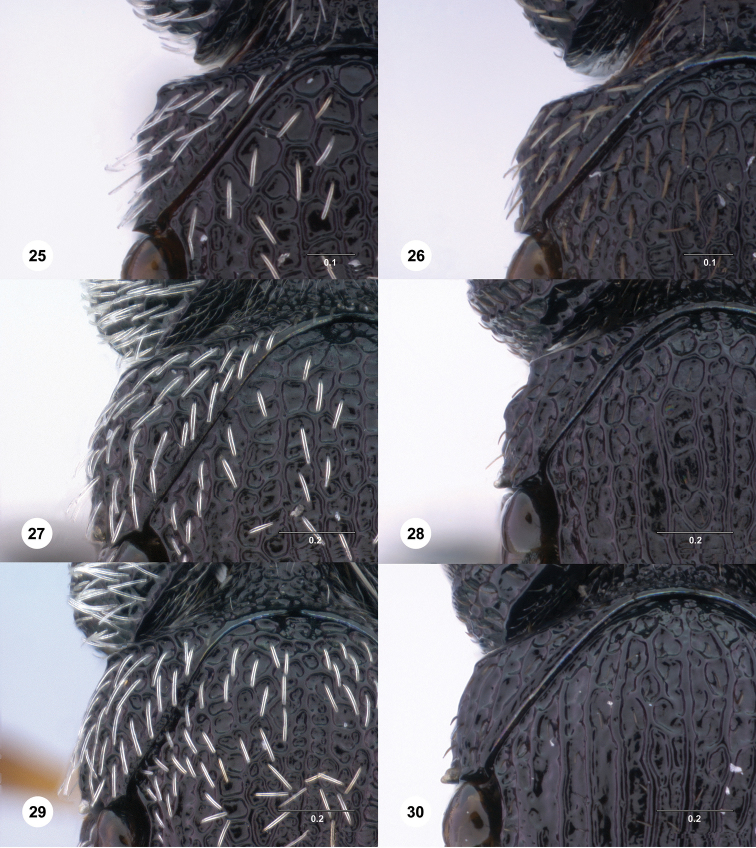
^118^ Female, anterior mesosoma, dorsal view. **25** *Scelio destico* sp. n. (OSUC 212612) **26** *Scelio latro* sp. n. (OSUC 212968) **27** *Scelio scomma* sp. n. (CASENT 8106513) **28** *Scelio susurro* sp. n. (OSUC 213654) **29** *Scelio zolotarevskyi* Ferrière (CASENT 2043560) **30** *Scelio grunnio* sp. n. (OSUC 214367). Scale bars in millimeters.

Afrotropical *Scelio* species show a full range of geographical distribution patterns, from locally endemic to transcontinental. We know of no species that are shared with the New World fauna or Australia. Species overlap between Afrotropical and the Palearctic, South, and Southeast Asian regions occurs in several cases in the *walkeri* and *howardi*-group species. Of the 11 species of Madagascar, only two are known from continental Africa.

We found that many species or species groups of Afrotropical *Scelio* are morphologically cryptic. For at least some of these groups, in particular the *walkeri* and *howardi*-groups, we believe that resolution of species will require a molecular barcoding approach. Extensive effort has been made to find the species limits, and this has been accomplished with varying degrees of confidence. While new specimens, more extensive collecting, and the addition of host or biological information will provide further clues, we feel that it is not only helpful but prudent to begin to include additional character systems (molecular) in the future. Those planning revision should take care to maintain the material in a state which enables molecular processing, i.e. specimens should not be immediately dried and mounted, but rather kept refrigerated in ethanol until a first pass can be made to remove exemplars for sequencing. Our recommendations of this approach is not a blanket endorsement of molecular barcoding per se, but rather a plea for consideration of additional sources of character data.

### Key to Afrotropical species groups of *Scelio*

With only a few exceptions species of Afrotropical *Scelio* are easily classified into larger groups. The *pulchripennis* species group is included in the species group key for completeness; a key to the species of the *pulchripennis* group may be found in Yoder et al. (2009b). The species group key is specific to females, although males of many species can successfully be identified as well. No more than 60× magnification should be necessary to use the key. Characters within parentheses are not necessarily diagnostic, but are included as an aid in confirmation of the identification. A digital version of the identification key is available at http://www.waspweb.org/Platygastroidea/Keys/index.htm

**Table:** 

1	Posterolateral margin of propodeum (seen in dorsal view) projecting posteriorly into a sharp angle (Figs 2, 3, *pa*) or quadrate plate (Fig. 1, *qp*)	2
–	Posterolateral margin of propodeum broadly rounded (Figs 4–6)	3
2	Posterior propodeum produced into single point (or appearing as a truncate lateral corner) (Fig. 2, *pa*), no semicircular notch present separating projection from propodeal nucha (most species with fine reticulate sculpture at base of mandible)	*ernstii* species group
–	Posterior propodeum produced into quadrate lateral projection (Fig. 1, *qp*), projection separated from propodeal nucha by deep semi-elliptical notch between inner margin and propodeal nucha (Fig. 1, *n*), posteroventral metapleuron sparsely setose to glabrous (Fig. 19)	*howardi* species group
3	Mesoscutum sculptured throughout with slightly flattened rounded-reticulate sculpture that appears punctate-foveate in places (Figs 313, 319, 325); pilosity throughout mesonotum brown, fine, and long (Fig. 326); netrion glabrous (Fig. 326) (anteclypeus coming to a broad point medially; wings not pictate; male RSS present; South Africa, Western Cape)	*simoni* species group
–	Mesoscutum variously sculptured, but never punctate-foveate throughout, typically reticulate-striate or strigose, sometimes smooth; pilosity of mesonotum variable, if long, fine and brown, then netrion setose; netrion glabrous or setose	4
4	Anteclypeus between medial teeth wide and slightly concave to truncate, with isolated projection medially, lateral projections well developed, pointed, often oriented outwards; netrion setose, pilosity reduced to anterior margin in some; head and mesosoma sometimes with metallic coloration; mesosoma with long semi-erect to erect setae scattered throughout; females with wings pictate (infuscate bands); mandibles narrowed, often appearing apically unidentate but never truly so, with at least a very reduced second tooth present (male A5 without RSS and nearly always with hyaline/white wings)	*pulchripennis* group species, see Yoder et al. (2009b)
–	Anteclypeus between medial teeth projected and truncate or evenly concave, without a isolated projection medially, lateral projections variously formed; netrion glabrous; head and mesosoma never with metallic coloration; mesosomal pilosity not elongate and erect; wings without distinct bands, typically evenly infuscate throughout; mandibles clearly bidentate apically, teeth not reduced	5
5	Pilosity throughout very short, fine, sparse and appressed (e.g., Figs 287, 293, 305); villus narrow, more or less equal width throughout (e.g. Fig. 11); frons usually with percurrent arcuate striae in lower half (e.g. Figs 291, 297); females with medial T3–T4 (Figs 292, 304) and S2–S5 usually with smooth patches; (in lateral view gena broad, somewhat flattened and with narrow occipital carina prominent; Madagascar)	*irwini* species group
–	Pilosity moderate length, setae variable in width, but predominantly thicker and moderately dense; villus thick, broadening posteriorly (e.g. Fig. 9); frons rarely with percurrent arcuate striae in lower half, sculpture usually interrupted medially, reticulate to rugulose (e.g. Figs 255, 267, 273); females with medial T3–T4 usually sculptured, medially S2–S5 variably sculptured or with smooth patch; (continental Africa, Madagascar)	6
6	Basal tooth on mandible present (Fig. 375, *bt*); metasoma dark brown to black, concolorous with or very slightly lighter than mesosoma, never orange or yellow; male RSS on A5 absent or apparently so; (body with at least some setae that are thickened and most commonly white (see Figs 329–406), or uncommonly golden, or brown; thickened white pilosity typically present on gena, pronotal shoulder and meso- and metapleural anterodorsal margins, tips of pilosity truncated or not)	*walkeri* species group
–	Basal tooth on mandible nearly always absent, if present then metasoma orange; metasoma orange (most species) to brown (a single species), never dark brown to black, nearly always lighter than mesosoma; male RSS on A5 present	*ipomeae* species group

### Checklist and species-group placement of the Afrotropical *Scelio* species

*Scelio ernstii* species group

*Scelio albatus* Yoder, sp. n.

*Scelio aphares* Yoder, sp. n.

*Scelio ardelio* Yoder, sp. n.

*Scelio bayanga* Yoder, sp. n.

*Scelio chapmani* Nixon

*Scelio copelandi* Yoder, sp. n.

*Scelio dupondi* Yoder, sp. n.

*Scelio exophthalmus* Yoder, sp. n.

*Scelio janseni* Yoder, sp. n.

*Scelio mauritanicus* Risbec

= *Scelio cahirensis* Priesner, syn. n.

*Scelio phaeoprora* Yoder, sp. n.

*Scelio taylori* Nixon

*Scelio howardi* species group

“white” subgroup

*Scelio fremo* Valerio & Yoder, sp. n.

*Scelio howardi* Crawford

= *Scelio aburiensis* Crawford

*Scelio igland* Yoder, sp. n.

*Scelio ructo* Yoder, sp. n.

*Scelio scomma* Yoder, sp. n.

*Scelio ululo* Yoder, sp. n.

*Scelio zolotarevskyi* Ferrière

= *Scelio cheops* Nixon, syn. n.

= *Scelio sudanensis* Ferrière, syn. n.

“brown” subgroup

*Scelio balo* Valerio & Yoder, sp. n.

*Scelio bubulo* Yoder, sp. n.

*Scelio cano* Yoder, sp. n.

*Scelio crepo* Yoder, sp. n.

*Scelio exaratus* (Kieffer)

*Scelio gemo* Yoder, sp. n.

*Scelio grunnio* Yoder, sp. n.

*Scelio latro* Yoder, sp. n.

*Scelio mutio* Yoder, sp. n.

*Scelio susurro* Yoder, sp. n.

*Scelio tono* Yoder, sp. n.

*Scelio tristis* Nixon

*Scelio effervesco* Yoder, sp. n.

*Scelio destico* Yoder, sp. n.

*Scelio memorabilis* Yoder, sp. n.

*Scelio philippinensis* Ashmead

*Scelio pipilo* Yoder, sp. n.

*Scelio ipomeae* species group

*Scelio aurantium* Yoder, sp. n.

*Scelio impostor* Yoder, sp. n.

*Scelio ipomeae* Risbec

*Scelio ntchisii* Yoder, sp. n.

*Scelio somaliensis* Yoder, sp. n.

*Scelio transtrum* Yoder, sp. n.

*Scelio irwini* species group

*Scelio harinhalai* Yoder, sp. n.

*Scelio irwini* Yoder, sp. n.

*Scelio obscuripennis* Johnson, n.n.

= *Lepidoscelio fuscipennis* Kieffer, 1905 (*nec* *Scelio fuscipennis* Ashmead, 1887)

*Scelio parkeri* Yoder, sp. n.

*Scelio pulchripennis* species group (see Yoder et al. 2009b)

*Scelio antorides* Nixon

*Scelio baoli* Risbec

*Scelio clarkei* Yoder

*Scelio corion* Nixon

*Scelio ememeye* Yoder

*Scelio habilis* Nixon

*Scelio leipo* Yoder

*Scelio marbis* Nixon

*Scelio masneri* Yoder

*Scelio nisa* Kozlov

*Scelio nitens* Brues

*Scelio paranitens* Yoder

*Scelio parapulchripennis* Yoder

*Scelio poecilopterus* Priesner

= *Scelio princeps* Nixon

*Scelio pulchripennis* Brues

*Scelio tria* Yoder & Masner

*Scelio turbidus* Yoder

*Scelio variegatus* Kozlov & Kononova

*Scelio simoni* species group

*Scelio simoni* Yoder, sp. n.

*Scelio simonolus* Yoder, sp. n.

*Scelio vannoorti* Valerio & Yoder, sp. n.

*Scelio walkeri* species group

*Scelio afer* (Kieffer)

= *Scelio clarus* Fouts, syn. n.

= *Scelio africanus* Risbec, syn. n.

*Scelio apospastos* Yoder, sp. n.

*Scelio clypeatus* Yoder, sp. n.

*Scelio concavus* Yoder, sp. n.

*Scelio erugatus* Yoder, sp. n.

*Scelio modulus* Yoder, sp. n.

*Scelio pilosilatus* Yoder, sp. n.

*Scelio quasiclypeatus* Yoder, sp. n.

*Scelio remaudierei* Ferrière

*Scelio retifrons* Yoder, sp. n.

*Scelio striatus* Priesner

= *Scelio gaudens* Nixon, syn. n.

*Scelio tritus* Yoder, sp. n.

### *Scelio ernstii* species group

**Description.** *General*. Body size: moderate; large. Body length: 3.12–5.35 mm). Habitus: typical, mesosoma not dorsoventrally flattened. Body color: brown to dark brown. Fore leg color: concolorous with mid and hind legs. Sculpture: moderate to robust, reticulate to strigose, generally with some longitudinal or parallel lineations. Wing type: macropterous.

*Pilosity*. General setation: moderate elongate and wide, variously vertically oriented. Thickened and truncate white pilosity: not typically present, or strongly truncate when so. Interommatidial pilosity: present; absent. Genal pilosity density: sparse; moderate; dense. Genal pilosity color: white; brown. Number of anteclypeal setal pairs: 3, with fine and much smaller fourth laterally. Ventrolateral postgenal cluster of erect setae: unknown. Antespiracular setal patch: very small, intersected by or immediately below lateral epomia. Netrion: glabrous. Propodeal shelf: narrow strip present laterally. Pilosity of laterotergites: absent.

*Head*. Sculpture of head: predominantly reticulate to rugulose throughout, never predominantly dorsoventral or longitudinal, without prominent smooth or obliterated patches, carinae moderate width. Ocelli size: moderate; large. Gap between antennal toruli and anteclypeus: narrow to moderate width. Width of ventral head across mandibles: narrow, mandibles relatively compact. Anteclypeus shape between outer teeth: thin immediately mesad of outer teeth, smoothly rounded to slightly trapezoidal medially, without sharp vertices, medially truncate to slightly concave. Malar sulcus: present. Medial portion of occipital carina: percurrent. Lateral portion of occipital carina: more or less linear throughout. Form of gena: narrow (lateral view), strongly sloped from posterior margin of eye to occipital carina; moderate width (lateral view), evenly rounded from posterior margin of eye to occipital carina. Genal carina: absent; present. RSS on A5 in males: present. Microsculpture at base of mandible: present; absent. Basal tooth of mandible: absent.

*Mesosoma*. Shape of mesoscutum in lateral view: bulging in anterior such that anterior sloping towards pronotum; moderately concave, not particularly bulging in anterior nor flattened throughout. Transverse pronotal carina in female: unknown. Surface of mesoscutum: sculptured throughout, never with smooth or obliterated patches, reticulate laterally and anteromedially, longitudinally striate to strigose posteromedially. Smooth or obliterated patches on mesoscutum: absent. Surface of the pronotal collar in females: unknown. Axillula: small, clearly discernible only in lateral view. Propodeal corners: truncate, with a single pointed vertex laterally, posterior edge more or less straight. Epomia: present. Surface of oxter: unknown. Fore wing length: reaching or surpassing anterior margin of T5 but not surpassing apex of metasoma. Fore wing submarginal vein near curve towards costal margin: nebulous to spectral; tubular. Pictation of fore wing in female: absent.

*Metasoma*. Anterior margin of T1: concave, with short rim.

**Diagnosis.** With one exception (*Scelio ardelio*) these species are easily distinguished from all other African *Scelio* by the angular corners of the posterolateral propodeum (Fig. 2).

**Comments.** The Afrotropical members of the cosmopolitan *ernstii* species group can be divided into two subgroups based on the presence or absence of the genal carina. Within these two subgroups the differences between species are very subtle and we expect several of our species (*Scelio aphares* and *Scelio taylori* in particular) to ultimately represent groupings of cryptic species. Nixon’s (1958) insights as to important characters for species in this group (he treats *Scelio taylori*, *Scelio chapmani*, and *Scelio mauritanicus*) are particularly helpful.

**Key to *ernstii*-group species** (also available online at http://www.waspweb.org/Platygastroidea/Keys/index.htm)

**Females**

**Table:** 

1	Metapleuron with a large patch of fine setae immediately above the hind coxa; scape and legs bright yellow (Fig. 58) (males similarly setose)	2
–	Metapleuron without or with only scattered sparse setae above the hind coxa (e.g. Figs 64, 70, 100)	3
2	Gena with dense white, somewhat thickened pilosity (Fig. 58); posterior propodeal margin with distinct angular corner (Fig. 57); patch of setae above hind coxa predominantly oriented posteriorly (Fig. 60); mesoscutum sculptured throughout (Fig. 57)	*Scelio chapmani*
–	Gena with sparse, brown, thin pilosity; posterior propodeal margin appearing rounded and without truncate margin, with only extremely short straight edge (Fig. 45); patch of setae above hind coxa predominantly oriented ventrally (Fig. 46); mesoscutum with a narrow strip of obliterated sculpture along humeral margin (Fig. 45)	*Scelio ardelio*
3	Lateral portions of T2–T5 glabrous in anterior half, posterior half with dense, thick, white setae (Figs 68, 80)	4
–	T2–T5 laterally with pilosity more or less evenly distributed throughout length of sclerites, pilosity frequently sparse, and most often thin and brown (e.g. Figs 62, 98)	5
4	Metasoma narrow (Fig. 67); sculpture of S3–S6 fine, relatively compact and predominantly longitudinal (Fig. 72); S3 medially sculptured more or less throughout, at most with a thin band of obliterated sculpture along midline (Fig. 72); S2 felt field extremely reduced, pinch-like and not strongly elevated; scape bright yellow; body often with metasoma lighter (brown) than mesosoma	*Scelio dupondi*
–	Metasoma broad (Fig. 79); sculpture of S3–S6 longitudinal but somewhat irregular, particularly at base of S2 which is rugulose, with larger gaps between interstices (Fig. 84); S3 medially smooth, with sculpture more or less absent throughout (Fig. 84); S2 felt field well developed and elevated on a keel-like projection; scape dark golden yellow; body often concolorous and dark brown throughout	*Scelio janseni*
5	Gena in lateral view narrow, with three well-defined carinae present (orbital, genal, occipital, Fig. 102: *orbc*, *gc*, *occ*) at narrowest point, and frequently genal well developed throughout; gena with sparse, shorter and usually brown but sometimes off-white setae whose tips are not typically overlapping; never with fine pilosity at base of lateral T1 (eye in lateral view large)	6
–	Gena in lateral view broad or without a single distinctly larger genal carina between orbital and occipital carinae (e.g. Fig. 88); gena with thick white setae whose tips may or may not overlap adjacent setae; often with micropilosity at base of lateral T1	7
6	Pilosity on the mesonotum, lateral gena, and pronotal shoulder completely white (Figs 39, 40); reticulations of the mesoscutum relatively fine and somewhat more dense (Fig. 39); villus elongate, narrow, and weakly curved ventrally; scape dark brown (South Africa)	*Scelio albatus*
–	Pilosity on the mesoscutum brown, on gena white or brown, on pronotal shoulder white and brown; reticulations of the mesoscutum relatively robust, and larger (Fig. 99); villus variably developed, but often somewhat thicker (Fig. 100); scape yellow or brown, only rarely dark brown (these individuals large and dark) (widespread; there are three general forms that key here, see Comments)	*Scelio taylori*
7	Pronotal nucha smooth throughout, nearly glabrous (Fig. 87, *pn*); mesoscutum more or less flat; dorsal head between lateral ocelli with some very slightly polished and bulging areas with irregular microsculpturing typically in the form of punctures; scape yellow in basal, becoming brown towards apex, rarely completely yellow and then legs beyond coxae concolorous throughout	*Scelio mauritanicus*
–	Pronotal nucha with at most a small smooth and glabrous patch anteriorly, if small smooth patch present then bounded laterally with slight transverse sculpture that fades medially; dorsal head between lateral ocelli variably developed, but typically sculptured throughout; mesoscutum usually bulging slightly in anterior third; scape entirely yellow or nearly entirely brown with at most slightly yellow base or apex	8
8	Pilosity of dorsal pronotal shoulder short and thicker, predominantly brown, concolorous with dorsal mesonotal setae (Fig. 51); mesonotum very robustly sculptured, with large cells (Fig. 51); gena in lateral view very broad (Central African Republic, single female)	*Scelio bayanga*
–	Pilosity of dorsal pronotal shoulder elongate, predominantly white or off-white, typically lighter than dorsal mesonotal setae, often distinctly so; mesonotum variously sculptured, with smaller more compact cells; gena in lateral view of moderate width	9
9	Frons below anterior ocellus with brown pilosity continuing to near lower margin of eye, pilosity below this white (Fig. 95); A3 bright yellow; pilosity of frons somewhat sparse, setae thick, and slightly truncate (Fig. 95); (frons somewhat narrowed between eyes; metasoma relatively depressed; Ivory Coast)	*Scelio phaeoprora*
–	Frons below anterior ocellus at most with few brown setae immediately adjacent to ocellus, setae otherwise white (e.g. Figs 65, 77); A3 brown to dark brown; pilosity of frons variously developed, truncate or pointed	10
10	Gena with dense, bushy pilosity (Fig. 78); eye bulging towards anterior (Figs 75, 78); A1 yellow, A3 onwards light brown to brown (T3 and often T2 with tendency for more reticulation in sculpture)	*Scelio exophthalmus*
–	Genal pilosity not distinctly bushy; eye not distinctly bulging in lateral view; A1 yellow or brown, A3 onwards varying	11
11	Sculpture of face relatively fine, with dorsoventral trend, with large interstices between carinae (Fig. 65); sculpture of mesonotum well-impressed, fine, somewhat longitudinal, particularly on medial mesoscutellum (Fig. 63); scape brown (villus often slightly concave ventrally and relatively narrow (Fig. 64, *vil*); head ovoid; sculpture of lateral margins of metasomal tergites very slightly obliterated or smoothed in parts; darker individuals)	*Scelio copelandi*
–	Sculpture of the face variable, but in general reticulate, without dorsoventral trend, and typically somewhat denser (Fig. 35); sculpture of mesonotum moderately impressed, relatively densely reticulate (Fig. 36); scape brown or yellow; villus variously formed (see Comments for information on the several morphotypes presently included in this relatively polymorphic species)	*Scelio aphares*

#### Scelio albatus

Yoder sp. n.

http://zoobank.org/05691BDD-30A4-476C-B05B-9B49539DC10B

urn:lsid:biosci.ohio-state.edu:osuc_concepts:244957

http://species-id.net/wiki/Scelio_albatus

**Figures 31–36.**
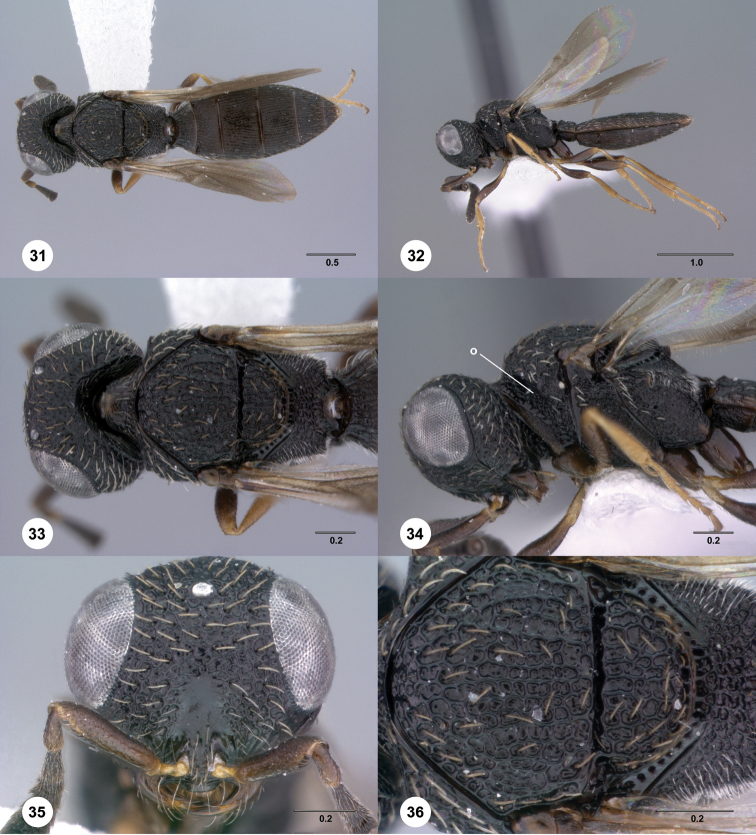
^119^ *Scelio aphares* sp. n., holotype female (OSUC 212250). **31** Habitus, dorsal view **32** Habitus, lateral view **33** Head and mesosoma, dorsal view **34** Head and mesosoma, lateral view **35** Head, anterior view **36** Mesonotum, dorsal view. *o*, oxter. Scale bars in millimeters.

Figures 31–36 ; Morphbank ^22^

##### Description.

Female body length: 3.52–3.64 mm (n=2). Male body length: 3.40-4.48 mm (n=2). Shape of compound eye: not or only slightly bulging. Color pattern of pilosity below anterior ocellus in female: predominantly white throughout. Sculpture of frons in female: reticulate rugulose, sculpture finer, typically without dorsoventral trend. Genal carina in female: present. Width of gena in lateral view: weakly expanded, posterior margin parallel to posterior margin of eye. Density of genal setae: moderately to highly dense, setae conspicuous. Color of genal setae: white to off-white. Sculpture of base of mandible in female: minutely reticulate. Color of A1 in female: light to dark brown throughout, or with apex and base slightly lighter, often yellowish. Color of A3 in female: brown. Sculpture of dorsal pronotal nucha in female: predominantly to completely sculptured. Color of pilosity of pronotal shoulder in female: predominantly white to off-white. Sculpture along humeral margin of mesoscutum: well-defined throughout. Color of pilosity of mesonotum in female: predominantly white to off-white. Transition from lateral to posterior margin of propodeum in dorsal view: forming distinct angle, corner of propodeum well defined. Shape of mesoscutum in lateral view: more or less flat. Pilosity on metapleuron above hind coxa: glabrous or with few scattered setae. Form of fore wing submarginal vein in female: tubular throughout from base to costal margin. Fine pilosity of lateral T1 in female: absent. Width of metasoma: moderately wide, width of S3 1.5–1.6 times medial length. Distribution of pilosity on T2–T5 in female: more or less evenly distributed throughout. Sculpture of T3 in female: longitudinally striate throughout. Overall sculpture of S3: with dense, fine longitudinal carinae. Sculpture of medial S3 in female: present throughout.

##### Diagnosis.

Similar to *Scelio taylori* which shares the presence of the well-developed genal carina (cf. Figs 102, 108, 114, *gc*). Differing from all individuals of *Scelio taylori* by the the completely white pilosity of the mesoscutum (and throughout body), the fine and relatively dense reticulations of the mesoscutum (cells larger, more robust in *Scelio taylori*, compare Figs 39 and 99) and, in most individuals, by the color of A1 (only rarely dark brown in *Scelio taylori*, and these individuals are distinctly larger and darker throughout).

**Figures 37–42.**
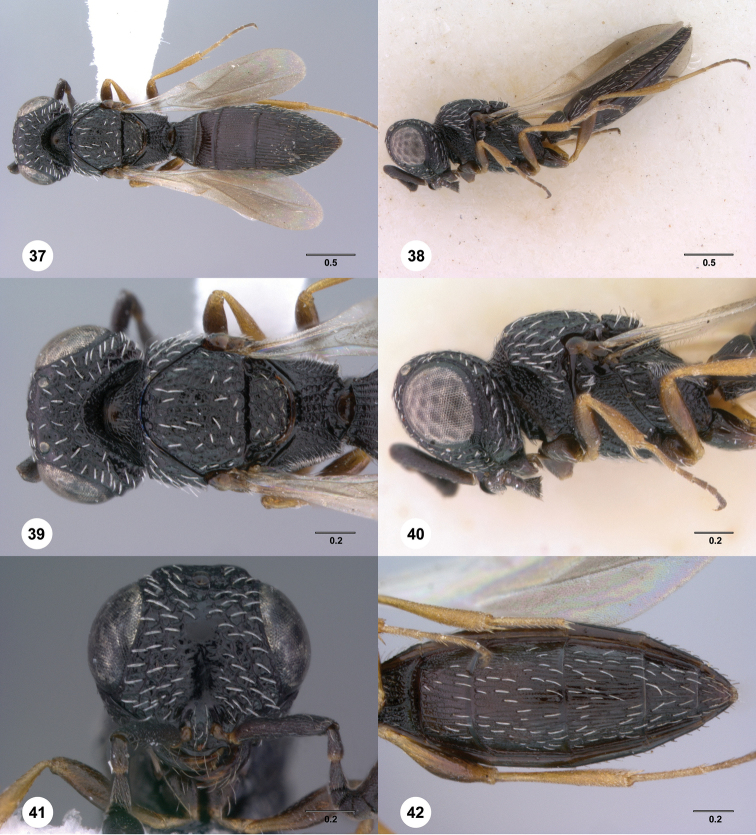
^120^ *Scelio albatus* sp. n., female. **37–40, 42** holotype female (OSUC 213338), 41 paratype female (OSUC 213481). **37** Habitus, dorsal view **38** Habitus, lateral view **39** Head and mesosoma dorsal view **40** Head and mesosoma lateral view **41** Head, anterior view **42** Metasoma, ventral view. Scale bars in millimeters.

##### Etymology.

The epithet is a Latin participle, meaning clothed in white, in reference to the pilosity of the mesoscutum.

##### Link to Distribution Map.

http://hol.osu.edu/map-large.html?id=244957

##### Material Examined.

*Holotype*, female: **SOUTH AFRICA**: KwaZulu-Natal Prov., Dukuduku forest Reserve, IV-1977, G. L. Prinsloo, OSUC 213338 (deposited in SANC). *Paratypes*: **SOUTH AFRICA**: 1 female, 2 males, OSUC 213393–213394, 213481 (SANC).

##### Comments.

*Scelio albatus* is clearly closely related to *Scelio taylori* and likely represents a geographically isolated extension of that species, being the only specimens of the larger clade from South Africa. *Scelio taylori* exhibits a fair amount of morphological variation, but is not noted for variability in the the color of pilosity on the mesoscutum. In general white pilosity on the mesoscutellum is rare for Afrotropical *Scelio*. The sculpture of the mesoscutum is more irregular and the course of the notauli is more visible than seen in *Scelio taylori*.

#### Scelio aphares

Yoder sp. n.

http://zoobank.org/E62D031E-5727-418D-92BE-46131F02EA65

urn:lsid:biosci.ohio-state.edu:osuc_concepts:244947

http://species-id.net/wiki/Scelio_aphares

Figures 2 , 31–36 ; Morphbank ^23^

##### Description.

Female body length: 3.18–4.21 mm (n=16). Shape of compound eye: not or only slightly bulging. Color pattern of pilosity below anterior ocellus in female: predominantly white throughout. Sculpture of frons in female: reticulate rugulose, sculpture finer, typically without dorsoventral trend. Genal carina in female: absent. Width of gena in lateral view: weakly expanded, posterior margin parallel to posterior margin of eye. Density of genal setae: moderately to highly dense, setae conspicuous. Color of genal setae: white to off-white. Sculpture of base of mandible in female: minutely reticulate. Color of A1 in female: yellow throughout; light to dark brown throughout, or with apex and base slightly lighter, often yellowish. Color of A3 in female: brown. Sculpture of dorsal pronotal nucha in female: predominantly to completely sculptured. Color of pilosity of pronotal shoulder in female: predominantly white to off-white. Sculpture along humeral margin of mesoscutum: well-defined throughout. Color of pilosity of mesonotum in female: predominantly light to dark brown. Transition from lateral to posterior margin of propodeum in dorsal view: forming distinct angle, corner of propodeum well defined. Shape of mesoscutum in lateral view: bulging in anterior third. Pilosity on metapleuron above hind coxa: glabrous or with few scattered setae. Form of fore wing submarginal vein in female: tubular throughout from base to costal margin. Fine pilosity of lateral T1 in female: present. Width of metasoma: very wide, width of S3 > 2 times medial length. Distribution of pilosity on T2–T5 in female: more or less evenly distributed throughout. Sculpture of T3 in female: longitudinally striate throughout; longitudinally striate laterally with prominent reticulate to rugulose elements medially. Overall sculpture of S3: with dense, fine longitudinal carinae. Sculpture of medial S3 in female: present throughout; with broadly obliterated or with distinct smooth patch.

##### Diagnosis.

Similar to other Afrotropical *ernstii*-group species without a genal carina. Most similar to *Scelio copelandi* but differing from this species by slightly more compact reticulations of the mesonotum (compare Figs 36 and 65), and the slightly more reticulate sculpture of the face (trending to dorsoventrally striate in *Scelio copelandi*).

##### Etymology.

The epithet is used as a noun in apposition derived from the Greek word for unclad or naked, in reference to the confusingly plain and relatively unremarkable species.

##### Link to distribution map.

http://hol.osu.edu/map-large.html?id=244947

##### Material examined.

*Holotype*, female: **SOUTH AFRICA**: Limpopo Prov., 30km W Trichardtsdal, Down’s forest, 1350 m, 30.XII.1985, sweeping, M. Sanborne, OSUC 212250 (deposited in CNCI). *Paratypes*: (16 females) **CAMEROON**: 1 female, OSUC 211215 (CNCI). **KENYA**: 1 female, OSUC 234699 (CNCI). **NIGERIA**: 1 female, OSUC 213266 (CNCI). **SOUTH AFRICA**: 10 females, OSUC 211213, 211267, 211386, 212251, 212364, 212440, 213117 (CNCI); OSUC 244034 (FMNH); OSUC 212365 (OSUC); OSUC 213439 (SANC). **TANZANIA**: 2 females, OSUC 250963 (CNCI); OSUC 212970 (OSUC). **UGANDA**: 1 female, OSUC 244071 (USNM).

##### Comments.

As presently defined this species is quite variable. Given the available material we have elected to take a conservative approach and treat these specimens as a single species. As noted for *Scelio copelandi* this may ultimately require the inclusion of that species as well. Four morphotypes were initially identified: 1) individuals (OSUC 212970, 250963, 211215, 213266) with a bright yellow scape and very slightly larger reticulations on the mesoscutum, from Tanzania, Cameroons, Nigeria and South Africa; 2) a single individual (OSUC 211267) with fine, somewhat more dense and confused sculpture of the frons, and confused reticulate sculpture on T1; 3) four individuals (OSUC 212250, 244034, 212364, 212365) with the scape brown and metasoma broader (South Africa); and 4) a series of individuals (OSUC 211386, 213439, 213117, 211213, 212440) similar to series 3 but with the metasoma narrower. Two additional specimens similar to series 1 but with somewhat more compact sculpture of the mesoscutum are also included (OSUC 244071, 234699). Most specimens can be broadly divided into 2 groups (yellow or brown scape). However, series 2 is somewhat intermediate in this state with the middle of the scape brown, and the base and apex somewhat lighter. The shape of the head varies from somewhat vertically elongate (series 1) to more rounded (specimens of series 4). All specimens observed have brown pilosity on the medial head and mesonotum.

#### Scelio ardelio

Yoder sp. n.

http://zoobank.org/1F7E0425-9B92-4434-8C21-ABC1F8ED4465

urn:lsid:biosci.ohio-state.edu:osuc_concepts:244960

http://species-id.net/wiki/Scelio_ardelio

**Figures 43–48.**
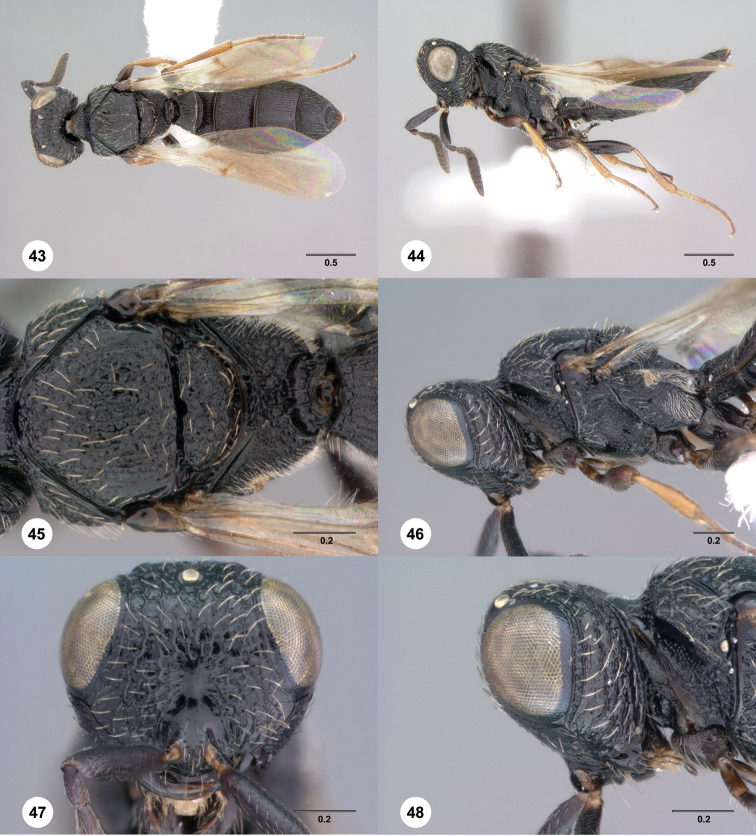
^121^ *Scelio ardelio* sp. n., holotype female (OSUC 212510). **43** Habitus, dorsal view **44** Habitus, lateral view **45** Head and mesosoma, dorsal view **46** Head and mesosoma, lateral view **47** Head, anterior view **48** Head, lateral view. Scale bars in millimeters.

Figures 43–48 ; Morphbank ^24^

##### Description.

Female body length: 3.45 mm (n=1). Shape of compound eye: not or only slightly bulging. Color pattern of pilosity below anterior ocellus in female: brown throughout. Sculpture of frons in female: reticulate rugulose, rugae finer, with slight dorsoventral trend. Genal carina in female: absent. Width of gena in lateral view: weakly expanded, posterior margin parallel to posterior margin of eye. Density of genal setae: sparse, setae generally inconspicuous. Color of genal setae: white to off-white. Sculpture of base of mandible in female: smooth. Color of A1 in female: light to dark brown throughout, or with apex and base slightly lighter, often yellowish. Color of A3 in female: brown. Sculpture of dorsal pronotal nucha in female: predominantly to completely sculptured. Color of pilosity of pronotal shoulder in female: predominantly light brown to brown. Sculpture along humeral margin of mesoscutum: with effaced areas. Color of pilosity of mesonotum in female: predominantly light to dark brown. Transition from lateral to posterior margin of propodeum in dorsal view: smoothly curved, corner of propodeum undefined. Shape of mesoscutum in lateral view: more or less flat. Pilosity on metapleuron above hind coxa: with large patch of dense fine setae. Form of fore wing submarginal vein in female: tubular throughout from base to costal margin. Fine pilosity of lateral T1 in female: present. Width of metasoma: very wide, width of S3 > 2 times medial length. Distribution of pilosity on T2–T5 in female: more or less evenly distributed throughout. Sculpture of T3 in female: longitudinally striate laterally with prominent reticulate to rugulose elements medially. Overall sculpture of S3: coarsely reticulate. Sculpture of medial S3 in female: present throughout.

##### Diagnosis.

Most similar to *Scelio chapmani*, with which it shares the dense patch of pilosity above the hind coxa on the posterolateral corner of the metapleuron (Fig. 46). This species may be distinguished from *Scelio chapmani* by the sparse brown pilosity of the gena (dense and white in *Scelio chapmani*), the the more or less rounded posterior propodeal margin (distinctly truncate with sharp lateral corner in *Scelio chapmani*), and the thin strip of obliterated sculpture along the humeral margin of the mesoscutum (sculptured throughout in *Scelio chapmani*, compare Figs 45 and 57).

##### Etymology.

The epithet is Latin, for meddler or busybody, and is used as a noun in apposition. This refers to the odd character combinations that meddle with an otherwise straightforwardly defined species group, and rhymes nicely.

##### Link to distribution map.

http://hol.osu.edu/map-large.html?id=244960

##### Material examined.

*Holotype*, female: **KENYA**: Eastern Prov., Gatab, Mount Kulal, 7000ft, IV-1980, D. Levin, OSUC 212510 (deposited in CNCI).

##### Comments.

*Scelio ardelio* has a strange mix of characters that contradicts the definition of all the major species groups. The species is tentatively included in the *ernstii*-group based in part on the patch of metapleural pilosity it shares with *Scelio chapmani*, similarities in the shape of the anteclypeus, and the general habitus. At first impression the state of the posterior propodeal margin appears closer to that observed in species of the *walkeri* and *pulchripennis* groups with a margin that appears more or less rounded (Fig. 45). A very narrow perpendicular carina is visible medially adjacent the nuchal area, however, which may indicate that *Scelio ardelio* may simply be highly derived with respect to other *ernstii*-group species. *Scelio ardelio* is also notable for several states not, or rarely observed in Afrotropical *ernstii*-group  species: the obliterated narrow strip of sculpture along the humeral margin of the mesoscutum, the absence of reticulate microsculpture at the base of the mandible, and the long and narrow setae in general. The fine pilosity of lateral T1 is particularly dense and the sculpture of T3 very reticulate.

#### Scelio bayanga

Yoder sp. n.

http://zoobank.org/F733A7E7-469D-4C1C-A6A6-6902777BA8C9

urn:lsid:biosci.ohio-state.edu:osuc_concepts:244948

http://species-id.net/wiki/Scelio_bayanga

**Figures 49–54.**
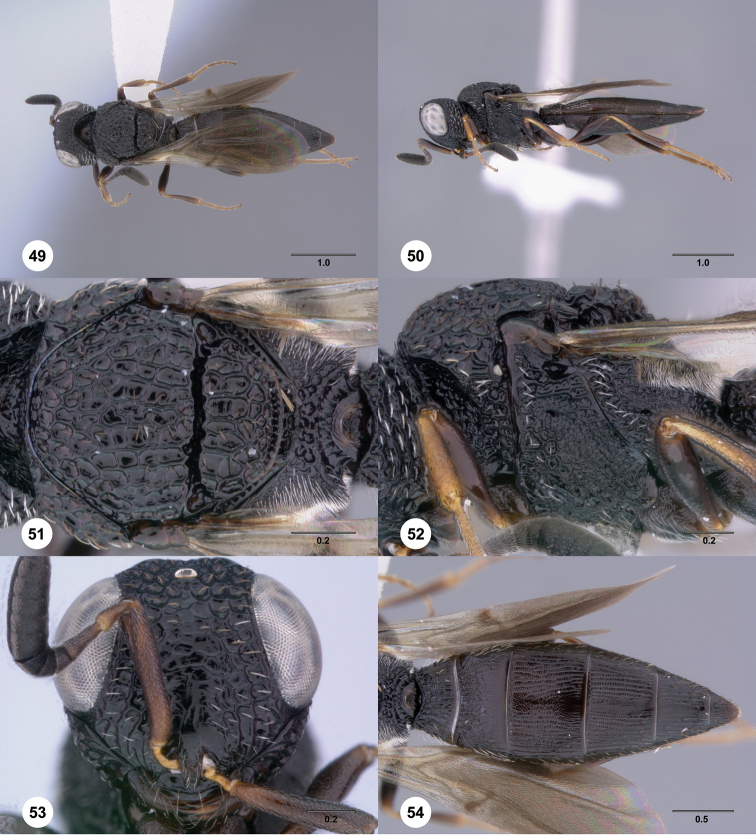
^122^ *Scelio bayanga* sp. n., holotype female (OSUC 211845). **49** Habitus, dorsal view **50** Habitus, lateral view **51** Mesosoma, dorsal view **52** Mesosoma, lateral view **53** Head, anterior view **54** Metasoma, dorsal view. Scale bars in millimeters.

Figures 49–54 ; Morphbank ^25^

##### Description.

Female body length: 4.79 mm (n=1). Shape of compound eye: not or only slightly bulging. Color pattern of pilosity below anterior ocellus in female: predominantly white throughout. Sculpture of frons in female: reticulate rugose, rugae somewhat thickened, without dorsoventral trend. Genal carina in female: absent. Width of gena in lateral view: weakly expanded, posterior margin parallel to posterior margin of eye. Density of genal setae: moderately to highly dense, setae conspicuous. Color of genal setae: white to off-white. Sculpture of base of mandible in female: minutely reticulate. Color of A1 in female: light to dark brown throughout, or with apex and base slightly lighter, often yellowish. Color of A3 in female: brown. Sculpture of dorsal pronotal nucha in female: predominantly to completely sculptured. Color of pilosity of pronotal shoulder in female: predominantly light brown to brown. Sculpture along humeral margin of mesoscutum: well-defined throughout. Color of pilosity of mesonotum in female: predominantly light to dark brown. Transition from lateral to posterior margin of propodeum in dorsal view: forming distinct angle, corner of propodeum well defined. Shape of mesoscutum in lateral view: bulging in anterior third. Pilosity on metapleuron above hind coxa: glabrous or with few scattered setae. Form of fore wing submarginal vein in female: tubular throughout from base to costal margin. Fine pilosity of lateral T1 in female: present. Width of metasoma: very wide, width of S3 > 2 times medial length. Distribution of pilosity on T2–T5 in female: more or less evenly distributed throughout. Sculpture of T3 in female: longitudinally striate laterally with prominent reticulate to rugulose elements medially. Overall sculpture of S3: with sparse, fine longitudinal carinae. Sculpture of medial S3 in female: with broadly obliterated or with distinct smooth patch.

##### Diagnosis.

Most similar to species that share the absence of the genal carina (*Scelio copelandi*, *Scelio aphares*, *Scelio phaeoprora*, *Scelio exophthalmus* and *Scelio mauritanicus*). *Scelio bayanga* differs from all these by the combination of size (larger), width of gena (very broad), and sculpture of the mesoscutum (robustly reticulate, with large cells).

##### Etymology.

A reference to the locality on the label of the holotype. The epithet is used as a noun in apposition.

##### Link to distribution map.

http://hol.osu.edu/map-large.html?id=244948

##### Material examined.

*Holotype*, female: **CENTRAL AFRICAN REPUBLIC**: Sangha-Mbaéré Préf. Écon., 21.4km (53°) NE Bayanga, Mabéa Bai, lowland rainforest / marsh clearing, CAR01-M03, 510m, 03°02.01'N, 16°24.57'E, Dzanga-Ndoki National Park, 1.V-2.V.2001, malaise trap, S. van Noort, OSUC 211845 (deposited in SAMC).

##### Comments.

The single known specimen is more similar in Gestalt to larger individuals of *Scelio taylori* which have the genal carina present.

#### Scelio chapmani

Nixon

http://zoobank.org/2C2253E5-FE53-4453-86D8-ADE3E109614F

urn:lsid:biosci.ohio-state.edu:osuc_concepts:5194

http://species-id.net/wiki/Scelio_chapmani

**Figures 55–60.**
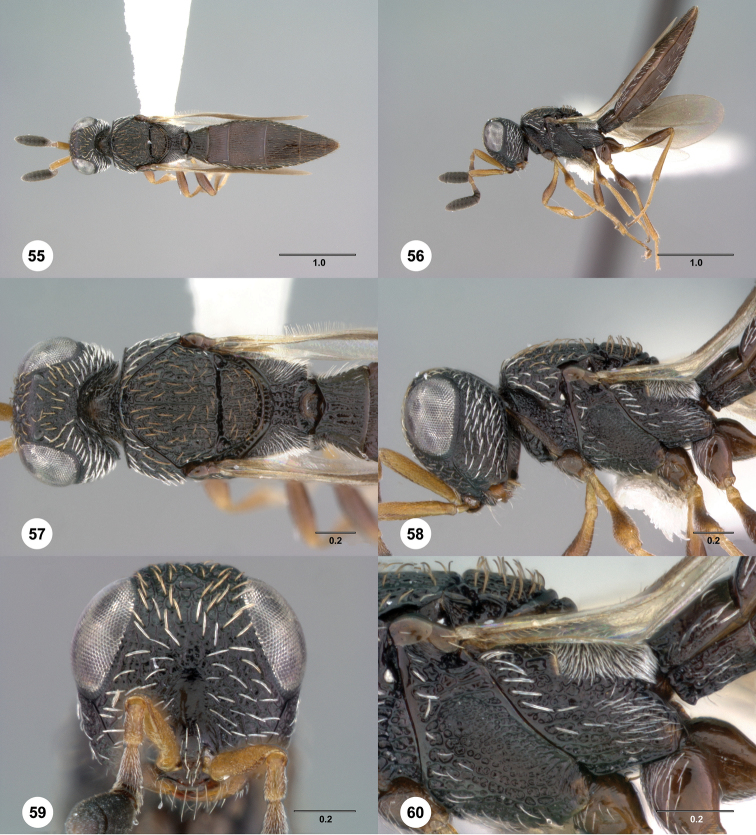
^123^ *Scelio chapmani* Nixon, female (OSUC 212119). **55** Habitus, dorsal view **56** Habitus, lateral view **57** Head and mesosoma, dorsal view **58** Head and mesosoma, lateral view **59** Head, anterior view **60** Propodeum, lateral view. Scale bars in millimeters.

Figures 55–60 ; Morphbank ^26^

Scelio chapmanni Nixon, 1958: 311 (original description, keyed); Masner 1965: 92 (type information).

##### Description.

Female body length: 3.16–4.10 mm (n=20). Male body length: 3.24–3.96 mm (n=13). Shape of compound eye: not or only slightly bulging. Color pattern of pilosity below anterior ocellus in female: predominantly white throughout. Sculpture of frons in female: reticulate rugulose, sculpture finer, typically without dorsoventral trend. Genal carina in female: absent. Width of gena in lateral view: strongly bulging, posterior margin diverging ventrally from posterior margin of eye. Density of genal setae: moderately to highly dense, setae conspicuous. Color of genal setae: white to off-white. Sculpture of base of mandible in female: minutely reticulate. Color of A1 in female: yellow throughout. Color of A3 in female: yellow; brown; yellow basally, darkening to brown near apex. Sculpture of dorsal pronotal nucha in female: predominantly to completely sculptured. Color of pilosity of pronotal shoulder in female: predominantly white to off-white. Sculpture along humeral margin of mesoscutum: well-defined throughout. Color of pilosity of mesonotum in female: predominantly light to dark brown. Transition from lateral to posterior margin of propodeum in dorsal view: forming distinct angle, corner of propodeum well defined. Shape of mesoscutum in lateral view: more or less flat. Pilosity on metapleuron above hind coxa: with large patch of dense fine setae. Form of fore wing submarginal vein in female: tubular throughout from base to costal margin. Fine pilosity of lateral T1 in female: present. Width of metasoma: moderately wide, width of S3 1.5–1.6 times medial length. Distribution of pilosity on T2–T5 in female: more or less evenly distributed throughout. Sculpture of T3 in female: longitudinally striate throughout. Overall sculpture of S3: with dense, fine longitudinal carinae. Sculpture of medial S3 in female: present throughout; with broadly obliterated or with distinct smooth patch.

##### Diagnosis.

Males and females are easily distinguished from all Afrotropical *ernstii*-group species except *Scelio ardelio* by the presence of a large setose patch (Fig. 58) above the hind coxa. This species may be separated from *Scelio ardelio* by the dense white pilosity of the gena (fine, sparse and brown in *Scelio ardelio*) and the well-developed posterior propodeal margin (more or less rounded in *Scelio ardelio*).

##### Link to distribution map.

http://hol.osu.edu/map-large.html?id=5194

##### Material examined.

*Holotype*, female: **TANZANIA**: Rukwa Reg., Rukwa Rift, Kafukola, 27.XI.1955, R. F. Chapman, B.M. TYPE HYM. 9.540 (deposited in BMNH). *Other material*: (21 females, 13 males) **CAMEROON**: 1 female, OSUC 212119 (CNCI). **CENTRAL AFRICAN REPUBLIC**: 5 females, 1 male, OSUC 213977 (OSUC); OSUC 211667, 213317, 213916, 214199, 214218 (SAMC). **IVORY COAST**: 2 females, OSUC 213222 (CNCI); OSUC 142576 (OSUC). **KENYA**: 1 female, OSUC 212354 (CNCI). NIGERIA: 8 females, 7 males, OSUC 211380, 212166, 212182, 212690, 212695, 212715, 212812, 212843, 213144, 213258, 213265, 213273, 250781, 250945 (CNCI); OSUC 59134 (OSUC). **SOUTH AFRICA**: 1 female, 2 males, OSUC 213337, 213357, 213477 (SANC). YEMEN: 1 male, OSUC 251064 (CNCI). **ZIMBABWE**: 3 females, 2 males, OSUC 212219, 212576, 213039 (CNCI); OSUC 57105, 57109 (OSUC).

##### Comments.

The identity of this species was determined by examination of the type by AP. This is perhaps the most easily diagnosed species within the group due to the characteristic distribution of metapleural setae. It is interesting to note that a similar state can be observed in species from other species groups, including *Scelio philippinensis*. The pilosity of the frons immediately below the anterior ocellus is characteristically oriented dorsoventrally. The color of the brown pilosity of the dorsal head is somewhat more golden/copper than the brown observed in other species. See also comments for *Scelio ardelio*.

In the original description of this species Nixon (1956) states in two places that the material was collected by R.F. Chapman. Therefore, the proper spelling of the genitive is *chapmani*, and not *chapmanni*. According to article 32.5.1 of the International Code of Zoological Nomenclature “If there is in the original publication itself, without recourse to any external source of information, clear evidence of an inadvertent error, such as a lapsus calami or a copyist’s or printer’s error, it must be corrected.” The change is a justified emendation and retains the original authorship and date.

#### Scelio copelandi

Yoder sp. n.

http://zoobank.org/9C6F7162-34A4-4403-BFD6-20E160151FBB

urn:lsid:biosci.ohio-state.edu:osuc_concepts:244950

http://species-id.net/wiki/Scelio_copelandi

**Figures 61–66.**
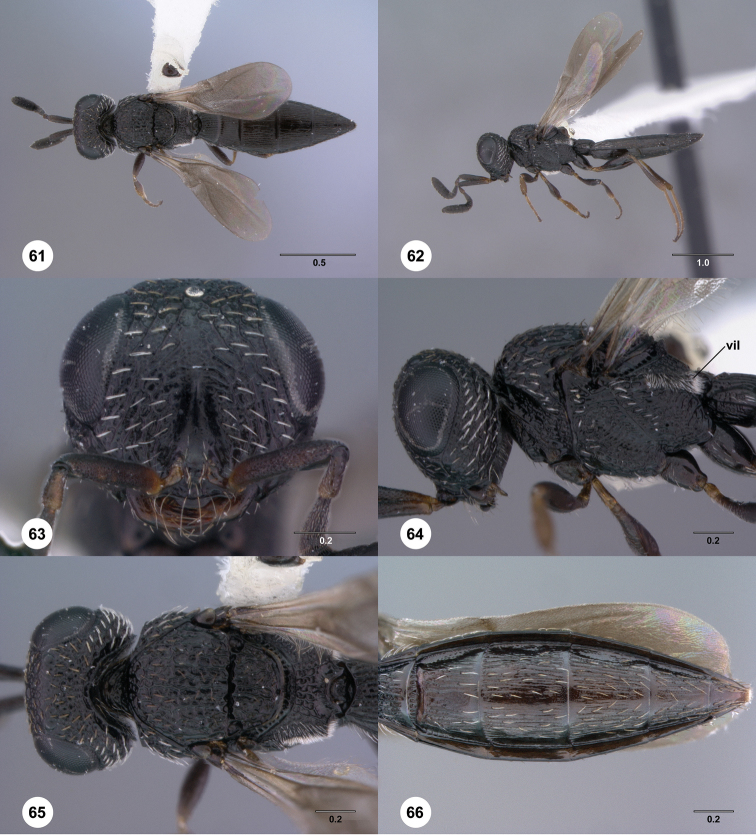
^124^ *Scelio copelandi* sp. n. **61–65** paratype female (OSUC 214063), 66, paratype female (OSUC 234640). **61** Habitus, dorsal view **62** Habitus, lateral view **63** Head and mesosoma, dorsal view **64** Head and mesosoma, lateral view **65** Head, anterior view **66** Metasoma, ventral view. *vil*, villus. Scale bars in millimeters.

Figures 61–66 ; Morphbank ^27^

##### Description.

Female body length: 3.38–3.86 mm (n=21). Male body length: 3.28–3.89 mm (n=12). Shape of compound eye: not or only slightly bulging. Color pattern of pilosity below anterior ocellus in female: predominantly white throughout. Sculpture of frons in female: reticulate rugulose, rugae finer, with slight dorsoventral trend. Genal carina in female: absent. Width of gena in lateral view: weakly expanded, posterior margin parallel to posterior margin of eye. Density of genal setae: moderately to highly dense, setae conspicuous. Color of genal setae: white to off-white. Sculpture of base of mandible in female: minutely reticulate. Color of A1 in female: light to dark brown throughout, or with apex and base slightly lighter, often yellowish. Color of A3 in female: brown. Sculpture of dorsal pronotal nucha in female: predominantly to completely sculptured. Color of pilosity of pronotal shoulder in female: predominantly white to off-white. Sculpture along humeral margin of mesoscutum: well-defined throughout. Color of pilosity of mesonotum in female: predominantly light to dark brown. Transition from lateral to posterior margin of propodeum in dorsal view: forming distinct angle, corner of propodeum well defined. Shape of mesoscutum in lateral view: bulging in anterior third. Pilosity on metapleuron above hind coxa: glabrous or with few scattered setae. Form of fore wing submarginal vein in female: tubular throughout from base to costal margin. Fine pilosity of lateral T1 in female: absent. Width of metasoma: very wide, width of S3 > 2 times medial length. Distribution of pilosity on T2–T5 in female: more or less evenly distributed throughout. Sculpture of T3 in female: longitudinally striate throughout. Overall sculpture of S3: with dense, fine longitudinal carinae. Sculpture of medial S3 in female: with broadly obliterated or with distinct smooth patch.

##### Diagnosis.

Most similar to *Scelio aphares*, which shares the absence of genal carina, the absence of a bulging eye, the bushy pilosity of the gena (for presence see Fig. 78), and the absence of brown setae on the frons near the ventral portion of the eye (for presence see Fig. 95). Differing by sculpture of the mesoscutum, with a longitudinal trend and slightly larger cells (Fig. 63), and sculpture of the face finer, with a slight dorsoventral trend (Fig. 65, compare with reticulate pattern in *Scelio aphares*, Fig. 35).

##### Etymology.

Named in reference to the collector, R. Copeland.

##### Link to distribution map.

http://hol.osu.edu/map-large.html?id=244950

##### Material examined.

*Holotype*, female: **KENYA**: Rift Valley Prov., Ol Pejeta Conservancy, 01°01.306'N, 36°54.818'E, 1779m, 12.III–26.III.2006, malaise trap, R. Copeland, OSUC 234620 (deposited in NMKE). *Paratypes*: **KENYA**: 35 females, 12 males, OSUC 212847, 214017, 214023, 214025, 214027, 214029, 214031–214032, 214036–214037, 214041, 214045, 214062–214063, 214077–214078, 234605, 234608–234611, 234613, 234615, 234619, 234621, 234625–234626, 234629, 234640, 234683–234684, 234687, 234689, 250905–250906, 250909–250910, 250912, 250915, 250928, 250936, 250944 (CNCI); OSUC 59027, 59087, 59098, 59104 (OSUC); OSUC 244085 (USNM).

##### Comments.

*Scelio copelandi* is very similar to the polymorphic *Scelio aphares*, and the two may ultimately need to be synonymized. While the differences are very subtle, the consistently fine and somewhat dorsoventrally oriented sculpture of the frons is noticeably different from the more reticulate trending sculpture in *Scelio aphares* (Fig. 35). A slight pattern of obliteration posteromedially on T2, T3 and T4 is present in most individuals, and similar smooth patches are found laterally as well. The villus is somewhat characteristic (Fig. 64, *vil*), being narrower and slightly curved ventrally.

#### Scelio dupondi

Yoder sp. n.

http://zoobank.org/F9362688-41AC-4608-92C7-13051873CFAF

urn:lsid:biosci.ohio-state.edu:osuc_concepts:244958

http://species-id.net/wiki/Scelio_dupondi

**Figures 67–72.**
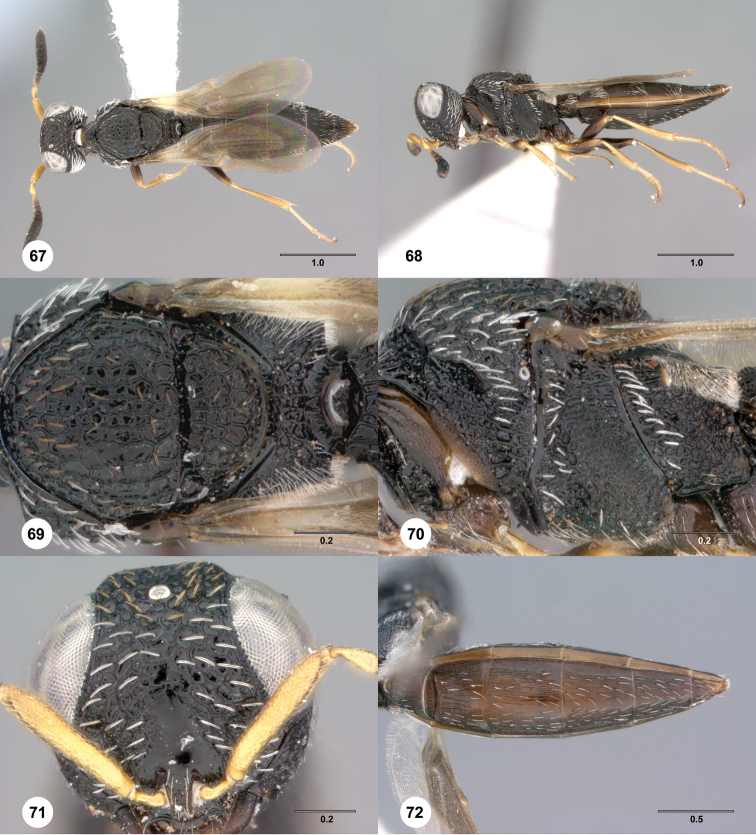
^125^ *Scelio dupondi* sp. n., paratype female (OSUC 213048). **67** Habitus, dorsal view **68** Habitus, lateral view **69** Mesosoma, dorsal view **70** Mesosoma, lateral view **71** Head, anterior view **72** Metasoma, ventral view. Scale bars in millimeters.

Figures 67–72 ; Morphbank ^28^

##### Description.

Female body length: 3.52–4.28 mm (n=24). Male body length: 3.36–3.52 mm (n=2). Shape of compound eye: not or only slightly bulging. Color pattern of pilosity below anterior ocellus in female: predominantly white throughout. Sculpture of frons in female: reticulate rugulose, sculpture finer, typically without dorsoventral trend. Genal carina in female: present. Width of gena in lateral view: weakly expanded, posterior margin parallel to posterior margin of eye. Density of genal setae: moderately to highly dense, setae conspicuous. Color of genal setae: white to off-white. Sculpture of base of mandible in female: minutely reticulate. Color of A1 in female: yellow throughout. Color of A3 in female: yellow basally, darkening to brown near apex. Sculpture of dorsal pronotal nucha in female: predominantly to completely sculptured. Color of pilosity of pronotal shoulder in female: predominantly white to off-white. Sculpture along humeral margin of mesoscutum: well-defined throughout. Color of pilosity of mesonotum in female: predominantly light to dark brown. Transition from lateral to posterior margin of propodeum in dorsal view: forming distinct angle, corner of propodeum well defined. Shape of mesoscutum in lateral view: bulging in anterior third. Pilosity on metapleuron above hind coxa: glabrous or with few scattered setae. Form of fore wing submarginal vein in female: nebulous at or just before upcurve to marginal vein, not reaching margin as a tubular vein; tubular throughout from base to costal margin. Fine pilosity of lateral T1 in female: absent. Width of metasoma: moderately wide, width of S3 1.5–1.6 times medial length. Distribution of pilosity on T2–T5 in female: individual tergites glabrous anteriorly, densely setose posteriorly. Sculpture of T3 in female: longitudinally striate laterally with prominent reticulate to rugulose elements medially. Overall sculpture of S3: with dense, fine longitudinal carinae. Sculpture of medial S3 in female: present throughout.

##### Diagnosis.

Differs from all other Afrotropical *ernstii*-group species except *Scelio janseni* by the setal pattern of the lateral tergites (Figs 68, 80). Differing from *Scelio janseni* in size (smaller), sculpture of the frons (finer), width of metasoma (much narrower), type and density of sculpture on the metasoma (finer, more compact, longitudinal, with S3 sculptured more or less throughout), and development of the S3 felt field (very reduced and not or barely elevated).

##### Etymology.

The epithet is used as a genitive noun derived from the French name for the one of the Thompson twin detective characters in Tintin comics. The two were always being confused with one another. See also *Scelio janseni*.

##### Link to distribution map.

http://hol.osu.edu/map-large.html?id=244958

##### Material examined.

*Holotype*, female: **SOUTH AFRICA**: Limpopo Prov., 15km E Klaserie, Guernsey Farm, 19.XII–31.XII.1985, malaise trap, H. Howden & A. Howden, OSUC 212309 (deposited in CNCI). *Paratypes*: (24 females, 2 males) **SOUTH AFRICA**: 6 females, OSUC 211204-211205, 211256, 211263 (CNCI); OSUC 211260 (OSUC); OSUC 213369 (SANC). **ZIMBABWE**: 18 females, 2 males, OSUC 211241, 211247, 212110, 212142, 212151, 212405, 212567, 212647, 212841, 213008, 213051, 213053-213054, 213205, 213208, 213210, 213228, 213249 (CNCI); OSUC 211225, 213048 (OSUC).

##### Comments.

This species is very similar to the larger, more robust *Scelio janseni*. However, all available specimens are relatively easily separated, with no indication of morphological gradation among the two. The sculpture of the frons in general tends to be finer than that of *Scelio janseni*. The area of reticulate sculpture at the base of the mandible is very reduced and difficult to see, but it is present. T3 medially is strongly reticulate in most individuals. Two males have been associated: A5 is more or less cylindrical, and the following flagellomeres are subequal in size. While small in number, the males of *Scelio dupondi* and *Scelio janseni* may be divided by the color of the fore wing (completely hyaline in *Scelio dupondi* and infuscate brown throughout in *Scelio janseni*) and the form of the flagellum (more compact and wider in *Scelio janseni*). In males the A5 RSS is a fine, linear carina, and A5 is not distinctly broader at its apex. This contrasts with *Scelio janseni* in which A5 is shaped more like a chalice with the apex much wider than base.

#### Scelio exophthalmus

Yoder sp. n.

http://zoobank.org/D3685D2B-DE60-4D04-B6C7-43083CEBA289

urn:lsid:biosci.ohio-state.edu:osuc_concepts:244955

http://species-id.net/wiki/Scelio_exophthalmus

**Figures 73–78.**
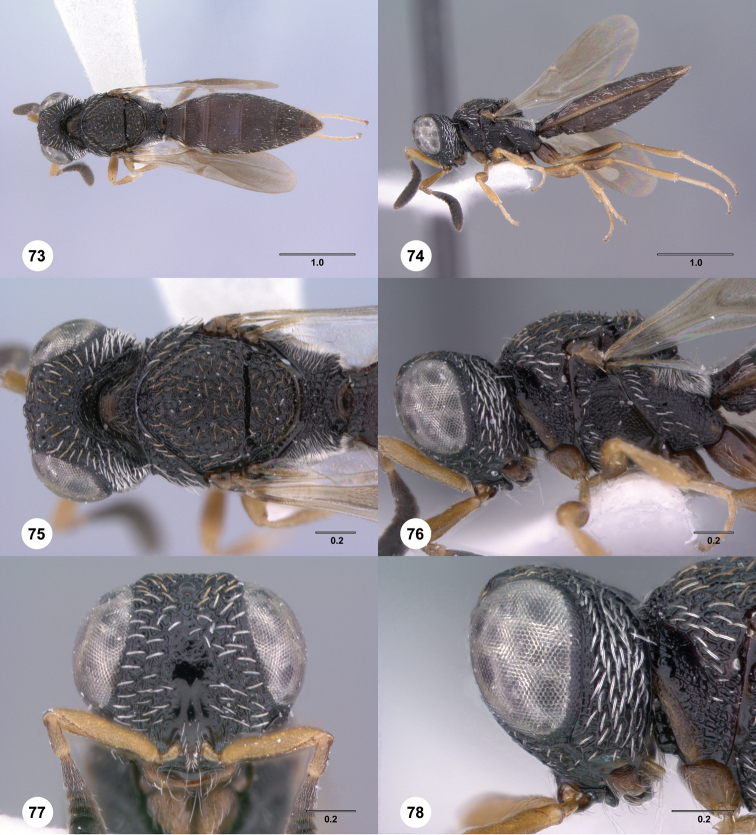
^126^ *Scelio exophthalmus* sp. n., holotype female (OSUC 212123). **73** Habitus, dorsal view **74** Habitus, lateral view **75** Head and mesosoma, dorsal view **76** Head and mesosoma, lateral view **77** Head, anterior view **78** Head, lateral view. Scale bars in millimeters.

Figures 73–78 ; Morphbank ^29^

##### Description.

Female body length: 3.32–3.91 mm (n=9). Shape of compound eye: conspicuously bulging. Color pattern of pilosity below anterior ocellus in female: predominantly white throughout. Sculpture of frons in female: reticulate rugulose, sculpture finer, typically without dorsoventral trend. Genal carina in female: absent. Width of gena in lateral view: strongly bulging, posterior margin diverging ventrally from posterior margin of eye. Density of genal setae: moderately to highly dense, setae conspicuous. Color of genal setae: white to off-white. Sculpture of base of mandible in female: minutely reticulate. Color of A1 in female: yellow throughout. Color of A3 in female: brown. Sculpture of dorsal pronotal nucha in female: predominantly to completely sculptured. Color of pilosity of pronotal shoulder in female: predominantly white to off-white. Sculpture along humeral margin of mesoscutum: well-defined throughout. Color of pilosity of mesonotum in female: predominantly light to dark brown. Transition from lateral to posterior margin of propodeum in dorsal view: forming distinct angle, corner of propodeum well defined. Shape of mesoscutum in lateral view: bulging in anterior third. Pilosity on metapleuron above hind coxa: glabrous or with few scattered setae. Form of fore wing submarginal vein in female: tubular throughout from base to costal margin. Fine pilosity of lateral T1 in female: present. Width of metasoma: moderately wide, width of S3 1.5–1.6 times medial length. Distribution of pilosity on T2–T5 in female: more or less evenly distributed throughout. Sculpture of T3 in female: longitudinally striate laterally with prominent reticulate to rugulose elements medially. Overall sculpture of S3: with dense, fine longitudinal carinae. Sculpture of medial S3 in female: with broadly obliterated or with distinct smooth patch.

##### Diagnosis.

Differs from all similar species without a genal carina by the combination of the presence of very bushy pilosity on the gena, the bulging eye (Fig. 78) the reticulate sculpture of medial T3, and the sculptured pronotal nucha.

##### Etymology.

The epithet is used as a Latinized adjective for the Greek word meaning bulging or protruding eyes.

##### Link to distribution map.

http://hol.osu.edu/map-large.html?id=244955

##### Material examined.

*Holotype*, female: **CAMEROON**: Nkoemvom, VIII-1980, malaise trap, D. Jackson, OSUC 212123 (deposited in BMNH). *Paratypes*: (8 females) **CAMEROON**: 2 females, OSUC 212108, 212115 (CNCI). **IVORY COAST**: 2 females, OSUC 213234 (CNCI); OSUC 213064 (OSUC). **NIGERIA**: 4 females, OSUC 212805-212806, 213276 (CNCI); OSUC 213259 (OSUC).

##### Comments.

The submarginal carina of T2 is particularly well developed and flange-like anteriorly.

#### Scelio janseni

Yoder sp. n.

http://zoobank.org/1EC6BF0A-416D-4868-9BCA-C93FCF85FC61

urn:lsid:biosci.ohio-state.edu:osuc_concepts:244959

http://species-id.net/wiki/Scelio_janseni

**Figures 79–84.**
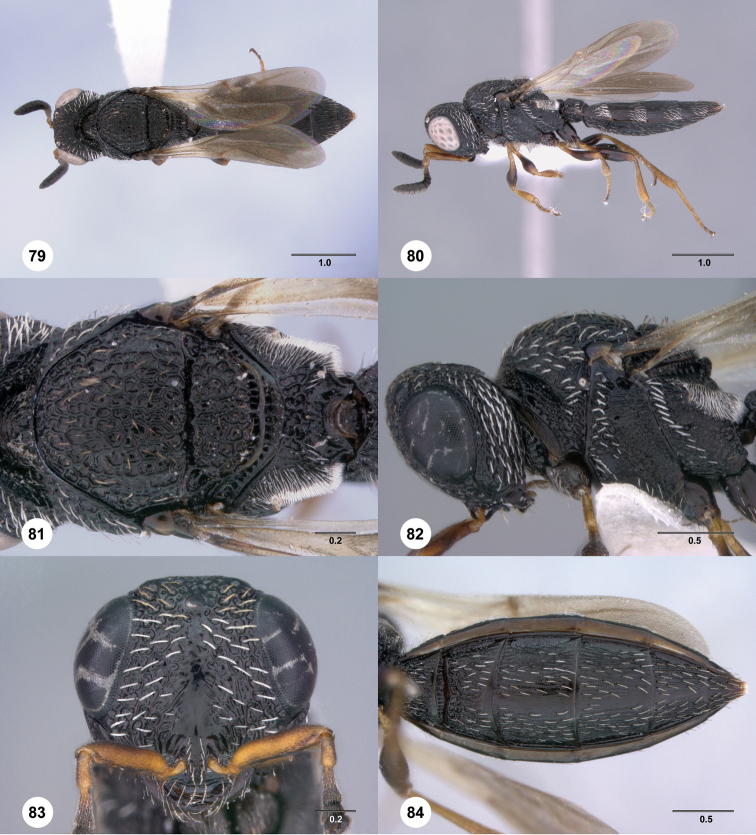
^127^ *Scelio janseni* sp. n. **79–81** holotype female (OSUC 222129), 82–84 paratype female (OSUC 212237). **79** Habitus, dorsal view **80** Habitus, lateral view **81** Mesosoma, dorsal view **82** Head and mesosoma, lateral view **83** Head, anterior view **84** Metasoma, ventral-oblique view. Scale bars in millimeters.

Figures 79–84 ; Morphbank ^30^

##### Description.

Female body length: 4.44–5.35 mm (n=12). Male body length: 4.20 mm (n=1). Shape of compound eye: not or only slightly bulging. Color pattern of pilosity below anterior ocellus in female: predominantly white throughout. Sculpture of frons in female: reticulate rugose, rugae somewhat thickened, without dorsoventral trend. Genal carina in female: absent. Width of gena in lateral view: weakly expanded, posterior margin parallel to posterior margin of eye. Density of genal setae: moderately to highly dense, setae conspicuous. Color of genal setae: white to off-white. Sculpture of base of mandible in female: minutely reticulate. Color of A1 in female: yellow throughout; light to dark brown throughout, or with apex and base slightly lighter, often yellowish. Color of A3 in female: brown; yellow basally, darkening to brown near apex. Sculpture of dorsal pronotal nucha in female: predominantly to completely sculptured. Color of pilosity of pronotal shoulder in female: predominantly white to off-white. Sculpture along humeral margin of mesoscutum: well-defined throughout. Color of pilosity of mesonotum in female: predominantly light to dark brown. Transition from lateral to posterior margin of propodeum in dorsal view: forming distinct angle, corner of propodeum well defined. Shape of mesoscutum in lateral view: bulging in anterior third. Pilosity on metapleuron above hind coxa: glabrous or with few scattered setae. Form of fore wing submarginal vein in female: nebulous at or just before upcurve to marginal vein, not reaching margin as a tubular vein. Fine pilosity of lateral T1 in female: absent. Width of metasoma: very wide, width of S3 > 2 times medial length. Distribution of pilosity on T2–T5 in female: individual tergites glabrous anteriorly, densely setose posteriorly. Sculpture of T3 in female: longitudinally striate throughout; longitudinally striate laterally with prominent reticulate to rugulose elements medially. Overall sculpture of S3: with dense, fine longitudinal carinae. Sculpture of medial S3 in female: with broadly obliterated or with distinct smooth patch.

##### Diagnosis.

Differs from all other Afrotropical *ernstii*-group species except *Scelio dupondi* by the setal pattern of the lateral metasoma (Figs 68, 80). Differing from *Scelio dupondi* in its in larger size, wider metasoma, the coarser, more irregular and less compact sculpture on the metasomal sterna (often with some unsculptured patches on S3, Fig. 84), and the well-developed S3 felt field, which is elevated on a keel-like projection (Fig. 84).

##### Etymology.

The epithet is used as a genitive noun derived from the Dutch name for the one of the Thompson twin detective characters in Tintin comics. The two were always being confused with one another. See also *Scelio dupondi*.

##### Link to distribution map.

http://hol.osu.edu/map-large.html?id=244959

##### Material examined.

*Holotype*, female: **SOUTH AFRICA**: Limpopo Prov., nr. Den Staat Farm, by Limpopo River, Samaria Farm, 22°11.504'S, 29°16.812'E, 1750ft, 18.XII–31.III.2000, flight intercept trap, T. K. Philips, OSUC 222129 (deposited in OSUC). *Paratypes*: (11 females, 1 male) **CAMEROON**: 1 female, OSUC 212121 (CNCI). **KENYA**: 1 female, 1 male, OSUC 234676, 250971 (CNCI). **SOUTH AFRICA**: 9 females, OSUC 212236-212238, 212455-212456, 212784, 213080 (CNCI); OSUC 222128, 222132 (OSUC).

##### Comments.

*Scelio janseni* is very similar to its smaller, putative sister species *Scelio dupondi*. A single male is known, its flagellum has a distinctly different form than *Scelio dupondi*, with A3–A5 distinctly broader towards the apex, and A5–A6 somewhat compact and shorter. The scape is often very slightly darker dorsomedially, A3 is more commonly brown more or less throughout, less commonly with yellow present in basal 1/3 to 1/2. The RSS is a fine linear carina that is slightly elevated apically. Sculpture of T3 varies from more or less longitudinal to more or less reticulate, the latter state is more common in the specimens observed. See also Comments for *Scelio dupondi*. In most individuals the sculpture of posteromedial T1 to anteromedial T3 is slightly obliterated in patches. The fine pilosity of lateral T1 common in many Afrotropical *Scelio* seems to be absent, though one or two of the most anterior setae are often slightly narrower and thinner than the more prominent thick setae seen throughout.

#### Scelio mauritanicus

Risbec

http://zoobank.org/283D3C16-4E6B-45DA-8FE4-952E7F018C5B

urn:lsid:biosci.ohio-state.edu:osuc_concepts:5272

http://species-id.net/wiki/Scelio_mauritanicus

**Figures 85–90.**
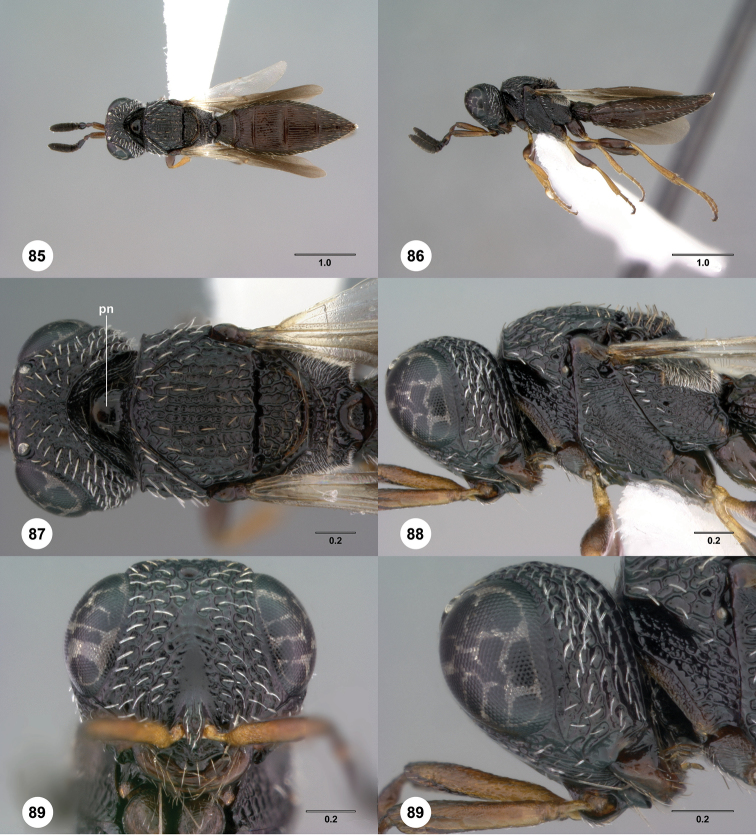
^128^ *Scelio mauritanicus* Risbec, female (OSUC 213146). **85** Habitus, dorsal view **86** Habitus, lateral view **87** Head and mesosoma, dorsal view **88** Head and mesosoma, lateral view **89** Head, anterior view **90** Head, lateral view. *pn*, pronotal nucha. Scale bars in millimeters.

Figures 85–90 ; Morphbank ^31^

Scelio mauritanicus Risbec, 1950: 587 (original description); Risbec 1954: 1038 (description, variation, suggested synonymy); Risbec 1957: 147 (variation); Nixon 1958: 311, 317 (keyed); Masner 1976: 18 (type information).
Scelio cahirensis Priesner, 1951: 144. (original description); Kononova and Kozlov 2008: 140, 156 (description, keyed). syn. n.
Scelio mauritanicus http://zoobank.org/458735E6-4A5B-447F-B719-386BB451163D
Scelio mauritanicus urn:lsid:biosci.ohio-state.edu:osuc_concepts:5186

##### Description.

Female body length: 3.12–4.30 mm (n=24). Male body length: 2.57–4.50 mm (n=37). Shape of compound eye: not or only slightly bulging. Color pattern of pilosity below anterior ocellus in female: predominantly white throughout. Sculpture of frons in female: reticulate rugulose, sculpture finer, typically without dorsoventral trend. Genal carina in female: absent. Width of gena in lateral view: weakly expanded, posterior margin parallel to posterior margin of eye. Density of genal setae: moderately to highly dense, setae conspicuous. Color of genal setae: white to off-white. Sculpture of base of mandible in female: smooth. Color of A1 in female: yellow to light brown at base, darkening near mid point to light to dark brown. Color of A3 in female: brown. Sculpture of dorsal pronotal nucha in female: predominantly to completely smooth. Color of pilosity of pronotal shoulder in female: predominantly white to off-white. Sculpture along humeral margin of mesoscutum: well-defined throughout. Color of pilosity of mesonotum in female: predominantly light to dark brown. Transition from lateral to posterior margin of propodeum in dorsal view: forming distinct angle, corner of propodeum well defined. Shape of mesoscutum in lateral view: more or less flat. Pilosity on metapleuron above hind coxa: glabrous or with few scattered setae. Form of fore wing submarginal vein in female: tubular throughout from base to costal margin. Fine pilosity of lateral T1 in female: present. Width of metasoma: very wide, width of S3 > 2 times medial length. Distribution of pilosity on T2–T5 in female: more or less evenly distributed throughout. Sculpture of T3 in female: longitudinally striate laterally with prominent reticulate to rugulose elements medially. Overall sculpture of S3: with dense, fine longitudinal carinae. Sculpture of medial S3 in female: present throughout.

##### Diagnosis.

Similar to other Afrotropical *ernstii*-group species with the genal carina absent. Distinguishable by the completely smooth pronotal nucha (Fig. 87, *pn*). The combination of the very flat mesoscutum, color transition from yellow to brown on the scape, and rugulose sculpture of medial T3 may be further used to aid in the diagnosis of many individuals.

##### Link to distribution map.

http://hol.osu.edu/map-large.html?id=5272

##### Material examined.

*Lectotype*, *Scelio mauritanicus*, female (**present designation**): **MAURITANIA**: Trarza Rég., millet stalk, Rkîz, 24.VI.1946, [RKIZ, ‘tige de mil’, 24.6.1946, boîte 10], OSUC 250704 (deposited in MNHN). *Holotype*, *Scelio cahirensis*: **EGYPT**: Pyramids, 15.10.33, Dr. H. Priesner, type No. 70877 (deposited in USNM). *Paralectotype*, *Scelio mauritanicus*: **SENEGAL**: 1 male, OSUC 254716 (MNHN). *Paratypes*, *Scelio cahirensis*: **EGYPT**: 2 females, OSUC 244152, 244153 (USNM). *Other material*: (36 females, 26 males, 9 unknowns) **BENIN**: 1 female, 1 unknown, BMNH(E)#790391 (BMNH); OSUC 211374 (CNCI). **BURKINA** **FASO**: 1 female, OSUC 213087 (CNCI). **BURUNDI**: 1 female, OSUC 182053 (RMCA). **CAMEROON**: 1 female, OSUC 212126 (CNCI). **EGYPT**: 2 females, OSUC 244152–244153 (USNM). **ERITREA**: 3 unknowns, BMNH(E)#790390, 790419–790420 (BMNH). **IVORY** **COAST**: 7 females, OSUC 212925, 213017, 213221, 213223, 213232, 213237 (CNCI); OSUC 213071 (OSUC). **KENYA**: 2 females, OSUC 234665–234666 (CNCI). **MALI**: 1 unknown, BMNH(E)#790392 (BMNH). **NIGERIA**: 9 females, 18 males, OSUC 211665–211666, 211888, 212172, 212177, 212179, 212184, 212188, 212194, 212685, 212691–212692, 212706, 212716, 212737, 212813, 213030–213031, 213033, 213143, 213146, 213154, 250970, 250995, 251013 (CNCI); OSUC 212696, 212738 (OSUC). **RWANDA**: 1 female, OSUC 182052 (RMCA). **SENEGAL**: 1 male, OSUC 254724 (MNHN). **SUDAN**: 1 female, OSUC 244077 (USNM). **TANZANIA**: 3 unknowns, BMNH(E)#790388, 790417–790418 (BMNH). **UNITED** **ARAB** **EMIRATES**: 3 females, 1 unknown, BMNH(E)#790389 (BMNH); OSUC 214076 (CNCI); OSUC 214057, 214060 (OSUC). **YEMEN**: 7 females, 7 males, OSUC 212476, 250681, 251008, 251065, 254669–254670, 254681, 254688, 254695–254696, 254775, 254784, 254786, 254788 (CNCI).

##### Comments.

The lectotype of *Scelio mauritanicus* (OSUC 250704) was identified by unique label data “Mauritanie, Rkiz., 24-6-1946”. It, and the type material of *Scelio cahirensis* match well, both being smaller individuals within the presently circumscribed size range (see below). The available material falls into a gradation between three general morphotypes. Given this variation we do not feel it prudent to subdivide *Scelio mauritanicus* into two or three separate species. All three morphotypes share several core characters, these first noted by Nixon (1958): the absence of sculpture on the pronotal nucha is constant and obvious (Fig. 87, *pn*); the mesonotum is relatively flat; the fore wing does not or just reaches T6; and sculpture medial to the lateral ocelli is usually obliterated or smoothed. Several additional characters link a majority of specimens: in females and larger males the fore wing has a golden infuscation more or less throughout; the eye is slightly quadrate in lateral view; the medial sculpture of T3 is variable, though it is usually at least slightly developed; and the base of the mandible is smooth in most individuals (contrasting with the fine reticulate sculpture seen in most Afrotropical *ernstii*-group species). The larger morphotype (Figs 85–90), predominantly from Nigeria and the Ivory Coast, has the scape with the basal 1/4 yellow, the remainder light brown (females and males). It also has a somewhat more wedge-shaped head, though this appears to vary with size. Smaller individuals, predominantly from Yemen and the UAE, have a slightly more rounded head, are lighter colored in general, and have slightly less infuscation in the wings. Large and small morphs are linked through moderately sized individuals known from Nigeria, Cameroon, and Burkina Faso. Specimens from Kenya and Sudan (OSUC 234665, 234666, and 244077) are moderately sized and differ primarily in the color of the scape and femora, which are yellow with at most only extremely slight infuscation. We anticipate that *Scelio mauritanicus* and *Scelio cahirensis* will remain in synonymy regardless of whether the species is split, both being exemplars of the smaller size range.

#### Scelio phaeoprora

Yoder sp. n.

http://zoobank.org/1B80AD17-5774-4B26-9706-6C17C6D56FF4

urn:lsid:biosci.ohio-state.edu:osuc_concepts:244953

http://species-id.net/wiki/Scelio_phaeoprora

**Figures 91–96.**
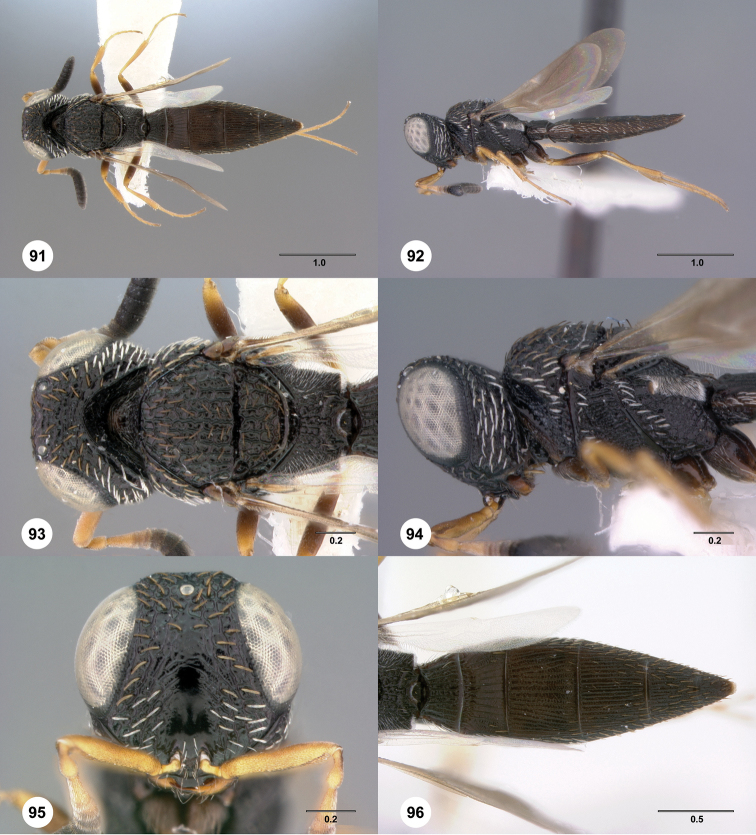
^129^ *Scelio phaeoprora* sp. n., holotype female (OSUC 213076). **91** Habitus, dorsal view **92** Habitus, lateral view **93** Head and mesosoma, dorsal view **94** Head and mesosoma, lateral view **95** Head, anterior view **96** Metasoma, dorsal view. Scale bars in millimeters.

Figures 91–96 ; Morphbank ^32^

##### Description.

Female body length: 3.80 mm (n=1). Shape of compound eye: not or only slightly bulging. Color pattern of pilosity below anterior ocellus in female: bicolored, with brown setae reaching to near ventral margin of eye. Sculpture of frons in female: reticulate rugulose, rugae finer, with slight dorsoventral trend. Genal carina in female: absent. Width of gena in lateral view: strongly bulging, posterior margin diverging ventrally from posterior margin of eye. Density of genal setae: moderately to highly dense, setae conspicuous. Color of genal setae: white to off-white. Sculpture of base of mandible in female: minutely reticulate. Color of A1 in female: yellow throughout. Color of A3 in female: yellow. Sculpture of dorsal pronotal nucha in female: predominantly to completely sculptured. Color of pilosity of pronotal shoulder in female: predominantly white to off-white. Sculpture along humeral margin of mesoscutum: well-defined throughout. Color of pilosity of mesonotum in female: predominantly light to dark brown. Transition from lateral to posterior margin of propodeum in dorsal view: forming distinct angle, corner of propodeum well defined. Shape of mesoscutum in lateral view: bulging in anterior third. Pilosity on metapleuron above hind coxa: glabrous or with few scattered setae. Form of fore wing submarginal vein in female: tubular throughout from base to costal margin. Fine pilosity of lateral T1 in female: present. Width of metasoma: moderately wide, width of S3 1.5–1.6 times medial length. Distribution of pilosity on T2–T5 in female: more or less evenly distributed throughout. Sculpture of T3 in female: longitudinally striate throughout. Overall sculpture of S3: with dense, fine longitudinal carinae. Sculpture of medial S3 in female: with broadly obliterated or with distinct smooth patch.

##### Diagnosis.

Similar to Afrotropical *ernstii*-group species without the genal carina. Differing from all these species by the unique color pattern of the setae of the frons, with brown pilosity reaching near to the base of the eye (Fig. 95).

##### Etymology.

From the Greek “phaeo” for brown and “prora”- prow/bow, in reference to the front or face of the insect; The epithet is used as a noun in apposition.

##### Link to distribution map.

http://hol.osu.edu/map-large.html?id=244953

##### Material examined.

*Holotype*, female: **IVORY COAST**: Ivory Coast, rice field, Bouaké, III-1980, P. Cochereau, OSUC 213076 (deposited in CNCI).

##### Comments.

*Scelio phaeoprora* is most similar to *Scelio exophthalmus* in general habitus and sculpture. The pilosity of the gena is slightly bushier in *Scelio phaeoprora*, but not to the degree observed in *Scelio exophthalmus*, and the eye is not distinctly bulging. All specimens of *Scelio exophthalmus* have A3 onwards light brown to brown, A3 is bright yellow in *Scelio phaeoprora*. While there are frequently a row or a few scattered brown setae below the anterior ocellus on the frons, the continuation of brown setae to the extent observed in *Scelio phaeoprora* (Fig. 95) is extremely rare for all Afrotropical *Scelio*. The metasoma of *Scelio phaeoprora* is quite narrow, and T2 appears to be slightly shorter and more elongate than in individuals of *Scelio exophthalmus*. The frons of *Scelio phaeoprora* is somewhat narrowed throughout.

#### Scelio taylori

Nixon

http://zoobank.org/4BCB74EB-232D-4748-A87D-2DBA0F96C0A7

urn:lsid:biosci.ohio-state.edu:osuc_concepts:5342

http://species-id.net/wiki/Scelio_taylori

Figures 8 , 97 – 114 ; Morphbank ^33^

Scelio taylori Nixon, 1958: 313 (original description, keyed); Masner 1965: 95 (type information).

##### Description.

Female body length: 3.16–4.73 mm (n=42). Male body length: 3.20–5.10 mm (n=37). Shape of compound eye: not or only slightly bulging. Color pattern of pilosity below anterior ocellus in female: brown throughout. Sculpture of frons in female: reticulate rugose, rugae somewhat thickened, without dorsoventral trend. Genal carina in female: present. Width of gena in lateral view: weakly expanded, posterior margin parallel to posterior margin of eye. Density of genal setae: sparse, setae generally inconspicuous. Color of genal setae: white to off-white; distinctly brown. Sculpture of base of mandible in female: minutely reticulate. Color of A1 in female: yellow throughout; light to dark brown throughout, or with apex and base slightly lighter, often yellowish. Color of A3 in female: brown. Sculpture of dorsal pronotal nucha in female: predominantly to completely sculptured. Color of pilosity of pronotal shoulder in female: predominantly white to off-white; predominantly light brown to brown. Sculpture along humeral margin of mesoscutum: well-defined throughout. Color of pilosity of mesonotum in female: predominantly light to dark brown. Transition from lateral to posterior margin of propodeum in dorsal view: forming distinct angle, corner of propodeum well defined. Shape of mesoscutum in lateral view: bulging in anterior third. Pilosity on metapleuron above hind coxa: glabrous or with few scattered setae. Form of fore wing submarginal vein in female: nebulous at or just before upcurve to marginal vein, not reaching margin as a tubular vein; tubular throughout from base to costal margin. Fine pilosity of lateral T1 in female: absent. Width of metasoma: moderately wide, width of S3 1.5–1.6 times medial length. Distribution of pilosity on T2–T5 in female: more or less evenly distributed throughout. Sculpture of T3 in female: longitudinally striate throughout. Overall sculpture of S3: with dense, fine longitudinal carinae. Sculpture of medial S3 in female: present throughout; with broadly obliterated or with distinct smooth patch.

##### Diagnosis.

Most similar to *Scelio albatus* which also has the genal carina well developed. Easily distinguished from *Scelio albatus* by the color of the pilosity (completely white in *Scelio albatus*), robustness of sculpture (finer, more compact in *Scelio albatus*) and, for most individuals, the color of the scape (dark brown in *Scelio albatus*, yellow to light brown in nearly all *Scelio taylori*).

**Figures 97–102.**
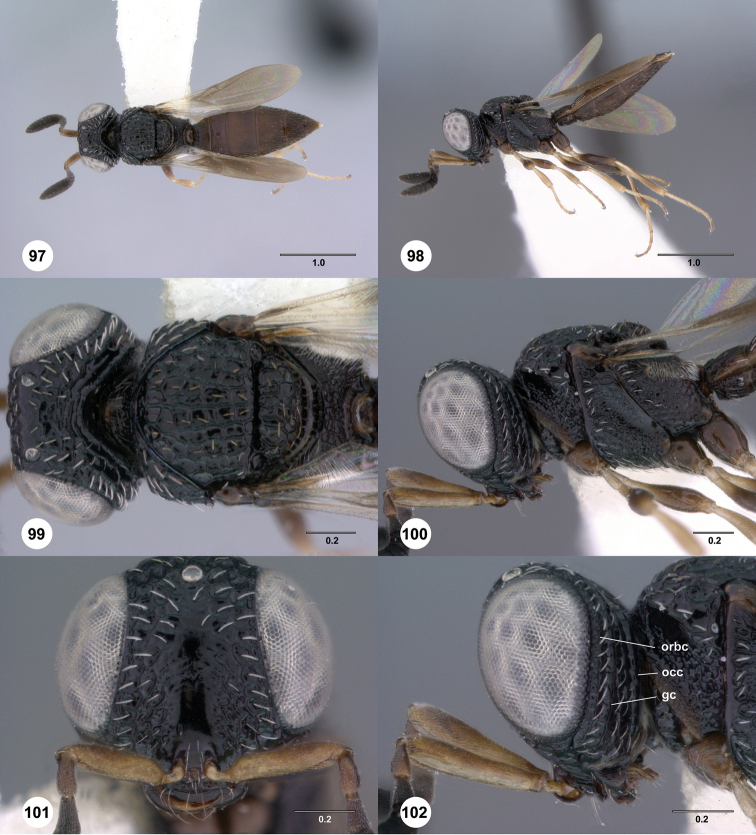
^130^ *Scelio taylori* Nixon, female (OSUC 212801). **97** Habitus, dorsal view **98** Habitus, lateral view **99** Head and mesosoma, dorsal view **100** Head and mesosoma, lateral view **101** Head, anterior view **102** Head, lateral view. Scale bars in millimeters. *gc*, genal carina; *occ*, occipital carina; *orbc*, orbital carina.

**Figures 103–108.**
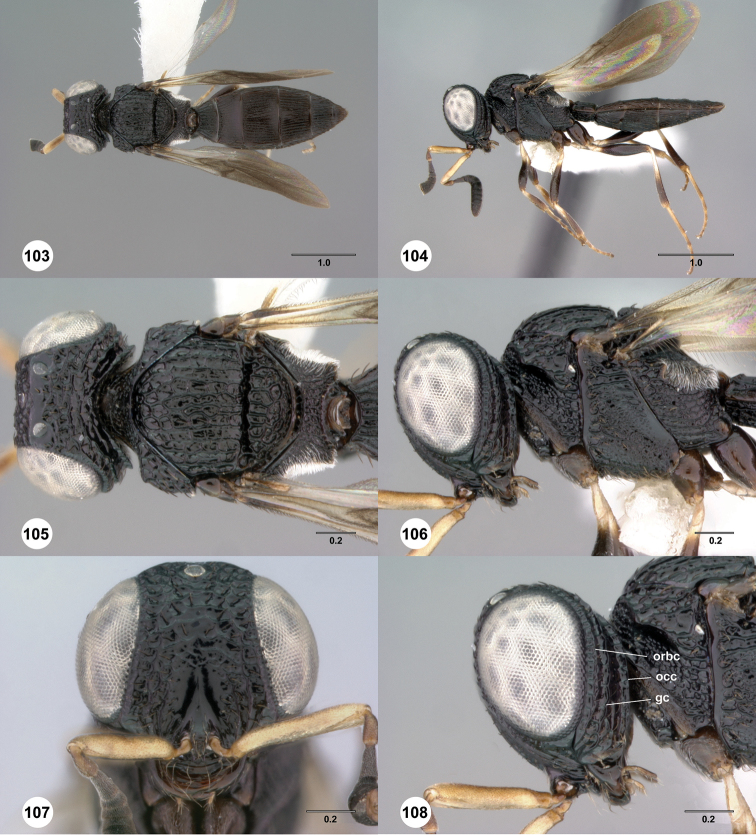
^131^ *Scelio taylori* Nixon, female (OSUC 213180). **103** Habitus, dorsal view **104** Habitus, lateral view **105** Head and mesosoma, dorsal view **106** Head and mesosoma, lateral view **107** Head, anterior view **108** Head, lateral view. *gc*, genal carina; *occ*, occipital carina; *orbc*, orbital carina. Scale bars in millimeters.

**Figures 109–114.**
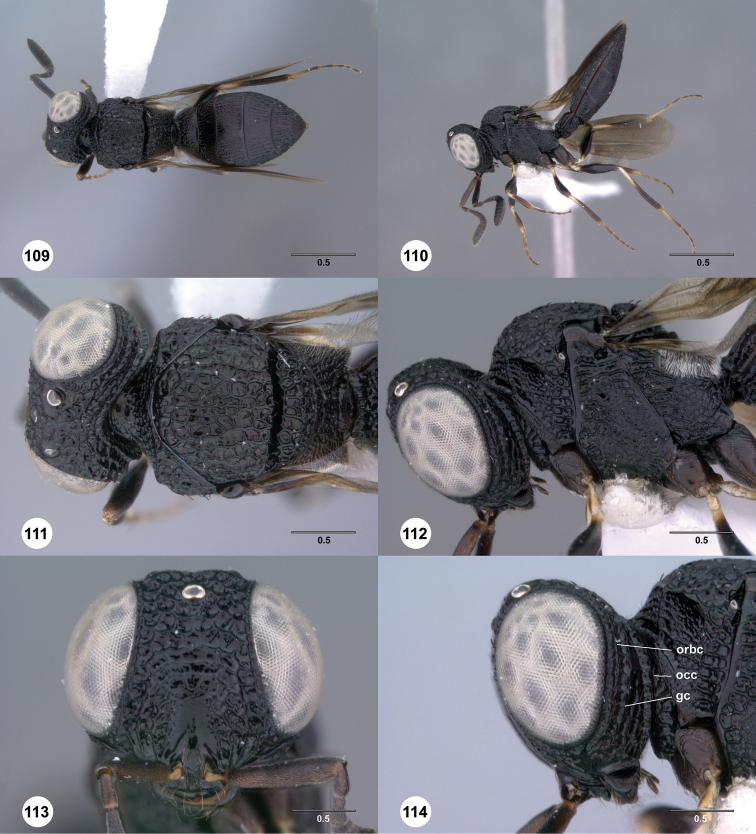
^132^ *Scelio taylori* Nixon, female (OSUC 213533). **109** Habitus, dorsal view **110** Habitus, lateral view **111** Head and mesosoma, dorsal view **112** Head and mesosoma, lateral view **113** Head, anterior view **114** Head, lateral view. *gc*, genal carina; *occ*, occipital carina; *orbc*, orbital carina. Scale bars in millimeters.

##### Link to distribution map.

http://hol.osu.edu/map-large.html?id=5342

##### Material examined.

*Holotype*, female: **UGANDA**: Toro, IV-1941, T. H. C. Taylor, B.M. TYPE HYM. 9.538 (deposited in BMNH). *Paratype*: **UGANDA**: 1 female, BMNH(E)#790423 (BMNH). *Other material*: (58 females, 39 males) **BENIN**: 3 females, 4 males, OSUC 211370, 212290, 491191 (CNCI); OSUC 142656, 142658, 142664, 142666 (OSUC). **CAMEROON**: 8 females, OSUC 211220–211221, 211399, 212114, 212125, 212259, 212261, 212898 (CNCI). **CENTRAL AFRICAN REPUBLIC**: 3 females, 5 males, OSUC 213971 (OSUC); OSUC 176092, 212400, 213533, 213925, 214187, 214197 (SAMC); OSUC 250667 (SANC). **GABON**: 1 female, OSUC 213128 (CNCI). **GHANA**: 1 female, OSUC 213563 (OSUC). **IVORY COAST**: 4 females, OSUC 211274, 213060, 213078, 213227 (CNCI). **KENYA**: 16 females, 22 males, OSUC 212506, 214083, 214088–214090, 214096, 214098–214100, 214117, 214119, 214121, 214123, 214125, 214127–214131, 214133, 214180, 234635, 234649, 234651–234652, 234654–234656, 234658–234659, 234692–234694, 234697–234698, 250821 (CNCI); OSUC 244089, 244093 (USNM). **MALAWI**: 3 females, OSUC 212744, 212799, 213138 (CNCI). **NIGERIA**: 12 females, 4 males, OSUC 202815, 202817 (AEIC); OSUC 211379, 211682, 212175, 212629–212630, 212694, 212700, 212801, 212803, 213025, 213140, 213142, 213180, 213279 (CNCI). **SIERRA LEONE**: 1 female, 1 male, OSUC 244058, 244061 (MZLU). **TANZANIA**: 1 female, OSUC 212513 (CNCI). **UGANDA**: 1 female, 2 males, OSUC 212931, 213021 (CNCI); OSUC 244070 (USNM). **ZIMBABWE**: 4 females, 1 male, OSUC 212201, 212209, 212212, 212827, 212923 (CNCI).

##### Comments.

As delimited here *Scelio taylori* is a widespread and somewhat polymorphic species. At times we considered the material to represent upwards of four species. However, as limits among those concepts blurred, we have elected to take a conservative approach to delimiting this taxon. When comparing opposite extremes (e.g., OSUC 212801 in Figs 97–102 and OSUC 213533 in Figs 109–114) there appears to be clearly differentiable species. However, intermediate series (such as OSUC 213180 IN Figs 103–108) are problematic. The concept of *Scelio taylori* broadly encompasses three core groups: 1) a larger series (to which the type belongs) of slightly smaller individuals, relatively easily diagnosed by the white setae of the gena (Figs 97–102); 2) a series of specimens similar to 1), but darker overall, with the pilosity of the head, pronotal shoulder and lateral T2 brown (Figs 103–108); and 3) four larger, darker (pilosity, body color, wing infuscation) specimens (Figs 109–114). A small series of intermediates blurs the line between 1 and 2, and contains several singletons that may represent additional morphotypes. The three groups are largely geographically separated, series 1 is widespread across central Africa (but absent in South Africa), series 2 is found in eastern Africa (Kenya, Uganda, and possibly Tanzania), and series 3 is from Central African Republic (with one similar specimen from Cameroon). The intermediate specimens are known from Malawi, Nigeria, Ghana, and Ivory Coast.

The pilosity of T1 laterally is relatively reduced, with 4–6 setae typically present along the ventral margin. This is the only species of Afrotropical *ernstii*-group to exhibit truly brown setae on lateral T2, the gena, and throughout the frons (this mostly restricted to individuals of series 2).

Preliminary concepts were based on the following characters: scape color (yellow, light-brown, brown), color of pilosity of the gena (white, light brown to brow), apparent size (small to moderate, large), sculpture of the mesoscutum (moderately sized polygonal cells, large polygonal cells), infuscation of the wing (infuscation reduced to absent around submarginal, marginal, and stigmal veins and elsewhere, infuscation present more or less throughout), form of the submarginal vein near junction with marginal vein (obliterated and nebulous, tubular throughout), form of the stigmal vein (more or less obliterated, well developed tubular and sometimes slightly concave). We now interpret these states as representing a continuum of morphological variation.

While the combination of yellow scape and white pilosity holds for all specimens of group 1, and most specimens of group 2 have light brown scape and brown pilosity on the gena, there are also specimens (intermediates) with brown pilosity on the gena and a yellow scape. Of the two females in group 3 one has a dark brown scape (as does the specimen from Cameroon) and one has a bright yellow scape. The first series, the smallest individuals, all appear to have slightly larger cells on the mesoscutum (compare Figs 99 and 111). Most individuals of the second series have a similar pattern of obliteration of sculpture on the medial metasomal sternites, though this is approached in some of the first series. Individuals of the second series also have the infuscation around the submarginal and stigmal veins reduced, the submarginal vein is nebulous near the marginal vein, and the stigmal vein more or less absent, however these states are also seen in some individuals of the first series. Series three individuals have the wing strongly infuscate throughout and a strongly developed stigma and marginal vein (that has a slight hook), but these states are also observed in some of the “intermediate” specimens. Two specimens from Nigeria (intermediates) have a very slight longitudinal trend to the sculpture of the mesoscutum. Larger specimens tend to have the metasomal 3–5 sculptured throughout medially, while most individuals of the series two, and some of series one have a pattern of obliteration posteromedially on each tergite (particularly noticeable on S3, S4).

All determinations of males are tentative, most have a reduced but identifiable genal carina.

### *Scelio howardi* species group

**Description.** *General*. Body size: moderate; large. Body length: 3.12–6.86 mm. Habitus: typical, mesosoma not dorsoventrally flattened. Body color: brown to dark brown; brown to dark brown, in some with metasoma yellow to orange. Fore leg color: concolorous with mid and hind legs. Sculpture: moderate to robust, reticulate to strigose, generally without longitudinal or parallel lineations. Wing type: macropterous; brachypterous.

*Pilosity*. General setation: moderate elongate and wide, variously vertically oriented. Thickened and truncate white pilosity: not typically present, or strongly truncate when so. Interommatidial pilosity: present; absent. Genal pilosity density: moderate; dense. Genal pilosity color: white. Number of anteclypeal setal pairs: 3. Ventrolateral postgenal cluster of erect setae: unknown. Antespiracular setal patch: very small, intersected by or immediately below lateral epomia. Netrion: setose; glabrous. Propodeal shelf: present throughout except narrow medial strip; narrow strip present laterally. Pilosity of laterotergites: absent.

*Head*. Sculpture of head: predominantly reticulate to rugulose throughout, never predominantly dorsoventral or longitudinal, without prominent smooth or obliterated patches, carinae moderate width. Ocelli size: moderate. Gap between antennal toruli and anteclypeus: narrow to moderate width. Width of ventral head across mandibles: moderate, mandibles typically formed. Anteclypeus shape between outer teeth: rounded trapezoidal, when more angular internal vertices never sharply cornered, medially truncate to slightly concave. Malar sulcus: present. Medial portion of occipital carina: percurrent. Lateral portion of occipital carina: more or less linear throughout. Form of gena: narrow (lateral view), strongly sloped from posterior margin of eye to occipital carina; moderate width (lateral view), evenly rounded from posterior margin of eye to occipital carina. Genal carina: absent; present. RSS on A5 in males: present. Microsculpture at base of mandible: absent. Basal tooth of mandible: absent.

*Mesosoma*. Shape of mesoscutum in lateral view: moderately concave, not particularly bulging in anterior nor flattened throughout. Transverse pronotal carina in female: unknown. Surface of mesoscutum: sculptured throughout, never with smooth patches, always reticulate at least in part, with at most slight longitudinal elements. Smooth or obliterated patches on mesoscutum: absent. Surface of the pronotal collar in females: unknown. Axillula: small, clearly discernible only in lateral view; moderately to well developed, carinate, visible in dorsal view. Propodeal corners: projecting, projection with two vertices, forming a semicircular gap with lateral margin of propodeal nucha. Epomia: present. Surface of oxter: unknown. Fore wing length: not reaching anterior margin of T5; reaching or surpassing anterior margin of T5 but not surpassing apex of metasoma. Fore wing submarginal vein near curve towards costal margin: tubular. Pictation of fore wing in female: absent.

*Metasoma*. Anterior margin of T1: concave, with short rim.

**Diagnosis.** Easily distinguished from all other African *Scelio* by the projected corner of the posterolateral propodeum (Fig. 1) that forms a small semicircular space with the medial propodeal nucha.

**Comments.** For pragmatic purposes we divide the *howardi* species-group into two subgroups, white and brown. This division is largely pragmatic, and in the absence of additional data this division is not meant to hypothesize monophyletic groups. Species in the white subgroup have white pilosity on the frons below the anterior ocellus and the pronotal shoulder, while that on the dorsal head and mesoscutum is white to light golden yellow. Species in the brown subgroup have brown pilosity on the pronotal shoulder, mesoscutum and frons below the anterior ocellus. Most species in the brown subgroup have a brown to dark brown scape whereas species in the white subgroup typically have some yellow on the scape. Within the brown subgroup there is a tendency for the axillula to be well developed, blade-like to lobed. This is never the case in the white group. The pilosity of the occiput is sparse in all species in the group.

**Notes on *Scelio exaratus* Kieffer.** Based on examination of the type (images available at http://hol.osu.edu/index.html?id=5226) *Scelio exaratus* is unique. See comments for *Scelio susurro*.

**Key to howardi-group species** (also available online at http://www.waspweb.org/Platygastroidea/Keys/index.htm)

**Females**

**Table:** 

1	Brachypterous (Fig. 233); metasoma with robust rugulose sculpture laterally (Fig. 238)	*Scelio memorabilis*
–	Macropterous; metasoma with moderately developed striate to reticulate-rugulose sculpture laterally	2
2	Base of metasoma yellow to light brown, then darkening to brown toward apex (Figs 145, 146); coxae yellow; ventral margin of villus straight (Fig. 148); (pilosity of gena sparse to moderate; smaller individuals, Kenya)	*Scelio ululo*
–	Metasoma uniformly colored throughout, never yellow; coxae typically light brown to brown; ventral margin of villus variously formed, but more commonly concave, bent, or angled (e.g. Figs 15, 16, 18, 19)	3
3	Metasoma elongate, wings not reaching T5 (Figs 115, 157) (large individuals)	4
–	Metasoma not elongate, wings typically meeting or surpassing anterior margin of T5	5
4	Ventral margin of villus straight (Fig. 118); frons below anterior ocellus with pilosity white (Fig. 119); gena with dense white pilosity (Fig. 118) (Gabon)	*Scelio fremo*
–	Ventral margin of villus bent medially (Fig. 160); frons below anterior ocellus with some brown pilosity (Fig. 161); gena moderately setose with off-white to golden brown pilosity (D.R. Congo)	*Scelio balo*
5	Netrion setose (Fig. 242); propodeal nucha with sparse setae throughout anterior portion (Fig. 241)	6
–	Netrion glabrous (Fig. 224); propodeal nucha glabrous	7
6	Mesoscutum predominantly sculptured with rounded foveae, pilosity arising from foveae long, extending into other cells, Fig. 244); axillular carina moderately developed, not forming lobed projection posterolaterally	*Scelio philippinensis*
–	Mesoscutum predominantly coarsely rugulose to reticulate, pilosity very short (Fig. 165); axillular carina very well developed, extended posterolaterally into lobed projection (Fig. 168) (gena in lateral view narrow, with genal carina irregularly developed; mandibles narrow, Fig. 167)	*Scelio bubulo*
7	Mesoscutal sculpture consisting predominantly of rounded punctures on an otherwise smooth surface, pilosity arising from punctures very short (Fig. 226); felt fields present on S2, S3 and S4; laterotergites minutely aciculate (Madagascar)	*Scelio effervesco* Yoder, sp. n.
–	Mesoscutal sculpture consisting predominantly of reticulate angular cells, rugulae, or longitudinal strigae, cells not particularly rounded, setae generally longer and more prominent; felt fields present on S2 and S3, absent on S4; laterotergites smooth or sculptured	8
8	Pilosity of frons below anterior ocellus white (e.g. Figs 125, 143, 155); pronotal shoulder predominantly covered with white setae, with at most several brown setae adjacent margin with mesoscutum, setae often slightly lighter than that of mesoscutum (e.g. Figs 25, 27, 29); pilosity of pronotal shoulder usually thick, white, and somewhat truncate at at tips (e.g. Figs 25, 27, 29)	9
–	Pilosity of frons below anterior ocellus golden brown to brown (e.g. Figs 179, 191, 209); pronotal shoulder with some, often all, setae brown (e.g. Figs 28, 30, and as in Fig. 26), setae usually concolorous with those of mesoscutum; thickness and length of pronotal shoulder pilosity variable but sometimes narrower and shorter (e.g. Fig. 30)	14
9	Villus somewhat compact, with ventral margin straight and dorsal margin parallel (Figs 14, 154); pilosity of dorsal head white to very light yellow; gena with dense pilosity at narrowest point in lateral view (Fig. 156); scape and legs usually yellow, in some with slight light brown infuscation	*Scelio zolotarevskyi*
–	Villus variously formed, in most with ventral margin curved or angled, if straight or nearly so then scape brown to dark brown throughout; pilosity of the dorsal head variously colored, but usually golden to light brown; gena with pilosity more or less uniformly distributed throughout, without denser patches; color of scape and legs variable, but hind femur often with brown infuscation medially	10
10	Robust, large (5.04–5.91 mm); dark-brown to nearly black individuals (Figs 139, 140); pilosity of head and mesoscutum completely white, on mesoscutellum off-white, yellowish; T2 laterally with dense white patch of thickened setae (Fig. 144); upper anterior margin of metapleuron with two rows or a slightly broader patch of white setae (Fig. 142); scape dark brown; mesopleural depression with a small smooth patch near mid coxa (Fig. 142) (Madagascar)	*Scelio scomma*
–	Size varying, but typically smaller than 5 mm; color varying, but often with metasoma brown and slightly lighter in color than mesosoma; color of pilosity of head and mesonotum varying, but for those in continental Africa usually golden brown on dorsal surfaces; pilosity of T2 sparse to dense; pilosity of upper anterior edge of metapleuron variable, but often only a single row of setae present; scape variously colored but usually not dark brown; mesopleural depression variously sculptured, with or without smooth patches	11
11	Villus with ventral margin in posterior half bent downwards forming a medial angle (Fig. 16); gena appearing sparsely setose due to somewhat shortened setae that only slightly overlap adjacent cells; pilosity of lateral T2 sparse (Fig. 132); scape brown (lateral T2 usually with reticulate cells surrounding setae; smaller individuals; Madagascar)	*Scelio igland*
–	Shape of villus variable, ventral margin typically not as sharply bent medially but rather slightly concave; pilosity of gena in lateral view varying in density and length; scape color varying, yellow to brown; pilosity of lateral T2 varying (continental Africa)	12
12	Pilosity of lateral T3–T5 predominantly brown; scape completely brown to dark brown, more or less concolorous with flagellum (see Comments for *Scelio destico*); S3 felt field narrow to absent; sculpture of the frons robustly reticulate (Figs 231, 249), reticulations nearly meeting malar sulcus (i.e. facial striae very short to absent) (mesonotum somewhat flattened; eye bulging towards anterodorsal corner, Fig. 230; clava somewhat elongate in most, Fig. 232)	13
–	Pilosity of lateral T3–T5 predominantly white; scape lighter at base (yellow to light brown) to brown at apex, lighter in color than flagellum; S3 felt field well developed; sculpture of frons variable, though almost never uniformly strongly reticulate throughout, facial striae generally longer (Fig. 125)	*Scelio howardi*
13	Gena narrow, with genal carina weakly developed but discernible (Fig. 230); T3–T5 with a sparse row of white setae along ventrolateral margin; S3 felt field extremely reduced to absent; villus relatively long and narrow, with concave lower margin, usually with slight elevation in posterodorsal corner (Fig. 17); size 3.75–4.34 mm (frons slightly concave to flat)	*Scelio destico*
–	Gena broad, genal carina not discernible (Fig. 248); T3–T5 with at most 1–2 white setae along ventrolateral margin; S3 felt field reduced, but never absent; villus with ventral margin straight or extremely slightly curved; size 4.64–5.52 mm	*Scelio pipilo*
14	Pilosity of lateral metasoma more dense towards posterior of each tergite (Fig. 210); frequently with both brown and white setae on a single tergite	*Scelio susurro* Yoder, sp. n.
–	Pilosity of lateral metasoma evenly distributed throughout each tergite, all setae on a given tergite uniform, brown, short and narrow (one exceptional specimen from Madagascar with white setae on lateral T2 but otherwise similar)	15
15	Sculpture of mesonotum irregularly obliterated in humeral area (Fig. 135); pronotal nucha predominantly smooth excluding setigerous punctures; mesopleural depression predominantly smooth (Fig. 136) (Madagascar)	*Scelio ructo*
–	Sculpture of humeral area of mesoscutum uniform, without obliterated patches; pronotal nuchal smooth or sculptured; mesopleural depression with at most a very small smooth patch	16
16	Interantennal process with a well-developed carina medially (Fig. 186, *iapc*); (Uganda)	*Scelio gemo*
–	Interantennal process with surface uninterrupted medially, smooth	17
17	Posteromedial head rounded such that occipital carina obliterated or obscured by similar sized rugae, in most cases a single carina not traceable throughout; T6 sculpture fine parallel longitudinal striae with few reticulations immediately adjacent to apical margin; hind tibia yellow; pilosity of pronotal shoulder slightly lighter colored than that of mesoscutum (Fig. 26); laterotergites with at least some aciculate sculpture (Tanzania, Malawi, D.R. Congo)	*Scelio latro*
–	Posteromedial head relatively abruptly sloped beyond occipital carina, occipital carina percurrent or at most minutely interrupted medially; T6 sculpture variable, rugulose to reticulate, rarely with finer longitudinal elements; hind tibia yellow or yellow and brown; pilosity of pronotal shoulder usually concolorous with that of mesoscutum (Fig. 30); laterotergites smooth or aciculately sculptured	18
18	Hind tibia completely yellow, without trace of infuscation or brown bands; notauli clearly visible posteriorly, a smooth channel without divisions, anteriorly irregularly confused (Fig. 177); sculpture of mesoscutum fine, somewhat more longitudinal than reticulate (Fig. 177); T6 with transverse rugae that are only sparsely reticulate (Fig. 180) (Tanzania)	*Scelio crepo*
–	Hind tibia with at least some brown coloration; notauli variously formed; sculpture of mesoscutum variable but generally more robust (e.g. Figs 171, 189); T6 rugulose reticulate	19
19	Hind tibia entirely brown; course of notauli clearly visible, with medial margin thickened	*Scelio tristis*
–	Hind tibia yellow at least in part; course of notauli variable, but where present without thickened medial margins	20
20	Hind tibia yellow with a short brown band at apex, or light brown throughout; scape color a gradient, yellow to light brown at base, brown at apex (Fig. 191)	21
–	Hind tibia with basal half yellow, apical half brown; scape evenly brown to dark brown throughout (axillula well developed)	22
21	Hind tibia light brown throughout, without darker apex; mesoscutum and propodeum with reticulate sculpture (Fig. 201) laterotergites smooth or nearly so; margin of metasomal sternites sculptured throughout with fine reticulate microsculpture; size 3.20–3.93 mm (South Africa)	*Scelio mutio*
–	Hind tibia yellow with a short brown band at apex (see Comments); mesoscutum and propodeum with thick percurrent longitudinal carinae that form more or less smooth channels with few interrupting transverse elements (Fig. 189); laterotergites with aciculate sculpture, usually throughout; margin of metasomal sternites smooth to very slightly irregular; size 4.69–5.16 mm (Ghana, Guinea, D.R.C)	*Scelio grunnio*
22	Notaulus not clearly indicated, sculpture of mesoscutum confused reticulate (Fig. 174); sculpture of mesonotum and frons (Fig. 173) narrow, somewhat fine; longitudinal carina at base of T2 truncated near base by smooth patches, not continuing more or less throughout length of T2; axillula developed, but not strongly elevated above dorsal surface of mesoscutellum posterolaterally (anterodorsal oblique view) (Uganda)	*Scelio cano*
–	Notaulus indicated posteriorly as smooth strip, sometimes with few irregular carina interrupting its course, not clearly indicated anteriorly; sculpture of mesoscutum with distinct percurrent longitudinal carinae; anteriormost longitudinal carinae of T2 not truncated by smooth patches near base of T2, more or less percurrent throughout T2; axillula well developed, elevated above dorsal surface of mesoscutellum posterolaterally (Cameroon, Central African Republic)	*Scelio tono*

#### “white” subgroup

##### Scelio fremo

Valerio & Yoder sp. n.

http://zoobank.org/85229D8E-72F0-45FD-91C0-B0BAA544A78B

urn:lsid:biosci.ohio-state.edu:osuc_concepts:244969

http://species-id.net/wiki/Scelio_fremo

**Figures 115–120.**
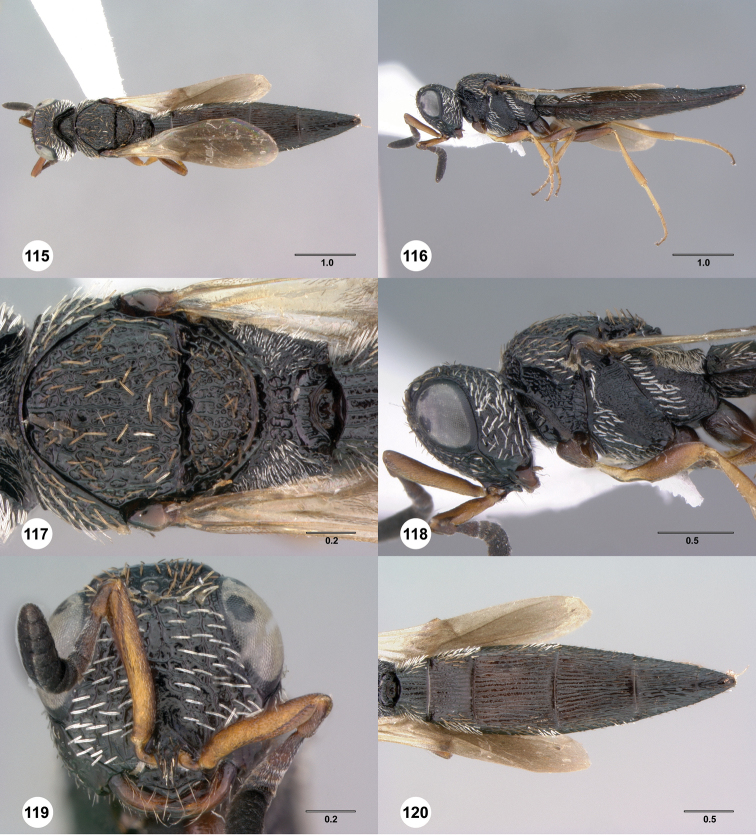
^133^ *Scelio fremo* sp. n., holotype female (OSUC 212795). **115** Habitus, dorsal view **116** Habitus, lateral view **117** Mesosoma, dorsal view **118** Head and mesosoma, lateral view **119** Head, anterior view **120** Metasoma, dorsal view. Scale bars in millimeters.

Figures 115–120 ; Morphbank ^34^

###### Description.

Female body length: 5.60 mm (n=1). Color of pilosity of dorsal head in female: golden to brown. Occipital carina in female: percurrent. Color of pilosity of the frons below the anterior ocellus in female: predominantly white. Pilosity of eye in female: absent. Medial keel on interantennal process: absent. Width of lower gena in lateral view: wide, posterior margin of lower half of gena parallel to posterior orbit. Genal carina: absent. Color of genal pilosity: white. Color of scape in female: yellow throughout. Surface of the pronotal nucha in female: predominantly sculptured. Color of pilosity of pronotal shoulder in female: white to light brown, lighter than that of mesoscutum; golden to dark brown, concolorous with that of mesoscutum. Sculpture of medial mesoscutum in female: predominantly angular reticulate to rugulose. Color of pilosity of mesoscutum in female: predominantly light brown to brown. Notaulus in female: not delimited. Form of axillular carina in female: small, not particularly expanded or projected from the lateral edge of the mesoscutellum. Pilosity of propodeal nucha: absent. Pilosity of netrion: absent. Surface of mesopleural depression in female: sculptured throughout. Form of ventral margin of villus in female: straight. Color of coxae in female: brown. Color of hind femur: light brown throughout. Color of hind tibia: yellow throughout. Fore wing length in female: apex not reaching anterior margin of T5. Color of metasoma: light reddish brown. Sculpture of laterotergites in female: predominantly smooth. Pilosity of laterotergites in female: absent. Sculpture of medial T1 in female: most prominent elements predominantly longitudinal. Sculpture of medial T2 in female: most prominent elements predominantly longitudinal. Pattern of sculpture on T3–T5 in female: T3 predominantly reticulate, T4–T5 predominantly longitudinally striate to strigose. Color of pilosity on lateral T3–T5 in female: more or less evenly split between white and brown. Lateral profile of T6 in female: more or less horizontal. Sculpture of T6 in female: predominantly longitudinally striate to strigose. Sculpture of lateral metasomal sternal bar in female: predominantly smooth to slightly irregularly rugose. Distribution of felt fields: 2 pairs present (S2, S3).

###### Diagnosis.

Similar to *Scelio balo* which also has a long metasoma and fore wings not reaching T5. Differing from *Scelio balo* by the straight ventral margin of the villus (compare Figs 118 and 160) and the white pilosity of the face (Fig. 119, compared with extent of brown pilosity in Fig. 161).

###### Etymology.

Derived from the Latin for roar, howl, grumble; the epithet is considered as a noun in apposition.

###### Link to distribution map.

http://hol.osu.edu/map-large.html?id=244969

###### Material examined.

*Holotype*, female: **GABON**: Estuaire Prov., Citrus orchard, Ntoum, VII-1984, malaise trap, A. Pauly, OSUC 212795 (deposited in CNCI).

###### Comments.

The amount of pilosity on the anterior meso- and metapleuron (Fig. 118) is more than that seen in other Afrotropical *howardi*-group species. The mix of light and dark pilosity on the lateral portions T2 and T3 is also notable. *Scelio fremo* is only superficially similar to *Scelio balo*. *Scelio balo* appears to be more closely related to those species with brown pilosity on the pronotal shoulder than with *Scelio fremo* shares the color pattern more typically seen in *Scelio howardi* and its putative relatives.

##### Scelio howardi

Crawford

http://zoobank.org/FA38C8B4-7723-46E8-9297-529D27ADBE40

urn:lsid:biosci.ohio-state.edu:osuc_concepts:5251

http://species-id.net/wiki/Scelio_howardi

**Figures 121–126.**
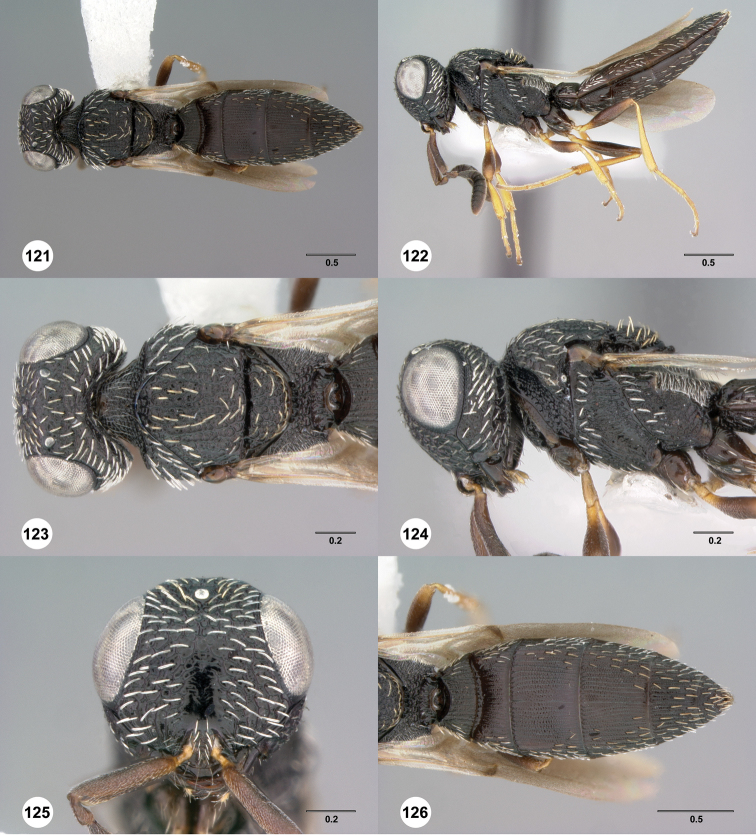
^134^ *Scelio howardi* Crawford, female (OSUC 211309). **121** Habitus, dorsal view **122** Habitus, lateral view **123** Head and mesosoma, dorsal view **124** Head and mesosoma, lateral view **125** Head, anterior view **126** Metasoma, dorsal view. Scale bars in millimeters.

Figures 13 , 121–126 ; Morphbank ^35^

Scelio howardi *Scelio Howardi* Crawford, 1910: 222 (original description).
Scelio howardi *Scelio (Scelio) Howardi* Crawford: Kieffer 1910: 74 (subgeneric assignment).
Scelio howardi Crawford: Kieffer 1926: 310, 323 (description, keyed); Ferrière 1952: 117 (diagnosis); Nixon 1958: 314, 316 (keyed, synonymy); Masner and Muesebeck 1968: 44 (type information).
Scelio aburiensis Kieffer, 1913: 109 (original description); Kieffer 1926: 310, 322 (description, keyed); Nixon 1958: 314 (junior synonym of *Scelio howardi* Crawford); Bin 1974: 464 (type information).
Scelio howardi http://zoobank.org/F07CF0F8-8355-445E-A383-2D7BF288B8BE
Scelio howardi urn:lsid:biosci.ohio-state.edu:osuc_concepts:9727

###### Description.

Female body length: 3.17–5.36 mm (n=29). Male body length: 2.86–5.13 mm (n=27). Color of pilosity of dorsal head in female: golden to brown. Occipital carina in female: percurrent. Color of pilosity of the frons below the anterior ocellus in female: predominantly white. Pilosity of eye in female: present. Medial keel on interantennal process: absent. Width of lower gena in lateral view: wide, posterior margin of lower half of gena parallel to posterior orbit. Genal carina: absent. Color of genal pilosity: white. Color of scape in female: yellow in basal half, darkening to light brown in apical half. Surface of the pronotal nucha in female: predominantly sculptured. Color of pilosity of pronotal shoulder in female: white to light brown, lighter than that of mesoscutum. Sculpture of medial mesoscutum in female: predominantly angular reticulate to rugulose. Color of pilosity of mesoscutum in female: predominantly yellow to golden. Notaulus in female: not delimited; indicated by a row of cells. Form of axillular carina in female: small, not particularly expanded or projected from the lateral edge of the mesoscutellum. Pilosity of propodeal nucha: absent. Pilosity of netrion: absent. Surface of mesopleural depression in female: sculptured throughout. Form of ventral margin of villus in female: bent ventrally in posterior, obviously not straight throughout. Color of coxae in female: brown. Color of hind femur: dark brown with white base. Color of hind tibia: yellow throughout. Fore wing length in female: apex between anterior margin of T5 and posterior margin of T6. Color of metasoma: entirely black. Sculpture of laterotergites in female: predominantly smooth. Pilosity of laterotergites in female: absent. Sculpture of medial T1 in female: most prominent elements predominantly longitudinal. Sculpture of medial T2 in female: most prominent elements predominantly longitudinal. Pattern of sculpture on T3–T5 in female: T3 predominantly reticulate, T4–T5 predominantly longitudinally striate to strigose. Color of pilosity on lateral T3–T5 in female: predominantly white. Lateral profile of T6 in female: more or less horizontal. Sculpture of T6 in female: predominantly rugulose to reticulate. Sculpture of lateral metasomal sternal bar in female: predominantly smooth to slightly irregularly rugose. Distribution of felt fields: 2 pairs present (S2, S3).

###### Diagnosis.

*Scelio howardi* largely may be distinguished from *Scelio zolotarevskyi* by its darkened femur, golden to brown setation, and the darkened apex of the antennal scape.

###### Link to distribution map.

http://hol.osu.edu/map-large.html?id=5251

###### Associations.

Emerged from egg of *Eyprepocnemis plorans plorans* (Charpentier) [Orthoptera: Acrididae (as *Euprepocnemis senegalensis* Bolívar)]; living in *Indigofera brachystachya* (DC.) [Fabales: Fabaceae]; emerged from diapausing eggs of *Kraussaria angulifera* (Krauss) [Orthoptera: Acrididae]; emerged from egg of *Kraussaria angulifera* (Krauss) [Orthoptera: Acrididae]; emerged from egg of *Nomadacris septemfasciata* (Serville) [Orthoptera: Acrididae]; solitary egg parasitoid of *Nomadacris septemfasciata* (Serville) [Orthoptera: Acrididae].

###### Material examined.

*Paratypes*: **MOZAMBIQUE**: 4 females, OSUC 244133–244135, 244137, 244140 (USNM). *Other material*: (590 females, 418 males, 13 unknowns) **ANGOLA**: 1 unknown, BMNH(E)#790376 (BMNH). **BENIN**: 3 females, 19 males, OSUC 211366, 211375, 211787, 212852 (CNCI); OSUC 142649–142655, 142657, 142659–142663, 142665, 142667–142668, 142670–142671 (OSUC). **BOTSWANA**: 62 females, 5 males, OSUC 202821 (AEIC); OSUC 213194 (CNCI); OSUC 164151, 164176, 164205, 166323, 166342–166344, 166347–166349, 166352, 166357, 166359–166360, 166364–166365, 166368–166369, 166371, 166376, 166379–166380, 211625, 212062–212063, 212079, 212088, 212090, 212092, 212094 (OSUC); OSUC 171117–171118, 171121–171123, 171128–171137, 171141, 171149, 171158–171159, 171338, 171340–171341, 171343, 171346, 171349–171351, 171354, 171356–171358, 171369, 171371, 207554, 207558 (USNM). **BURKINA FASO**: 1 male, OSUC 214094 (CNCI). **CAMEROON**: 7 females, OSUC 211216, 211219, 211322, 212117, 212120, 212122, 212127 (CNCI). **CENTRAL AFRICAN REPUBLIC**: 71 females, 116 males, OSUC 212527–212528 (CNCI); OSUC 213969–213970, 213972–213976, 213978, 41792 (OSUC); OSUC 180934, 211400, 211662–211663, 211669, 211676, 211681, 211686–211687, 211826–211830, 211834–211836, 211838, 211840–211844, 211846–211848, 211850–211856, 212396–212398, 213200, 213313–213314, 213316, 213513–213517, 213520–213532, 213570–213573, 213603–213608, 213637, 213639–213640, 213642–213653, 213667–213671, 213909–213915, 213917–213919, 213921–213924, 213926–213927, 213934–213943, 213958–213964, 214190–214193, 214195–214196, 214198, 214200, 214216–214217, 214221–214228, 244018–244024, 244027–244033, 254634–254635, 254638–254646, 254649–254650, 254652–254657, 254659, 254713–254715 (SAMC). **GABON**: 12 females, 3 males, OSUC 212787, 212789–212791, 212793–212794, 213124, 213126–213127, 213157, 213161, 213165–213168 (CNCI). **GHANA**: 3 females, OSUC 213549, 213553, 213569 (OSUC). **GUINEA**: 1 female, OSUC 213135 (CNCI). **ISRAEL**: 2 females, OSUC 218883–218884 (INHS). **IVORY COAST**: 19 females, 8 males, OSUC 211357–211359, 212849, 212853, 212855, 212861, 213095, 213097, 213171–213174 (CNCI); OSUC 142581, 142589, 143121, 234735–234736, 59110–59111, 59118–59119, 59121, 59125, 59131, 59137, 64711 (OSUC). **KENYA**: 69 females, 20 males, OSUC 211351, 212342, 212361, 212509, 212637, 212729, 212949, 212952, 214030, 214033–214034, 214043, 214048, 214080–214082, 214109–214110, 214120, 214124, 214132, 214134, 214178–214179, 234604, 234606, 234614, 234630, 234632, 234639, 234641, 234645, 234647, 234663, 234677, 234679–234682, 234688, 234690–234691, 234695, 234702, 250820, 250822, 250926–250927, 250930, 254706 (CNCI); OSUC 142675, 58958–58959, 59023, 59025, 59030, 59034, 59039–59040, 59043–59044, 59050, 59053–59055, 59057–59059, 59061, 59063, 59066, 59071, 59080, 59084–59085, 59091, 59093, 59095, 59097, 59102, 59105, 70693–70695, 70700, 70702–70704, 70706 (OSUC). MALAWI: 6 females, 2 males, 1 unknown, BMNH(E)#790421 (BMNH); OSUC 212380–212382, 212454, 212465, 212778, 213137, 213197 (CNCI). **MALI**: 3 unknowns, BMNH(E)#790366, 790369, 790422 (BMNH). **MOZAMBIQUE**: 4 females, OSUC 212370, 212734–212735, 213104 (CNCI). **NAMIBIA**: 1 female, 1 male, CASENT 2042536 (CASC); OSUC 213345 (SANC). **NIGERIA**: 53 females, 142 males, OSUC 202818 (AEIC); BMNH(E)#790370 (BMNH); OSUC 211376, 211378, 211381, 211670, 211674–211675, 211677, 211690, 211887, 212159–212162, 212164–212165, 212167–212169, 212171, 212173–212174, 212176, 212180–212181, 212183, 212185–212187, 212189–212193, 212195, 212609–212611, 212615–212617, 212622, 212624, 212627, 212633–212636, 212684, 212686–212687, 212693, 212697–212699, 212701–212705, 212707–212712, 212714, 212717–212728, 212733, 212740, 212804, 212808–212809, 212811, 213026–213029, 213032, 213035–213038, 213123, 213145, 213148–213150, 213152, 213155, 213175, 213179, 213181, 213183, 213186, 213190, 213192–213193, 213256–213257, 213260–213262, 213264, 213268, 213270–213271, 213277–213278, 213280, 250711, 250719, 250724–250725, 250727, 250732, 250734, 250765, 250768–250769, 250771–250772, 250774–250777, 250780, 250782–250786, 250788–250789, 250791, 250794, 250796–250811, 250818, 250950, 250991, 250993, 250997, 251000, 251009–251012, 251014, 251016–251030 (CNCI); OSUC 142618, 244204–244205, 59133, 59136 (OSUC). **SIERRA LEONE**: 1 female, 9 males, OSUC 202823 (AEIC); OSUC 244046, 244048, 244055–244057, 244066–244069 (MZLU). **SOUTH AFRICA**: 169 females, 72 males, 5 unknowns, OSUC 202812, 202819, 202827–202828, 202830–202831 (AEIC); BMNH(E)#790371–790375 (BMNH); OSUC 211207, 211209–211210, 211258, 211261–211262, 211276–211277, 211281, 211283–211284, 211288–211290, 211296, 211299–211303, 211305–211306, 211308–211310, 211312, 211314, 211316–211319, 211323, 211331, 211338, 211383–211385, 211387–211389, 211394, 211923, 211932, 211935, 212109, 212204–212205, 212222, 212235, 212246, 212248, 212269–212274, 212282, 212295, 212299–212300, 212306, 212310–212313, 212323, 212327–212328, 212353, 212356, 212363, 212369, 212384, 212386, 212420, 212423–212425, 212434, 212441–212446, 212463, 212516, 212553, 212558, 212563–212565, 212602–212605, 212650–212651, 212653–212655, 212658–212659, 212674, 212683, 212747, 212753, 212755, 212757, 212760–212761, 212763–212764, 212767, 212771, 212775, 212777, 212779, 212819, 212871, 212874, 212882, 212887, 212892–212894, 212954–212964, 213004, 213079, 213108, 213110–213112, 213118, 213130–213132, 213134, 213253, 234673, 250687, 250696 (CNCI); OSUC 214229 (FMNH); OSUC 244060, 254547 (MZLU); OSUC 142594, 142602, 143288, 176243, 211678, 222130, 222134, 222302, 234724, 234726–234727, 234734, 250975, 250977, 250979 (OSUC); OSUC 211688, 211858–211861, 212800, 213537, 213655, 213673, 213682, 213932, 214203, 214220, 214230–214231, 214245–214247, 234714, 250714, 254712 (SAMC); OSUC 213318–213319, 213322, 213324, 213335–213336, 213359, 213366, 213368, 213372, 213376–213377, 213388–213389, 213400–213401, 213404, 213412–213414, 213418, 213420, 213426, 213431–213432, 213440, 213449, 213451, 213454–213455, 213462, 213464, 213466–213467, 213469, 213474, 213493, 213495, 213497, 213499, 214384, 214386–214387 (SANC). **SWAZILAND**: 1 male, OSUC 244062 (MZLU). **TANZANIA**: 11 females, 4 males, OSUC 212890, 212971, 212975, 212977, 250767, 250954–250957, 250960–250961, 250965, 254629–254630, 254632 (CNCI). **UGANDA**: 1 female, OSUC 250752 (SAMC). **YEMEN**: 4 females, 3 males, OSUC 250938, 251034, 251037, 251046, 251049, 251062, 251066 (CNCI). ZAMBIA: 1 unknown, BMNH(E)#790377 (BMNH). **ZIMBABWE**: 91 females, 12 males, 2 unknowns, OSUC 202820 (AEIC); BMNH(E)#790367–790368 (BMNH); OSUC 211226, 211228, 211232, 211239–211240, 211242–211244, 211246, 211250–211251, 211255, 211346, 211350, 212106–212107, 212134, 212136, 212138, 212141, 212146–212149, 212158, 212207, 212211, 212215, 212218, 212231, 212233, 212239, 212401, 212404, 212406–212408, 212410–212412, 212415, 212568–212569, 212572, 212574, 212582–212584, 212588, 212590–212591, 212593–212594, 212596, 212598–212599, 212639, 212641, 212814–212815, 212825, 212829, 212896–212897, 212905, 212908, 212910–212911, 213001–213002, 213010, 213012, 213041, 213047, 213049–213050, 213052, 213057, 213202, 213204, 213206–213207, 213209, 213211–213213, 213215–213216, 213218, 213244–213248, 213250 (CNCI); OSUC 199567 (FSCA); OSUC 57106, 57110, 57112–57114 (OSUC); OSUC 244074 (USNM).

###### Comments.

The type series of *Scelio howardi* contains two species with 4 and 3 specimens respectively of *Scelio howardi* and *Scelio zolotarevskyi*. Crawford (1910) noted that the leg color is intraspecifically variable. While some slight variation does exist, he did note a relatively useful character in the separation of *Scelio howardi* and *Scelio zolotarevskyi*. The latter very rarely has darker brown infuscations on the femora, while the former nearly always does.

*Scelio howardi* is one of, if not the most commonly collected and widespread species of continental Africa. As circumscribed here it is variable in general size, shape of the head, sculpture, and to a lesser degree color. The sculpture of the frons is highly variable, though it tends to be relatively weakly developed, with broken reticulations common, and usually without robust reticulations meeting the malar sulcus (cf. *Scelio destico*). In a large majority of individuals the base of the scape is yellow; in some cases it is slightly darker to light brown though even then the apex is darker still. The pilosity of the lateral portions T2 is always quite dense and prominent in *Scelio howardi*.

Males usually have a very small smooth patch along the ventral margin of the metapleural depression. Males are particularly similar to those of *Scelio igland*. The two may be distinguished by subtle differences in the form of the villus (more strongly bent medially in *Scelio igland*) and the white and somewhat dense pilosity of the lateral tergites (vs. sparse and golden or brown and sparse in *Scelio igland*).

##### Scelio igland

Yoder sp. n.

http://zoobank.org/49E35DBC-ED0B-43BD-9E17-1611CDF6997E

urn:lsid:biosci.ohio-state.edu:osuc_concepts:244975

http://species-id.net/wiki/Scelio_igland

**Figures 127–132.**
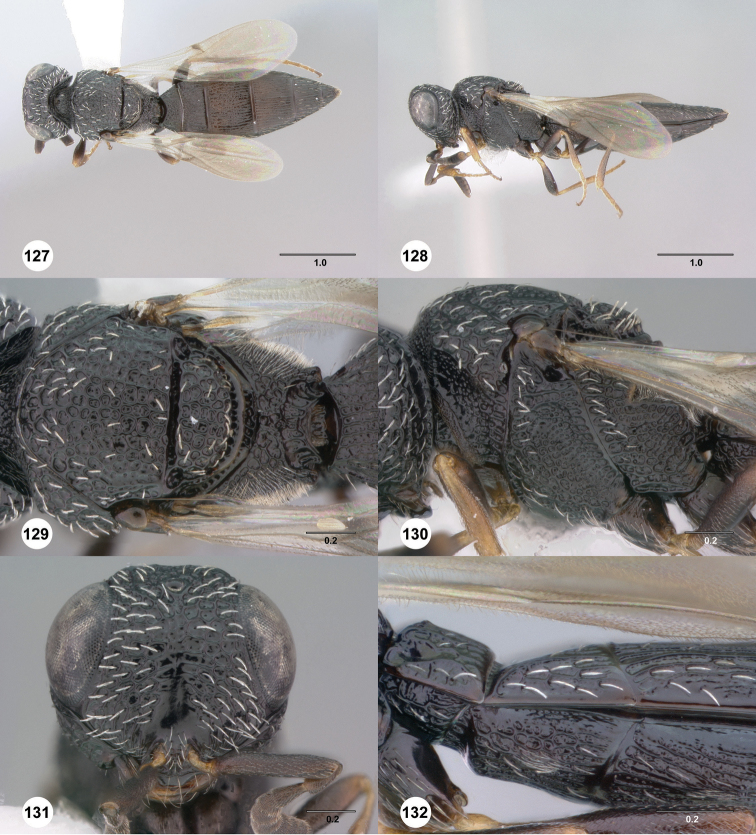
^135^ *Scelio igland* sp. n. **127–131** paratype female (CASENT 2042209), 132 holotype female (CASENT 8106886). **127** Habitus, dorsal view **128** Habitus, lateral view **129** Mesosoma, dorsal view **130** Mesosoma, lateral view **131** Head, anterior view **132** Base of metasoma (segments 1–3), lateral view. Scale bars in millimeters.

Figures 16 , 127–132 ; Morphbank ^36^

###### Description.

Female body length: 3.20–4.74 mm (n=26). Male body length: 3.10–4.28 mm (n=22). Color of pilosity of dorsal head in female: white. Occipital carina in female: percurrent. Color of pilosity of the frons below the anterior ocellus in female: predominantly white. Pilosity of eye in female: present. Medial keel on interantennal process: absent. Width of lower gena in lateral view: wide, posterior margin of lower half of gena parallel to posterior orbit. Genal carina: absent. Color of genal pilosity: white. Color of scape in female: brown to dark brown throughout. Surface of the pronotal nucha in female: predominantly sculptured. Color of pilosity of pronotal shoulder in female: white to light brown, lighter than that of mesoscutum. Sculpture of medial mesoscutum in female: predominantly angular reticulate to rugulose. Color of pilosity of mesoscutum in female: predominantly white to off-white. Notaulus in female: not delimited; indicated by a row of cells. Notaulus in male: not delimited; delimited by row of cells. Form of axillular carina in female: small, not particularly expanded or projected from the lateral edge of the mesoscutellum. Pilosity of propodeal nucha: absent. Pilosity of netrion: absent. Surface of mesopleural depression in female: sculptured throughout. Form of ventral margin of villus in female: bent ventrally in posterior, obviously not straight throughout. Color of coxae in female: brown. Color of hind femur: dark brown with white base. Color of hind tibia: yellow at extreme base, otherwise light brown. Fore wing length in female: apex between anterior margin of T5 and posterior margin of T6. Color of metasoma: entirely dark brown. Sculpture of laterotergites in female: predominantly smooth. Pilosity of laterotergites in female: absent. Sculpture of medial T1 in female: most prominent elements predominantly longitudinal. Sculpture of medial T2 in female: most prominent elements predominantly longitudinal. Pattern of sculpture on T3–T5 in female: T3 predominantly reticulate, T4–T5 predominantly longitudinally striate to strigose. Color of pilosity on lateral T3–T5 in female: predominantly white; predominantly golden to brown. Lateral profile of T6 in female: more or less horizontal. Sculpture of T6 in female: predominantly longitudinally striate to strigose; predominantly rugulose to reticulate. Sculpture of lateral metasomal sternal bar in female: predominantly smooth to slightly irregularly rugose. Distribution of felt fields: 2 pairs present (S2, S3).

###### Diagnosis.

Similar to *Scelio ructo*, with which this species shares relatively sparse pilosity of the head and mesonotum. Differing from *Scelio ructo* and all white subgroup species by the combination of the weakly impressed to absent notauli, the relatively evenly reticulate and not particularly thickened sculpture of the mesonotum, the sculptured mesopleuron, and the medially bent ventral villus.

###### Etymology.

The epithet is used as a noun in apposition derived from the Anglo-Saxon word for island.

###### Link to distribution map.

http://hol.osu.edu/map-large.html?id=244975

###### Material examined.

*Holotype*, female: **MADAGASCAR**: Toliara Auto. Prov., 8km NW Amboasary, gallery forest, MA-02-22-15, Berenty Private Reserve, 25°00.40'S, 46°18.20'E, 85m, 5.II–15.II.2003, malaise trap, M. Irwin, F. Parker & R. Harin’Hala, CASENT 8106886 (deposited in CASC). *Paratypes*: **MADAGASCAR**: 283 females, 412 males, 1 unknown, CASENT 2042052, 2042059-2042060, 2042063-2042064, 2042066-2042067, 2042070-2042071, 2042073-2042074, 2042077, 2042079-2042085, 2042087-2042098, 2042100-2042103, 2042112-2042113, 2042158-2042159, 2042162, 2042170-2042171, 2042174, 2042178, 2042181, 2042186, 2042188, 2042193-2042195, 2042204-2042205, 2042209, 2042257, 2042272, 2042294, 2042299, 2043152, 2043154-2043155, 2043157-2043159, 2043166, 2043310-2043311, 2043319, 2043335-2043338, 2043375-2043376, 2043378, 2043380, 2043382-2043383, 2043387, 2043396-2043397, 2043423, 2043427, 2043429, 2043431, 2043459-2043461, 2043463, 2043465, 2043475, 2043570, 2043572, 2043575, 2043587, 2043618, 2043668-2043670, 2043672, 2043725, 2043929, 2118399, 2132016, 2132021, 2132029, 2132089-2132090, 2132098-2132099, 2132119, 2132135-2132136, 2132139, 2132144, 2132152, 2132157-2132159, 2132164, 2132170-2132171, 2132174-2132175, 2132179, 2132184, 2132188, 2132193, 2132198, 2132205, 2132214-2132215, 2132217, 2132232-2132233, 2132242, 2132262, 2132268-2132269, 2132289, 2132297, 2132300, 2132303-2132305, 2132314-2132315, 2132317, 2132327, 2132331, 2132340, 2132345, 2132354, 2132365, 2132373, 2132381, 2132396, 2132406, 2132412, 2132437-2132446, 2132533, 2132585, 2132590, 2132593-2132594, 2132599, 2132606, 2132631, 2132635, 2132643-2132644, 2132647-2132648, 2132652, 2132654, 2132661, 2132675, 2132692, 2132702, 2132709, 2132711, 2132745, 2132765-2132766, 2132772, 2132775-2132776, 2132790-2132791, 2132808-2132809, 2132822-2132825, 2132829, 2132846, 2132848, 2132863, 2132869, 2132892, 2132900, 2132902, 2132928, 2132935, 2132977, 2132993, 2133187, 2133199, 2133219, 2133222, 2133231, 2133233, 2133241, 2133247, 2133253, 2133256, 2133293, 2133320, 2133355, 2133373, 2133405, 2133434, 2133444, 2133454-2133456, 2133458, 2133460, 2133462-2133465, 2133494, 2133508, 2133510-2133511, 2133539-2133540, 2133543-2133544, 2133546, 2133548, 2133584, 2133596, 2133608, 2133626, 2133629, 2133640, 2133643, 2133652, 2133662, 2133670, 2133673, 2133771, 2133774, 2133776, 2133781-2133782, 2133791, 2133795-2133796, 2133799, 2133945, 2133988, 2134145, 2134194-2134195, 2134224, 2134241-2134242, 2134245, 2134250-2134251, 2134255-2134259, 2134261-2134262, 2134298, 2134344, 2134401, 2134407, 2134447, 2134460, 2134520, 2134526, 2134531-2134532, 2134547-2134548, 2134557-2134560, 2134562-2134563, 2134565-2134566, 2134568, 2134570, 2134581-2134582, 2134590, 2134600, 2134603, 2134607-2134608, 2134624, 2134634-2134635, 2134637-2134638, 2134642-2134643, 2134645-2134646, 2134651, 2134653, 2134657-2134658, 2134695, 2134699-2134705, 2134710-2134712, 2134725, 2134729, 2134740, 2134748, 2134752, 2134765, 2134773, 2134785, 2134789, 2134797-2134799, 2134818, 2134854, 2134861-2134864, 2134871, 2134880, 2135050-2135051, 2135054, 2135056-2135059, 2135061-2135063, 2135065-2135066, 2135142, 2135148, 2135922-2135923, 2135925, 2135927, 2135931, 2135934, 2136569, 2136588, 8097463, 8097467, 8097469, 8097485, 8097495, 8097499, 8106005, 8106010, 8106015, 8106052, 8106065, 8106117, 8106131-8106132, 8106177, 8106227, 8106306, 8106360, 8106380, 8106511, 8106517, 8106529, 8106542, 8106551, 8106582, 8106601, 8106604, 8106627, 8106681, 8106684, 8106687, 8106691, 8106693, 8106699, 8106705-8106706, 8106710, 8106716-8106717, 8106727, 8106751, 8106755, 8106773, 8106780-8106782, 8106789, 8106791-8106792, 8106804, 8106810, 8106812, 8106853, 8106866, 8106892, 8106976, 8106988, 8106992 (CASC); OSUC 212467, 212469, 212531, 212533, 212535-212536 (CNCI); CASENT 2042062, 2042065, 2042069, 2042076, 2042086, 2042099, 2042146-2042157, 2042160-2042161, 2042163-2042169, 2042172-2042173, 2042175-2042177, 2042179-2042180, 2042182-2042185, 2042187, 2042189, 2042191-2042192, 2042196-2042203, 2042206, 2042264, 2042278, 2042289, 2042295-2042296, 2042677, 2043081, 2043200, 2043202, 2043204, 2043253, 2043270, 2043275-2043279, 2043281, 2043320, 2043334, 2043428, 2043464, 2043486, 2043566, 2043573, 2043576, 2043626, 2043628, 2043666-2043667, 2043671, 2043673-2043678, 2132014, 2132221, 2132398, 2133084, 2133096, 2133110, 2133177, 2133186, 2133188, 2133191, 2133198, 2133200, 2133202, 2133237-2133240, 2133246, 2133255, 2133257-2133259, 2133309, 2133340, 2133356, 2133358, 2133360-2133362, 2133364-2133367, 2133378, 2133431, 2133445, 2133457, 2133459, 2133461, 2133493, 2133499, 2133507, 2133509, 2133512-2133513, 2133515, 2133542, 2133547, 2133551, 2133565, 2133586-2133587, 2133589, 2133595, 2133602, 2133627, 2133634, 2133654, 2133664, 2133666, 2133675, 2133678, 2133692-2133693, 2133696, 2133699, 2133704, 2133710, 2133713, 2133727-2133728, 2133745, 2133753, 2133760, 2133767, 2133805, 2133823, 2133828, 2133836, 2133840-2133841, 2133854, 2133858, 2133863, 2133865-2133866, 2133874-2133875, 2133950, 2133960, 2133968, 2133973, 2133979-2133980, 2133999-2134000, 2134017, 2134020, 2134032, 2134036-2134037, 2134040-2134041, 2134222-2134223, 2134252, 2134319, 2134368, 2134385, 2134446, 2134451-2134452, 2134458-2134459, 2134461, 2134525, 2134527, 2134538-2134543, 2134546, 2134549-2134551, 2134561, 2134564, 2134606, 2134644, 2134665, 2134706, 2134770, 2134837, 2134889, 2135978, 2136127, 2136421, 2136570, 2136572, 2136578, 2136581, 8106221, 8106262, 8106308, 8106311, 8106357, 8106507, 8106620, 8106642, 8106669, 8106704, 8106814, 8106849, 8106877, OSUC 211202 (OSUC); OSUC 210183-210184 (UCDC); OSUC 244080 (USNM). Other material: MADAGASCAR: 1 female, 1 male, OSUC 244188a, 244192b (MNHN). *Other material*: **MADAGASCAR**: 1 female, 1 male, OSUC 244188a, 244192b (MNHN).

###### Comments.

The pilosity of the mesonotum and dorsal pronotal shoulders is relatively sparse (Fig. 129) in comparison to other species in the white subgroup. *Scelio igland* is slightly variable in the size and nature of sculpture, particularly on the mesonotum. In larger individuals the course of the notauli is sometimes slightly impressed, but never to the extent seen in *Scelio ructo* (Fig. 138). The shape of the head is somewhat ovoid in lateral view in comparison to the slightly more wedge-shaped form seen in *Scelio ructo*. In the long series of available material there is almost no variation in the degree of sculpture on the mesopleural depression (Fig. 136) and the presence of sculpture on the pronotal nucha. Males have a somewhat more compact flagellum than typically seen in the *howardi* species group. In males patches of obliterated sculpture are more common than in males of *Scelio howardi*. *Scelio igland* is very commonly collected in Madagascar.

There are two specimens from the Muséum National d’Histoire Naturelle mounted with other specimens on the same pins. They are excluded from the type series in order to try to avoid future confusions.

##### Scelio ructo

Yoder sp. n.

http://zoobank.org/9F7C84FE-823C-4457-8721-A46CEF5FBDB2

urn:lsid:biosci.ohio-state.edu:osuc_concepts:244974

http://species-id.net/wiki/Scelio_ructo

**Figures 133–138.**
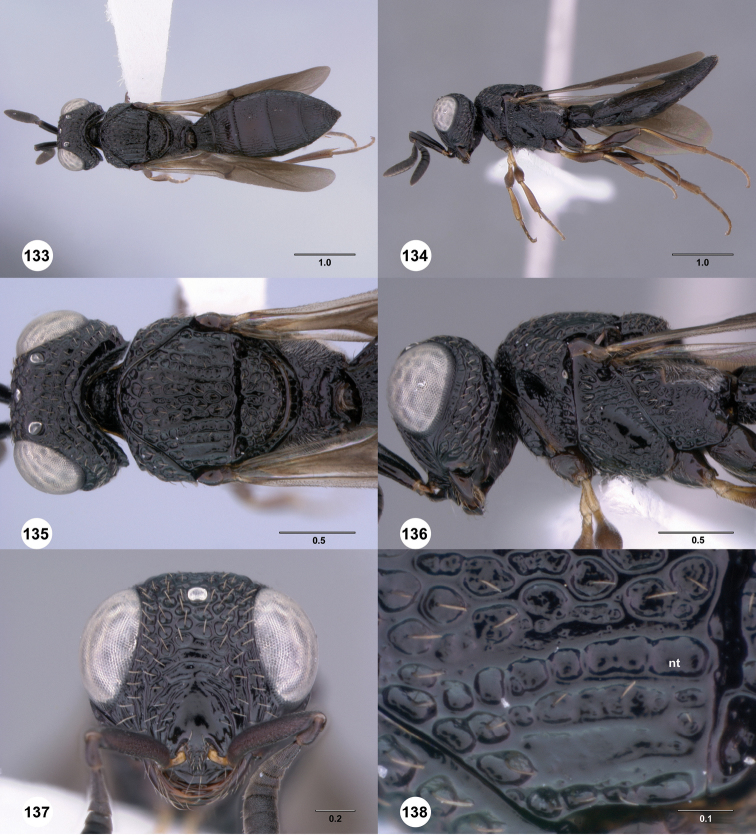
^136^ *Scelio ructo* sp. n., holotype female (OSUC 2042190). **133** Habitus, dorsal view **134** Habitus, lateral view **135** Head and mesosoma, dorsal view **136** Head and mesosoma, lateral view **137** Head, anterior view **138** Lateral mesoscutum, dorsal view. *nt*, notaulus. Scale bars in millimeters.

Figures 133–138 ; Morphbank ^37^

###### Description.

Female body length: 3.12–5.21 mm (n=10). Male body length: 3.56–4.66 mm (n=4). Color of pilosity of dorsal head in female: golden to brown. Occipital carina in female: percurrent. Color of pilosity of the frons below the anterior ocellus in female: predominantly golden to brown. Pilosity of eye in female: present. Medial keel on interantennal process: absent. Width of lower gena in lateral view: wide, posterior margin of lower half of gena parallel to posterior orbit. Genal carina: present. Color of genal pilosity: white. Color of scape in female: brown to dark brown throughout. Surface of the pronotal nucha in female: predominantly smooth. Color of pilosity of pronotal shoulder in female: golden to dark brown, concolorous with that of mesoscutum. Sculpture of medial mesoscutum in female: predominantly angular reticulate to rugulose. Color of pilosity of mesoscutum in female: predominantly light brown to brown. Notaulus in female: indicated by a row of cells. Notaulus in male: delimited by row of cells. Form of axillular carina in female: small, not particularly expanded or projected from the lateral edge of the mesoscutellum. Pilosity of propodeal nucha: absent. Pilosity of netrion: absent. Surface of mesopleural depression in female: with large smooth patch. Form of ventral margin of villus in female: bent ventrally in posterior, obviously not straight throughout. Color of coxae in female: brown. Color of hind femur: dark brown throughout. Color of hind tibia: yellow at extreme base, otherwise light brown. Fore wing length in female: apex between anterior margin of T5 and posterior margin of T6; apex surpassing posterior margin of T6. Color of metasoma: light reddish brown. Sculpture of laterotergites in female: predominantly smooth. Pilosity of laterotergites in female: absent. Sculpture of medial T1 in female: most prominent elements predominantly longitudinal. Sculpture of medial T2 in female: most prominent elements predominantly longitudinal. Pattern of sculpture on T3–T5 in female: predominantly longitudinally striate. Color of pilosity on lateral T3–T5 in female: predominantly golden to brown. Lateral profile of T6 in female: more or less horizontal. Sculpture of T6 in female: predominantly rugulose to reticulate. Sculpture of lateral metasomal sternal bar in female: predominantly smooth to slightly irregularly rugose. Distribution of felt fields: 2 pairs present (S2, S3).

###### Diagnosis.

Most similar to *Scelio igland* which shares the somewhat sparse pilosity of the head and mesonotum (Fig. 135) and is also restricted to Madagascar. *Scelio ructo* differs from this and all other Afrotropical *howardi*-group species by the flattened and smoothly rounded sculpture of the mesoscutum (Fig. 135) in combination with the well-impressed notauli and the smooth pronotal nucha and mesopleural depression.

###### Etymology.

The epithet is used as a noun in apposition derived from the Latin word for belch.

###### Link to distribution map.

http://hol.osu.edu/map-large.html?id=244974

###### Material examined.

*Holotype*, female: **MADAGASCAR**: Antsiranana Auto. Prov., MA-01-01D-11, Montagne d’Ambre National Park, 12°31'13"S, 49°10'45"E, 1125m, 14.V–30.V.2001, malaise trap, R. Harin’Hala, CASENT 2042190 (deposited in CASC). *Paratypes*: **MADAGASCAR**: 16 females, 4 males, CASENT 2132545–2132546, 2133133, 2133136–2133137, 2133139, 2133275, 2134126, 2134225, 2134244, 2134260, 2134553, 2134579, 2134588–2134589, 8106002 (CASC); CASENT 2043853 (CNCI); CASENT 2133210, 2134554, 8106000 (OSUC). *Other material*: **MADAGASCAR**: 1 female, CASENT 2043185 (CASC).

###### Comments.

The notauli in *Scelio ructo* are particularly well developed for species of Afrotropical *Scelio*. The material includes a short series of distinctly smaller but morphologically consistent individuals (CASENT 2133137, 2133136, 2134579, 2134588). The size of the smooth patches on the dorsal metasoma is highly variable. A single larger individual (CASENT 2043185) is identified as *Scelio ructo* but excluded from the type series. The specimen is missing the large smooth patch of the mesopleuron, and the gena is somewhat broader. Overall, its similar mesoscutal sculpture and general habitus suggests that it represents a morphologically larger outlier. The surface where a third pair of felt fields on S4 would be expected is slightly pinched and raised; nevertheless, lacking the setae we have coded the felt fields as absent on this sclerite. When the structure and function of of the felt fields is discovered this character should be reviewed for *Scelio ructo*. Females have a slightly wedge-shaped head with a moderate to narrow gena, often with an irregularly developed genal carina.

##### Scelio scomma

Yoder sp. n.

http://zoobank.org/34244FAF-132C-47F5-A3F0-1CC0DB1BDE3D

urn:lsid:biosci.ohio-state.edu:osuc_concepts:244973

http://species-id.net/wiki/Scelio_scomma

Figures 15 , 27 , 139–144 ; Morphbank ^38^

###### Description.

Female body length: 5.04–5.91 mm (n=16). Male body length: 4.66–5.42 mm (n=7). Color of pilosity of dorsal head in female: white. Occipital carina in female: percurrent. Color of pilosity of the frons below the anterior ocellus in female: predominantly white. Pilosity of eye in female: present. Medial keel on interantennal process: absent. Width of lower gena in lateral view: wide, posterior margin of lower half of gena parallel to posterior orbit. Genal carina: absent. Color of genal pilosity: white. Color of scape in female: brown to dark brown throughout. Surface of the pronotal nucha in female: predominantly sculptured. Color of pilosity of pronotal shoulder in female: white to light brown, lighter than that of mesoscutum. Sculpture of medial mesoscutum in female: predominantly angular reticulate to rugulose. Color of pilosity of mesoscutum in female: predominantly white to off-white. Notaulus in female: not delimited. Notaulus in male: not delimited. Form of axillular carina in female: small, not particularly expanded or projected from the lateral edge of the mesoscutellum. Pilosity of propodeal nucha: absent. Pilosity of netrion: absent. Surface of mesopleural depression in female: with small smooth patch ventrally. Form of ventral margin of villus in female: bent ventrally in posterior, obviously not straight throughout. Color of coxae in female: brown. Color of hind femur: dark brown throughout. Color of hind tibia: yellow at extreme base, otherwise light brown. Fore wing length in female: apex between anterior margin of T5 and posterior margin of T6. Color of metasoma: entirely dark brown. Sculpture of laterotergites in female: predominantly smooth. Pilosity of laterotergites in female: absent. Sculpture of medial T1 in female: most prominent elements predominantly longitudinal. Sculpture of medial T2 in female: most prominent elements predominantly longitudinal. Pattern of sculpture on T3–T5 in female: T3 predominantly reticulate, T4–T5 predominantly longitudinally striate to strigose. Color of pilosity on lateral T3–T5 in female: predominantly white. Lateral profile of T6 in female: more or less horizontal. Sculpture of T6 in female: predominantly longitudinally striate to strigose. Sculpture of lateral metasomal sternal bar in female: predominantly smooth to slightly irregularly rugose. Distribution of felt fields: 2 pairs present (S2, S3).

###### Diagnosis.

Similar to other white subgroup species, in particular to *Scelio zolotarevskyi* and larger individuals of *Scelio howardi*. Differing from the former by the curved ventral margin of the villus, and from the latter by its large size and predominantly white setae of the dorsal head and mesoscutum (*Scelio howardi* usually with some golden setae present) together with the brown to dark brown scape (*Scelio howardi* usually with at least the base of scape yellow) and the presence of a small smooth patch on the lower extent of the mesopleural depression (Fig. 142) (*Scelio howardi* sculptured throughout).

**Figures 139–144.**
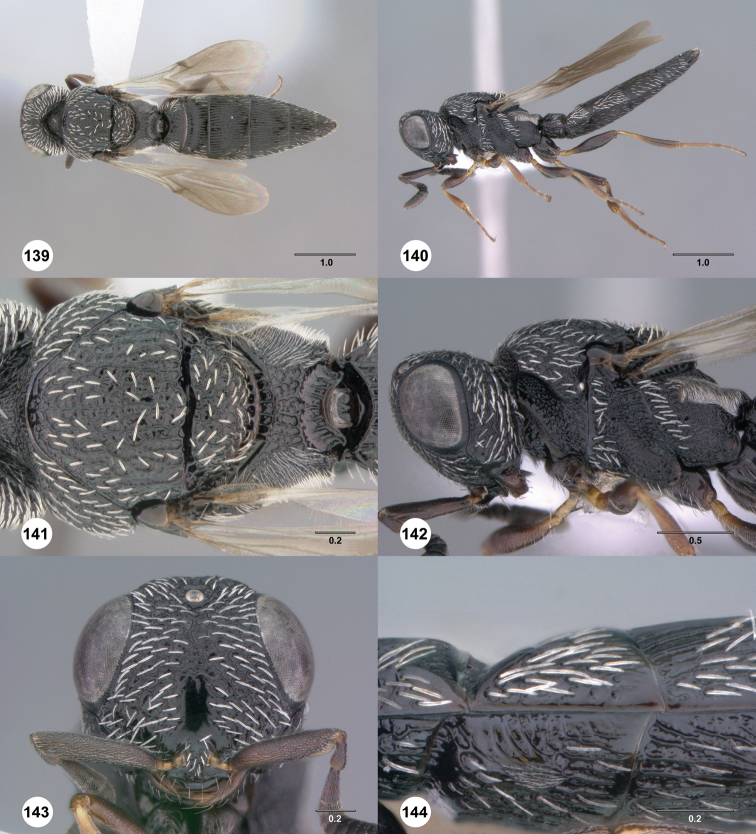
^137^ *Scelio scomma* sp. n. **139** Habitus, dorsal view, paratype female (CASENT 2042253) **140** Habitus, lateral view, holotype female (CASENT 2133165) **141** Mesosoma, dorsal view, paratype female (CASENT 2042253) **142** Head and mesosoma, lateral view, holotype female (CASENT 2133165) **143** Head, anterior view, holotype female (CASENT 2133165) **144** Metasomal segment 2, lateral view, paratype female (CASENT 2042253). Scale bars in millimeters.

###### Etymology.

The epithet is used as a noun in apposition derived from the Latin word for taunt, jeer, or jest.

###### Link to distribution map.

http://hol.osu.edu/map-large.html?id=244973

###### Material examined.

*Holotype*, female: **MADAGASCAR**: Toliara Auto. Prov., Andohahela N.P., parcel II, transitional forest, MA-02-20-52, Tsimelahy, 24°56.21'S, 46°37.60'E, 180m, 15.I–28.I.2004, malaise trap, M. E. Irwin, F. Parker & R. Harin’Hala, CASENT 2133165 (deposited in CASC). *Paratypes*: **MADAGASCAR**: 16 females, 7 males, CASENT 2043612–2043614, 2043909, 2132679, 2132894, 2133068, 2133071, 2133073, 2133145, 2133153, 2133156, 2134231, 2134276, 2134890, 8106504, 8106513, 8106531, 8106840 (CASC); CASENT 2042253, 2043462, 2133142, 8106546 (OSUC).

###### Comments.

*Scelio scomma* is endemic to Madagascar where it is the largest species within the *howardi*-species group and large for species within the white subgroup. All the specimens are uniform in morphology. *Scelio scomma* is extremely similar to larger individuals of *Scelio howardi* and the two cannot be casually differentiated. The pilosity of the lateral portions T2 is particularly dense (Fig. 144). The small smooth patch on the lower mesopleural depression (Fig. 142) is constant for all individuals observed, and it is present in both males and females. Males can be confused with *Scelio igland*, but may be differentiated by their larger and more robust habitus, completely sculptured lateral metasoma (smooth or obliterated patches present in *Scelio igland*), and the absence of notauli (indicated by a row of cells in *Scelio igland*).

##### Scelio ululo

Yoder sp. n.

http://zoobank.org/48EFD5FD-99F5-47EB-8E88-E338F4C61A17

urn:lsid:biosci.ohio-state.edu:osuc_concepts:244977

http://species-id.net/wiki/Scelio_ululo

**Figures 145–150.**
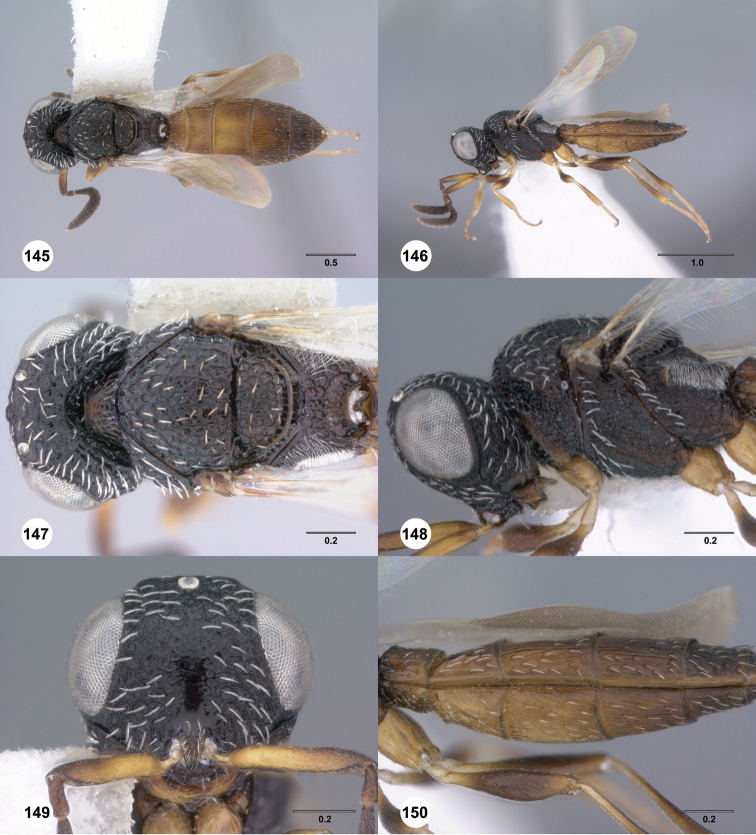
^138^ *Scelio ululo* sp. n., holotype female (OSUC 244090). **145** Habitus, dorsal view **146** Habitus, lateral view **147** Head and mesosoma, dorsal view **148** Head and mesosoma, lateral view **149** Head, anterior view **150** Metasoma, lateral view. Scale bars in millimeters.

Figures 145–150 ; Morphbank ^39^

###### Description.

Female body length: 3.30–3.76 mm (n=5). Color of pilosity of dorsal head in female: white. Occipital carina in female: percurrent. Color of pilosity of the frons below the anterior ocellus in female: predominantly white. Pilosity of eye in female: absent; present. Medial keel on interantennal process: absent. Width of lower gena in lateral view: wide, posterior margin of lower half of gena parallel to posterior orbit. Genal carina: absent. Color of genal pilosity: white. Color of scape in female: yellow in basal half, darkening to light brown in apical half. Surface of the pronotal nucha in female: predominantly sculptured. Color of pilosity of pronotal shoulder in female: white to light brown, lighter than that of mesoscutum. Sculpture of medial mesoscutum in female: predominantly longitudinally strigose to rugulose. Color of pilosity of mesoscutum in female: predominantly yellow to golden. Notaulus in female: not delimited. Form of axillular carina in female: small, not particularly expanded or projected from the lateral edge of the mesoscutellum. Pilosity of propodeal nucha: absent. Pilosity of netrion: absent. Surface of mesopleural depression in female: sculptured throughout. Form of ventral margin of villus in female: straight. Color of coxae in female: yellow. Color of hind femur: light brown throughout. Color of hind tibia: yellow throughout. Fore wing length in female: apex between anterior margin of T5 and posterior margin of T6. Color of metasoma: yellow anteriorly, grading to brown posteriorly. Sculpture of laterotergites in female: predominantly smooth. Pilosity of laterotergites in female: absent. Sculpture of medial T1 in female: most prominent elements predominantly longitudinal. Sculpture of medial T2 in female: most prominent elements predominantly longitudinal. Pattern of sculpture on T3–T5 in female: T3 predominantly reticulate, T4–T5 predominantly longitudinally striate to strigose; predominantly longitudinally striate. Color of pilosity on lateral T3–T5 in female: predominantly white; predominantly golden to brown. Lateral profile of T6 in female: more or less horizontal. Sculpture of T6 in female: predominantly transversely rugose. Sculpture of lateral metasomal sternal bar in female: predominantly smooth to slightly irregularly rugose. Distribution of felt fields: 2 pairs present (S2, S3).

###### Diagnosis.

Most similar to *Scelio zolotarevskyi* which shares the straight ventral margin of the villus. Differing from *Scelio zolotarevskyi* and all other Afrotropical *howardi*-group species by the yellow to brown color gradient of the metasoma (vs. uniform coloration). Further, females may be distinguished from most Afrotropical *Scelio howardi* by the yellow coxae.

###### Etymology.

The epithet is used as a noun in apposition derived from the Latin word for to make a loud cry.

###### Link to distribution map.

http://hol.osu.edu/map-large.html?id=244977

###### Material examined.

*Holotype*, female: **KENYA**: Nyanza Prov., Ungoye (ICIPE) Field Site, 2° forest, Lake Victoria, 0.615°S, 34.092°E, 1145m, 9.II-16.II.1999, malaise trap, S. Miller & P. Otieno, OSUC 244090 (deposited in NMKE). *Paratypes*: (4 females) **KENYA**: 3 females, OSUC 214091 (CNCI); OSUC 244087–244088 (USNM). **ZIMBABWE**: 1 female, OSUC 212155 (CNCI).

###### Comments.

Individuals of *Scelio ululo* are smaller than most *Scelio zolotarevskyi* and also have distinctly less pilosity on the gena. In the ample material of *Scelio zolotarevskyi* there is no hint of color variation of the metasoma. The inner margin of the notauli is slightly indicated in some individuals, though we have coded the notauli as absent. As in *Scelio zolotarevskyi* there appears to be short setae between the ommatidia in individuals with silver eyes, in those with black eyes these cannot be seen but are likely present. The color of the coxae in one individual is slightly darker yellow, but distinctly lighter than the brown color of the corresponding femora.

##### Scelio zolotarevskyi

Ferrière

http://zoobank.org/BFEF6AA9-7916-4CDD-B2E0-43FB0C1F659E

urn:lsid:biosci.ohio-state.edu:osuc_concepts:5364

http://species-id.net/wiki/Scelio_zolotarevskyi

Figures 1 , 14 , 29 , 151–156 ; Morphbank ^40^

Scelio zolotarevskyi Ferrière, 1930: 42 (original description); Ferrière 1952: 117 (diagnosis); Masner 1965: 96 (type information).
Scelio sudanensis Ferrière, 1952: 115 (original description), new synonymy; Nixon 1958: 314, 315 (keyed).
Scelio zolotarevskyi http://zoobank.org/2356D29C-854A-4F82-B421-7A318C77E47C
Scelio zolotarevskyi urn:lsid:biosci.ohio-state.edu:osuc_concepts:5340
Scelio cheops Nixon, 1958: 315 (original description) syn. n.; Masner 1965: 92 (type information)
Scelio zolotarevskyi http://zoobank.org/938667BC-30CC-45F3-A0B9-BD95D0E95E52
Scelio zolotarevskyi urn:lsid:biosci.ohio-state.edu:osuc_concepts:5195

###### Description.

Female body length: 3.53–5.53 mm (n=28). Male body length: 3.28 mm (n=1). Color of pilosity of dorsal head in female: white. Occipital carina in female: percurrent. Color of pilosity of the frons below the anterior ocellus in female: predominantly white. Pilosity of eye in female: absent; present. Medial keel on interantennal process: absent. Width of lower gena in lateral view: wide, posterior margin of lower half of gena parallel to posterior orbit. Genal carina: absent. Color of genal pilosity: white. Color of scape in female: yellow throughout. Surface of the pronotal nucha in female: predominantly sculptured. Color of pilosity of pronotal shoulder in female: white to light brown, lighter than that of mesoscutum. Sculpture of medial mesoscutum in female: predominantly angular reticulate to rugulose. Color of pilosity of mesoscutum in female: predominantly white to off–white. Notaulus in female: indicated by a row of cells. Notaulus in male: delimited by row of cells. Form of axillular carina in female: small, not particularly expanded or projected from the lateral edge of the mesoscutellum. Pilosity of propodeal nucha: absent. Pilosity of netrion: absent. Surface of mesopleural depression in female: sculptured throughout. Form of ventral margin of villus in female: straight. Color of coxae in female: brown. Color of hind femur: dark brown medially, otherwise honey yellow. Color of hind tibia: yellow throughout. Fore wing length in female: apex between anterior margin of T5 and posterior margin of T6. Color of metasoma: entirely dark brown. Sculpture of laterotergites in female: predominantly smooth. Pilosity of laterotergites in female: absent. Sculpture of medial T1 in female: most prominent elements predominantly longitudinal. Sculpture of medial T2 in female: most prominent elements predominantly longitudinal. Pattern of sculpture on T3–T5 in female: T3 predominantly reticulate, T4–T5 predominantly longitudinally striate to strigose. Color of pilosity on lateral T3–T5 in female: predominantly white. Lateral profile of T6 in female: more or less horizontal. Sculpture of T6 in female: predominantly longitudinally striate to strigose. Sculpture of lateral metasomal sternal bar in female: predominantly smooth to slightly irregularly rugose. Distribution of felt fields: 2 pairs present (S2, S3).

###### Diagnosis.

Most similar to *Scelio howardi* in its habitus, color and form of pilosity, and general sculpture, as well as to *Scelio pipilo* with which it shares the straight ventral margin of the villus (as in Fig. 8). *Scelio zolotarevskyi* may be distinguished from the former by the compact and parallel-sided villus (Figs 8, 155, compare with concave or bent lateral margin of villus, Fig. 124) and from the latter by its smaller size and color of the scape (never completely brown to dark brown scape).

**Figures 151–156.**
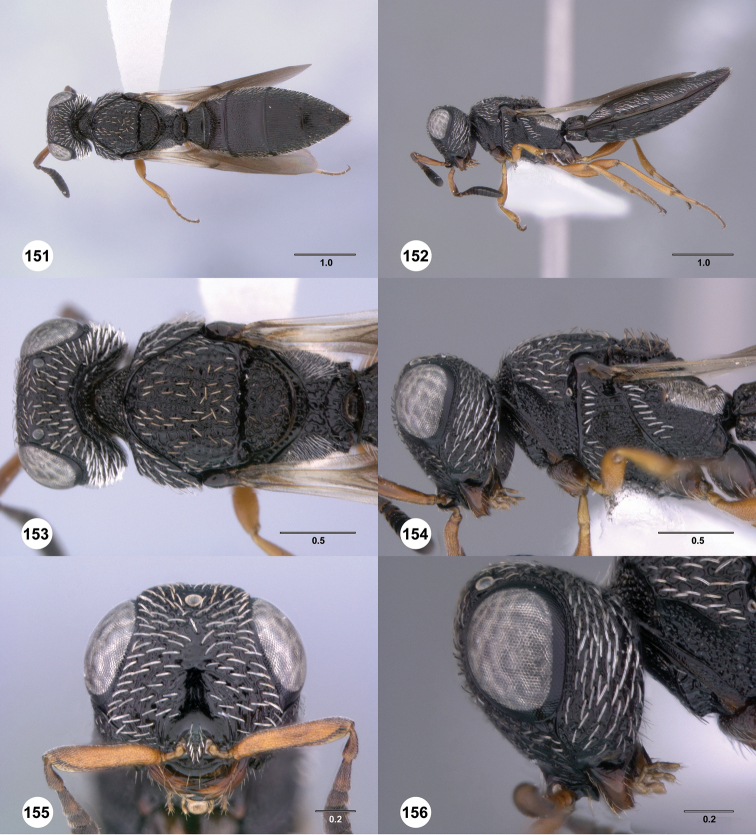
^139^ *Scelio zolotarevskyi* Ferrière, female (OSUC 213559). **151** Habitus, dorsal view **152** Habitus, lateral view **153** Head and mesosoma, dorsal view **154** Head and mesosoma, lateral view **155** Head, anterior view **156** Head, lateral view. Scale bars in millimeters.

###### Link to distribution map.

http://hol.osu.edu/map-large.html?id=5364

###### Associations.

Emerged from Acrididae [Orthoptera: Acrididae]; emerged from egg of *Acrotylus* Fieber [Orthoptera: Acrididae]; emerged from ootheca of *Acrotylus patruelis* (Herrich-Schaeffer) [Orthoptera: Acrididae]; emerged from ootheca of *Cyrtacanthacris tatarica tatarica* (Linnaeus) [Orthoptera: Acrididae]; emerged from egg of *Eyprepocnemis plorans plorans* (Charpentier) [Orthoptera: Acrididae, as *Euprepocnemis senegalensis* Bolívar]; emerged from egg of *Eyprepocnemis smaragdipes* Bruner [Orthoptera: Acrididae]; emerged from ootheca of *Eyprepocnemis smaragdipes* Bruner [Orthoptera: Acrididae]; emerged from egg of *Gastrimargus africanus* (Saussure) [Orthoptera: Acrididae]; emerged from ootheca of *Gastrimargus africanus* (Saussure) [Orthoptera: Acrididae]; emerged from ootheca of *Heteracris* Walker [Orthoptera: Acrididae]; emerged from egg of *Locusta* Linnaeus [Orthoptera: Acrididae]; emerged from egg of *Locusta cinerascens capito* Saussure [Orthoptera: Acrididae, as *Locusta migratoria capito*]; emerged from ootheca of *Locusta cinerascens capito* Saussure [Orthoptera: Acrididae, as *Locusta migratoria capito*]; emerged from egg of *Locusta cinerascens migratorioides* (Reiche & Fairmaire) [Orthoptera: Acrididae, as *Locusta migratorioides*]; emerged from egg of *Nomadacris* Uvarov [Orthoptera: Acrididae]; emerged from ootheca of *Nomadacris septemfasciata* (Serville) [Orthoptera: Acrididae]; emerged from ootheca of *Oedaleus virgula* (Snellen van Vollenhoven) [Orthoptera: Acrididae].

###### Material examined.

*Lectotype* (**present designation**), female, *Scelio zolotarevskyi*: **MADAGASCAR**: Toliara Auto. Prov., Ejeda, 18.II.1928, Zolotarevsky, B.M. TYPE HYM. 9.535 (deposited in BMNH). *Holotype*, female, *Scelio cheops*: [**MALI**]: Central flood plain of Niger R[iver], i-iii.1956, G. Popov, ex acridid eggs CIE Coll 14777 A/502, ex *Eupropocnemis senegalensis*, B.M. TYPE HYM 9.537 (deposited in BMNH). *Lectotype* (**present designation**), female, *Scelio sudanensis*: **MALI**: Dogo, II-1950, Remaudière, OSUC 173946 (deposited in MNHN). *Paralectotype*, *Scelio zolotarevskyi*: **MADAGASCAR**: 1 male, BMNH(E)#790468 (BMNH). *Other material*: (541 females, 125 males, 9 unknowns) **BENIN**: 2 females, OSUC 211365, 212850 (CNCI). **BOTSWANA**: 15 females, 3 males, OSUC 160051, 160053 (EMEC); OSUC 164132, 164134, 164137, 164140, 164145, 164165–164166, 164171, 164189, 164193, 164200, 164215, 211689 (OSUC); OSUC 171353, 207550–207551 (USNM). **ERITREA**: 1 male, 1 unknown, BMNH(E)#790463 (BMNH); OSUC 210357 (MCSN). **GHANA**: 7 females, OSUC 176009, 213548, 213551, 213559–213561, 213564 (OSUC). **KENYA**: 9 females, 1 male, CASENT 2042600 (CASC); OSUC 212343, 212357–212358, 234631, 234648, 234678 (CNCI); OSUC 244091–244092, 248101 (USNM). **MADAGASCAR**: 443 females, 98 males, 1 unknown, CASENT 2042061, 2042068, 2042072, 2042075, 2042078, 2042672, 2043323, 2043417, 2043560, 2043631–2043632, 2133514, 2133516, 2134571, 2134709 (CASC); OSUC 254614, 259910–259998, 260001–260019, 260021–260062, 260263–260268, 261063–261193, 261195, 261199–261340, 261359–261380, 261387–261414, 261417–261462 (MNHN). **MALI**: 6 unknowns, BMNH(E)#790459–790461, 790464–790466 (BMNH). **MOZAMBIQUE**: 3 females, 3 males, OSUC 212371–212374, 212378 (CNCI); OSUC 244138 (USNM). **NIGERIA**: 1 female, 5 males, OSUC 212614, 212618–212621, 212623 (CNCI). RWANDA: 1 female, OSUC 182054 (RMCA). **SOUTH** **AFRICA**: 5 females, 2 males, OSUC 212451, 212872 (CNCI); OSUC 213399, 213468, 213487, 214383, 214385 (SANC). **TANZANIA**: 1 unknown, BMNH(E)#790462 (BMNH). **UNITED ARAB EMIRATES**: 8 females, OSUC 214052–214053, 214064, 214066–214068, 214071, 214079 (CNCI). **YEMEN**: 47 females, 12 males, OSUC 212479–212480, 212486–212487, 212489, 212939, 250671, 250676, 250683–250684, 250888, 250895–250896, 250898, 250900, 250937, 251031–251032, 251038, 251040–251043, 251045, 251048, 251050, 251053, 251055, 251057, 251060–251061, 251063, 251067, 254661, 254663, 254677, 254684, 254687, 254694, 254785, 254787, 254789–254792, 254795–254796, 254798–254799, 254804, 254807–254808, 254813–254814, 254819, 254821–254822, 254826, 254828 (CNCI).

###### Comments.

The specimens with the identifiers OSUC 244136 and 244139 (USNM) are paratypes of *Scelio howardi* but we believe actually are *Scelio zolotarevskyi*.

Nixon (1958) noted the similarities between *Scelio zolotarevskyi* and *Scelio sudanensis* but chose to maintain the latter as a valid species. Variation in this species is well documented by the hundreds of reared specimens (material at MNHN) now available. Based on this variation and review of the types (see images via http://hol.osu.edu/index.html?id=5364), we propose the current synonymy.

The flat ventral margin of the villus, and its general form are extremely constant, as are the colors of the scape and legs, and in combination with the particularly dense pilosity of the gena, one can instantly distinguish this species. Confusion is only possible with *Scelio pipilo* and *Scelio ululo*. Most African individuals of *Scelio zolotarevskyi* have the dorsomedial pilosity of the head and mesoscutum white, which is somewhat uncommon for Afrotropical *Scelio*. The trend to white contrasts with that observed in *Scelio howardi* in which the setae are nearly universally golden to light brown. Neither color pattern is unique, but may be found in either species. Many Asian specimens of *Scelio zolotarevskyi* have the setae of the dorsal head and mesonotum brown to golden brown. There is significant variation in the sculpture of T6 in females. Most individuals are predominantly longitudinally striate to strigose, with a tendency towards some reticulation posteriorly. A few of the largest individuals have completely reticulate rugulose sculpture, and a few of the smallest individuals are almost completely longitudinally sculptured.

In individuals with silver eyes there is often the appearance of very short setae between the ommatidia, though these perhaps do not extend above the surface of the eye. In individuals with black or mottled black and silver eyes these can not be discerned. Whether these are in fact setae or rather some refractory phenomenon should be tested with dissection and SEM. We consider *Scelio zolotarevskyi* to be polymorphic for these two states, and this was best observed in the long series of material from Yemen. The notauli in males are percurrent, though faintly so. This is useful in distinguishing them from the extremely similar males of *Scelio howardi*. See also comments for *Scelio ululo*.

*Scelio zolotarevskyi* is extremely widespread, being known throughout Africa and Asia. It has been reared from acridids of the subfamilies Eyprepocnemidinae, Cyrtacanthacridinae, and Oedipodinae, including the migratory locust (*Locusta migratoria*).

#### “brown” subgroup

##### Scelio balo

Valerio & Yoder sp. n.

http://zoobank.org/BA624755-30C0-4443-A50A-F215D0A21FBE

urn:lsid:biosci.ohio-state.edu:osuc_concepts:244970

http://species-id.net/wiki/Scelio_balo

**Figures 157–162.**
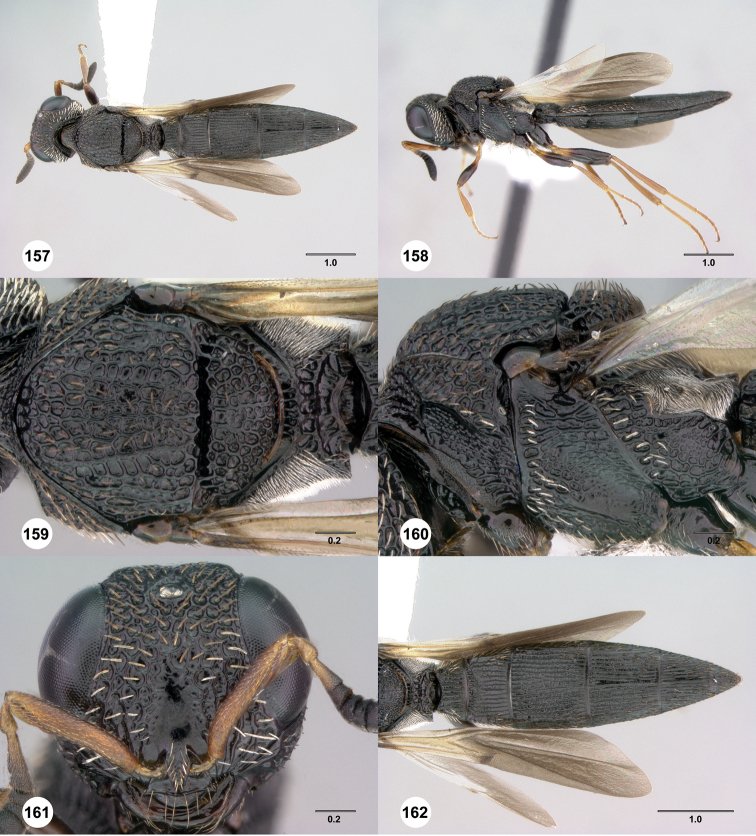
^140^ *Scelio balo* sp. n., holotype female (OSUC 212375). **157** Habitus, dorsal view **158** Habitus, lateral view **159** Mesosoma, dorsal view **160** Mesosoma, lateral view **161** Head, anterior view **162** Metasoma, dorsal view. Scale bars in millimeters.

Figures 157–162 ; Morphbank ^41^

###### Description.

Female body length: 6.86 mm (n=1). Color of pilosity of dorsal head in female: golden to brown. Occipital carina in female: percurrent. Color of pilosity of the frons below the anterior ocellus in female: predominantly golden to brown. Pilosity of eye in female: absent. Medial keel on interantennal process: absent. Width of lower gena in lateral view: wide, posterior margin of lower half of gena parallel to posterior orbit. Genal carina: absent. Color of genal pilosity: white. Color of scape in female: yellow throughout. Surface of the pronotal nucha in female: predominantly sculptured. Color of pilosity of pronotal shoulder in female: golden to dark brown, concolorous with that of mesoscutum. Sculpture of medial mesoscutum in female: predominantly angular reticulate to rugulose. Color of pilosity of mesoscutum in female: predominantly light brown to brown. Notaulus in female: indicated by a row of cells. Form of axillular carina in female: small, not particularly expanded or projected from the lateral edge of the mesoscutellum. Pilosity of propodeal nucha: absent. Pilosity of netrion: absent. Surface of mesopleural depression in female: sculptured throughout. Form of ventral margin of villus in female: bent ventrally in posterior, obviously not straight throughout. Color of coxae in female: brown. Color of hind femur: dark brown throughout. Color of hind tibia: yellow throughout. Fore wing length in female: apex not reaching anterior margin of T5. Color of metasoma: entirely dark brown. Sculpture of laterotergites in female: predominantly smooth. Pilosity of laterotergites in female: absent. Sculpture of medial T1 in female: most prominent elements predominantly longitudinal. Sculpture of medial T2 in female: most prominent elements predominantly longitudinal. Pattern of sculpture on T3–T5 in female: T3 predominantly reticulate, T4–T5 predominantly longitudinally striate to strigose. Color of pilosity on lateral T3–T5 in female: predominantly golden to brown. Lateral profile of T6 in female: more or less horizontal. Sculpture of T6 in female: predominantly rugulose to reticulate. Sculpture of lateral metasomal sternal bar in female: predominantly smooth to slightly irregularly rugose. Distribution of felt fields: 2 pairs present (S2, S3).

###### Diagnosis.

Similar to *Scelio fremo*, which also has a long metasoma and fore wings not reaching T5. Differing from *Scelio fremo* by the bent ventral margin of the villus (vs. straight, compare Figs 118 and 160), and the extent of the brown pilosity of the frons (vs. white, compare Figs 119 and 161). *Scelio balo* is darker overall than *Scelio fremo*, although this may be an artifact.

###### Etymology.

The epithet is used as a noun in apposition derived from the Latin word for roar, howl, grumble, or snort (things taxonomists do during a revision).

###### Link to distribution map.

http://hol.osu.edu/map-large.html?id=244970

###### Material examined.

*Holotype*, female: **DEMOCRATIC REPUBLIC OF THE CONGO**: Bandundu Prov., Wamba River, Kikongo Mission, 04°15'S, 17°10'E, 15.IV.2006, black light, S. L. Heydon & S. E. Stevenson, OSUC 212375 (deposited in CNCI).

###### Comments.

The metasoma of *Scelio balo* is near black and only very slightly lighter than the mesosoma. The fore wing is not as obviously shortened as in *Scelio fremo*. See also comments for *Scelio fremo*.

##### Scelio bubulo

Yoder sp. n.

http://zoobank.org/6EB0D77E-3F05-4EA9-BEEC-814E01E81DE4

urn:lsid:biosci.ohio-state.edu:osuc_concepts:244978

http://species-id.net/wiki/Scelio_bubulo

**Figures 163–168.**
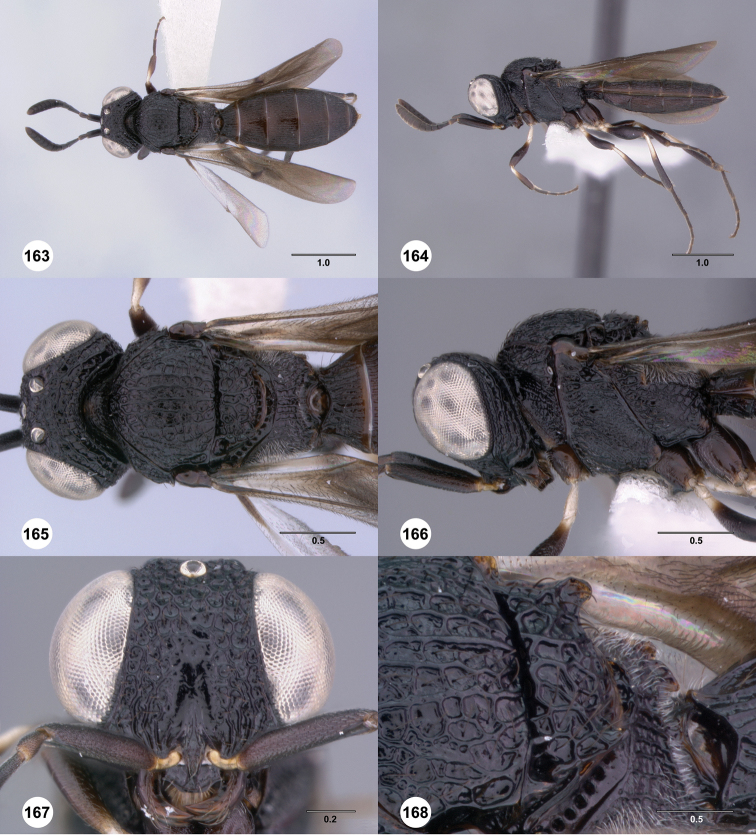
^141^ *Scelio bubulo* sp. n., holotype female (OSUC 213024). **163** Habitus, dorsal view **164** Habitus, lateral view **165** Head and mesosoma, dorsal view **166** Head and mesosoma, lateral view **167** Head, anterior view **168** Mesoscutellum, oblique dorsolateral view. Scale bars in millimeters.

Figures 163–168 ; Morphbank ^42^

###### Description.

Female body length: 4.24–4.87 mm (n=7). Color of pilosity of dorsal head in female: golden to brown. Occipital carina in female: percurrent. Color of pilosity of the frons below the anterior ocellus in female: predominantly golden to brown. Pilosity of eye in female: absent; present. Medial keel on interantennal process: absent. Width of lower gena in lateral view: narrowing dorsally, posterior margin of lower half of gena angled with respect to posterior orbit. Genal carina: present. Color of genal pilosity: brown. Color of scape in female: brown to dark brown throughout. Surface of the pronotal nucha in female: predominantly sculptured; predominantly smooth. Color of pilosity of pronotal shoulder in female: golden to dark brown, concolorous with that of mesoscutum. Sculpture of medial mesoscutum in female: predominantly angular reticulate to rugulose. Color of pilosity of mesoscutum in female: predominantly light brown to brown. Notaulus in female: not delimited. Form of axillular carina in female: lobelike in posterolateral corner. Pilosity of propodeal nucha: present. Pilosity of netrion: present. Surface of mesopleural depression in female: sculptured throughout. Form of ventral margin of villus in female: very slightly concave, almost straight. Color of coxae in female: brown. Color of hind femur: dark brown with white base. Color of hind tibia: white basally, dark brown apically. fore wing length in female: apex surpassing posterior margin of T6. Color of metasoma: entirely dark brown. Sculpture of laterotergites in female: predominantly smooth. Pilosity of laterotergites in female: absent. Sculpture of medial T1 in female: most prominent elements predominantly longitudinal. Sculpture of medial T2 in female: most prominent elements predominantly longitudinal. Pattern of sculpture on T3–T5 in female: predominantly longitudinally striate. Color of pilosity on lateral T3–T5 in female: predominantly golden to brown. Lateral profile of T6 in female: vertically sloped in posterior half. Sculpture of T6 in female: predominantly rugulose to reticulate. Sculpture of lateral metasomal sternal bar in female: minutely reticulate throughout. Distribution of felt fields: 2 pairs present (S2, S3).

###### Diagnosis.

Similar to other species of the brown subgroup that have the lateral node of the mesoscutellum developed into a flange or node. Differing from all these species by presence of setae on the netrion and the degree of development and form of the lateral node of the axillular carina which is large, round, lobelike vs. small, sharp and bladelike.

###### Etymology.

The epithet is used as a noun in apposition derived from the Latin word for hoot like an owl.

###### Link to distribution map.

http://hol.osu.edu/map-large.html?id=244978

###### Material examined.

*Holotype*, female: **CAMEROON**: Nkoemvom, 24.VIII.1983, malaise trap, D. Jackson, OSUC 213024 (deposited in BMNH). *Paratypes*: (6 females) **GHANA**: 1 female, OSUC 212504 (CNCI). **GUINEA**: 3 females, OSUC 213099, 213101 (CNCI); OSUC 213102 (OSUC). **SIERRA LEONE**: 1 female, OSUC 244054 (MZLU). **UGANDA**: 1 female, OSUC 212348 (CNCI).

###### Comments.

The presence of pilosity on the propodeal nucha and netrion may not be independent as both states are also found together in *Scelio philippinensis*, though the two species do not appear to be closely related. The frons between the eyes is notably narrow, as are the mandibles (Fig. 167). The fore wing in females appears to extend just past the apex of the metasoma.

##### Scelio cano

Yoder sp. n.

http://zoobank.org/259A3DFF-EE10-4FF0-87E6-38B82AA05636

urn:lsid:biosci.ohio-state.edu:osuc_concepts:244979

http://species-id.net/wiki/Scelio_cano

**Figures 169–174.**
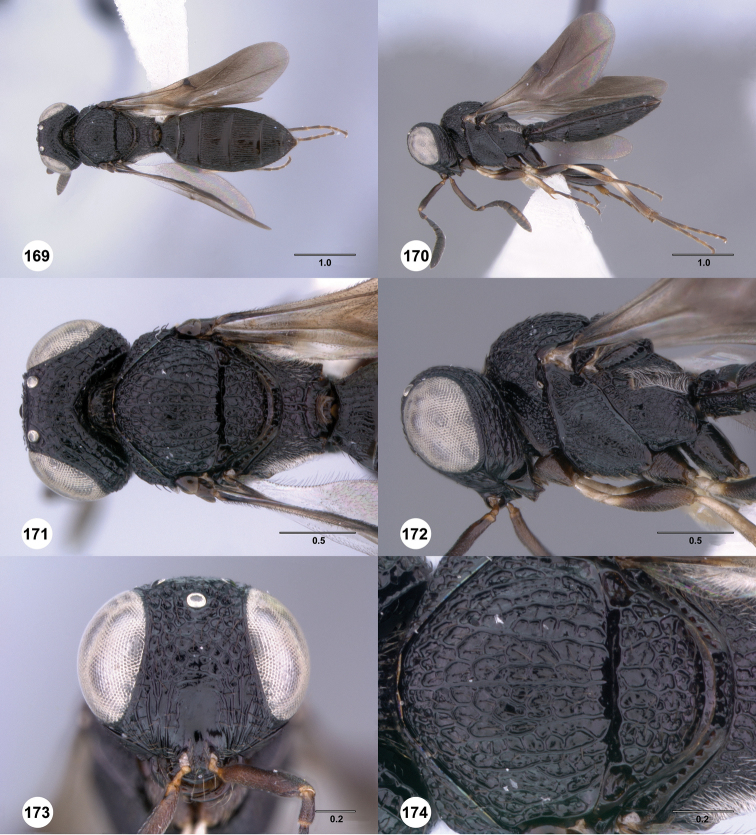
^142^ *Scelio cano* sp. n., holotype female (OSUC 212930). **169** Habitus, dorsal view **170** Habitus, lateral view **171** Head and mesosoma, dorsal view **172** Head and mesosoma, lateral view **173** Head, anterior view **174** Mesoscutum, dorsal view. Scale bars in millimeters.

Figures 169–174 ; Morphbank ^43^

###### Description.

Female body length: 4.16–4.60 mm (n=5). Male body length: 4.17 mm (n=1). Color of pilosity of dorsal head in female: golden to brown. Occipital carina in female: percurrent. Color of pilosity of the frons below the anterior ocellus in female: predominantly golden to brown. Pilosity of eye in female: absent. Medial keel on interantennal process: present. Width of lower gena in lateral view: narrowing dorsally, posterior margin of lower half of gena angled with respect to posterior orbit. Genal carina: absent. Color of genal pilosity: brown. Color of scape in female: brown to dark brown throughout. Surface of the pronotal nucha in female: predominantly sculptured. Color of pilosity of pronotal shoulder in female: golden to dark brown, concolorous with that of mesoscutum. Sculpture of medial mesoscutum in female: predominantly angular reticulate to rugulose. Color of pilosity of mesoscutum in female: predominantly light brown to brown. Notaulus in female: not delimited. Notaulus in male: not delimited. Form of axillular carina in female: small, not particularly expanded or projected from the lateral edge of the mesoscutellum. Pilosity of propodeal nucha: absent. Pilosity of netrion: absent. Surface of mesopleural depression in female: with small smooth patch ventrally. Form of ventral margin of villus in female: bent ventrally in posterior, obviously not straight throughout. Color of coxae in female: brown. Color of hind femur: dark brown throughout. Color of hind tibia: white basally, dark brown apically. Fore wing length in female: apex between anterior margin of T5 and posterior margin of T6; apex surpassing posterior margin of T6. Color of metasoma: entirely dark brown. Sculpture of laterotergites in female: predominantly aciculate. Pilosity of laterotergites in female: absent. Sculpture of medial T1 in female: most prominent elements predominantly reticulate rugulose. Sculpture of medial T2 in female: predominantly smooth or obliterated. Pattern of sculpture on T3–T5 in female: T3 predominantly reticulate, T4–T5 predominantly longitudinally striate to strigose; predominantly longitudinally striate. Color of pilosity on lateral T3–T5 in female: predominantly golden to brown. Lateral profile of T6 in female: vertically sloped in posterior half. Sculpture of T6 in female: predominantly rugulose to reticulate. Sculpture of lateral metasomal sternal bar in female: minutely reticulate throughout. Distribution of felt fields: 2 pairs present (S2, S3).

###### Diagnosis.

Most similar to *Scelio tono*, also a brown subgroup species that shares a similarly colored tibia (as in Fig. 212). Differing from *Scelio tono* by the absence of notauli (a clear channel in posterior half of mesoscutum present in *Scelio tono*) and the less well-developed axillular carina that does not extend above the dorsal surface of the mesoscutum. Similar also to *Scelio gemo*, but may be differentiated by the brown scape and sloping T6 (vs. yellow to brown and horizontally oriented respectively).

###### Etymology.

The epithet is used as a noun in apposition derived from the Latin word for sing.

###### Link to distribution map.

http://hol.osu.edu/map-large.html?id=244979

###### Material examined.

*Holotype*, female: **UGANDA**: Kabarole Dist., 20km SE Fort Portal, nr. Mikana, stream, Makerere University Biological Field Station (MUBFS), 00°34.37'N, 30°21.66'E, 1530m, 7.X–21.X.2001, yellow pan trap/flight intercept trap, B. Gill & J. Gill, OSUC 212930 (deposited in CNCI). *Paratypes*: **UGANDA**: 4 females, 1 male, OSUC 212346, 212349–212350, 250819 (CNCI); OSUC 212347 (OSUC).

###### Comments.

In males and females the inner course of the notauli is weakly discernible but the outer course is not. For this reason we have coded the notauli as absent. There is a small irregular patch of smoother obliterated sculpture on the ventral mesopleural depression. The sculpture of the medial metasoma is somewhat more obliterated and irregular than typical. Specimens of this species were taken together in identical collecting events with *Scelio gemo*.

##### Scelio crepo

Yoder sp. n.

http://zoobank.org/235A51AE-639B-4103-A2D9-81E81CDBBCC8

urn:lsid:biosci.ohio-state.edu:osuc_concepts:244980

http://species-id.net/wiki/Scelio_crepo

**Figures 175–180.**
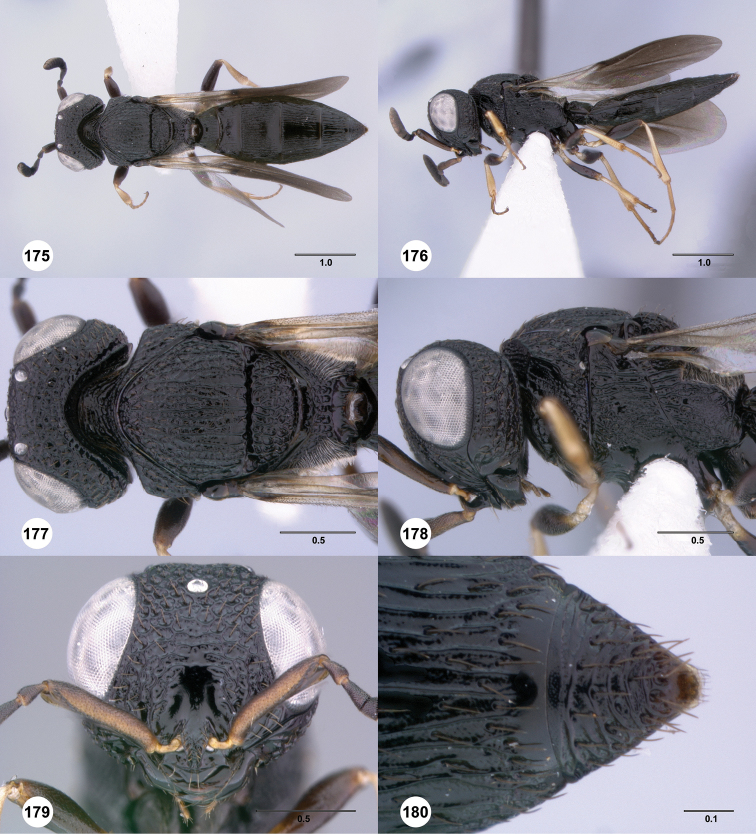
^143^ *Scelio crepo* sp. n., holotype female (OSUC 212511). **175** Habitus, dorsal view **176** Habitus, lateral view **177** Head and mesosoma, dorsal view **178** Head and mesosoma, lateral view **179** Head, anterior view **180** Metasomal T6, dorsal view. Scale bars in millimeters.

Figures 175–180 ; Morphbank ^44^

###### Description.

Female body length: 4.36–5.26 mm (n=2). Color of pilosity of dorsal head in female: golden to brown. Occipital carina in female: percurrent. Color of pilosity of the frons below the anterior ocellus in female: predominantly golden to brown. Pilosity of eye in female: absent. Medial keel on interantennal process: absent. Width of lower gena in lateral view: wide, posterior margin of lower half of gena parallel to posterior orbit. Genal carina: absent. Color of genal pilosity: brown. Color of scape in female: brown to dark brown throughout. Surface of the pronotal nucha in female: predominantly sculptured. Color of pilosity of pronotal shoulder in female: white to light brown, lighter than that of mesoscutum. Sculpture of medial mesoscutum in female: predominantly longitudinally strigose to rugulose. Color of pilosity of mesoscutum in female: predominantly light brown to brown. Notaulus in female: present as more or less uninterrupted channel in posterior 1/2 of mesoscutum. Form of axillular carina in female: small, not particularly expanded or projected from the lateral edge of the mesoscutellum. Pilosity of propodeal nucha: absent. Pilosity of netrion: absent. Surface of mesopleural depression in female: sculptured throughout. Form of ventral margin of villus in female: bent ventrally in posterior, obviously not straight throughout. Color of coxae in female: brown. Color of hind femur: dark brown throughout. Color of hind tibia: yellow throughout. Fore wing length in female: apex between anterior margin of T5 and posterior margin of T6. Color of metasoma: light reddish brown. Sculpture of laterotergites in female: predominantly smooth. Pilosity of laterotergites in female: absent. Sculpture of medial T1 in female: most prominent elements predominantly longitudinal. Sculpture of medial T2 in female: most prominent elements predominantly longitudinal. Pattern of sculpture on T3–T5 in female: predominantly longitudinally striate. Color of pilosity on lateral T3–T5 in female: predominantly golden to brown. Lateral profile of T6 in female: more or less horizontal. Sculpture of T6 in female: predominantly transversely rugose. Sculpture of lateral metasomal sternal bar in female: predominantly smooth to slightly irregularly rugose. Distribution of felt fields: 2 pairs present (S2, S3).

###### Diagnosis.

Differing from other brown subgroup species by the combination of the completely yellow tibia (Fig. 176) and the transverse rugae of T6 (Fig. 180, more reticulate in most others).

###### Etymology.

The epithet is used as a noun in apposition derived from the Latin word for to rattle, clatter, crackle.

###### Link to distribution map.

http://hol.osu.edu/map-large.html?id=244980

###### Material examined.

*Holotype*, female: **TANZANIA**: Tanga Reg., hills, Amani, 23.VI–24.VII.2001, D. Quicke, OSUC 212511 (deposited in CNCI). *Paratype*: **TANZANIA**: 1 female, OSUC 212514 (CNCI).

###### Comments.

The sculpture of the mesoscutum is somewhat finer (Fig. 177) than seen in other closely related species. There is some aciculate sculpture on the laterotergites, but they are predominantly smooth. There is a hint of a third felt field pair on S4, but we have coded only two pairs as present.

##### Scelio gemo

Yoder sp. n.

http://zoobank.org/D32913DF-A349-4568-9EC8-58ED077919CF

urn:lsid:biosci.ohio-state.edu:osuc_concepts:244981

http://species-id.net/wiki/Scelio_gemo

**Figures 181–186.**
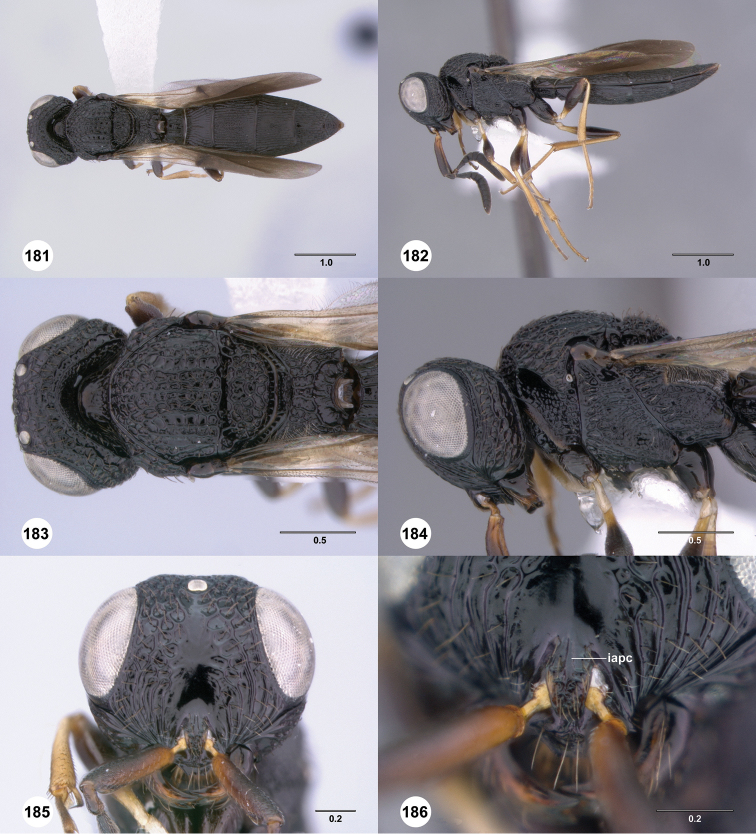
^144^ *Scelio gemo* sp. n., holotype female (OSUC 212932). **181** Habitus, dorsal view **182** Habitus, lateral view **183** Head and mesosoma, dorsal view **184** Head and mesosoma, lateral view **185** Head, anterior view **186** Head, interantennal process, oblique anterodorsal view. *iapc*, interantennal process carina. Scale bars in millimeters.

Figures 19 , 181–186 ; Morphbank ^45^

###### Description.

Female body length: 5.37–5.77 mm (n=5). Male body length: 4.80 mm (n=1). Color of pilosity of dorsal head in female: golden to brown. Occipital carina in female: percurrent. Color of pilosity of the frons below the anterior ocellus in female: predominantly golden to brown. Pilosity of eye in female: absent; present. Medial keel on interantennal process: present. Width of lower gena in lateral view: wide, posterior margin of lower half of gena parallel to posterior orbit. Genal carina: present. Color of genal pilosity: brown. Color of scape in female: yellow in basal half, darkening to light brown in apical half. Surface of the pronotal nucha in female: predominantly sculptured; predominantly smooth. Color of pilosity of pronotal shoulder in female: golden to dark brown, concolorous with that of mesoscutum. Sculpture of medial mesoscutum in female: predominantly angular reticulate to rugulose. Color of pilosity of mesoscutum in female: predominantly light brown to brown. Notaulus in female: indicated by a row of cells. Notaulus in male: delimited by row of cells. Form of axillular carina in female: small, not particularly expanded or projected from the lateral edge of the mesoscutellum. Pilosity of propodeal nucha: absent. Pilosity of netrion: absent. Surface of mesopleural depression in female: sculptured throughout. Form of ventral margin of villus in female: bent ventrally in posterior, obviously not straight throughout. Color of coxae in female: brown. Color of hind femur: yellow throughout. Color of hind tibia: yellow at extreme base, otherwise light brown. Fore wing length in female: apex between anterior margin of T5 and posterior margin of T6. Color of metasoma: entirely black. Sculpture of laterotergites in female: predominantly smooth. Pilosity of laterotergites in female: absent. Sculpture of medial T1 in female: most prominent elements predominantly longitudinal. Sculpture of medial T2 in female: most prominent elements predominantly longitudinal. Pattern of sculpture on T3–T5 in female: T3 predominantly reticulate, T4–T5 predominantly longitudinally striate to strigose. Color of pilosity on lateral T3–T5 in female: predominantly golden to brown. Lateral profile of T6 in female: more or less horizontal. Sculpture of T6 in female: predominantly transversely rugose. Sculpture of lateral metasomal sternal bar in female: minutely reticulate throughout. Distribution of felt fields: 2 pairs present (S2, S3).

###### Diagnosis.

Similar to other species of the brown subgroup, particularly *Scelio cano*. Differing from these by the combination of the presence of a medial ridge of interantennal process, the notauli indicated by a row of cells, the yellow hind tibia, the reticulate sculpture of the mesoscutum and the nearly horizontal T6 (as seen in lateral view).

###### Etymology.

The epithet is used as a noun in apposition derived from the Latin word for to groan, moan, sigh.

###### Link to distribution map.

http://hol.osu.edu/map-large.html?id=244981

###### Material examined.

*Holotype*, female: **UGANDA**: Kabarole Dist., 20km SE Fort Portal, Makerere University Biological Field Station (MUBFS), 0°33.08'N, 30°21.54'E, 1570m, 9.X–11.X.2001, yellow pan trap, B. Gill & J. Gill, OSUC 212932 (deposited in CNCI). *Paratypes*:(4 females, 1 male) **CENTRAL AFRICAN REPUBLIC**: 1 male, OSUC 250982 (SAMC). **UGANDA**: 4 females, OSUC 212345, 212933 (CNCI); OSUC 250749, 250751 (SAMC).

###### Comments.

See *Scelio cano*.

##### Scelio grunnio

Yoder sp. n.

http://zoobank.org/5BEEF810-51C3-41BC-B67B-3EBBD1598893

urn:lsid:biosci.ohio-state.edu:osuc_concepts:244986

http://species-id.net/wiki/Scelio_grunnio

**Figures 187–192.**
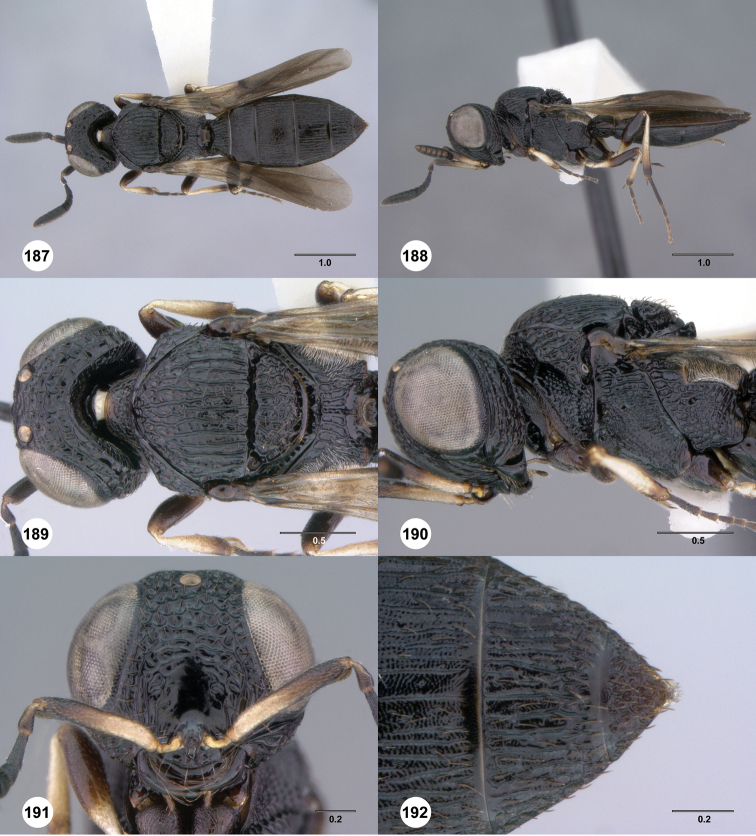
^145^ *Scelio grunnio* sp. n., holotype female (OSUC 213098). **187** Habitus, dorsal view **188** Habitus, lateral view **189** Head and mesosoma, dorsal view **190** Head and mesosoma, lateral view **191** Head, anterior view **192** Apex of metasoma, dorsal view. Scale bars in millimeters.

Figures 30 , 187–192 ; Morphbank ^46^

###### Description.

Female body length: 4.69–5.16 mm (n=6). Male body length: 4.78 mm (n=1). Color of pilosity of dorsal head in female: golden to brown. Occipital carina in female: percurrent. Color of pilosity of the frons below the anterior ocellus in female: predominantly golden to brown. Pilosity of eye in female: absent; present. Medial keel on interantennal process: absent. Width of lower gena in lateral view: narrowing dorsally, posterior margin of lower half of gena angled with respect to posterior orbit. Genal carina: absent. Color of genal pilosity: brown. Color of scape in female: yellow in basal half, darkening to light brown in apical half. Surface of the pronotal nucha in female: predominantly sculptured. Color of pilosity of pronotal shoulder in female: golden to dark brown, concolorous with that of mesoscutum. Sculpture of medial mesoscutum in female: predominantly longitudinally strigose to rugulose. Color of pilosity of mesoscutum in female: predominantly light brown to brown. Notaulus in female: present as more or less uninterrupted channel in posterior 1/2 of mesoscutum. Form of axillular carina in female: small, not particularly expanded or projected from the lateral edge of the mesoscutellum; bladelike or carinate in posterolateral corner but not forming distinct lobe. Pilosity of propodeal nucha: absent. Pilosity of netrion: absent. Surface of mesopleural depression in female: sculptured throughout; with small smooth patch ventrally. Form of ventral margin of villus in female: bent ventrally in posterior, obviously not straight throughout. Color of coxae in female: brown. Color of hind femur: dark brown with white base. Color of hind tibia: yellow at extreme base, otherwise light brown. Fore wing length in female: apex between anterior margin of T5 and posterior margin of T6. Color of metasoma: light reddish brown. Sculpture of laterotergites in female: predominantly smooth; predominantly aciculate. Pilosity of laterotergites in female: absent. Sculpture of medial T1 in female: most prominent elements predominantly longitudinal. Sculpture of medial T2 in female: most prominent elements predominantly longitudinal. Pattern of sculpture on T3–T5 in female: T3 predominantly reticulate, T4–T5 predominantly longitudinally striate to strigose; predominantly longitudinally striate. Color of pilosity on lateral T3–T5 in female: predominantly golden to brown. Lateral profile of T6 in female: vertically sloped in posterior half; more or less horizontal. Sculpture of T6 in female: predominantly rugulose to reticulate. Sculpture of lateral metasomal sternal bar in female: predominantly smooth to slightly irregularly rugose. Distribution of felt fields: 2 pairs present (S2, S3).

###### Diagnosis.

Most similar to *Scelio susurro*, *Scelio cano*, *Scelio crepo*, and *Scelio tono* which have the combination of brown pilosity on the pronotal shoulders, evenly distributed pilosity of the metasoma and the absence of a carina within the interantennal process. Differing from these species by the combination of the color pattern of the hind tibia (white with brown band at apex, or yellow throughout), color pattern of the scape (yellow at base and brown at apex), and sculpture of the mesoscutum (with longitudinal trend).

###### Etymology.

The epithet is used as a noun in apposition derived from the Latin word for grunt.

###### Link to distribution map.

http://hol.osu.edu/map-large.html?id=244986

###### Material examined.

*Holotype*, female: **GUINEA**: Lola Pref., rainforest, Mount Nimba, 07°41–42'N, 08°23'W, 514–740m, XII-1990–III-1991, flight intercept trap, L. Leblanc, OSUC 213098 (deposited in CNCI). *Paratypes*: (5 females, 1 male) **DEMOCRATIC REPUBLIC OF THE CONGO**: 2 females, OSUC 212376–212377 (CNCI). **GHANA**: 2 females, 1 male, OSUC 214366–214367, 250741 (OSUC). **GUINEA**: 1 female, OSUC 213100 (CNCI).

###### Comments.

As presently delimited this species is relatively highly variable. The known material consists of three sets collected in different localities. Specimens within each set are morphologically variable, and there is noticeable variation between the sets as well. Each set may ultimately represent different species, but more specimens are needed to circumscribe the observed variation. There is a tendency among all individuals to have thicker, longitudinal carinae on the mesonotum and propodeum, usually with relatively uninterrupted interstices. In the largest specimens, however, there is more reticulation between the longitudinal elements. In all but the two specimens from the D.R.C. (OSUC 212376, 212377) the tibia is yellow with a short brown band at the apex. The two specimens from the D.R.C. appear somewhat translucent, and this may indicate modification of the natural color by collecting method. The sculpture of T6 may ultimately be of use in delimiting species, but there appears to be a transition from smaller individuals with thinner somewhat longitudinal carinae to larger individuals with thicker reticulations. The sculpture of the lateral portion of T1 also varies: in the two individuals from Ghana the lateral margin is smooth and with only a single longitudinal carina; additional rugulae are present in the other four individuals. The largest individual (OSUC 212377) is perhaps the best candidate for a separate species. In this specimen the mid tibia is nearly completely yellow (as compared to the brown to yellow pattern seen in other individuals), the sculpture of the metasoma is more robust than the other individuals, there is no glabrous patch on the ventral metasoma, and the mesoscutum and propodeum have more reticulations partially obscuring the longitudinal trend in sculpture seen in the other individuals. The sculpture of the frons in the two individuals from Ghana is more transverse than the more uniform reticulate sculpture seen in the other four.

While variation exists all six specimens may be firmly grouped by the characters listed in the diagnosis and others including the relatively large ocelli and eyes (resulting in a narrow frons), the similar sculpture pattern of the frons, the color pattern on the scape, the slightly developed axilluar carinae, and the ventromedially bent villus.

##### Scelio latro

Yoder sp. n.

http://zoobank.org/F1F5D68A-AFC1-4D2E-ACF6-6A07962F5130

urn:lsid:biosci.ohio-state.edu:osuc_concepts:244982

http://species-id.net/wiki/Scelio_latro

Figures 21 , 26 , 193–198 ; Morphbank ^47^

###### Description.

Female body length: 3.25–4.40 mm (n=9). Color of pilosity of dorsal head in female: golden to brown. Occipital carina in female: broadly obliterated to absent medially. Color of pilosity of the frons below the anterior ocellus in female: predominantly white. Pilosity of eye in female: absent. Medial keel on interantennal process: absent. Width of lower gena in lateral view: wide, posterior margin of lower half of gena parallel to posterior orbit. Genal carina: absent. Color of genal pilosity: white. Color of scape in female: yellow throughout. Surface of the pronotal nucha in female: predominantly sculptured; predominantly smooth. Color of pilosity of pronotal shoulder in female: white to light brown, lighter than that of mesoscutum; golden to dark brown, concolorous with that of mesoscutum. Sculpture of medial mesoscutum in female: predominantly angular reticulate to rugulose. Color of pilosity of mesoscutum in female: predominantly light brown to brown. Notaulus in female: indicated by a row of cells. Form of axillular carina in female: small, not particularly expanded or projected from the lateral edge of the mesoscutellum. Pilosity of propodeal nucha: absent. Pilosity of netrion: absent. Surface of mesopleural depression in female: sculptured throughout. Form of ventral margin of villus in female: bent ventrally in posterior, obviously not straight throughout. Color of coxae in female: brown. Color of hind femur: dark brown throughout. Color of hind tibia: yellow throughout. Fore wing length in female: apex between anterior margin of T5 and posterior margin of T6. Color of metasoma: entirely dark brown. Sculpture of laterotergites in female: predominantly aciculate. Pilosity of laterotergites in female: absent. Sculpture of medial T1 in female: most prominent elements predominantly longitudinal. Sculpture of medial T2 in female: most prominent elements predominantly longitudinal. Pattern of sculpture on T3–T5 in female: predominantly longitudinally striate. Color of pilosity on lateral T3–T5 in female: predominantly golden to brown. Lateral profile of T6 in female: vertically sloped in posterior half; more or less horizontal. Sculpture of T6 in female: predominantly longitudinally striate to strigose. Sculpture of lateral metasomal sternal bar in female: minutely reticulate throughout. Distribution of felt fields: 2 pairs present (S2, S3).

###### Diagnosis.

**Figures 193–198.**
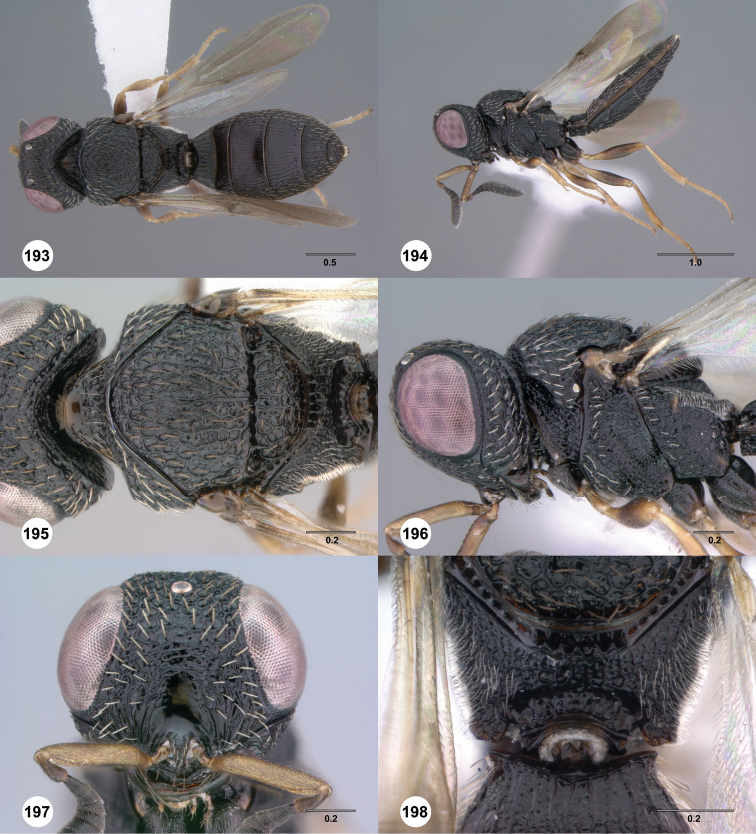
^146^ *Scelio latro* sp. n. **193, 194, 196, 198** holotype female (OSUC 250953) **195, 197** paratype female (OSUC 212968). **193** Habitus, dorsal view **194** Habitus, lateral view **195** Mesosoma, dorsal view **196** Head and mesosoma, lateral view **197** Head, anterior view **198** Propodeum and base of metasoma, dorsal view. Scale bars in millimeters.

Most similar to *Scelio mutio* which shares a similarly shaped villus (Fig. 21), pilosity and sculpture of the mesoscutum (Fig. 195), and general habitus (Figs 193, 194). *Scelio latro* differs from *Scelio mutio* in the fine longitudinal sculpture of T6 (vs. transversely rugulose), the aciculate sculpture of the laterotergites (vs. smooth), the pilosity of the pronotal shoulder which is lighter than that of the mesoscutum (vs. concolorous), the light brown hind femur (vs. yellow), and, subtly, the incomplete occipital carina (vs. percurrent).

###### Etymology.

The epithet is used as a noun in apposition derived from the Latin word for bark or howl.

###### Link to distribution map.

http://hol.osu.edu/map-large.html?id=244982

###### Material examined.

*Holotype*, female: **TANZANIA**: Dar es Salaam Reg., Dar es Salaam, II-1985, flight intercept trap, J. Middleton, OSUC 250953 (deposited in CNCI). *Paratypes*: (8 females) **MALAWI**: 2 females, OSUC 212453, 212929 (CNCI). **TANZANIA**: 6 females, OSUC 212968, 212976, 250951, 250959, 250968 (CNCI); OSUC 212969 (OSUC).

###### Comments.

The differences between *Scelio latro* and *Scelio mutio* are subtle but consistent. The color of pilosity on the head and pronotal shoulders is intermediate between white and the brown which may lead to some confusion in the key if it is interpreted as white. Though subtle, the color of the pilosity of the frons is not white. The propodeal projections (Fig. 198) are somewhat rounded and blunt. The preapical margin of T6 is relatively broad and well developed. The longitudinal sculpture of the propodeum is generally angled outwards. The ventral margin of the villus curves upwards forming an acute angle just past the anterior origin. In other species its course extends below the ventral extent of the propodeal spiracle forming a right or obtuse angle.

##### Scelio mutio

Yoder sp. n.

http://zoobank.org/153F4575-ACAA-49DC-BEA0-689A0C99B1D4

urn:lsid:biosci.ohio-state.edu:osuc_concepts:244983

http://species-id.net/wiki/Scelio_mutio

**Figures 199–204.**
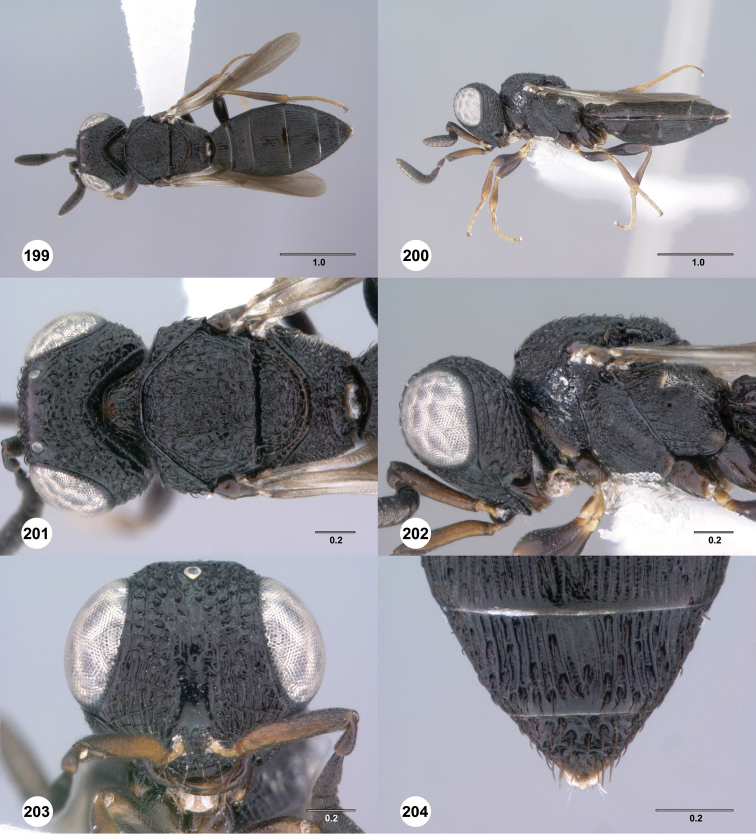
^147^ *Scelio mutio* sp. n., holotype female (OSUC 244035). **199** Habitus, dorsal view **200** Habitus, lateral view **201** Head and mesosoma, dorsal view **202** Head and mesosoma, lateral view **203** Head, anterior view **204** Apex of metasoma, dorsal view. Scale bars in millimeters.

Figures 199–204 ; Morphbank ^48^

###### Description.

Female body length: 3.20–3.93 mm (n=6). Color of pilosity of dorsal head in female: golden to brown. Occipital carina in female: percurrent. Color of pilosity of the frons below the anterior ocellus in female: predominantly golden to brown. Pilosity of eye in female: present. Medial keel on interantennal process: absent. Width of lower gena in lateral view: wide, posterior margin of lower half of gena parallel to posterior orbit. Genal carina: absent. Color of genal pilosity: brown. Color of scape in female: yellow in basal half, darkening to light brown in apical half. Surface of the pronotal nucha in female: predominantly sculptured. Color of pilosity of pronotal shoulder in female: golden to dark brown, concolorous with that of mesoscutum. Sculpture of medial mesoscutum in female: predominantly angular reticulate to rugulose. Color of pilosity of mesoscutum in female: predominantly light brown to brown. Notaulus in female: not delimited. Form of axillular carina in female: small, not particularly expanded or projected from the lateral edge of the mesoscutellum. Pilosity of propodeal nucha: absent. Pilosity of netrion: absent. Surface of mesopleural depression in female: sculptured throughout. Form of ventral margin of villus in female: bent ventrally in posterior, obviously not straight throughout. Color of coxae in female: brown. Color of hind femur: dark brown throughout. Color of hind tibia: yellow at extreme base, otherwise light brown. Fore wing length in female: apex between anterior margin of T5 and posterior margin of T6. Color of metasoma: entirely dark brown. Sculpture of laterotergites in female: predominantly smooth. Pilosity of laterotergites in female: absent. Sculpture of medial T1 in female: most prominent elements predominantly longitudinal. Sculpture of medial T2 in female: most prominent elements predominantly longitudinal. Pattern of sculpture on T3–T5 in female: predominantly longitudinally striate. Color of pilosity on lateral T3–T5 in female: predominantly golden to brown. Lateral profile of T6 in female: vertically sloped in posterior half. Sculpture of T6 in female: predominantly rugulose to reticulate; predominantly transversely rugose. Sculpture of lateral metasomal sternal bar in female: minutely reticulate throughout. Distribution of felt fields: 2 pairs present (S2, S3).

###### Diagnosis.

Similar to other species in the brown subgroup. Differing from these by the combination of scape color (yellow or light brown at base, darkening to brown at apex), hind femur color (light brown nearly throughout), and sculpture of the lateral margin of the metasomal sternites. See also diagnosis for *Scelio latro*.

###### Etymology.

The epithet is used as a noun in apposition derived from the Latin word for mutter.

###### Link to distribution map.

http://hol.osu.edu/map-large.html?id=244983

###### Material examined.

*Holotype*, female: **SOUTH AFRICA**: Eastern Cape Prov., Blue Duiker Trail, ~2.5km W Storms River Mouth, Tsitsikamma Section, tall coastal forest, FMHD#2004-016 / ANMT site 1079, Garden Route National Park, 34°01.05'S, 23°52.66'E, 100m, 29.I–25.II.2004, flight intercept trap, Solodovnikov, Newton & Thayer, OSUC 244035 (deposited in FMNH). *Paratypes*: **SOUTH AFRICA**: 5 females, OSUC 211353 (CNCI); OSUC 244036–244038 (FMNH); OSUC 244039 (OSUC).

###### Comments.

*Scelio mutio*, the southernmost member of the brown subgroup, is also the smallest member of the group. T6 is only slightly wider than long and is relatively more conical than similar species. The metasoma in dorsal view is somewhat pointed and ovoid. The pronotal nucha is smooth in the anterior half, and sculptured posteriorly.

##### Scelio susurro

Yoder sp. n.

http://zoobank.org/29960D20-85C8-46D3-BE13-07F6CD43260E

urn:lsid:biosci.ohio-state.edu:osuc_concepts:244985

http://species-id.net/wiki/Scelio_susurro

**Figures 205–210.**
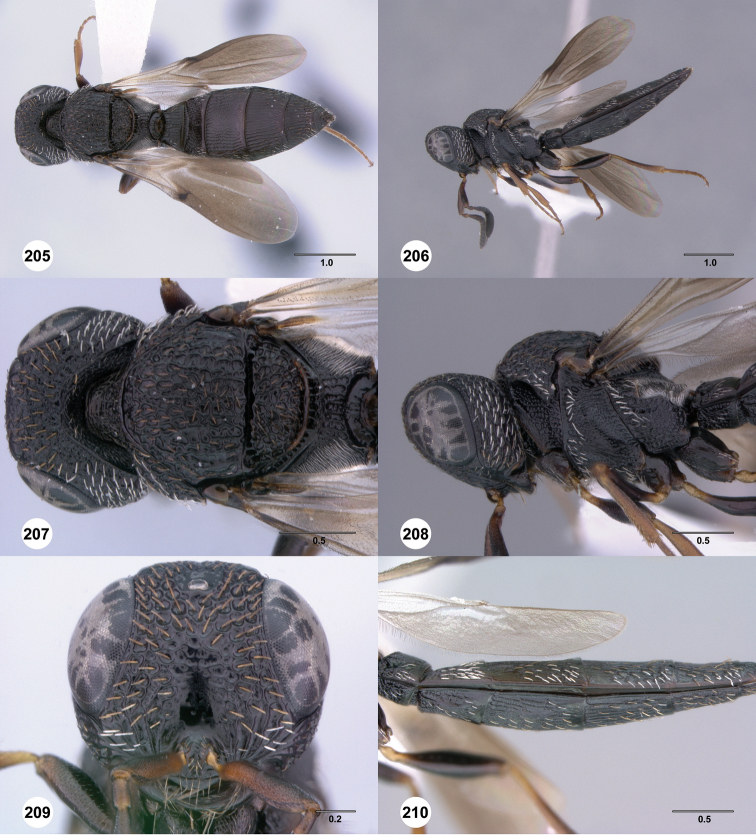
^148^ *Scelio susurro* sp. n., holotype female (OSUC 213654). **205** Habitus, dorsal view **206** Habitus, lateral view **207** Head and mesosoma, dorsal view **208** Head and mesosoma, lateral view **209** Head, anterior view **210**, Metasoma, lateral view. Scale bars in millimeters.

Figures 28 , 205–210 ; Morphbank ^49^

###### Description.

Female body length: 4.90–5.88 mm (n=2). Male body length: 4.41–4.80 mm (n=15). Color of pilosity of dorsal head in female: golden to brown. Occipital carina in female: percurrent. Color of pilosity of the frons below the anterior ocellus in female: predominantly golden to brown. Pilosity of eye in female: absent. Medial keel on interantennal process: absent. Width of lower gena in lateral view: wide, posterior margin of lower half of gena parallel to posterior orbit. Genal carina: absent. Color of genal pilosity: white. Color of scape in female: brown to dark brown throughout; yellow in basal half, darkening to light brown in apical half. Surface of the pronotal nucha in female: predominantly sculptured. Color of pilosity of pronotal shoulder in female: white to light brown, lighter than that of mesoscutum; golden to dark brown, concolorous with that of mesoscutum. Sculpture of medial mesoscutum in female: predominantly angular reticulate to rugulose. Color of pilosity of mesoscutum in female: predominantly light brown to brown. Notaulus in female: not delimited; present as more or less uninterrupted channel in posterior 1/2 of mesoscutum. Notaulus in male: delimited by row of cells. Form of axillular carina in female: small, not particularly expanded or projected from the lateral edge of the mesoscutellum. Pilosity of propodeal nucha: absent. Pilosity of netrion: absent. Surface of mesopleural depression in female: with small smooth patch ventrally. Form of ventral margin of villus in female: bent ventrally in posterior, obviously not straight throughout. Color of coxae in female: brown. Color of hind femur: dark brown throughout. Color of hind tibia: yellow at extreme base, otherwise light brown. Fore wing length in female: apex between anterior margin of T5 and posterior margin of T6. Color of metasoma: entirely dark brown. Sculpture of laterotergites in female: predominantly smooth. Pilosity of laterotergites in female: absent. Sculpture of medial T1 in female: most prominent elements predominantly longitudinal. Sculpture of medial T2 in female: most prominent elements predominantly reticulate rugulose; most prominent elements predominantly longitudinal. Pattern of sculpture on T3–T5 in female: T3 predominantly reticulate, T4–T5 predominantly longitudinally striate to strigose. Color of pilosity on lateral T3–T5 in female: predominantly white. Lateral profile of T6 in female: more or less horizontal. Sculpture of T6 in female: predominantly rugulose to reticulate. Sculpture of lateral metasomal sternal bar in female: predominantly smooth to slightly irregularly rugose; minutely reticulate throughout. Distribution of felt fields: 2 pairs present (S2, S3).

###### Diagnosis.

Most similar to *Scelio fremo* which shares similar setal patterns on the gena, lateral metasomal tergites and to some extent the pronotal shoulder and frons. In both species there are both white and golden brown setae in these areas, their distribution on the lateral metasoma is towards the posterior (anterior glabrous to sparsely setose, Fig. 210). Differing from *Scelio fremo* by the shorter metasoma and fore wings that surpass T5, the curved ventral margin of the villus, and the small obliterated patch of sculpture on the ventral mesopleural depression.

###### Etymology.

The epithet is used as a noun in apposition derived from the Latin word for murmur, whisper, hum, or buzz.

###### Link to distribution map.

http://hol.osu.edu/map-large.html?id=244985

###### Material examined.

*Holotype*, female: **CENTRAL AFRICAN REPUBLIC**: Sangha-Mbaéré Préf. Écon., 38.6km (173°) S Lidjombo, lowland rainforest, CAR01-M177, Dzanga-Ndoki National Park, 02°21.60'N, 16°03.20'E, 350m, 22.V–23.V.2001, malaise trap, S. van Noort, OSUC 213654 (deposited in SAMC). *Paratypes*: (1 female, 15 males) **CENTRAL AFRICAN REPUBLIC**: 7 males, OSUC 212523–212526 (CNCI); OSUC 211831, 213672, 214189 (SAMC). **GHANA**: 6 males, OSUC 212502–212503 (CNCI); OSUC 250737–250740 (OSUC). **GUINEA**: 1 female, OSUC 213136 (CNCI). **LIBERIA**: 1 male, OSUC 244096 (USNM). **NIGERIA**: 1 male, OSUC 211382 (CNCI).

###### Comments.

At present only two females are known, and while we here treat them as one species, they may ultimately warrant division. There is some difference in the general robustness of sculpture between the two individuals, the larger (OSUC 213654) being more robust in general, with less of a longitudinal trend on the mesoscutum and a broader gena (lateral view). *Scelio susurro* appears to be related to *Scelio exaratus* (Kieffer) from the Seychelles (not treated here, but see http://hol.osu.edu/index.html?id=5226), the two sharing obliterated patches on the mesopleural depression, the arrangement of setae on the lateral metasoma, infuscate wings, and a similar habitus. The pattern of sculpture on the mesoscutum (predominantly longitudinal with few reticulations) and narrow gena differentiate *Scelio exaratus* from the present species. *Scelio susurro* is somewhat intermediate between the brown and white subgroups, with a “salt and pepper” setal pattern (both white and brown mixed together) in various locations on the body (see Diagnosis). The brown pilosity of the mesoscutum is more typically found in brown subgroup species. Some of the tentatively associated males have the posterior lateral tergal setal patches brown contrasting with the predominantly white setae in females. The base of the scape in the two females is slightly lighter than the apex. In one the scape could be considered dark yellow, though we expect the scape to be brown throughout in a majority of individuals.

##### Scelio tono

Yoder sp. n.

http://zoobank.org/676BB0D5-E407-4F8A-AAEC-9775308F0C1B

urn:lsid:biosci.ohio-state.edu:osuc_concepts:244976

http://species-id.net/wiki/Scelio_tono

**Figures 211–216.**
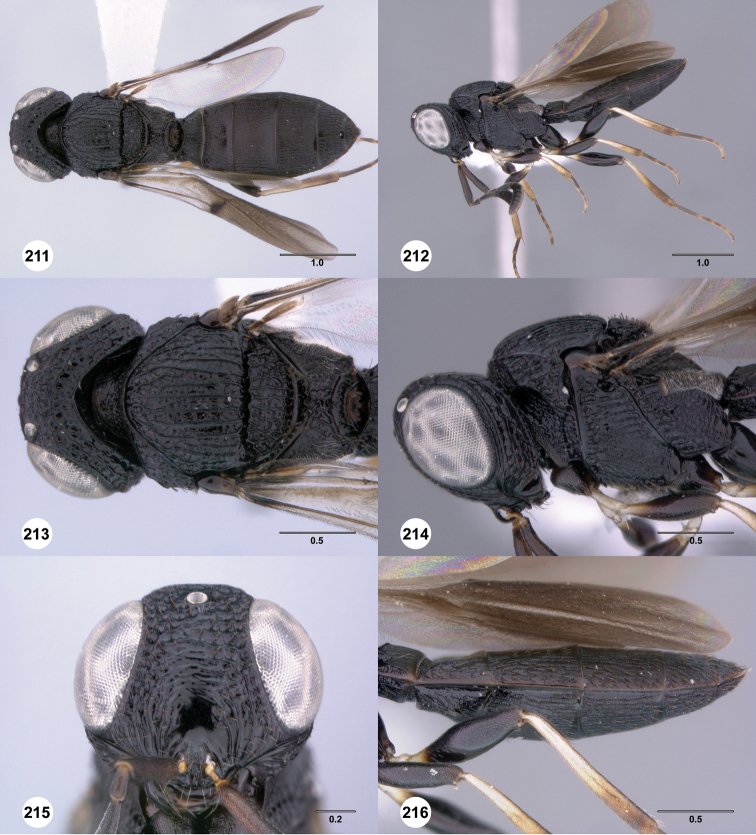
^149^ *Scelio tono* sp. n., holotype female (OSUC 211832). **211** Habitus, dorsal view **212** Habitus, lateral view **213** Head and mesosoma, dorsal view **214** Head and mesosoma, lateral view **215** Head, anterior view **216** Metasoma, lateral view. Scale bars in millimeters.

Figures 211–216 ; Morphbank ^50^

###### Description.

Female body length: 4.72–5.31 mm (n=7). Color of pilosity of dorsal head in female: golden to brown. Occipital carina in female: percurrent. Color of pilosity of the frons below the anterior ocellus in female: predominantly golden to brown. Pilosity of eye in female: absent. Medial keel on interantennal process: absent. Width of lower gena in lateral view: narrowing dorsally, posterior margin of lower half of gena angled with respect to posterior orbit. Genal carina: present. Color of genal pilosity: brown. Color of scape in female: brown to dark brown throughout. Surface of the pronotal nucha in female: predominantly sculptured. Color of pilosity of pronotal shoulder in female: golden to dark brown, concolorous with that of mesoscutum. Sculpture of medial mesoscutum in female: predominantly longitudinally strigose to rugulose. Color of pilosity of mesoscutum in female: predominantly light brown to brown. Notaulus in female: present as more or less uninterrupted channel in posterior 1/2 of mesoscutum. Notaulus in male: present as more or less uninterrupted channel in posterior 1/2 of mesoscutum. Form of axillular carina in female: bladelike or carinate in posterolateral corner but not forming distinct lobe. Pilosity of propodeal nucha: absent. Pilosity of netrion: absent. Surface of mesopleural depression in female: sculptured throughout. Form of ventral margin of villus in female: bent ventrally in posterior, obviously not straight throughout. Color of coxae in female: brown. Color of hind femur: dark brown throughout. Color of hind tibia: yellow at extreme base, otherwise light brown. Fore wing length in female: apex between anterior margin of T5 and posterior margin of T6; apex surpassing posterior margin of T6. Color of metasoma: entirely dark brown. Sculpture of laterotergites in female: predominantly smooth. Pilosity of laterotergites in female: absent. Sculpture of medial T1 in female: most prominent elements predominantly longitudinal. Sculpture of medial T2 in female: most prominent elements predominantly longitudinal. Pattern of sculpture on T3–T5 in female: T3 predominantly reticulate, T4–T5 predominantly longitudinally striate to strigose. Color of pilosity on lateral T3–T5 in female: predominantly golden to brown. Lateral profile of T6 in female: more or less horizontal. Sculpture of T6 in female: predominantly rugulose to reticulate. Sculpture of lateral metasomal sternal bar in female: predominantly smooth to slightly irregularly rugose. Distribution of felt fields: 2 pairs present (S2, S3).

###### Diagnosis.

Most similar to *Scelio cano* which shares the brown pilosity of the pronotal shoulder and mesoscutum, the brown scape and similarly colored tibiae (Fig. 212). Differing from *Scelio cano* by the combination of the presence of notauli (indicated as a clear channel in posterior half vs. more or less obscured) and the well-developed axillular carina that extends above the dorsal surface of the mesoscutellum (not expanded past dorsal surface in *Scelio cano*).

###### Etymology.

The epithet is used as a noun in apposition derived from the Latin word for sound.

###### Link to distribution map.

http://hol.osu.edu/map-large.html?id=244976

###### Material examined.

*Holotype*, female: **CENTRAL AFRICAN REPUBLIC**: Sangha-Mbaéré Préf. Écon., 38.6km (173°) S Lidjombo, lowland rainforest, CAR01-Y52, Dzanga-Ndoki National Park, 02°21.60'N, 16°03.20'E, 350m, 21.V–27.V.2001, yellow pan trap, S. van Noort, OSUC 211832 (deposited in SAMC). *Paratypes*: (6 females) **CAMEROON**: 5 females, OSUC 211222, 212459, 212461–212462 (CNCI); OSUC 212460 (OSUC). **CENTRAL AFRICAN REPUBLIC**: 1 female, OSUC 211833 (SAMC).

###### Comments.

The occiput is nearly completely smooth and more or less vertical. The gena is relatively narrow (Fig. 214), with a slight indication of a genal carina dorsally. The fore wing in some females appears to just barely extend past the apex of the metasoma.

##### Scelio tristis

Nixon

http://zoobank.org/6B9A6590-44CA-446E-9680-FA5DCE583EFC

urn:lsid:biosci.ohio-state.edu:osuc_concepts:5348

http://species-id.net/wiki/Scelio_tristis

**Figures 217–220.**
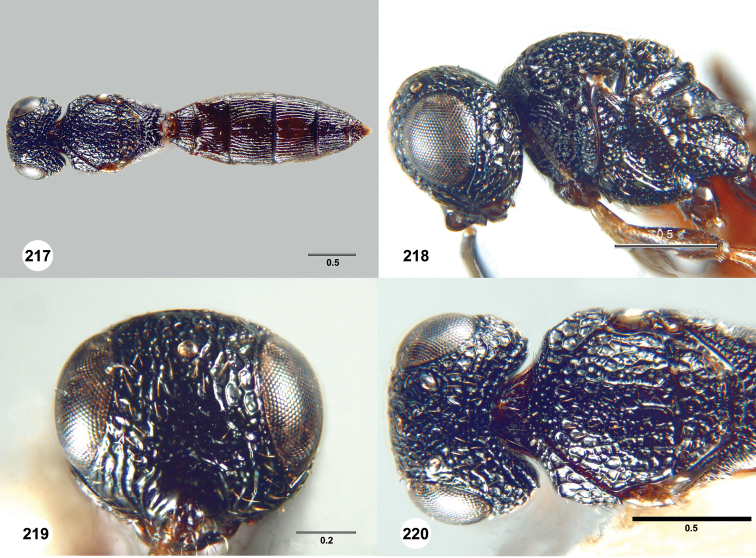
^150^ *Scelio tristis* Nixon, holotype female (B.M. TYPE HYM 9.539). **217** Habitus, dorsal view **218** Head and mesosoma, lateral view **219** Head, anterior view **220** Head and mesosoma, dorsal view. Photographs by A. Polaszek. Scale bars in millimeters.

Figures 217–220 ; Morphbank ^51^

Scelio tristis Nixon, 1958: 314 (original Description. Keyed); Masner, 1965: 95 (type information).

###### Description.

Female body length: 4.10 mm (n=1). Color of pilosity of dorsal head in female: golden to brown. Occipital carina in female: percurrent. Color of pilosity of the frons below the anterior ocellus in female: predominantly golden to brown. Pilosity of eye in female: absent. Medial keel on interantennal process: absent. Width of lower gena in lateral view: wide, posterior margin of lower half of gena parallel to posterior orbit. Genal carina: absent. Color of genal pilosity: brown. Color of scape in female: brown to dark brown throughout. Surface of the pronotal nucha in female: predominantly sculptured. Color of pilosity of pronotal shoulder in female: golden to dark brown, concolorous with that of mesoscutum. Sculpture of medial mesoscutum in female: predominantly angular reticulate to rugulose. Color of pilosity of mesoscutum in female: predominantly light brown to brown. Notaulus in female: indicated by a row of cells. Form of axillular carina in female: small, not particularly expanded or projected from the lateral edge of the mesoscutellum. Pilosity of propodeal nucha: present. Pilosity of netrion: absent. Surface of mesopleural depression in female: sculptured throughout. Form of ventral margin of villus in female: bent ventrally in posterior, obviously not straight throughout. Color of coxae in female: brown. Color of hind femur: dark brown throughout. Color of hind tibia: brown throughout. Fore wing length in female: apex between anterior margin of T5 and posterior margin of T6. Color of metasoma: entirely black. Sculpture of laterotergites in female: predominantly smooth. Pilosity of laterotergites in female: absent. Sculpture of medial T1 in female: most prominent elements predominantly longitudinal. Sculpture of medial T2 in female: most prominent elements predominantly longitudinal. Pattern of sculpture on T3-T5 in female: predominantly longitudinally striate. Color of pilosity on lateral T3-T5 in female: predominantly golden to brown. Lateral profile of T6 in female: more or less horizontal. Sculpture of T6 in female: predominantly rugulose to reticulate. Sculpture of lateral metasomal sternal bar in female: predominantly smooth to slightly irregularly rugose. Distribution of felt fields: 2 pairs present (S2, S3).

###### Diagnosis.

Differing from other brown subgroup species by the combination of the uniformly brown scape and tibiae, and the distinct notauli with thickened medial margins.

###### Link to distribution map.

http://hol.osu.edu/map-large.html?id=5348

###### Material examined.

*Holotype*, female: **UGANDA**: Rwenzori (Ruwenzori) Mountains, 6000ft, VIII-1940, T. H. C. Taylor, B.M. TYPE HYM. 9.539 (deposited in BMNH).

###### Comments.

Nixon (1958) recorded the unique holotype as collected in 1941, but the label data clearly read 1940. The holotype was earlier dissected with the parts mounted separately on a single card point. It resembles the Madagascan *Scelio ructo*, differing in the broader gena, more elongate metasoma and several aspects of color and sculpture.

#### Additional unplaced *howardi*-group species

##### Scelio effervesco

Yoder sp. n.

http://zoobank.org/E0DFE285-85E9-40F1-AF2F-F3FCA826656B

urn:lsid:biosci.ohio-state.edu:osuc_concepts:244971

http://species-id.net/wiki/Scelio_effervesco

**Figures 221–226.**
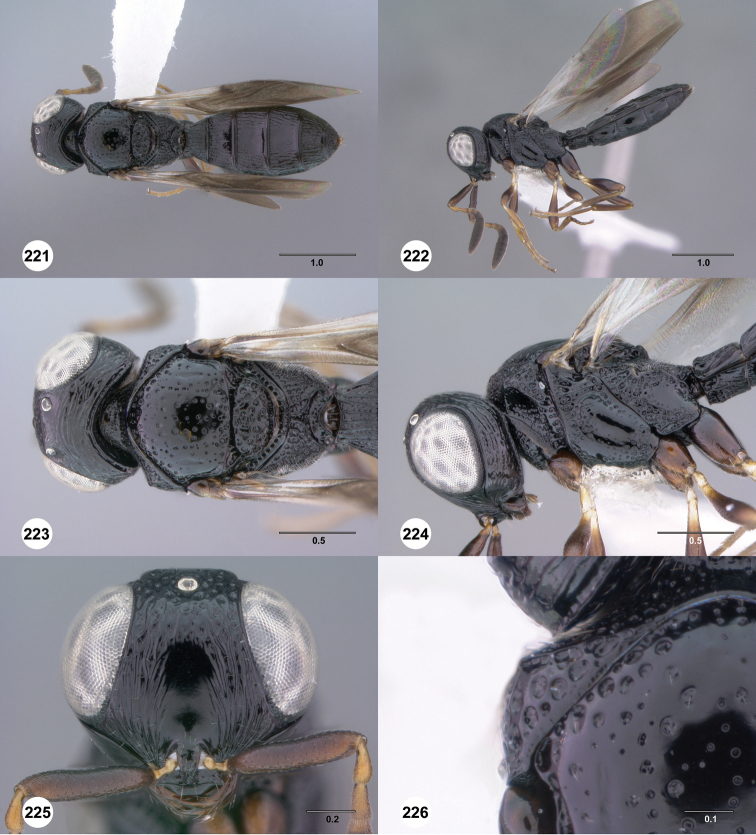
^151^ *Scelio effervesco* sp. n., holotype female (CASENT 2134281). **221** Habitus, dorsal view **222** Habitus, lateral view **223** Head and mesosoma, dorsal view **224** Head and mesosoma, lateral view **225** Head, anterior view **226** Anterolateral mesosoma, dorsal view. Scale bars in millimeters.

Figures 12 , 221–226 ; Morphbank ^52^

###### Description.

Female body length: 3.98–4.34 mm (n=4). Male body length: 3.76–4.44 mm (n=2). Color of pilosity of dorsal head in female: white. Occipital carina in female: percurrent. Color of pilosity of the frons below the anterior ocellus in female: predominantly white. Pilosity of eye in female: absent. Medial keel on interantennal process: absent. Width of lower gena in lateral view: wide, posterior margin of lower half of gena parallel to posterior orbit. Genal carina: absent. Color of genal pilosity: brown. Color of scape in female: brown to dark brown throughout. Surface of the pronotal nucha in female: predominantly smooth. Color of pilosity of pronotal shoulder in female: white to light brown, lighter than that of mesoscutum. Sculpture of medial mesoscutum in female: smooth with rounded punctures. Color of pilosity of mesoscutum in female: predominantly white to off-white. Notaulus in female: not delimited. Notaulus in male: not delimited. Form of axillular carina in female: small, not particularly expanded or projected from the lateral edge of the mesoscutellum. Pilosity of propodeal nucha: absent. Pilosity of netrion: absent. Surface of mesopleural depression in female: with large smooth patch. Form of ventral margin of villus in female: very slightly concave, almost straight. Color of coxae in female: brown. Color of hind femur: dark brown throughout. Color of hind tibia: yellow throughout. Fore wing length in female: apex surpassing posterior margin of T6. Color of metasoma: entirely black. Sculpture of laterotergites in female: predominantly aciculate. Pilosity of laterotergites in female: absent. Sculpture of medial T1 in female: most prominent elements predominantly longitudinal. Sculpture of medial T2 in female: most prominent elements predominantly longitudinal. Pattern of sculpture on T3–T5 in female: predominantly longitudinally striate. Color of pilosity on lateral T3–T5 in female: predominantly white. Lateral profile of T6 in female: more or less horizontal. Sculpture of T6 in female: predominantly rugulose to reticulate. Sculpture of lateral metasomal sternal bar in female: predominantly smooth to slightly irregularly rugose. Distribution of felt fields: 3 pairs present (S2, S3, S4).

###### Diagnosis.

Unique among all Afrotropical *Scelio* in the presence of 3 well-developed pairs of felt fields (on S2, S3 and S4) and the nearly completely smooth mesoscutum with only small punctures (vs. angular to roundly reticulate or striate strigose).

###### Etymology.

The epithet is used as a noun in apposition derived from the Latin word for boil over, in reference to the bubblelike sculpture of the mesoscutellum.

###### Link to distribution map.

http://hol.osu.edu/map-large.html?id=244971

###### Material examined.

*Holotype*, female: **MADAGASCAR**: Fianarantsoa Auto. Prov., Talatakely, Bellevue, secondary tropical forest, MA-02-09C-04, Ranomafana National Park, 21°15.99'S, 47°25.21'E, 1020m, 22.XI–28.XI.2001, malaise trap, R. Harin’Hala, CASENT 2134281 (deposited in CASC). *Paratypes*: **MADAGASCAR**: 3 females, 2 males, CASENT 2043535, 2132796, 2132826, 2134282 (CASC); CASENT 2133413 (OSUC).

###### Comments.

*Scelio effervesco* is aberrant in many ways, and is only tentatively placed into the *howardi*-group on the basis of the presence of a reduced propodeal projection and associated concavity. The form of the propodeal projection differs, however, from other *howardi*-group species in that there is not an acute outer corner. In this respect the form of the propodeum of *Scelio effervesco* more closely resembles that of species in the *irwini*-group. The pilosity throughout is extremely short and determining the precise color in specific areas is not possible, though it appears to be off-white to light brown. The legs and antennae of males are yellow throughout as compared to the brown of females.

##### Scelio destico

Yoder sp. n.

http://zoobank.org/0DB0FF23-6300-4034-9F7E-0C029A7F450F

urn:lsid:biosci.ohio-state.edu:osuc_concepts:244987

http://species-id.net/wiki/Scelio_destico

Figures 17 , 25 , 227–232 ; Morphbank ^53^

###### Description.

Female body length: 3.75–4.34 mm (n=20). Male body length: 3.80–4.32 mm (n=13). Color of pilosity of dorsal head in female: white; golden to brown. Occipital carina in female: percurrent. Color of pilosity of the frons below the anterior ocellus in female: predominantly white. Pilosity of eye in female: absent; present. Medial keel on interantennal process: absent. Width of lower gena in lateral view: narrowing dorsally, posterior margin of lower half of gena angled with respect to posterior orbit. Genal carina: absent. Color of genal pilosity: white. Color of scape in female: brown to dark brown throughout. Surface of the pronotal nucha in female: predominantly sculptured. Color of pilosity of pronotal shoulder in female: white to light brown, lighter than that of mesoscutum. Sculpture of medial mesoscutum in female: predominantly angular reticulate to rugulose. Color of pilosity of mesoscutum in female: predominantly yellow to golden. Notaulus in female: not delimited; indicated by a row of cells. Form of axillular carina in female: small, not particularly expanded or projected from the lateral edge of the mesoscutellum. Pilosity of propodeal nucha: absent. Pilosity of netrion: absent. Surface of mesopleural depression in female: sculptured throughout; with small smooth patch ventrally. Form of ventral margin of villus in female: very slightly concave, almost straight. Color of coxae in female: brown. Color of hind femur: dark brown with white base. Color of hind tibia: yellow throughout. Fore wing length in female: apex between anterior margin of T5 and posterior margin of T6. Color of metasoma: entirely dark brown. Sculpture of laterotergites in female: predominantly smooth. Pilosity of laterotergites in female: absent. Sculpture of medial T1 in female: most prominent elements predominantly longitudinal. Sculpture of medial T2 in female: most prominent elements predominantly reticulate rugulose; most prominent elements predominantly longitudinal. Pattern of sculpture on T3–T5 in female: T3 predominantly reticulate, T4–T5 predominantly longitudinally striate to strigose. Color of pilosity on lateral T3–T5 in female: predominantly white; predominantly golden to brown; more or less evenly split between white and brown. Lateral profile of T6 in female: more or less horizontal. Sculpture of T6 in female: predominantly rugulose to reticulate. Sculpture of lateral metasomal sternal bar in female: minutely reticulate throughout. Distribution of felt fields: 2 pairs present (S2, S3).

###### Diagnosis.

Very similar to *Scelio pipilo* which shares a similar habitus, reticulate sculpture of the frons, brown to dark brown scape, somewhat bulging eye, and brown pilosity of the lateral metasoma. Differing from *Scelio pipilo* by the narrow gena with genal carina weakly developed (gena wide and no genal carina discernible in *Scelio pipilo*).

**Figures 227–232.**
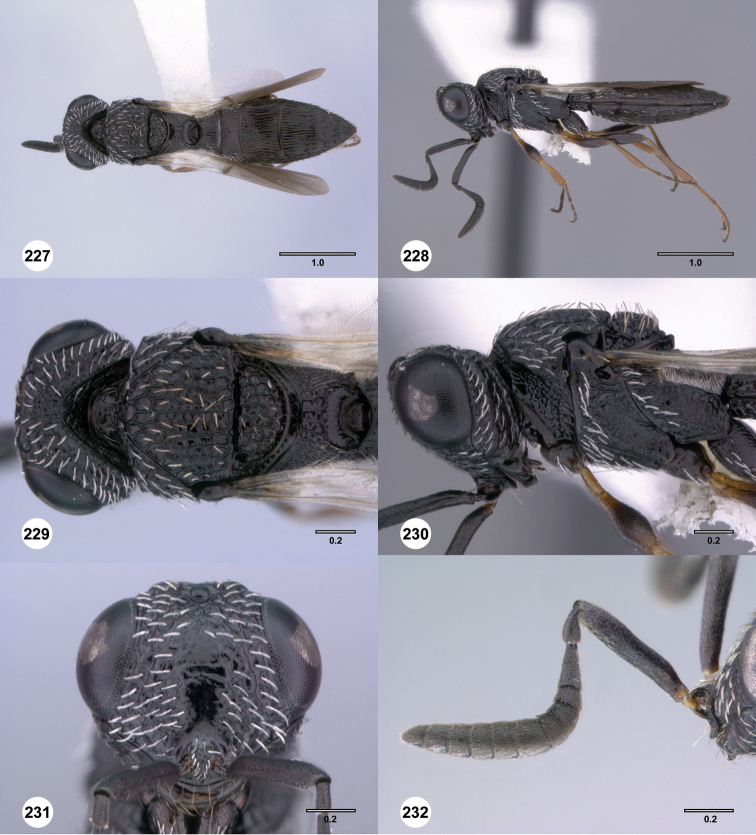
^152^ *Scelio destico* sp. n., paratype female (OSUC 214085). **227** Habitus, dorsal view **228** Habitus, lateral view **229** Head and mesosoma, dorsal view **230** Head and mesosoma, lateral view **231** Head, anterior view **232** Antenna, lateral view. Scale bars in millimeters.

###### Etymology.

The epithet is used as a noun in apposition derived from the Latin word for squeak like a mouse.

###### Link to distribution map.

http://hol.osu.edu/map-large.html?id=244987

###### Material examined.

*Holotype*, female: **KENYA**: Nyanza Prov., Nyangera, 00°03'55.9"S, 34°04'52.2"E, 10.X–6.XII.2003, malaise trap, I. Prikryl, OSUC 214122 (deposited in CNCI). *Paratypes*: (32 females, 13 males) **BENIN**: 1 female, OSUC 212848 (CNCI). BURKINA FASO: 1 female, OSUC 214085 (CNCI). IVORY COAST: 11 females, OSUC 211360, 212859, 212919, 212921, 213014, 213062, 213066, 213069 (CNCI); OSUC 142586, 142593, 57143 (OSUC). **KENYA**: 12 females, 5 males, OSUC 214104, 214113, 214115–214116, 214138, 214140, 214142, 214146, 214148–214152, 214181, 234634, 234701, 234703 (CNCI). **NIGERIA**: 2 females, 7 males, OSUC 212170, 212178, 212612, 212626, 212631–212632, 212688–212689, 213034 (CNCI). **UGANDA**: 1 female, 1 male, OSUC 214136, 214160 (CNCI). **ZIMBABWE**: 4 females, OSUC 212100, 212344, 213006–213007 (CNCI). *Other material*: (3 females) **GHANA**: 1 female, OSUC 213557 (OSUC). **NIGERIA**: 2 females, OSUC 212810, 213156 (CNCI).

###### Comments.

The villus of *Scelio destico* is very similar to that of *Scelio pipilo*. However, in most specimens it tends to be slightly more concave ventrally, while in *Scelio pipilo* it is more or less straight. *Scelio destico* is smaller than *Scelio pipilo* (3.80–4.32 mm vs. 4.64–5.52 mm in females). The color of pilosity of the lateral portion of the metasoma in *Scelio destico* is highly variable, from more or less completely white to nearly completely brown. This is more variation than observed in *Scelio pipilo* which has predominantly brown setae in all individuals. The interstitial sculpture (between reticulations or longitudinal striae) of the lateral metasoma is somewhat less dense (more smooth patches) and more irregular in *Scelio destico* than in *Scelio pipilo* in which there is dense colliculate sculpture more or less throughout. Three individuals from Nigeria and Ghana (OSUC 212810, 213156, 213557) have the scape is distinctly yellow at the base and brown apically as in *Scelio howardi*. These three are not included in the paratype series and may ultimately represent a separate species based on the scape color and the absence of the genal carina.

##### Scelio memorabilis

Yoder sp. n.

http://zoobank.org/327231C3-8FF7-4C29-ABA5-17A82B0D6C4D

urn:lsid:biosci.ohio-state.edu:osuc_concepts:244972

http://species-id.net/wiki/Scelio_memorabilis

**Figures 233–238.**
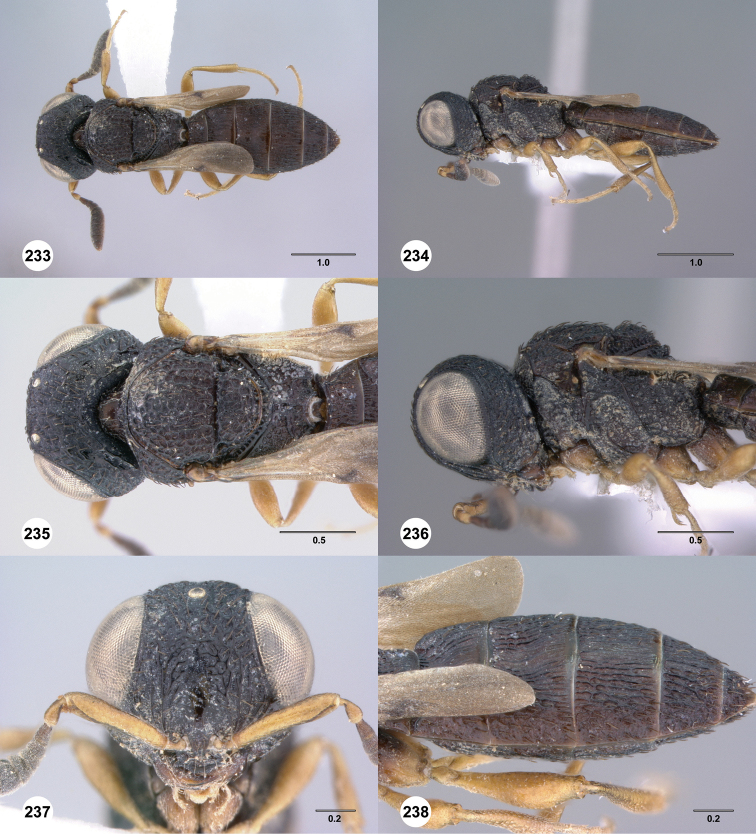
^153^ *Scelio memorabilis* sp. n., holotype female (OSUC 244026). **233** Habitus, dorsal view **234** Habitus, lateral view **235** Head and mesosoma, dorsal view **236** Head and mesosoma, lateral view **237** Head, anterior view; **238** Metasoma, dorsolateral view. Scale bars in millimeters.

Figures 22 , 233–238 ; Morphbank ^54^

###### Description.

Female body length: 4.08 mm (n=1). Color of pilosity of dorsal head in female: golden to brown. Occipital carina in female: percurrent. Color of pilosity of the frons below the anterior ocellus in female: predominantly golden to brown. Pilosity of eye in female: absent. Medial keel on interantennal process: absent. Width of lower gena in lateral view: wide, posterior margin of lower half of gena parallel to posterior orbit. Genal carina: absent. Color of genal pilosity: brown. Color of scape in female: yellow throughout. Surface of the pronotal nucha in female: predominantly smooth. Color of pilosity of pronotal shoulder in female: golden to dark brown, concolorous with that of mesoscutum. Sculpture of medial mesoscutum in female: predominantly angular reticulate to rugulose. Color of pilosity of mesoscutum in female: predominantly light brown to brown. Notaulus in female: indicated by a row of cells. Form of axillular carina in female: small, not particularly expanded or projected from the lateral edge of the mesoscutellum. Pilosity of propodeal nucha: absent. Pilosity of netrion: absent. Surface of mesopleural depression in female: sculptured throughout. Form of ventral margin of villus in female: bent ventrally in posterior, obviously not straight throughout. Color of coxae in female: basal half brown, distal half yellow. Color of hind femur: dark brown throughout. Color of hind tibia: yellow throughout. Fore wing length in female: apex not reaching anterior margin of T5. Color of metasoma: light reddish brown. Sculpture of laterotergites in female: predominantly aciculate. Pilosity of laterotergites in female: absent. Sculpture of medial T1 in female: most prominent elements predominantly reticulate rugulose. Sculpture of medial T2 in female: most prominent elements predominantly longitudinal. Pattern of sculpture on T3–T5 in female: predominantly longitudinally striate. Color of pilosity on lateral T3–T5 in female: predominantly golden to brown. Lateral profile of T6 in female: vertically sloped in posterior half. Sculpture of T6 in female: predominantly rugulose to reticulate. Sculpture of lateral metasomal sternal bar in female: predominantly smooth to slightly irregularly rugose. Distribution of felt fields: 3 pairs present (S2, S3, S4).

###### Diagnosis.

Superficially similar to other Afrotropical *howardi*-group species of the brown subgroup, sharing the brown pilosity of the pronotal shoulder. This species may be distinguished from all African *Scelio* by the brachypterous wings (Fig. 233). The robust sculpture of the lateral metasoma (Fig. 238) is also relatively distinct for African *Scelio*.

###### Etymology.

The epithet is an adjective, the Latin word for memorable, in reference to the unique state of the wing development.

###### Link to distribution map.

http://hol.osu.edu/map-large.html?id=244972

###### Material examined.

*Holotype*, female: **SOUTH AFRICA**: KwaZulu-Natal Prov., coastal forest, KW00-Y71, Umtamvuna Provincial Nature Reserve, 31°03.798'S, 30°10.448'E, 20m, 13.XI–20.XI.2000, yellow pan trap, S. van Noort, OSUC 244026 (deposited in SAMC).

###### Comments.

*Scelio memorabilis* is apparently the only known brachypterous species of *Scelio*. Though only a single specimen is known, its odd combination of characters states suggests that it warrants species status. The basal half of the coxa is brown and the apex yellow. The sculpture of the metanotum is somewhat rugulose reticulate, though it is still dominated by longitudinal elements. We have coded the third pair of felt fields as present which is quite rare for Afrotropical *Scelio. A*dditional specimens will be required to confirm their consistent presence.

##### Scelio philippinensis

Ashmead

http://zoobank.org/36FC99A8-50F2-404F-925B-2AB225DE7F12

urn:lsid:biosci.ohio-state.edu:osuc_concepts:5303

http://species-id.net/wiki/Scelio_philippinensis

**Figures 239–244.**
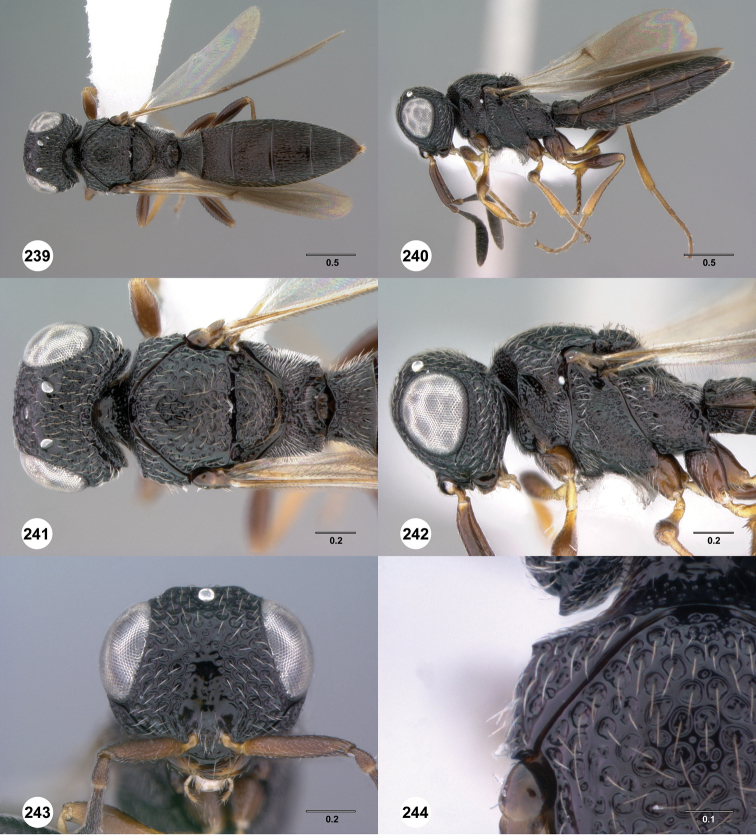
^154^ *Scelio philippinensis* Ashmead, female (OSUC 213315). **239** Habitus, dorsal view **240** Habitus, lateral view **241** Head and mesosoma, dorsal view **242** Head and mesosoma, lateral view **243** Head, anterior view **244** Anterolateral portion of mesosoma, dorsal view. Scale bars in millimeters.

Figures 18 , 239–244 ; Morphbank ^55^

Scelio philippinensis Ashmead, 1905: 963 (original description); Kieffer 1908: 126 (keyed); Kieffer 1914: 289 (keyed); Kieffer 1926: 310, 321 (description, keyed); Timberlake 1932: 157 (diagnosis); Masner and Muesebeck 1968: 45 (type information).
Scelio (Scelio) philippinensis Ashmead: Kieffer 1910: 74 (subgeneric assignment).

###### Description.

Female body length: 3.32–4.06 mm (n=15). Male body length: 3.46–4.00 mm (n=2). Color of pilosity of dorsal head in female: golden to brown. Occipital carina in female: percurrent. Color of pilosity of the frons below the anterior ocellus in female: predominantly white; predominantly golden to brown. Pilosity of eye in female: absent. Medial keel on interantennal process: absent. Width of lower gena in lateral view: wide, posterior margin of lower half of gena parallel to posterior orbit. Genal carina: absent. Color of genal pilosity: white. Color of scape in female: brown to dark brown throughout. Surface of the pronotal nucha in female: predominantly smooth. Color of pilosity of pronotal shoulder in female: golden to dark brown, concolorous with that of mesoscutum. Sculpture of medial mesoscutum in female: predominantly with rounded cells. Color of pilosity of mesoscutum in female: predominantly light brown to brown. Notaulus in female: not delimited. Notaulus in male: not delimited. Form of axillular carina in female: small, not particularly expanded or projected from the lateral edge of the mesoscutellum. Pilosity of propodeal nucha: present. Pilosity of netrion: present. Surface of mesopleural depression in female: sculptured throughout. Form of ventral margin of villus in female: very slightly concave, almost straight. Color of coxae in female: brown. Color of hind femur: dark brown throughout. Color of hind tibia: yellow at extreme base, otherwise light brown. Fore wing length in female: apex between anterior margin of T5 and posterior margin of T6. Color of metasoma: entirely dark brown. Sculpture of laterotergites in female: predominantly smooth. Pilosity of laterotergites in female: present. Sculpture of medial T1 in female: most prominent elements predominantly longitudinal. Sculpture of medial T2 in female: most prominent elements predominantly longitudinal. Pattern of sculpture on T3–T5 in female: predominantly longitudinally striate. Color of pilosity on lateral T3–T5 in female: predominantly white; predominantly golden to brown. Lateral profile of T6 in female: vertically sloped in posterior half. Sculpture of T6 in female: predominantly rugulose to reticulate. Sculpture of lateral metasomal sternal bar in female: predominantly smooth to slightly irregularly rugose. Distribution of felt fields: 2 pairs present (S2, S3).

###### Diagnosis.

Easily differentiated from all other Afrotropical *howardi*-group species by the setose netrion and the sparsely setose anterior of the propodeal nucha.

###### Link to distribution map.

http://hol.osu.edu/map-large.html?id=5303

###### Associations.

Emerged from *Oxya* Serville [Orthoptera: Acrididae].

###### Material examined.

*Holotype*, male, **PHILIPPINES**: Manila, R. Brown, USNM no. 8336 (deposited in USNM). *Other material*: (15 females, 2 males) **CAMEROON**: 1 female, OSUC 212920 (CNCI). **CENTRAL AFRICAN REPUBLIC**: 6 females, OSUC 213315, 213638, 213920, 214194, 244017, 254658 (SAMC). **GUINEA**: 1 female, 1 male, OSUC 211270–211271 (CNCI). **IVORY COAST**: 7 females, OSUC 213073, 213077, 213220, 213224, 213242–213243 (CNCI); OSUC 142590 (OSUC). **MOZAMBIQUE**: 1 male, OSUC 213106 (CNCI).

###### Comments.

We here adopt a broad concept of *Scelio philippinensis* which includes both African and Oriental specimens. While specimens from the two regions can be consistently distinguished on the basis of coxal color–Africa: brown, Oriental: yellow–we found no other corroborating morphological characters. We suspect that it will be necessary to adopt molecular or morphometric approaches to further test species boundaries of this taxon. *Scelio philippinensis* is highly atypical for Afrotropical *Scelio* in general. The setae of the anterior propodeal nucha are not seen in any other species of African *Scelio*. The color of the pilosity of the frons and the lateral metasoma is difficult to interpret due to its short length and narrow width. It appears that there is some variation, with individuals possessing either predominantly white or predominantly brown pilosity. The sculpture of the lateral metasomal sternal bar is variable: in some individuals the surface is more or less smooth, in others only a thin smooth strip is present ventrally.

##### Scelio pipilo

Yoder sp. n.

http://zoobank.org/CAFFCA2C-09BA-4D1A-8894-0BDD5C568EC8

urn:lsid:biosci.ohio-state.edu:osuc_concepts:244984

http://species-id.net/wiki/Scelio_pipilo

**Figures 245–250.**
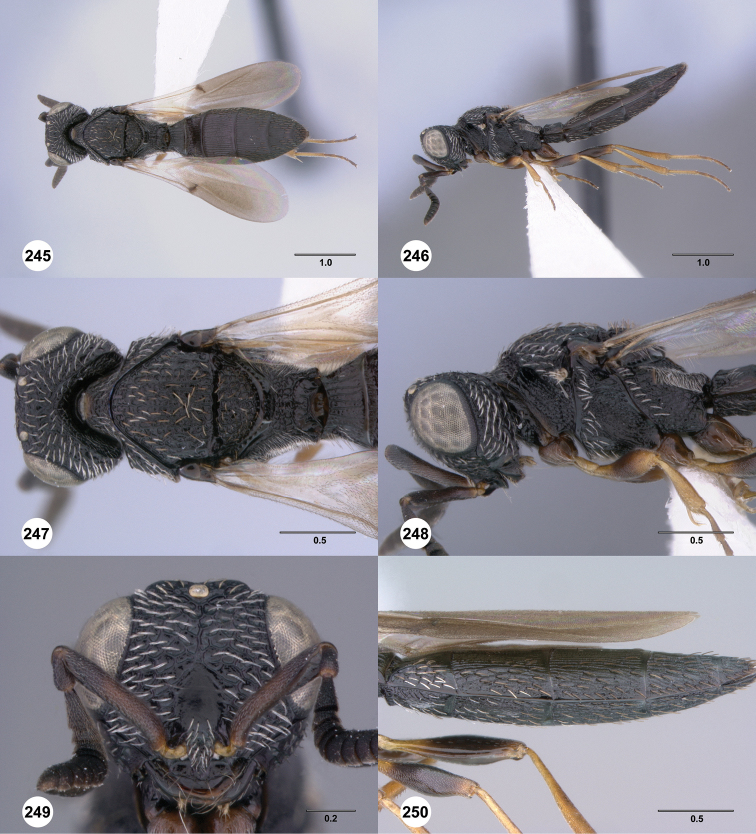
^155^ *Scelio pipilo* sp. n., paratype female (OSUC 214135). **245** Habitus, dorsal view **246** Habitus, lateral view **247** Head and mesosoma, dorsal view **248** Head and mesosoma, lateral view **249** Head, anterior view **250** Metasoma, lateral view. Scale bars in millimeters.

Figures 20 , 245–250 ; Morphbank ^56^

###### Description.

Female body length: 4.64–5.52 mm (n=13). Male body length: 4.20–5.39 mm (n=5). Color of pilosity of dorsal head in female: white. Occipital carina in female: percurrent. Color of pilosity of the frons below the anterior ocellus in female: predominantly white. Pilosity of eye in female: absent; present. Medial keel on interantennal process: absent. Width of lower gena in lateral view: wide, posterior margin of lower half of gena parallel to posterior orbit. Genal carina: absent. Color of genal pilosity: white. Color of scape in female: brown to dark brown throughout. Surface of the pronotal nucha in female: predominantly sculptured. Color of pilosity of pronotal shoulder in female: white to light brown, lighter than that of mesoscutum. Sculpture of medial mesoscutum in female: predominantly angular reticulate to rugulose. Color of pilosity of mesoscutum in female: predominantly yellow to golden. Notaulus in female: indicated by a row of cells. Form of axillular carina in female: small, not particularly expanded or projected from the lateral edge of the mesoscutellum. Pilosity of propodeal nucha: absent. Pilosity of netrion: absent. Surface of mesopleural depression in female: sculptured throughout. Form of ventral margin of villus in female: very slightly concave, almost straight. Color of coxae in female: brown. Color of hind femur: dark brown throughout. Color of hind tibia: yellow throughout. Fore wing length in female: apex between anterior margin of T5 and posterior margin of T6. Color of metasoma: entirely dark brown. Sculpture of laterotergites in female: predominantly smooth. Pilosity of laterotergites in female: absent. Sculpture of medial T1 in female: most prominent elements predominantly longitudinal. Sculpture of medial T2 in female: most prominent elements predominantly longitudinal. Pattern of sculpture on T3–T5 in female: T3 predominantly reticulate, T4–T5 predominantly longitudinally striate to strigose. Color of pilosity on lateral T3–T5 in female: predominantly golden to brown. Lateral profile of T6 in female: more or less horizontal. Sculpture of T6 in female: predominantly rugulose to reticulate. Sculpture of lateral metasomal sternal bar in female: minutely reticulate throughout. Distribution of felt fields: 2 pairs present (S2, S3).

###### Diagnosis.

Very similar to *Scelio destico* in habitus, reticulate sculpture of the frons, brown to dark brown scape, somewhat bulging eye, and brown pilosity of the lateral metasoma. This species differs in the absence of a genal carina and, in lateral view, the posterior margin of the lower half of the gena is parallel to the posterior orbit making the gena appear wider.

###### Etymology.

The epithet is used as a noun in apposition derived from the Latin word for chirp, twitter.

###### Link to distribution map.

http://hol.osu.edu/map-large.html?id=244984

###### Material examined.

*Holotype*, female: **CENTRAL AFRICAN REPUBLIC**: Sangha-Mbaéré Préf. Écon., 21.4km (53°) NE Bayanga, Mabéa Bai, lowland rainforest / marsh clearing, CAR01-M49, Dzanga-Ndoki National Park, 03°02.01'N, 16°24.57'E, 510m, 5.V–6.V.2001, malaise trap, S. van Noort, OSUC 244025 (deposited in SAMC). *Paratypes*: (12 females, 5 males) **BENIN**: 3 females, OSUC 211367, 211371–211372 (CNCI). **CAMEROON**: 1 female, OSUC 211217 (CNCI). **CENTRAL AFRICAN REPUBLIC**: 3 females, OSUC 213979 (OSUC); OSUC 254636–254637 (SAMC). **GHANA**: 1 female, OSUC 213554 (OSUC). **KENYA**: 1 female, 1 male, OSUC 214114, 214126 (CNCI). **NIGERIA**: 1 female, OSUC 212613 (CNCI). **UGANDA**: 2 females, 4 males, OSUC 214135, 214153–214154, 214159, 214161–214162 (CNCI).

### *Scelio ipomeae* species group

**Description.** *General*. Body size: small; moderate; large. Body length: 2.86–5.72 mm. Habitus: typical, mesosoma not dorsoventrally flattened. Body color: brown to dark brown, in some with metasoma yellow to orange. Fore leg color: concolorous with mid and hind legs. Sculpture: moderate to robust, reticulate to strigose, generally without longitudinal or parallel lineations. Wing type: macropterous.

*Pilosity*. General setation: moderate elongate and wide, variously vertically oriented. Thickened and truncate white pilosity: not typically present, or strongly truncate when so. Interommatidial pilosity: unknown. Genal pilosity density: moderate. Genal pilosity color: white. Number of anteclypeal setal pairs: 3. Ventrolateral postgenal cluster of erect setae: present. Antespiracular setal patch: small to moderate size, present immediately below posterior extension of lateral epomia. Netrion: glabrous. Propodeal shelf: present throughout except narrow medial strip. Pilosity of laterotergites: absent.

*Head*. Sculpture of head: predominantly reticulate to rugulose throughout, sculpture of the dorsal head very fine to partially obliterated. Ocelli size: moderate. Gap between antennal toruli and anteclypeus: unknown. Width of ventral head across mandibles: narrow, mandibles relatively compact; moderate, mandibles typically formed. Anteclypeus shape between outer teeth: thin immediately mesad of outer teeth, smoothly rounded to slightly trapezoidal medially, without sharp vertices, medially truncate to slightly concave. Malar sulcus: present. Medial portion of occipital carina: percurrent; broadly obliterated or obscured medially. Lateral portion of occipital carina: more or less linear throughout. Form of gena: broad, somewhat flattened, minimally rounded between posterior margin of eye and occipital carina. Genal carina: absent. RSS on A5 in males: present. Microsculpture at base of mandible: present; absent. Basal tooth of mandible: unknown.

*Mesosoma*. Shape of mesoscutum in lateral view: flattened, not or very weakly sloping towards pronotum anteriorly. Transverse pronotal carina in female: unknown. Surface of mesoscutum: unknown. Smooth or obliterated patches on mesoscutum: unknown. Surface of the pronotal collar in females: unknown. Axillula: small, clearly discernible only in lateral view. Propodeal corners: rounded, without pointed vertices. Epomia: present. Surface of oxter: often smooth or with sculpture obliterated or very faint. Fore wing length: reaching or surpassing anterior margin of T5 but not surpassing apex of metasoma. Fore wing submarginal vein near curve towards costal margin: tubular. Pictation of fore wing in female: present; absent.

*Metasoma*. Anterior margin of T1: concave, with short rim.

**Diagnosis.** Species of the *ipomeae*-group are most similar to those in the *walkeri*-group, both sharing a similar bent ventral margin of the villus and rounded posterolateral propodeal corners. Most species can be distinguished from *walkeri*-group species by the combination of a) the pattern of pilosity on lateral T1 in which there is dense line of fine pilosity in addition to the thicker pilosity (Fig. 268), b) the general absence of thick white truncate setae (present in *walkeri*-group species), c) the orange metasoma (brown to black in *walkeri*-group species), d) the presence of an RSS on male A5 (absent or apparently so at 60× in *walkeri*-group species), and 3) in most the absence of a well-defined basal tooth on the mandible (present in all *walkeri*-group species).

**Comments.** We consider the division of *walkeri* and *ipomeae*-groups as monophyletic units as tenuous and ultimately they may need to be combined. However, the division is pragmatically useful. Most species of the *ipomeae*-group have the characteristic orange metasoma, relatively reduced sculpture on the dorsal portion of the head (noted by Nixon 1958), a distinct pattern of pilosity on T1 (Fig. 268), broad gena, and apically broad A5 in males. The *ipomeae*-group can be further divided on the basis of the presence or absence of a percurrent transverse pronotal carina and general shape of the mesosoma. The mesosoma may be somewhat compact with a more or less vertical medial propodeal shelf (e.g. Fig. 284), or more typically formed with the propodeal shelf less strongly sloped (e.g. Fig. 266). Individuals of the former subgroup are robust and compact, with thicker coxae and femora.

**Key to *ipomeae*-group species** (also available online at http://www.waspweb.org/Platygastroidea/Keys/index.htm)

**Table:** 

1	Transverse pronotal carina percurrent (Fig. 286, *tpc*); mesosoma somewhat compact, with propodeal shelf short and strongly sloping (Figs 272, 275, 284); propodeal nucha smooth except for slight medial furrow (Fig. 274, *prn*)	2
–	Transverse pronotal carina interrupted medially by mesoscutum; mesosoma typically developed, with propodeal shelf flatter (e.g. Figs 254, 265, 266); propodeal nucha usually with transverse to reticulate sculpture in addition to medial furrow (Fig. 265)	5
2	Clypeus strongly projecting, subquadrate with rounded corners (Fig. 285); metasoma dark orange brown (Figs 281, 282); mesoscutellum transverse, broadly depressed posteromedially with posterolateral margins very slightly elevated (Fig. 283)	*Scelio transtrum*
–	Clypeus not strongly projecting (Figs 273, 280); metasoma brown to dark brown (Figs 269, 270); mesoscutellum semicircular to slightly transverse, evenly rounded posteriorly	3
3	Very small (2.86 mm); pronotal nucha sculptured throughout (Fig. 277); scape yellow; T6 with fine longitudinal sculpture (Somalia)	*Scelio somaliensis*
–	Moderately sized (4.40 mm); anterior half of pronotal nucha smooth (Fig. 274); scape dark brown; T6 with coarse rugulose reticulations (Malawi)	*Scelio ntchisii*
4	Anteclypeus strongly bilobed (Fig. 256); occipital carina broadly obliterated medially (Fig. 253); metasoma orange throughout (Figs 251, 252)	*Scelio aurantium*
–	Anteclypeus very weakly concave to truncate (Figs 261, 267); occipital carina percurrent or interrupted for only a very short distance medially; metasoma with at least T5–T6 brown, or brown throughout	5
5	Metasoma brown, with T5–T6 dark brown (Fig. 258); gena with sparse to moderate pilosity (Fig. 262); head somewhat rounded in lateral view (South Africa)	*Scelio impostor* Yoder
–	Metasoma orange with T5-T6 brown (Figs 263, 264); gena at narrowest point behind eye with dense thick setae (Fig. 266); head somewhat wedge-shaped in lateral view (widespread throughout Africa)	*Scelio ipomeae*

#### Scelio aurantium

Yoder sp. n.

http://zoobank.org/C33D6810-33DF-4D5D-8BC4-F910822056F7

urn:lsid:biosci.ohio-state.edu:osuc_concepts:244752

http://species-id.net/wiki/Scelio_aurantium

**Figures 251–256.**
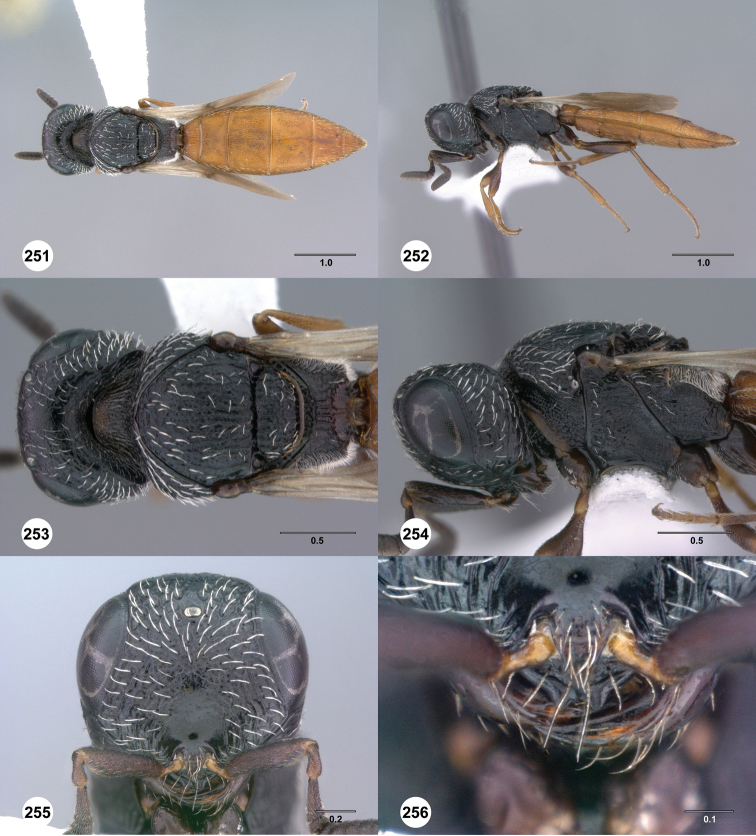
^156^ *Scelio aurantium* sp. n., holotype female (OSUC 212992). **251** Habitus, dorsal view **252** Habitus, lateral view **253** Head and mesosoma, dorsal view **254** Head and mesosoma, lateral view **255** Head, anterior view **256** Mouthparts, anterior view. Scale bars in millimeters.

Figures 251–256 ; Morphbank ^57^

##### Description.

Female body length: 5.12–5.72 mm (n=9). Male body length: 5.32–5.50 mm (n=2). Color of scape in female: brown. Surface of dorsal head in female: covered throughout with very fine sculpture. Occipital carina in female: broadly obliterated medially. Profile of posterior margin of head in lateral view: produced posteriorly, head appearing wedge-shaped. Width of genal setae: narrow to moderately wide. Shape of medial anteclypeus in female: strongly projected, trapezoidal, bilobed apically. Surface of mandible base in female: smooth. Form of mesosoma in female: typically formed, with propodeal shelf moderately elongate and clearly visible in dorsal view. Surface of pronotal nucha in female: sculptured throughout. Transverse pronotal carina in female: developed laterally, absent medially, not percurrent. Shape of mesoscutellum: semicircular to weakly transverse, evenly rounded posteriorly. Surface of propodeal nucha in female: with medial furrow, otherwise smooth. Surface of propodeal shelf in female: sculptured throughout. Color of metasoma in female: orange throughout. Sculpture of T6: finely longitudinally striae.

##### Diagnosis.

This species is most similar to *Scelio ipomeae* and *Scelio impostor* which share the medially interrupted transverse pronotal carina. It is easily distinguished from both these species by the prominently bilobed anteclypeus (truncate to slightly concave in both others), the broadly obliterated occipital carina (percurrent in both others), and the completely orange metasoma (apically brown in *Scelio ipomeae* and brown throughout in *Scelio impostor*, but see Comments for these species). *Scelio aurantium* is further recognizable by its relatively large size and slightly elongate habitus.

##### Etymology.

The epithet is used as a noun in apposition derived from the Latin word for orange, the fruit.

##### Link to distribution map.

http://hol.osu.edu/map-large.html?id=244752

##### Material examined.

*Holotype*, female: **SOUTH AFRICA**: Limpopo Prov., 15km NE Klaserie, woodland, Guernsey Farm, 19.XII-31.XII.1985, malaise trap/flight intercept trap, S. Peck & J. Peck, OSUC 212992 (deposited in CNCI). *Paratypes*: **SOUTH AFRICA**: 9 females, 2 males, OSUC 211286, 211393, 212308, 212672, 212985 (CNCI); OSUC 234720 (OSUC); OSUC 213354, 213370, 213395–213396, 213423 (SANC).

##### Comments.

The posterior propodeal margin in *Scelio aurantium* approaches the angular to dentate state seen in species of Afrotropical *ernstii*-group, although it does not reach that extreme. The dense fine pilosity of T1, characteristic of the *ipomeae*-group, is well developed. The humeral sulcus is relatively well-developed as a short broad channel along the posterolateral mesoscutum. *Scelio aurantium* is one of the few Afrotropical species in which the occipital carina is broadly obliterated medially. No variation of metasomal color was observed: it is orange throughout in all individuals. In most specimens the mesoscutum at a position roughly corresponding to the location of the parapsidal lines is irregularly flattened and slightly polished in a short strip. Two males are associated and well match the females.

#### Scelio impostor

Yoder sp. n.

http://zoobank.org/2904255F-3B70-437A-A86A-9227A582EE87

urn:lsid:biosci.ohio-state.edu:osuc_concepts:244754

http://species-id.net/wiki/Scelio_impostor

**Figures 257–262.**
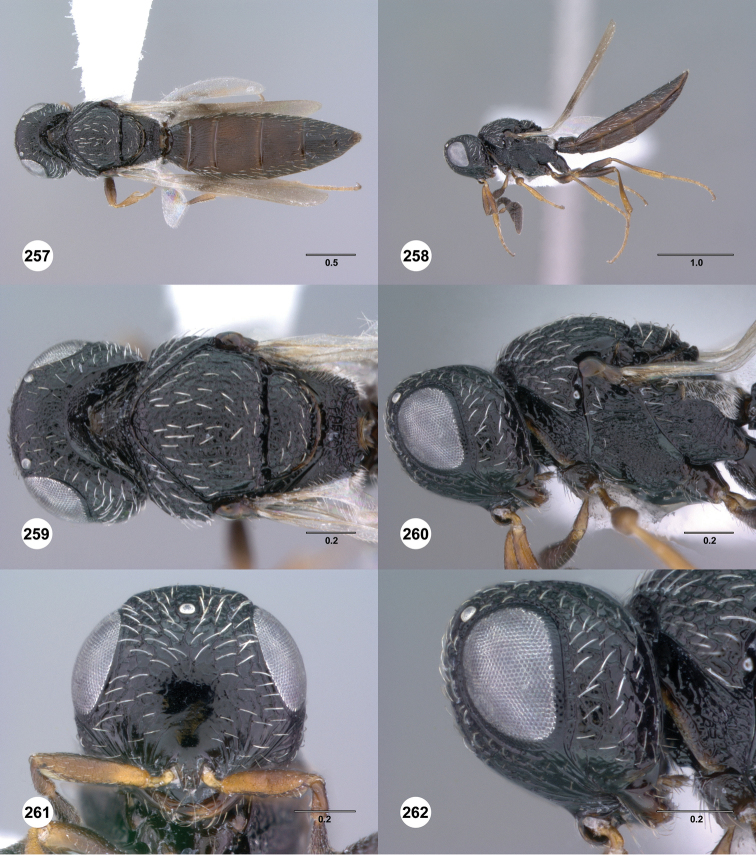
^157^ *Scelio impostor* sp. n., holotype female (OSUC 250816). **257** Habitus, dorsal view **258** Habitus, lateral view **259** Head and mesosoma, dorsal view **260** Head and mesosoma, lateral view **261** Head, anterior view **262** Head, lateral view. Scale bars in millimeters.

Figures 257–262 ; Morphbank ^58^

##### Description.

Female body length: 3.20–4.44 mm (n=12). Male body length: 3.80 mm (n=1). Color of scape in female: brown. Surface of dorsal head in female: covered throughout with very fine sculpture. Occipital carina in female: percurrent. Profile of posterior margin of head in lateral view: produced posteriorly, head appearing wedge-shaped. Width of genal setae: narrow to moderately wide. Shape of medial anteclypeus in female: narrow, trapezoidal, not strongly projected medially, apically slightly truncate to very weakly bilobed. Surface of mandible base in female: smooth. Form of mesosoma in female: typically formed, with propodeal shelf moderately elongate and clearly visible in dorsal view. Surface of pronotal nucha in female: sculptured throughout. Transverse pronotal carina in female: developed laterally, absent medially, not percurrent. Shape of mesoscutellum: semicircular to weakly transverse, evenly rounded posteriorly. Surface of propodeal nucha in female: sculptured throughout. Surface of propodeal shelf in female: sculptured throughout. Color of metasoma in female: brown, T5-T6 dark brown. Sculpture of T6: finely longitudinally striae.

##### Diagnosis.

This species is most similar to *Scelio ipomeae* which shares a similarly developed clypeus and anteclypeus and similar general habitus. *Scelio impostor* may be distinguished from *Scelio ipomeae* by the sparse, narrow pilosity of the gena (*Scelio ipomeae* with pilosity of the gena denser, setae thicker) and the brown metasoma (orange in *Scelio ipomeae*, but see Comments for that species). Individuals of *Scelio ipomeae* have the head somewhat wedge shaped in lateral view (Fig. 266), whereas those of *Scelio impostor* are typically more rounded (Fig. 262).

##### Etymology.

The epithet is used as a noun in apposition derived from the English, in reference to the similarity to *Scelio ipomeae*.

##### Link to distribution map.

http://hol.osu.edu/map-large.html?id=244754

##### Material examined.

*Holotype*, female: **SOUTH AFRICA**: Western Cape Prov., 25km N Prince Albert, damp wash, SA-018, Kat River Crossing, 33°05'39"S, 21°49'59"E, 500m, 3.X–24.X.2004, malaise trap, Irwin, Parker & Hauser, OSUC 250816 (deposited in SANC). *Paratypes*: **SOUTH AFRICA**: 12 females, 1 male, OSUC 211315, 212515, 250812–250815, 250817, 250972–250974 (CNCI); OSUC 213944 (OSUC); OSUC 222098, 234715 (SAMC).

#### Scelio ipomeae

Risbec

http://zoobank.org/BE3BD4CD-B4DD-496F-BF9F-EEE081D3DEF0

urn:lsid:biosci.ohio-state.edu:osuc_concepts:9739

http://species-id.net/wiki/Scelio_ipomeae

Figures 6 , 9 , 263–268 ; Morphbank ^59^

Scelio ipomeae Risbec, 1950: 589 (original description); Risbec 1954: 1038 (junior synonym of *Scelio mauritanicus* Risbec).

##### Description.

Female body length: 3.34–3.48 mm (n=2). Male body length: 2.77–4.50 mm (n=19). Color of scape in female: yellow. Surface of dorsal head in female: covered throughout with very fine sculpture. Occipital carina in female: percurrent. Profile of posterior margin of head in lateral view: evenly arcuate, head appearing lenticular. Width of genal setae: thick. Shape of medial anteclypeus in female: narrow, trapezoidal, not strongly projected medially, apically slightly truncate to very weakly bilobed. Surface of mandible base in female: smooth. Form of mesosoma in female: typically formed, with propodeal shelf moderately elongate and clearly visible in dorsal view. Surface of pronotal nucha in female: sculptured throughout. Transverse pronotal carina in female: developed laterally, absent medially, not percurrent. Shape of mesoscutellum: semicircular to weakly transverse, evenly rounded posteriorly. Surface of propodeal nucha in female: sculptured throughout. Surface of propodeal shelf in female: sculptured throughout. Color of metasoma in female: orange, T6 and/or T5 infuscate brown. Sculpture of T6: finely longitudinally striae.

##### Diagnosis.

This species is most similar to *Scelio aurantium* and *Scelio impostor* which share the medially interrupted transverse pronotal carina. *Scelio ipomeae* differs from *Scelio aurantium* by the complete occipital carina (vs. broadly obliterated) and the brown infuscation of T5 and T6 (vs. orange metasoma throughout). It differs from *Scelio impostor* by the dense patch of thicker white setae on the gena (vs. sparse thinner setae), wedge-shaped head in lateral view (vs. more rounded), and orange metasoma (vs. brown).

**Figures 263–268.**
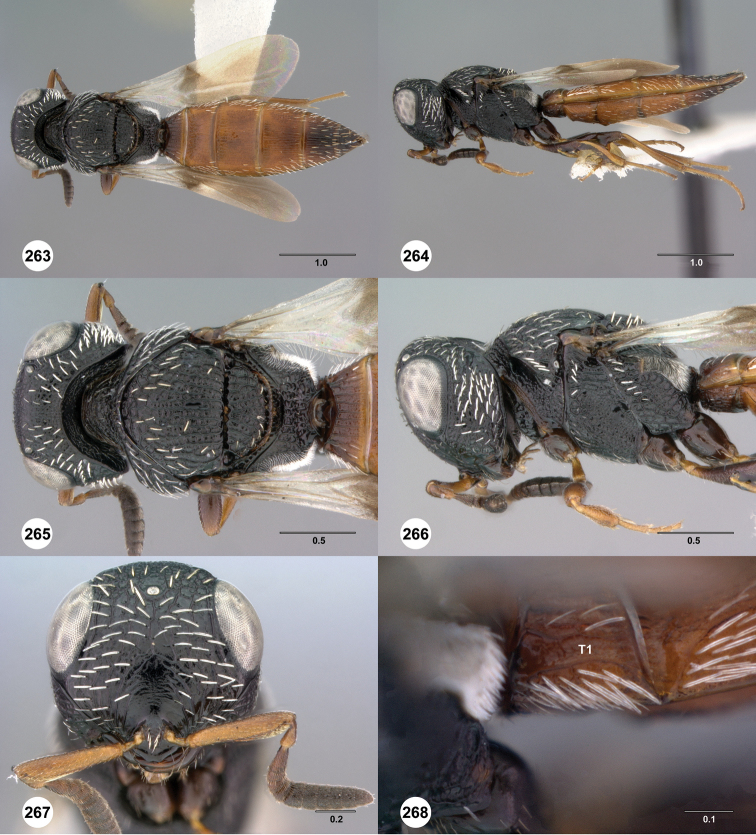
^158^ *Scelio ipomeae* Risbec., female (OSUC 213040). **263** Habitus, dorsal view **264** Habitus, lateral view **265** Head and mesosoma, dorsal view **266** Head and mesosoma, lateral view **267** Head, anterior view **268** Base of metasoma, lateral view. *T1*, first metasomal tergite. Scale bars in millimeters.

##### Link to distribution map.

http://hol.osu.edu/map-large.html?id=9739

##### Associations.

Solitary egg parasitoid of *Locusta migratoria* (Linnaeus) [Orthoptera: Acrididae].

##### Material examined.

*Lectotype* (**present designation**), female: **SENEGAL**: Diourbel Rég., sweet potato leaves, Bambey, 16.XI.1946, OSUC 254718 (deposited in MNHN). *Paralectotype*: **SENEGAL**: 1 female, OSUC 254719 (MNHN). *Other material*: (450 females, 215 males, 3 unknowns) **BENIN**: 2 females, 1 male, OSUC 211363–211364 (CNCI); OSUC 142669 (OSUC). **BOTSWANA**: 187 females, 117 males, OSUC 202824–202825 (AEIC); OSUC 211266, 213195 (CNCI); OSUC 160041–160042, 160045–160050, 160052 (EMEC); OSUC 254551 (MZLU); OSUC 164131, 164133, 164135–164136, 164138–164139, 164141–164144, 164146–164150, 164152–164164, 164167–164170, 164172–164175, 164177–164188, 164190–164192, 164194–164198, 164201–164204, 164206–164214, 164216–164232, 166262–166270, 166303–166322, 166324–166341, 166345–166346, 166350–166351, 166353, 166355–166356, 166358, 166361–166363, 166366–166367, 166370, 166372–166375, 166377–166378, 166381–166392, 211626, 211683, 212041–212061, 212064–212078, 212080–212084, 212089, 212093, 212096, 254728 (OSUC); OSUC 171009, 171116, 171119–171120, 171124–171127, 171142–171148, 171150–171157, 171160–171164, 171339, 171342, 171344–171345, 171347–171348, 171352, 171355, 171359–171361, 171364–171368, 207549, 207552–207553, 207555–207557, 207559–207574, 211805–211811, 211813, 211815, 211818, 211821, 244076 (USNM). **BURKINA FASO**: 4 females, 2 males, OSUC 213084–213085, 213090, 213092, 214092 (CNCI); OSUC 213387 (SANC). **ERITREA**: 1 female, OSUC 210359 (MCSN). **KENYA**: 6 females, 12 males, CASENT 2042603–2042610 (CASC); OSUC 212341, 212355, 214106–214107, 214111, 214118, 234633, 234642 (CNCI); OSUC 244094–244095 (USNM). **MALAWI**: 1 male, OSUC 212926 (CNCI). **MOZAMBIQUE**: 1 female, OSUC 213103 (CNCI). **NIGER**: 1 female, OSUC 251071 (TAMU). **NIGERIA**: 17 females, 2 males, OSUC 212196, 212730–212732, 213147, 213177, 250793, 250947–250949, 250969, 250989–250990, 250994, 251003–251007 (CNCI). **SENEGAL**: 1 female, OSUC 254720 (MNHN). **SOUTH AFRICA**: 215 females, 79 males, 3 unknowns, OSUC 202813 (AEIC); OSUC 211280, 211285, 211304, 211307, 211311, 211313, 211320–211321, 211324–211325, 211328, 211330, 211332–211337, 211339–211345, 211391–211392, 211396–211397, 211924–211927, 211929, 211931, 211933, 211936–211937, 211939–211940, 212221, 212223, 212240–212241, 212243–212245, 212249, 212254, 212263–212268, 212275–212281, 212283, 212285–212287, 212289, 212291–212293, 212297–212298, 212301–212305, 212307, 212314–212318, 212321–212322, 212324–212326, 212329–212336, 212368, 212383, 212385, 212387, 212389–212391, 212418, 212428, 212430–212431, 212437–212439, 212447, 212601, 212652, 212656, 212660–212664, 212666–212668, 212671, 212673, 212675–212682, 212745–212746, 212748, 212750–212752, 212754, 212759, 212765–212766, 212769–212770, 212772–212774, 212856–212857, 212863, 212865–212867, 212869, 212875, 212878–212881, 212883–212885, 212888–212889, 212913–212915, 212917–212918, 212922, 212927, 212978–212979, 212981–212982, 212984, 212986–212991, 212994–212999, 213114, 213129, 234667, 234669, 234674–234675, 250729 (CNCI); OSUC 250712 (MZLU); OSUC 142596, 142598, 142603–142604, 142607–142615, 171686–171687, 176244–176248, 211672–211673, 211685, 212388, 213609, 213623–213633, 222121–222127, 222131, 222135, 222303–222309, 234719, 234721–234723, 234725, 234728–234730, 244041–244045, 244202, 250735–250736, 250976, 250978, 250981 (OSUC); OSUC 213535, 213661 (SAMC); OSUC 213330, 213334, 213343, 213347, 213356, 213363, 213379, 213382, 213384, 213397, 213402–213403, 213415, 213435–213436, 213441, 213448, 213456–213459, 213471–213472, 213478, 213484, 213490, 214381 (SANC). **TANZANIA**: 1 female, 1 male, OSUC 212972, 250952 (CNCI). **YEMEN**: 2 females, OSUC 212492, 212948 (CNCI). **ZIMBABWE**: 12 females, OSUC 212102–212103, 212105, 212112, 212214, 212220, 212229, 212402, 212414, 213040, 213214, 213217 (CNCI).

##### Comments.

*Scelio ipomeae* is a widespread species and one of the most abundant in collections. The species exhibits relatively large variation in size, but large and small individuals are easily diagnosable. The S3 felt field is generally somewhat narrower than in other species. The coloration throughout (yellow scape, brown head and mesosoma, orange metasoma with brown T5–T6) is remarkably constant for all but the short series of atypical specimens discussed below. The sculpture of the dorsal head is often somewhat obliterated.

We include several atypical specimens in *Scelio ipomeae*. Three specimens from Yemen (OSUC 212948, 212341, 212492) have the metasoma yellow throughout but otherwise match well. They are all smaller than individuals of *Scelio aurantium*, which has a similarly colored metasoma, and have the occipital carina complete (vs. broadly obliterated). Two individuals from Kenya (OSUC 224094, 214118) have dark coloration as in *Scelio impostor* (brown metasoma), but the genal pilosity and head shape matches well with the core series. A third individual from Kenya (OSUC 244095) has a pictate fore wing, with a white band just past the apical extent of the stigmal vein. A smaller individual from Mozambique (OSUC 213103) has a more ovoid head and slightly sparser setae than typical.

#### Scelio ntchisii

Yoder sp. n.

http://zoobank.org/334BB00F-BE49-495D-A7B8-188090035B16

urn:lsid:biosci.ohio-state.edu:osuc_concepts:244751

http://species-id.net/wiki/Scelio_ntchisii

**Figures 269–274.**
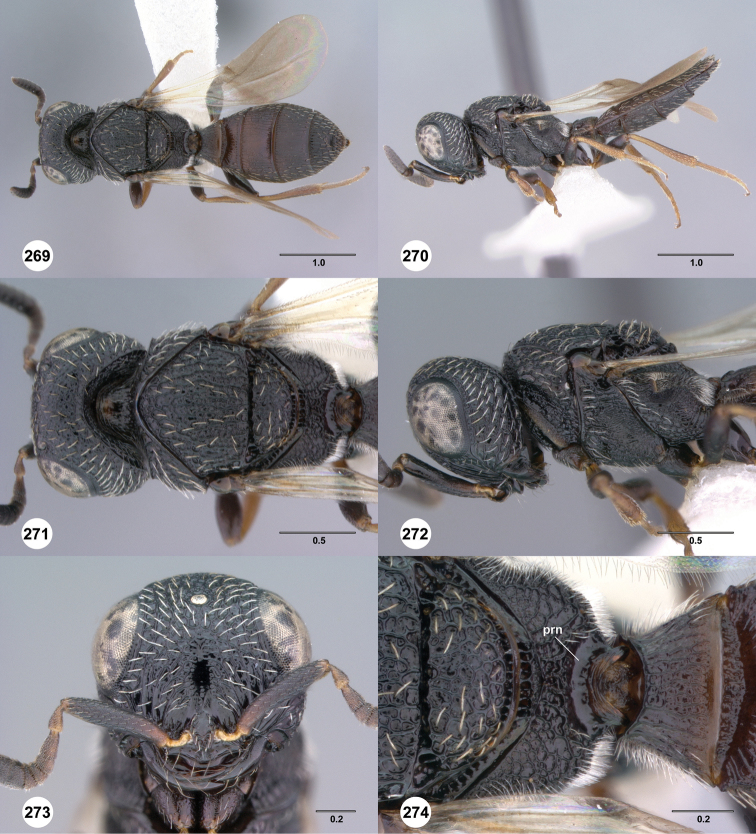
^159^ *Scelio ntchisii* sp. n., holotype female (OSUC 212835). **269** Habitus, dorsal view **270** Habitus, lateral view **271** Head and mesosoma, dorsal view **272** Head and mesosoma, lateral view **273** Head, anterior view **274** Propodeum, dorsal view. *prn*, propodeal nucha. Scale bars in millimeters.

Figures 269–274 ; Morphbank ^60^

##### Description.

Female body length: 4.40 mm (n=1). Color of scape in female: brown. Surface of dorsal head in female: covered throughout with very fine sculpture. Occipital carina in female: percurrent. Profile of posterior margin of head in lateral view: evenly arcuate, head appearing lenticular. Width of genal setae: narrow to moderately wide. Shape of medial anteclypeus in female: narrow, strip like, truncate apically. Surface of mandible base in female: with fine reticulate sculpture. Form of mesosoma in female: compact, with propodeal shelf short very strongly sloped and barely visible in dorsal view. Surface of pronotal nucha in female: with slight but prominent obliterated/smooth patch, otherwise sculptured throughout. Transverse pronotal carina in female: percurrent, not interrupted medially. Shape of mesoscutellum: semicircular to weakly transverse, evenly rounded posteriorly. Surface of propodeal nucha in female: with medial furrow, otherwise smooth. Surface of propodeal shelf in female: sculptured throughout. Color of metasoma in female: brown, T5-T6 dark brown. Sculpture of T6: coarsely rugose reticulate.

##### Diagnosis.

*Scelio ntchisii* is most similar to *Scelio somaliensis* with which it shares the similarly shaped, compact mesosoma and percurrent transverse pronotal carina. It differs from *Scelio somaliensis* by the coarse reticulate sculpture of T6 (longitudinal, fine in *Scelio somaliensis*) and the brown scape (yellow in *Scelio somaliensis*). *Scelio ntchisii* is the only species in the *ipomeae*-group to have the oxter more or less sculptured (reticulate) throughout.

##### Etymology.

The epithet is used as a genitive noun derived from the name of the type locality.

##### Link to distribution map.

http://hol.osu.edu/map-large.html?id=244751

##### Material examined.

*Holotype*, female: **MALAWI**: Ntchisi forest Reserve, 1500m, 3.XII–4.XII.1980, Lundt & Stuckenberg, OSUC 212835 (deposited in CNCI).

#### Scelio somaliensis

Yoder sp. n.

http://zoobank.org/FF4FD874-394B-4A1D-ACC7-C7DEA0677E91

urn:lsid:biosci.ohio-state.edu:osuc_concepts:244753

http://species-id.net/wiki/Scelio_somaliensis

**Figures 275–280.**
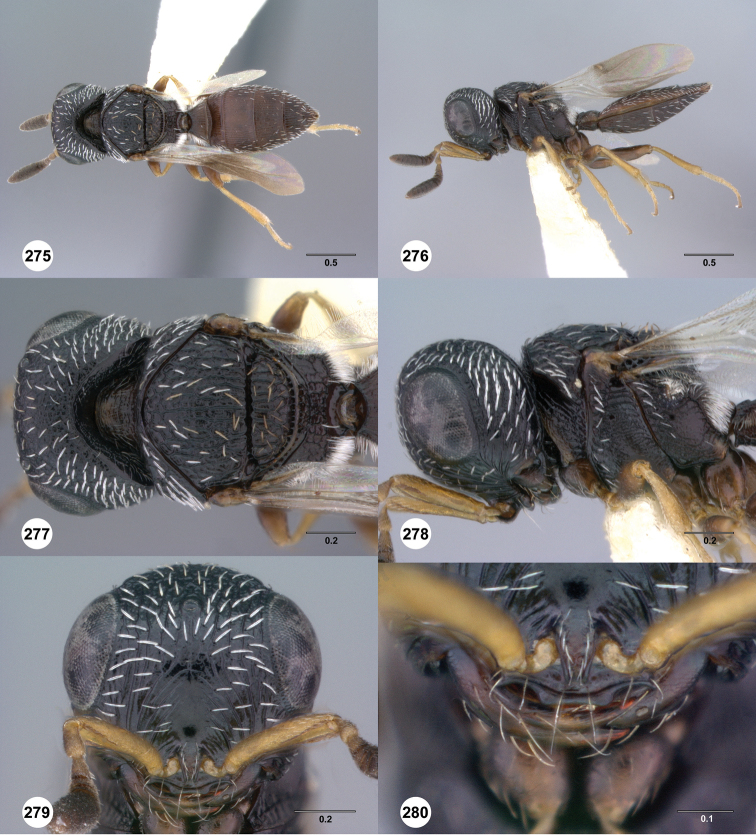
^160^ *Scelio somaliensis* sp. n., holotype female (OSUC 212608). **275** Habitus, dorsal view **276** Habitus, lateral view **277** Head and mesosoma, dorsal view **278** Head and mesosoma, lateral view **279** Head, anterior view **280** Mouthparts, anterior view. Scale bars in millimeters.

Figures 275–280 ; Morphbank ^61^

##### Description.

Female body length: 2.86 mm (n=1). Color of scape in female: yellow. Surface of dorsal head in female: covered throughout with very fine sculpture. Occipital carina in female: percurrent. Profile of posterior margin of head in lateral view: evenly arcuate, head appearing lenticular. Width of genal setae: thick. Shape of medial anteclypeus in female: narrow, strip like, truncate apically. Surface of mandible base in female: with fine reticulate sculpture. Form of mesosoma in female: compact, with propodeal shelf short very strongly sloped and barely visible in dorsal view. Surface of pronotal nucha in female: sculptured throughout. Transverse pronotal carina in female: percurrent, not interrupted medially. Shape of mesoscutellum: semicircular to weakly transverse, evenly rounded posteriorly. Surface of propodeal nucha in female: smooth throughout. Surface of propodeal shelf in female: sculptured throughout. Color of metasoma in female: brown. Sculpture of T6: finely longitudinally striae.

##### Diagnosis.

This species is most similar to *Scelio ntchisii* which shares the similarly shaped compact mesosoma and percurrent transverse pronotal carina. It differs in the fine longitudinal sculpture of T6 (coarsely reticulate in *Scelio ntchisii*) and the yellow scape (brown in *Scelio ntchisii*).

##### Etymology.

The epithet is used as an adjective derived from the country of the type locality.

##### Link to distribution map.

http://hol.osu.edu/map-large.html?id=244753

##### Material examined.

*Holotype*, female: **SOMALIA**: Mogadishu, Shabelle (Shabelli) Valley, Afgooye (Afgoi), 25.V–31.V.1979, malaise trap, F. Bin, OSUC 212608 (deposited in CNCI).

#### Scelio transtrum

Yoder sp. n.

http://zoobank.org/C0E42997-D071-4A11-B226-A6822FF1787C

urn:lsid:biosci.ohio-state.edu:osuc_concepts:244750

http://species-id.net/wiki/Scelio_transtrum

**Figures 281–286.**
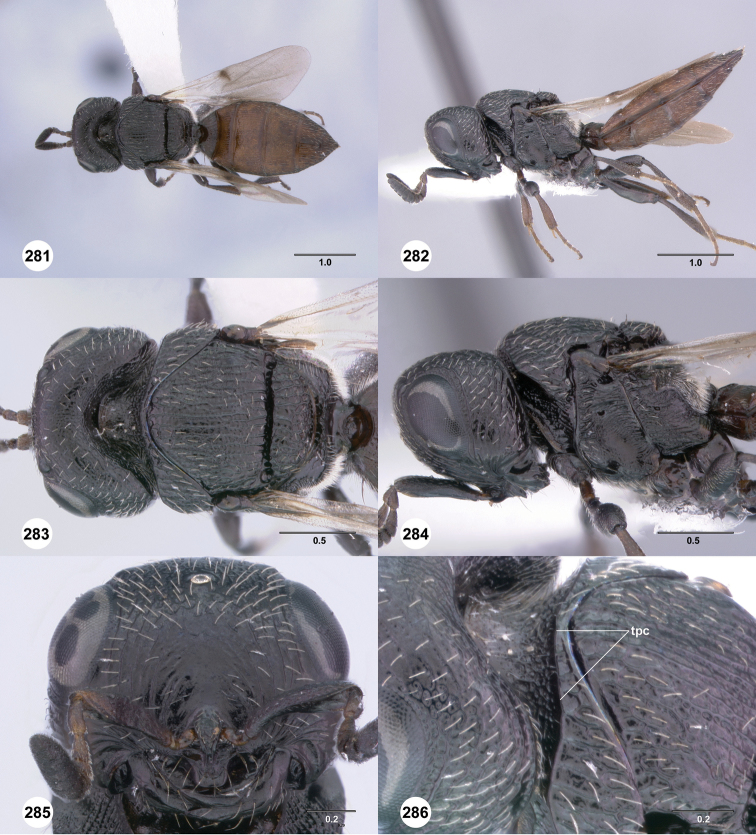
^161^ *Scelio transtrum* sp. n., holotype female (OSUC 212935). **281** Habitus, dorsal view **282** Habitus, lateral view **283** Head and mesosoma, dorsal view **284** Head and mesosoma, lateral view **285** Head, anterior view **286** Pronotum, oblique anterolateral view. *tpc*, transverse pronotal carina. Scale bars in millimeters.

Figures 281–286 ; Morphbank ^62^

##### Description.

Female Body length: 3.88–4.68 mm (n=10). Male Body length: 4.45–4.65 mm (n=2). Color of scape in female: brown. Surface of dorsal head in female: with obliterated patches between lateral ocelli, otherwise with somewhat flattened sculpture. Occipital carina in female: percurrent; broadly obliterated medially. Profile of posterior margin of head in lateral view: produced posteriorly, head appearing wedge-shaped. Width of genal setae: narrow to moderately wide. Shape of medial anteclypeus in female: thicker, with rounded corners, extended forward on strongly projected clypeus. Surface of mandible base in female: smooth. Form of mesosoma in female: compact, with propodeal shelf short very strongly sloped and barely visible in dorsal view. Surface of pronotal nucha in female: with slight but prominent obliterated/smooth patch, otherwise sculptured throughout. Transverse pronotal carina in female: percurrent, not interrupted medially. Shape of mesoscutellum: transverse, broadly depressed posteromedially, posterolateral margins slightly elevated. Surface of propodeal nucha in female: with medial furrow, otherwise smooth; smooth throughout. Surface of propodeal shelf in female: with obliterated to smooth patches laterally; sculptured throughout. Color of metasoma in female: orange throughout. Sculpture of T6: finely longitudinally striae.

##### Diagnosis.

*Scelio transtrum* is most similar to *Scelio ntchisii* and, to a slightly lesser degree, *Scelio somaliensis*, all sharing the presence of the percurrent transverse pronotal carina and the compact form of the mesosoma with strongly sloped medial propodeal shelf. *Scelio transtrum* differs from those two species by the strongly projected clypeus/anteclypeus (strip like and not projecting in both others), the transverse mesoscutellum that has slightly elevated posterolateral margins and medial depression (rounded, not particularly transverse, and otherwise unmodified in both others), and the orange metasoma (brown in both of the others). All but one individual (OSUC 213460 from South Africa) have obliterated or smoother patches on the anterolateral propodeal shelf. All other *ipomeae*-group species have the shelf sculptured throughout.

##### Etymology.

The epithet is used as a noun in apposition derived from the Latin word for crossbeam, in reference to the percurrent transverse pronotal carina.

##### Link to distribution map.

http://hol.osu.edu/map-large.html?id=244750

##### Material examined.

*Holotype*, female: **YEMEN**: #5046, Lahij (Lahj), VIII-2000, malaise trap, A. van Harten, OSUC 212935 (deposited in CNCI). *Paratypes*: (9 females, 2 males) **KENYA**: 2 females, CASENT 2042611 (CASC); OSUC 212505 (CNCI). **SOUTH** **AFRICA**: 1 female, OSUC 213460 (SANC). **TANZANIA**: 2 females, OSUC 212965, 212973 (CNCI). **YEMEN**: 4 females, 2 males, OSUC 212483, 212493, 212924, 212934, 212947 (CNCI); OSUC 212496 (OSUC).

##### Comments.

The occipital carina is generally present although it is obscured or absent for a short medial stretch in some. The gap is not as extensive as in *Scelio aurantium*. Two males are known and match the females well. The male has somewhat more robust sculpture which is particularly noticeable on the dorsal head and pronotal nucha. The male A5 is not as stalked basally and apically broad as in *Scelio ipomeae*.

### *Scelio irwini* species group

**Description.** *General*. Body size: small; moderate; large. Body length: 1.78–5.41 mm. Habitus: typical, mesosoma not dorsoventrally flattened. Body color: brown to dark brown. Fore leg color: concolorous with mid and hind legs. Sculpture: moderate to robust, reticulate to strigose, generally without longitudinal or parallel lineations. Wing type: macropterous.

*Pilosity*. General setation: predominantly very short, thin, and appressed. Thickened and truncate white pilosity: not typically present, or strongly truncate when so. Interommatidial pilosity: present. Genal pilosity density: sparse. Genal pilosity color: white; brown. Number of anteclypeal setal pairs: unknown. Ventrolateral postgenal cluster of erect setae: present. Antespiracular setal patch: moderately sized, intersected by posterior extension of lateral epomia. Netrion: glabrous. Propodeal shelf: present throughout except narrow medial strip. Pilosity of laterotergites: absent.

*Head*. Sculpture of head: variously sculptured, with well developed arcuate carinae of the lower frons, without smooth or obliterated patches, carinae generally moderate width. Ocelli size: moderate; large. Gap between antennal toruli and anteclypeus: narrow to moderate width. Width of ventral head across mandibles: moderate, mandibles typically formed. Anteclypeus shape between outer teeth: thin immediately mesad of outer teeth, smoothly rounded to slightly trapezoidal medially, without sharp vertices, medially truncate to slightly concave. Malar sulcus: present. Medial portion of occipital carina: percurrent. Lateral portion of occipital carina: more or less linear throughout. Form of gena: broad, somewhat flattened, minimally rounded between posterior margin of eye and occipital carina. Genal carina: absent. RSS on A5 in males: present. Microsculpture at base of mandible: absent. Basal tooth of mandible: absent.

*Mesosoma*. Shape of mesoscutum in lateral view: moderately concave, not particularly bulging in anterior nor flattened throughout. Transverse pronotal carina in female: unknown. Surface of mesoscutum: sculptured throughout, never with smooth or obliterated patches, reticulate laterally and anteromedially, longitudinally striate to strigose posteromedially. Smooth or obliterated patches on mesoscutum: absent. Surface of the pronotal collar in females: unknown. Axillula: small, clearly discernible only in lateral view. Propodeal corners: rounded, without pointed vertices. Epomia: present. Surface of oxter: often smooth or with sculpture obliterated or very faint. Fore wing length: reaching or surpassing anterior margin of T5 but not surpassing apex of metasoma. Fore wing submarginal vein near curve towards costal margin: unknown. Pictation of fore wing in female: absent.

*Metasoma*. Anterior margin of T1: concave, with short rim.

**Diagnosis.** Most similar to species of the *walkeri* and *ipomeae*-groups and, to a lesser degree, the *pulchripennis*-group. These all share the rounded posterior propodeal margin and medially bent villus. The *irwini*-group differs from these and all other species by the unique pilosity which is very short, narrow and sparse. The setae in the *walkeri* and *ipomeae*-groups is moderate to thick and prominent. Most individuals are further differentiated by the combination of the arcuate carinae of the lower frons, the broad, and somewhat flattened gena, the absence of a basal tooth on the mandible, and the flattened mesoscutum.

**Comments.** The *irwini*-group is a small and morphologically cohesive group of species restricted to Madagascar. While there are some possible affinities with the *walkeri* and *ipomeae*-groups ,this group appears to be relatively isolated from other species. Individuals usually have a slight projection on the posteromedial margin of the propodeum possibly homologous to the inner corner of the propodeal projection as seen in *howardi*-group species. The pattern of sculpture of the mesonotum is similar among all four species with slight differences in the amount of reticulation present (see Comments for individual species). The male genitalia of all species and a morph potentially representing a fifth species were surveyed (stereomicroscopy) for gross differences, and none were found.

**Key to *irwini*-group species** (also available online at http://www.waspweb.org/Platygastroidea/Keys/index.htm)

**Males and females**

**Table:** 

1	Mesopleural depression and metapleuron above hind coxa sculptured throughout (Fig. 290), without glabrous and smooth patch (dorsal head and upper frons with well-developed reticulations (Fig. 291); medial T2–T4 with only narrow smooth patches or sculptured throughout (Fig. 292)	*Scelio obscuripennis*
–	Mesopleural depression and metapleuron above hind coxa with a smooth and glabrous patch (e.g. Figs 297, 302, 310)	2
2	Upper frons dorsoventrally carinate (Fig. 303); medial T3, and to slightly lesser degree T2 and T4, with prominent smooth patches, patches often lighter color (Fig. 304)	*Scelio irwini*
–	Upper frons reticulate (Figs 297, 309); smooth patches of dorsal metasoma variously developed but typically smaller, and not lighter color	3
3	Arcuate sculpture of frons “open”: arching carinae terminating along the the malar sulcus (Fig. 297), arcuate sculpture near malar sulcus often obscured by reticulation in males; propodeum often with patch of obliterated to smooth sculpture anterolaterally, and usually with several prominent sinuate striae (Fig. 298); male RSS linear, finely carinate	*Scelio harinhalai*
–	Arcuate sculpture of frons “closed”, arching carinae terminating at or very near the base of the mandible (Fig. 309), arcuate sculpture not obscured by reticulation in males; propodeum more or less uniformly reticulate throughout, without smooth patches (Fig. 307); male RSS node-like	*Scelio parkeri*

**Figures 287–292.**
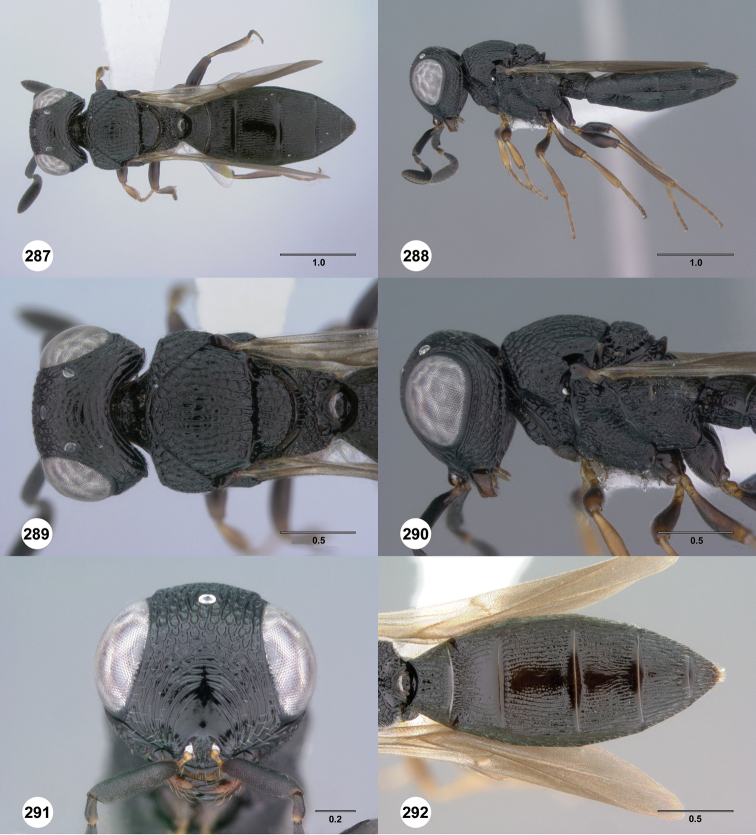
^162^ *Scelio obscuripennis* sp. n., **287–291** female (CASENT 8106402), 292 (OSUC 212529). **287** Habitus, dorsal view **288** Habitus, lateral view **289** Head and mesosoma, dorsal view **290** Head and mesosoma, lateral view **291** Head, anterior view **292** Metasoma, dorsal. Scale bars in millimeters.

#### Scelio harinhalai

Yoder sp. n.

http://zoobank.org/9BAE589D-BFD1-4E13-B4E7-FA98CDDEA477

urn:lsid:biosci.ohio-state.edu:osuc_concepts:244777

http://species-id.net/wiki/Scelio_harinhalai

**Figures 293–298.**
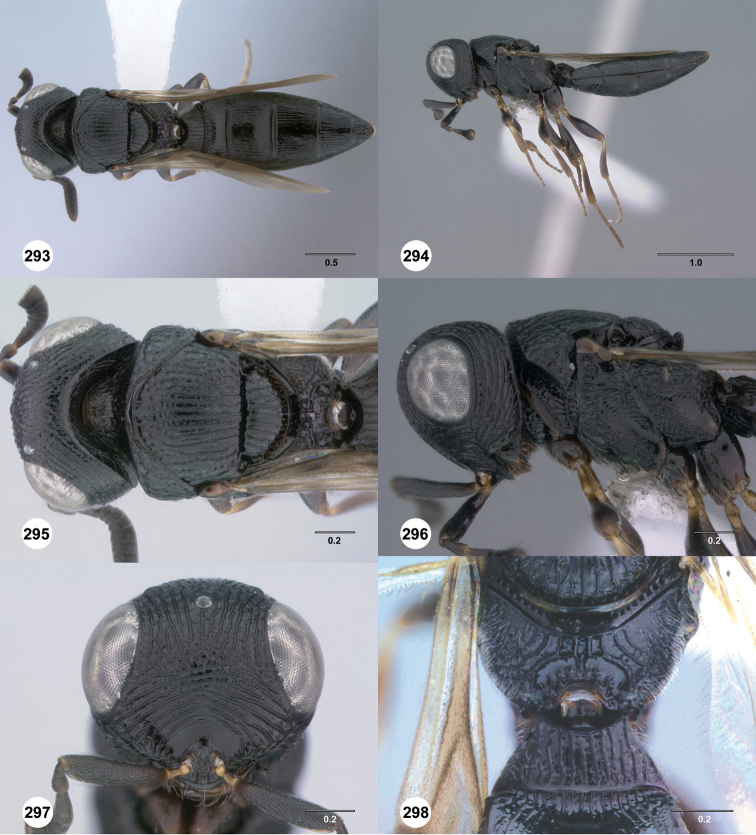
^163^ *Scelio harinhalai* sp. n. **293–297** holotype female (CASENT 2132273), 298 paratype female (CASENT 8106088). **293** Habitus, dorsal view **294** Habitus, lateral view **295** Head and mesosoma, dorsal view **296** Head and mesosoma, lateral view **297** Head, anterior view **298** Propodeum, dorsal view. Scale bars in millimeters.

Figures 293–298 ; Morphbank ^63^

##### Description.

Female body length: 3.32–4.00 mm (n=20). Male body length: 3.32–3.92 mm (n=20). Sculpture of posterior vertex: weakly reticulate. Sculpture of frons in female: reticulate throughout; reticulate in upper 3/4. Arcuate carinae of lower frons: “open”, arcuate carina terminating along malar sulcus. Form of RSS on A5 in male: carinate. Sculpture of medial mesonotum: longitudinally striate in posterior half, otherwise reticulate. Surface of propodeal shelf in females: anterolaterally with small smooth patch, otherwise with slightly irregular curved sinuate carinae. Surface of meso- and metapleural depressions: sculptured throughout. Fore wing length: shorter than apex of metasoma.

##### Diagnosis.

This species is most similar to *Scelio parkeri* with which it shares the combination of smooth patches on the meso- and metapleuron, a tendency for the mesoscutellum to be longitudinally striate, and reticulate sculpture of the posterior head. It differs from *Scelio parkeri* by the “closed” arcuate sculpture of the frons, with arcs terminating near base of mandible, not along the malar sulcus.

##### Etymology.

The epithet is used as a genitive noun derived from the name of one of the three collectors who together amassed hundreds of specimens of the *irwini*-group.

##### Link to distribution map.

http://hol.osu.edu/map-large.html?id=244777

##### Material examined.

*Holotype*, female: **MADAGASCAR**: Toliara Auto. Prov., 8km NW Amboasary, gallery forest, MA-02-22-10, Berenty Private Reserve, 25°00.40'S, 46°18.20'E, 85m, 16.XII–27.XII.2002, malaise trap, M. Irwin, F. Parker & R. Harin’Hala, CASENT 2132273 (deposited in CASC). *Paratypes*: **MADAGASCAR**: 141 females, 889 males, 2 unknowns, CASENT 2042048, 2042057, 2042118, 2042125–2042126, 2042293, 2042962, 2043062, 2043153, 2043271, 2043273, 2043407, 2043624, 2043911, 2043922, 2132024, 2132088, 2132113–2132114, 2132117, 2132120–2132122, 2132125–2132126, 2132131–2132133, 2132137, 2132141–2132142, 2132145–2132146, 2132148–2132149, 2132153, 2132156, 2132160–2132163, 2132165–2132169, 2132173, 2132177, 2132182–2132183, 2132185–2132187, 2132189–2132192, 2132197, 2132201–2132204, 2132210, 2132213, 2132218, 2132220, 2132222–2132224, 2132228–2132230, 2132237, 2132239–2132241, 2132247, 2132249–2132252, 2132255, 2132261, 2132265, 2132271, 2132275–2132277, 2132284, 2132287–2132288, 2132292–2132294, 2132301, 2132306, 2132308–2132309, 2132311, 2132316, 2132319, 2132333, 2132337, 2132342, 2132344, 2132349, 2132355–2132357, 2132362–2132364, 2132366–2132368, 2132370, 2132372, 2132374, 2132376–2132377, 2132379, 2132385–2132386, 2132388–2132395, 2132399, 2132405, 2132415, 2132572–2132576, 2132580, 2132584, 2132591, 2132597, 2132600–2132601, 2132603–2132605, 2132607, 2132610–2132611, 2132614, 2132616, 2132619–2132621, 2132626–2132628, 2132630, 2132633, 2132637–2132639, 2132653, 2132663, 2132665–2132671, 2132673, 2132677–2132678, 2132683, 2132689–2132691, 2132695–2132699, 2132704–2132705, 2132771, 2132782, 2132801, 2132807, 2132815, 2132852–2132859, 2132861–2132862, 2132867, 2132870–2132871, 2132873, 2132875–2132882, 2132885–2132886, 2132888–2132890, 2132893, 2132895–2132896, 2132899, 2132901, 2132903–2132907, 2132909–2132911, 2132914, 2132916–2132917, 2132919–2132921, 2132923, 2132925, 2132927, 2132930–2132933, 2132937–2132938, 2132940–2132942, 2132944, 2132947, 2132949–2132950, 2132952–2132954, 2132962, 2132964, 2132966, 2132970, 2132979–2132980, 2132985–2132986, 2132988–2132989, 2133079, 2133082, 2133090, 2133095, 2133100, 2133105–2133106, 2133115–2133116, 2133149, 2133161, 2133168, 2133184, 2133250, 2133302, 2133329, 2133332, 2133338, 2133342, 2133344, 2133346, 2133359, 2133430, 2133503, 2133527–2133528, 2133530, 2133538, 2133550, 2133553, 2133555–2133557, 2133560, 2133563, 2133567, 2133569–2133570, 2133572, 2133574–2133576, 2133578, 2133585, 2133591, 2133593–2133594, 2133597–2133598, 2133600, 2133607, 2133610–2133612, 2133616–2133617, 2133619, 2133621–2133623, 2133632, 2133635, 2133637–2133639, 2133642, 2133644, 2133646, 2133648–2133651, 2133653, 2133657–2133659, 2133663, 2133669, 2133671, 2133674, 2133677, 2133683, 2133686, 2133688–2133689, 2133691, 2133695, 2133698, 2133700, 2133705, 2133708, 2133714–2133715, 2133717, 2133721–2133722, 2133724, 2133726, 2133729–2133730, 2133733, 2133735–2133736, 2133739–2133740, 2133744, 2133746–2133747, 2133749–2133752, 2133756, 2133758, 2133763, 2133768, 2133770, 2133772, 2133775, 2133778–2133780, 2133786–2133789, 2133792, 2133794, 2133801, 2133804, 2133825–2133826, 2133830, 2133835, 2133838, 2133842–2133844, 2133849, 2133851, 2133855–2133856, 2133860, 2133864, 2133871–2133872, 2133877–2133878, 2133938, 2133942, 2133953, 2133956, 2133958–2133959, 2133962, 2133966–2133967, 2133969, 2133971–2133972, 2133974, 2133978, 2133981–2133982, 2133986–2133987, 2133989, 2133992–2133994, 2133998, 2134012–2134016, 2134018–2134019, 2134021–2134022, 2134024–2134025, 2134027–2134031, 2134034, 2134038–2134039, 2134042–2134046, 2134048, 2134051–2134052, 2134116, 2134216, 2134253, 2134264, 2134268–2134269, 2134272, 2134275, 2134277, 2134301, 2134310–2134313, 2134315–2134316, 2134318, 2134322–2134324, 2134326, 2134329–2134331, 2134333, 2134335–2134336, 2134338–2134341, 2134343, 2134346–2134350, 2134352, 2134355, 2134357–2134367, 2134370–2134373, 2134377–2134384, 2134387–2134391, 2134393–2134400, 2134402–2134404, 2134444–2134445, 2134448–2134450, 2134521–2134522, 2134585–2134586, 2134629, 2134632, 2134639–2134640, 2134650, 2134654–2134655, 2134691, 2134769, 2134843, 2134847, 2134853, 2134878, 2134883, 2134893, 2135052–2135053, 2135296, 2135920, 2135933, 2135937, 2135981–2135982, 8097491, 8106051, 8106053, 8106060, 8106066–8106068, 8106090–8106091, 8106097, 8106119, 8106305, 8106673, 8106675–8106676, 8106679, 8106682–8106683, 8106686, 8106688–8106689, 8106694, 8106697, 8106700, 8106707, 8106709, 8106712, 8106714, 8106720–8106721, 8106724, 8106729, 8106731, 8106735–8106742, 8106744, 8106746–8106747, 8106759–8106760, 8106762, 8106764, 8106766–8106768, 8106770–8106772, 8106776–8106779, 8106784, 8106787, 8106790, 8106803, 8106805, 8106811, 8106813, 8106815, 8106820, 8106822, 8106825–8106826, 8106829, 8106831–8106832, 8106854, 8106858, 8106860, 8106862–8106863, 8106865, 8106867–8106870, 8106875, 8106878–8106879, 8106881, 8106887–8106890, 8106895, 8106898–8106899, 8106977, 8106993 (CASC); CASENT 2042007, 2042043, 2042045, 2042055, 2042107, 2042115, 2042120–2042122, 2042127–2042128, 2042250–2042251, 2042273, 2042670–2042671, 2042674, 2043069, 2043073, 2043075–2043078, 2043080, 2043082–2043083, 2043085–2043087, 2043089, 2043092–2043094, 2043097–2043098, 2043156, 2043201, 2043252, 2043272, 2043274, 2043298, 2043300, 2043349, 2043401, 2043405, 2043408, 2043410–2043412, 2043418, 2043453, 2043548–2043549, 2043615, 2043856, 2043913, 2043916, 2043921, 2043927–2043928, 2132096, 2132130, 2132140, 2132176, 2132178, 2132209, 2132226, 2132234, 2132267, 2132279, 2132281, 2132283, 2132291, 2132346–2132348, 2132783, 2132908, 2133072, 2133080–2133081, 2133089, 2133091, 2133093–2133094, 2133098–2133099, 2133101–2133102, 2133111–2133114, 2133117, 2133120–2133122, 2133160, 2133180, 2133189, 2133193, 2133236, 2133248–2133249, 2133254, 2133313–2133314, 2133318, 2133321, 2133323–2133324, 2133326, 2133331, 2133335, 2133337, 2133343, 2133345, 2133357, 2133363, 2133929, 2133951, 2134026, 2134406, 2134524, 2134604, 2134630–2134631, 2134633, 2134649, 2134661, 2134858, 2134888, 2135930, 8097460–8097461, 8097465, 8097468, 8097473, 8097477–8097479, 8097482–8097483, 8097486–8097487, 8097489–8097490, 8097493–8097494, 8106047, 8106049, 8106055, 8106057, 8106063–8106064, 8106072, 8106075, 8106077–8106078, 8106081–8106089, 8106092, 8106094–8106096, 8106098, 8106100–8106101, 8106103, 8106106–8106108, 8106110, 8106112, 8106115–8106116, 8106118, 8106120, 8106122, 8106124–8106130, 8106133–8106139, 8106215–8106216, 8106218–8106220, 8106223–8106224, 8106229, 8106231–8106232, 8106234–8106236, 8106239, 8106246, 8106252–8106253, 8106255–8106256, 8106264–8106265, 8106268–8106269, 8106282–8106283, 8106291–8106293, 8106298, 8106302–8106303, 8106307, 8106309–8106310, 8106312–8106313, 8106316–8106317, 8106321, 8106326–8106327, 8106329–8106331, 8106333–8106339, 8106344, 8106346, 8106351–8106353, 8106359, 8106362–8106364, 8106367–8106373, 8106376–8106379, 8106389, 8106394–8106395, 8106451, 8106506, 8106524, 8106527, 8106536, 8106538, 8106543, 8106547, 8106553–8106554, 8106566–8106567, 8106571–8106574, 8106576–8106580, 8106583, 8106591, 8106594–8106596, 8106599, 8106603, 8106608–8106609, 8106611–8106619, 8106621–8106624, 8106626, 8106629, 8106632–8106634, 8106639, 8106641, 8106643, 8106645–8106647, 8106653, 8106655, 8106658, 8106660, 8106662, 8106666–8106668, 8106670, 8106674, 8106677, 8106708, 8106749–8106750, 8106752–8106753, 8106757, 8106763, 8106824, 8106836–8106839, 8106841–8106842, 8106846–8106848, 8106850–8106851, 8106872, 8106876, 8106884 (OSUC).

##### Comments.

*Scelio harinhalai* has the smallest smooth patches on the meso- and metapleuron among species who share this character (*Scelio irwini* has the largest). The arcuate carinae of the face are somewhat hidden due to the surrounding reticulate elements that are present more or less throughout. See also *Scelio parkeri*. The mesonotal sculpture medially tends to be more reticulate than longitudinal.

#### Scelio irwini

Yoder sp. n.

http://zoobank.org/6DEF9531-C244-447F-BD3C-4F0944EAEAD7

urn:lsid:biosci.ohio-state.edu:osuc_concepts:244778

http://species-id.net/wiki/Scelio_irwini

**Figures 299–304.**
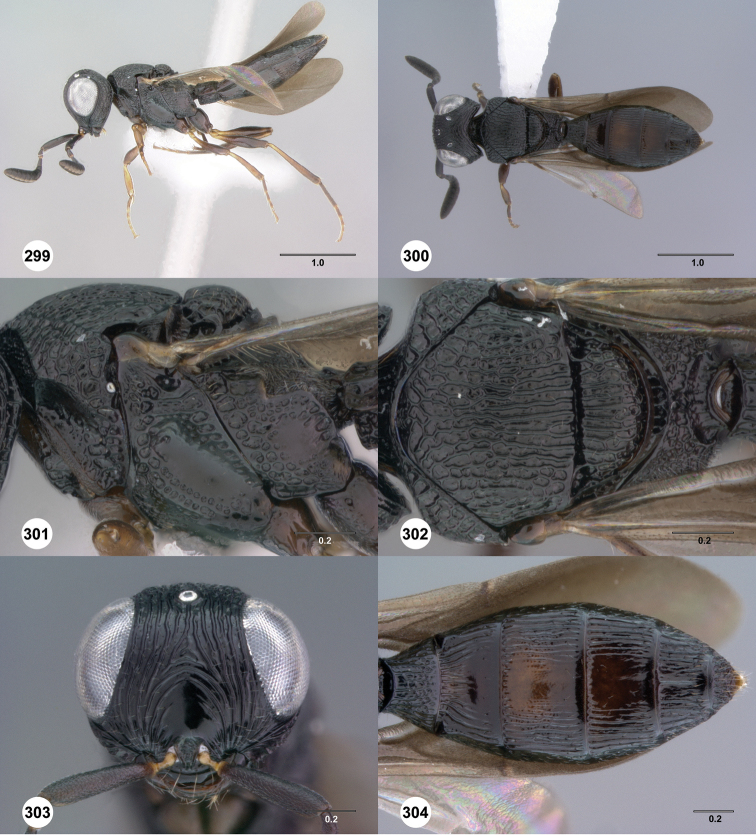
^164^ *Scelio irwini* sp. n., holotype female (CASENT 2042009). **299** Habitus, dorsal view **300** Habitus, lateral view **301** Mesosoma, dorsal view **302** Mesosoma, lateral view **303** Head, anterior view **304** Metasoma, dorsal view. Scale bars in millimeters.

Figures 299–304 ; Morphbank ^64^

##### Description.

Female body length: 3.23–3.99 mm (n=19). Male body length: 3.74–4.32 mm (n=2). Sculpture of posterior vertex: transversely carinate. Sculpture of frons in female: linear throughout. Arcuate carinae of lower frons: “closed”, arcuate carinae terminating at ventrolateral corner of anteclypeus. Form of RSS on A5 in male: carinate. Sculpture of medial mesonotum: longitudinally striate in posterior half, otherwise reticulate. Surface of propodeal shelf in females: uniformly reticulate throughout. Surface of meso- and metapleural depressions: with prominent glabrous and smooth patch. Fore wing length: longer than apex of metasoma.

##### Diagnosis.

*Scelio irwini* is most similar to *Scelio parkeri* with which it shares the smooth patches on the meso- and metapleural depressions. It differs from this and all other *irwini*-group species by the dorsoventrally carinate to very slightly strigose sculpture of the frons, and unbroken transverse carinae on the posterior head (with at least some prominent reticulate areas outside the ocellar triangle in all others).

##### Etymology.

The epithet is used as a genitive noun derived from the name of our colleague Mike Irwin, one of the three collectors who together amassed hundreds of specimens of the *irwini*-group.

##### Link to distribution map.

http://hol.osu.edu/map-large.html?id=244778

##### Material examined.

*Holotype*, female: **MADAGASCAR**: Antsiranana Auto. Prov., Montagne d’Ambre National Park, 12°30'52"S, 49°10'53"E, 960m, 21.I–26.I.2001, malaise trap, M. E. Irwin, E. I. Schlinger & R. Harin’Hala, CASENT 2042009 (deposited in CASC). *Paratypes*: **MADAGASCAR**: 35 females, 4 males, OSUC 211647 (AMNH); CASENT 2042003–2042004, 2042006, 2042011–2042015, 2042017–2042021, 2042023–2042026, 2042030, 2042032–2042033, 2042038–2042041, 2042056, 2042106, 2043318, 2043433, 2132030, 2134300, 2134572, 2135604, 2136585 (CASC); CASENT 2042008, 2042022, 2042027, 2042029, 2042031 (OSUC).

##### Comments.

The sculpture within the ocellar triangular can be irregularly reticulate in some individuals. The male RSS of A5 is intermediate in form between *Scelio parkeri* and *Scelio obscuripennis*, projecting slightly like the former, but retaining a bit more of a distinct carina like the latter. The medial mesonotal sculpture tends to be longitudinally striate strigose.

#### Scelio obscuripennis

Johnson nom. nov.

http://zoobank.org/C2FA67A3-148A-4B6D-BCC8-DD9CD741D524

urn:lsid:biosci.ohio-state.edu:osuc_concepts:4728

http://species-id.net/wiki/Scelio_obscuripennis

Figures 287–292 ; Morphbank ^65^

Lepidoscelio fuscipennis Kieffer, 1905: 130 (original description); Kieffer 1926: 348 (description); Masner 1976: 16 (type information).
Scelio obscuripennis http://zoobank.org/F9066C7F-A437-4FD2-85EF-B2A2DEE21AD2

##### Description.

Female body length: 3.08–5.00 mm (n=39). Male body length: 2.74–4.58 mm (n=33). Sculpture of posterior vertex: transversely carinate. Sculpture of frons in female: reticulate in upper half. Arcuate carinae of lower frons: “closed”, arcuate carinae terminating at ventrolateral corner of anteclypeus. Form of RSS on A5 in male: carinate. Sculpture of medial mesonotum: reticulate throughout; longitudinally striate in posterior half, otherwise reticulate. Surface of propodeal shelf in females: uniformly reticulate throughout. Surface of meso- and metapleural depressions: sculptured throughout. Fore wing length: shorter than apex of metasoma; meeting apex of metasoma.

##### Diagnosis.

*Scelio obscuripennis* may be distinguished from all other species of the *irwini*-group by the completely sculptured mesopleural and metapleural depressions (all other *irwini*-group species with a smooth patch).

##### Link to distribution map.

http://hol.osu.edu/map-large.html?id=4728

##### Material examined.

*Holotype*, female: **MADAGASCAR**: Antsiranana Auto. Prov., Antsiranana (Diégo-Suarez), 1893, C. Alluaud (deposited in MNHN). *Other material*: **MADAGASCAR**: 149 females, 139 males, CASENT 2042010, 2042028, 2042042, 2042044, 2042046, 2042050–2042051, 2042053, 2042108, 2042116–2042117, 2042123–2042124, 2042256, 2042263, 2042265, 2042703–2042710, 2042712–2042713, 2042715, 2042978–2042980, 2042982, 2043066, 2043251, 2043304, 2043332, 2043348, 2043394–2043395, 2043398–2043400, 2043403, 2043419, 2043422, 2043424–2043425, 2043466, 2043569, 2043571, 2043589, 2043616, 2043630, 2043959, 2132015, 2132022, 2132118, 2132151, 2132553, 2132565, 2132710, 2132712, 2132784, 2133069, 2133075, 2133128, 2133134, 2133138, 2133190, 2133211, 2133330, 2133341, 2133423, 2133923, 2133957, 2134127–2134134, 2134221, 2134229–2134230, 2134243, 2134246–2134248, 2134254, 2134263, 2134274, 2134530, 2134569, 2134578, 2134851, 2134869, 2134884, 2135113, 2135115–2135117, 2135120–2135121, 2135123, 2135125, 2135683, 2135924, 8106193–8106208, 8106210–8106213, 8106276–8106278, 8106286–8106287, 8106296, 8106348–8106349, 8106392–8106393, 8106400–8106401, 8106403–8106406, 8106408, 8106410–8106414, 8106417–8106418, 8106420–8106427, 8106430–8106439, 8106441, 8106505, 8106516, 8106522, 8106563, 8106631, 8106986–8106987, 8106997 (CASC); OSUC 212529, 212534, 212537 (CNCI); CASENT 2042005, 2042016, 2042047, 2042049, 2042058, 2042104–2042105, 2042111, 2042114, 2042130, 2042266, 2042277, 2042626–2042627, 2042711, 2042714, 2043001, 2043056, 2043165, 2043254, 2043280, 2043282, 2043285, 2043345, 2043420–2043421, 2043426, 2043493–2043495, 2043574, 2043617, 2118395, 2132100–2132103, 2132402, 2132418, 2132554–2132557, 2132566, 2132762–2132763, 2132792, 2132830, 2133104, 2133127, 2133130, 2133167, 2133201, 2133212, 2133245, 2133276, 2133278, 2133291–2133292, 2133294, 2133466, 2133496, 2133926–2133928, 2134144, 2134597, 2134656, 2134692, 2134697, 2134787, 2134816, 2134850, 2134866–2134867, 2134874, 2135109, 2135114, 2135118–2135119, 2135122, 2135124, 2135126–2135127, 2135143, 2135912, 2135914, 2135916, 2135918, 2135926, 8106209, 8106280, 8106284, 8106315, 8106386, 8106402, 8106407, 8106409, 8106415, 8106419, 8106428–8106429, 8106440, 8106593, 8106817, OSUC 212038 (OSUC).

##### Comments.

Kieffer created the genus *Lepidoscelio* for *Scelio*-like species in which the metascutellum (which he referred to as the postscutellum in the original French description and later as the Metanotum in German in Das Tierreich) is upright and bilobed. This circumscription includes quite a number of species from the Old and New World. There is no additional evidence that these species are each others closest relatives. In fact, a number of apparently unrelated Neotropical species have such a metascutellum. We conclude that based solely on this character state, the genus *Lepidoscelio* is polyphyletic. Further, recognition of the concept in a more narrow sense to include only the *irwini*-group species would render *Scelio* paraphyletic. Therefore, we propose that *Lepidoscelio* is a junior synonym of *Scelio* (new synonymy). Transfer of the type species into *Scelio* necessitates a change in species name. The name *Scelio fuscipennis* is preoccupied, having been used by Ashmead (1887) for a Nearctic species. Therefore, we propose the name *Scelio obscuripennis* as a replacement name. Other than the type species, four other species are currently classified in *Lepidoscelio*. These are here formally transferred to *Scelio* (type material examined): *Scelio cayennensis* (Risbec), comb. n., *Scelio insularis* Ashmead, comb. r., *Scelio luteus* (Cameron), comb. n., and *Scelio thoracicus* Ashmead, comb. r.

*Scelio obscuripennis* is the most size variable and phenotypically plastic in the *irwini* species group. The sculpture of the mesonotum varies, though in most (particularly larger individuals) there is a stronger trend to more reticulate and less longitudinal sculpture medially. A series of individuals presently included, but excluded from the type series, were at one point considered to represent a separate species, however, given the general variation in size and the associated differences that accompany these differences we have elected to describe one rather polymorphic species. The second series (e.g. CASENT 2042111, 2042266, 2043254, 2043574, 2132100, 2132101) is composed of small individuals that have the submarginal vein obliterated prior to reaching the costal margin. Males in this series (e.g. CASENT 2043056, 2043494, 2042114, 8106280, 2132402, 2043282) have somewhat transverse flagellomeres following A5, and stockier legs. Most individuals in this series have a whitish stigma and broader, more compact habitus. These characters may be associated with allometric differences in size.

#### Scelio parkeri

Yoder sp. n.

http://zoobank.org/66056236-643B-44BC-A718-13C67E1CA30F

urn:lsid:biosci.ohio-state.edu:osuc_concepts:244776

http://species-id.net/wiki/Scelio_parkeri

**Figures 305–310.**
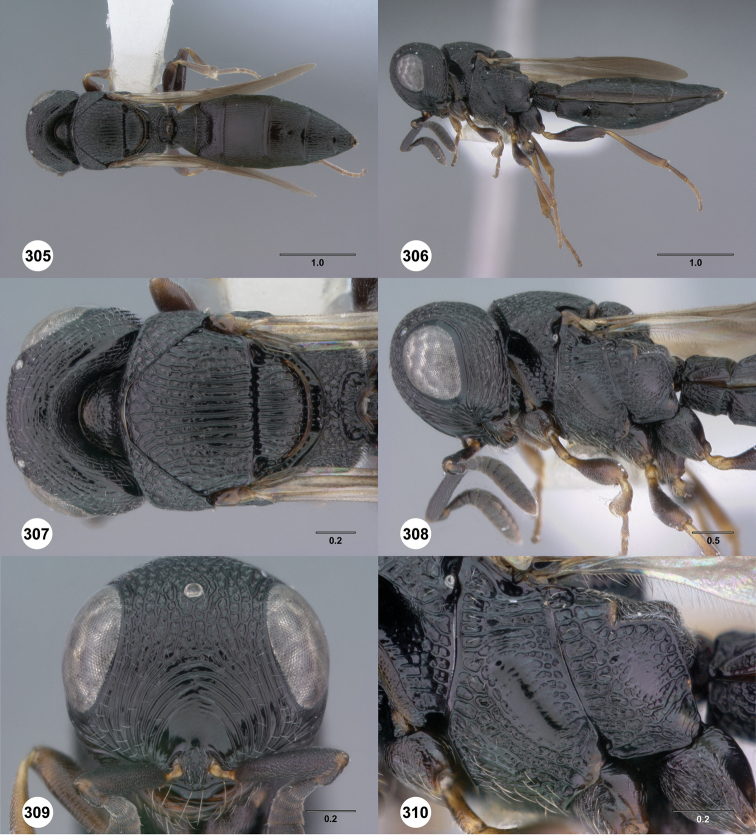
^165^ *Scelio parkeri* sp. n., holotype female (CASENT 2042035). **305** Habitus, dorsal view **306** Habitus, lateral view **307** Head and mesosoma, dorsal view **308** Head and mesosoma, lateral view **309** Head, anterior view **310** Mesopleuron and metapleuron, lateral view. Scale bars in millimeters.

Figures 11 , 305–310 ; Morphbank ^66^

##### Description.

Female body length: 1.78–5.41 mm (n=19). Male body length: 3.76–4.73 mm (n=20). Sculpture of posterior vertex: weakly reticulate. Sculpture of frons in female: reticulate in upper half. Arcuate carinae of lower frons: “closed”, arcuate carinae terminating at ventrolateral corner of anteclypeus. Form of RSS on A5 in male: node like and somewhat pointed. Sculpture of medial mesonotum: longitudinally striate in posterior half, otherwise reticulate. Surface of propodeal shelf in females: uniformly reticulate throughout. Surface of meso- and metapleural depressions: with prominent glabrous and smooth patch. Fore wing length: shorter than apex of metasoma.

##### Diagnosis.

*Scelio parkeri* is most similar to *Scelio harinhalai* which shares the smooth patches on the meso- and metapleural depressions. It differs from *Scelio harinhalai* by the “closed” arcuate sculpture of the frons, with arcs terminating near base of mandible, not along the malar sulcus. The ventralmost extension of the arcuate sculpture is never confused with reticulations in *Scelio parkeri* as it is in *Scelio harinhalai*. Males are unique among *irwini*-group species for the nodelike RSS (carinate in all other *irwini*-group species).

##### Etymology.

The epithet is used as a genitive noun derived from the name of Frank Parker, one of the three collectors who together amassed hundreds of specimens of the *irwini*-group.

##### Link to distribution map.

http://hol.osu.edu/map-large.html?id=244776

##### Material examined.

*Holotype*, female: **MADAGASCAR**: Toliara Auto. Prov., nr. research station, parcel I, dry deciduous forest, MA-02-14A-04, Bezaha Mahafaly Special Reserve, 23°41.19'S, 44°35.46'E, 165m, 28.XI–4.XII.2001, malaise trap, Harin’Hala, CASENT 2042035 (deposited in CASC). *Paratypes*: **MADAGASCAR**: 164 females, 317 males, CASENT 2042034, 2042036–2042037, 2042054, 2042109–2042110, 2042119, 2042292, 2042673, 2042675, 2042981, 2043079, 2043084, 2043090–2043091, 2043095–2043096, 2043099, 2043269, 2043305, 2043312, 2043343, 2043377, 2043402, 2043406, 2043413, 2043416, 2043430, 2043454, 2043619–2043623, 2043625, 2043633, 2043915, 2043917–2043919, 2132002, 2132013, 2132115–2132116, 2132127, 2132129, 2132195–2132196, 2132219, 2132225, 2132227, 2132238, 2132245, 2132257–2132259, 2132270, 2132278, 2132280, 2132296, 2132318, 2132321, 2132323, 2132325, 2132334, 2132341, 2132378, 2132382, 2132450, 2132587, 2132651, 2132672, 2132674, 2132680, 2132686, 2132806, 2132883–2132884, 2132887, 2132912–2132913, 2132918, 2132936, 2132959, 2132969, 2133070, 2133076–2133078, 2133088, 2133118, 2133140, 2133148, 2133150–2133152, 2133158–2133159, 2133162–2133164, 2133166, 2133169–2133173, 2133175, 2133178, 2133181–2133182, 2133235, 2133274, 2133287, 2133299, 2133307, 2133317, 2133322, 2133325, 2133327–2133328, 2133333–2133334, 2133336, 2133339, 2133351–2133354, 2133391, 2133429, 2133531, 2133541, 2133579, 2133592, 2133609, 2133613–2133614, 2133631, 2133633, 2133647, 2133655, 2133665, 2133667, 2133680, 2133682, 2133684, 2133694, 2133701, 2133703, 2133706, 2133709, 2133719, 2133725, 2133732, 2133737–2133738, 2133748, 2133759, 2133761, 2133790, 2133824, 2133827, 2133832, 2133839, 2133848, 2133943, 2133952, 2133954, 2133961, 2133963, 2133965, 2133970, 2133975–2133977, 2133984, 2134135, 2134180, 2134273, 2134314, 2134317, 2134325, 2134327–2134328, 2134342, 2134354, 2134374, 2134392, 2134602, 2134605, 2134659–2134660, 2134766, 2134784, 2134873, 2135060, 2135913, 2135915, 2135928, 2135980, 2136128, 8097462, 8097466, 8097470, 8097472, 8097488, 8097496, 8097498, 8106048, 8106050, 8106054, 8106056, 8106058–8106059, 8106061, 8106069–8106070, 8106074, 8106079–8106080, 8106099, 8106102, 8106104–8106105, 8106111, 8106113, 8106123, 8106214, 8106217, 8106222, 8106226, 8106237, 8106243, 8106254, 8106258–8106259, 8106261, 8106266–8106267, 8106273, 8106281, 8106290, 8106294–8106295, 8106297, 8106299, 8106318, 8106320, 8106324, 8106328, 8106340, 8106343, 8106350, 8106354, 8106356, 8106358, 8106375, 8106501–8106503, 8106520, 8106526, 8106528, 8106530, 8106548–8106550, 8106552, 8106555, 8106558, 8106570, 8106584, 8106586, 8106589–8106590, 8106600, 8106605, 8106610, 8106625, 8106628, 8106636–8106637, 8106644, 8106648–8106649, 8106651–8106652, 8106671–8106672, 8106680, 8106685, 8106692, 8106701, 8106718, 8106728, 8106743, 8106754, 8106758, 8106761, 8106765, 8106769, 8106783, 8106786, 8106796, 8106806, 8106827, 8106833, 8106835, 8106844, 8106864, 8106873, 8106883, 8106893 (CASC); CASENT 2042129, 2042276, 2043070, 2043250, 2043908, 2043910, 2043912, 2043920, 2043923–2043925, 2132004, 2132097, 2132128, 2132147, 2132155, 2132199, 2132231, 2132248, 2132253–2132254, 2132263, 2132272, 2132286, 2132299, 2132352, 2132387, 2132436, 2132577, 2132581–2132582, 2132592, 2132595–2132596, 2132598, 2132609, 2132615, 2132622, 2132632, 2132646, 2132650, 2132664, 2132676, 2132684, 2132688, 2132693, 2132706–2132707, 2132868, 2132915, 2132922, 2132924, 2132926, 2132934, 2132939, 2132955–2132956, 2132960, 2132974, 2132978, 2132982, 2132994, 2133092, 2133103, 2133107–2133109, 2133123–2133124, 2133141, 2133143–2133144, 2133146–2133147, 2133154–2133155, 2133157, 2133174, 2133176, 2133179, 2133315–2133316, 2133319, 2133371, 2133451, 2133504, 2133526, 2133554, 2133562, 2133566, 2133580–2133582, 2133590, 2133603, 2133605–2133606, 2133618, 2133620, 2133645, 2133668, 2133702, 2133716, 2133720, 2133741, 2133755, 2133765, 2133769, 2133785, 2133800, 2133803, 2133829, 2133964, 2133983, 2133990–2133991, 2133995–2133997, 2134023, 2134033, 2134215, 2134217, 2134320, 2134332, 2134334, 2134337, 2134345, 2134351, 2134356, 2134369, 2134375–2134376, 2134386, 2134601, 2134652, 2134800, 2135917, 8106071, 8106073, 8106093, 8106109, 8106114, 8106121, 8106175, 8106240, 8106300–8106301, 8106304, 8106396, 8106450, 8106452–8106453, 8106597, 8106602, 8106703, 8106711, 8106713, 8106722, 8106725, 8106756, 8106871, 8106891, 8106973–8106974, 8106978–8106979 (OSUC).

##### Comments.

Individuals of *Scelio parkeri* are generally larger than those of *Scelio harinhalai*, and the smooth patch on the meso- and metapleuron is generally larger and more prominent. The mesonotum, particularly the mesoscutellum, tends to have slightly more longitudinally striate strigose and also often somewhat finer sculpture than seen in the other species.

### *Scelio simoni* species group

**Description.** *General*. Body size: small; moderate. Body length: 3.06–3.94 mm. Habitus: typical, mesosoma not dorsoventrally flattened. Body color: brown to dark brown. Fore leg color: concolorous with mid and hind legs. Sculpture: moderate to robust, reticulate to strigose, generally without longitudinal or parallel lineations. Wing type: macropterous.

*Pilosity*. General setation: moderate elongate and wide, variously vertically oriented. Thickened and truncate white pilosity: not typically present, or strongly truncate when so. Interommatidial pilosity: present; absent. Genal pilosity density: sparse. Genal pilosity color: brown. Number of anteclypeal setal pairs: 4 more or less similarly sized pairs. Ventrolateral postgenal cluster of erect setae: absent. Antespiracular setal patch: small to moderate size, present immediately below posterior extension of lateral epomia. Netrion: setose; glabrous. Propodeal shelf: narrow strip present laterally. Pilosity of laterotergites: absent.

*Head*. Sculpture of head: predominantly reticulate to rugulose throughout, never predominantly dorsoventral or longitudinal, without prominent smooth or obliterated patches, carinae moderate width. Ocelli size: small. Gap between antennal toruli and anteclypeus: narrow to moderate width. Width of ventral head across mandibles: moderate, mandibles typically formed. Anteclypeus shape between outer teeth: slightly concave, with thicker medial projection whose margins nearly reach outer teeth. Malar sulcus: present. Medial portion of occipital carina: percurrent; broadly obliterated or obscured medially. Lateral portion of occipital carina: more or less linear throughout. Form of gena: narrow (lateral view), strongly sloped from posterior margin of eye to occipital carina. Genal carina: absent. RSS on A5 in males: present. Microsculpture at base of mandible: present; absent. Basal tooth of mandible: absent.

*Mesosoma*. Shape of mesoscutum in lateral view: moderately concave, not particularly bulging in anterior nor flattened throughout. Transverse pronotal carina in female: absent or very irregularly developed, anterolateral shoulder somewhat rounded, without clear division between dorsal and anterior areas. Surface of mesoscutum: sculptured throughout, never with smooth patches, usually reticulate to rugulose. Smooth or obliterated patches on mesoscutum: absent. Surface of the pronotal collar in females: unknown. Axillula: small, clearly discernible only in lateral view. Propodeal corners: rounded, without pointed vertices. Epomia: present. Surface of oxter: often smooth or with sculpture obliterated or very faint. Fore wing length: reaching or surpassing anterior margin of T5 but not surpassing apex of metasoma. Fore wing submarginal vein near curve towards costal margin: nebulous to spectral. Pictation of fore wing in female: absent.

*Metasoma*. Anterior margin of T1: concave, with short rim.

**Diagnosis.** Members are most similar to species in the *walkeri*, *ipomeae*, and *pulchripennis* species groups which share the rounded posterolateral propodeal corners (Figs 3–6) and the presence of moderate to long pilosity throughout. Species of the *irwini*-group have similar propodeal corners but much shorter setae throughout. Of these species groups the roundly reticulate to almost punctate-foveate sculpture of the mesoscutum (Figs 313, 319, 325) is diagnostic for the *simoni*-group. Its species may be differentiated from the *pulchripennis*-group by the glabrous netrion (or nearly so); the netrion is densely setose in the *pulchripennis*-group. From the *walkeri*-group they may be distinguished by the absence of thick white truncated setae and the presence of brown setae of the gena (always white in walkeri species). Males may can be differentiated from the *walkeri* and *pulchripennis* groups by the presence of the RSS on A5 (absent or not visible at 60× in those groups).

**Comments.** The three species in this group are very similar. If the degree of development of sculpture, in particular the transverse carina of T6, is correlated with size, the discovery of intermediates in body size may require concepts to be synonymized. The sculpture of the mesoscutum is diagnostic and is approached in form only by males of some *pulchripennis*-group species. The transverse pronotal carina is notably more or less absent in all species, with the pronotal shoulder somewhat rounded. The fore wing submarginal vein is absent all but basally where it is indicated as a slightly darkened sclerotized fold. The ventrolateral postgenal cluster of setae is perhaps represented by one or two erect setae, although it also could be interpreted as absent. Several individuals of *Scelio simoni* have a single setae arising from the anteromedial netrion, though in most individuals within the group this sclerite is glabrous. Overall, the pilosity of the body is relatively sparse, this is particularly noticeable on the propodeum. There is a slight rounded point (Fig. 3) corresponding to the inner margin of the propodeal projection (as in Fig. 1) in some individuals. For the three species this state seems to be somewhat plastic.

**Key to *simoni*-group species** (also available online at http://www.waspweb.org/Platygastroidea/Keys/index.htm)

**Table:** 

1	Mesopleural depression nearly completely smooth; T6 in females not transversely divided by well-developed carina (Fig. 322); body light brown to brown; female body length 3.1 mm	*Scelio simonolus*
–	Mesopleural depression predominantly sculptured with only small smooth patches if present; T6 in females transversely divided by well-developed carina (Fig. 328); body dark brown to black; female body length 3.1–3.9 mm	2
2	Eye with short microtrichia present between ommatidia; males with notauli irregularly impressed, but clearly present; male RSS on A5, short, thick, nodelike; female body length 3.1–3.7 mm	*Scelio simoni*
–	Eye glabrous; males with notauli absent; male RSS on A5 linear, finely carinate; female body length 3.8–3.9 mm	*Scelio vannoorti*

#### Scelio simoni

Yoder sp. n.

http://zoobank.org/4DC5B8BB-301C-4D58-B7CD-A8FE6BD3D0BD

urn:lsid:biosci.ohio-state.edu:osuc_concepts:244593

http://species-id.net/wiki/Scelio_simoni

Figures 3 , 10 , 311–316 ; Morphbank ^67^

##### Description.

Female body length: 3.08–3.73 mm (n=10). Male body length: 3.16–3.57 mm (n=16). Body color in female: dark brown to black. Setae between ommatidia in female: present. Form of RSS on A5 in male: nodelike. Surface of pronotal nucha in female: partially to completely transversely striate, at most with slight obliterated patch. Notauli in males: present. Surface of mesopleural depression in female: more or less sculptured throughout. Carinate division of posterior T6 in female: present.

##### Diagnosis.

This species differs from *Scelio simonolus* by presence of a well-developed carina dividing T6 (as in Fig. 328) and the sculptured mesopleural depression. It differs from *Scelio vannoorti* by the presence of short microtrichia among the ommatidia, the nodelike RSS on male A5 (vs. linear and carinate) and the presence of notauli in the males.

**Figures 311–316.**
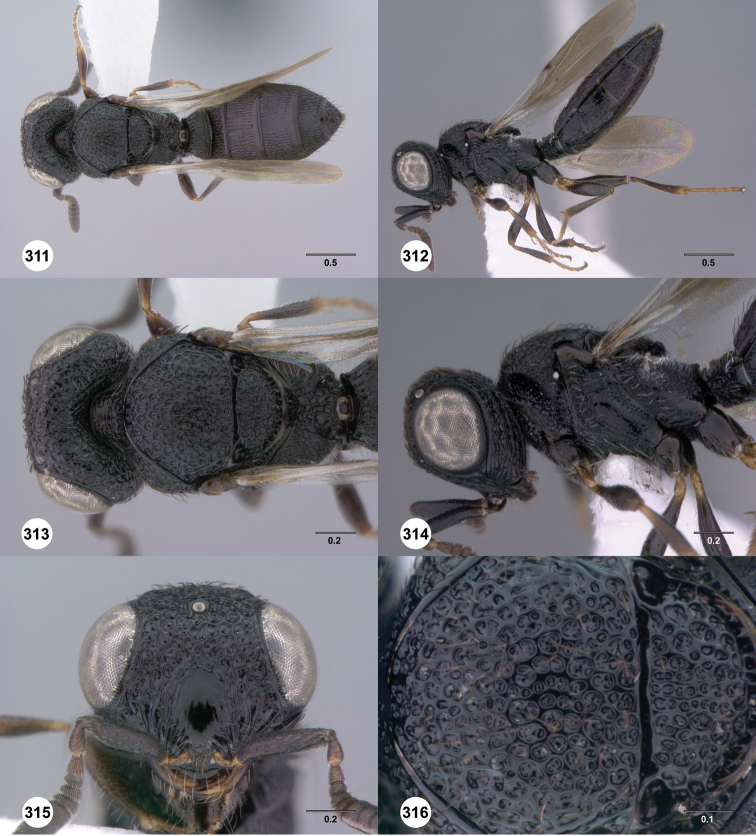
^166^ *Scelio simoni* sp. n. **311, 313, 315** paratype female (OSUC 250748); 312, 314, 316 holotype female (OSUC 214201). **311** Habitus, dorsal view **312** Habitus, lateral view **313** Head and mesosoma, dorsal view **314** Head and mesosoma, lateral view **315** Head, anterior view **316** Mesonotum, dorsal view. Scale bars in millimeters.

##### Etymology.

The epithet is used as a genitive noun derived from the name of the sole collector of all known material, Simon van Noort.

##### Link to distribution map.

http://hol.osu.edu/map-large.html?id=244593

##### Material examined.

*Holotype*, female: **SOUTH AFRICA**: Western Cape Prov., Cape Town, Constantiaberg, 34°02'S, 18°23'E, 460m, 10.III–17.III.1995, malaise trap, S. van Noort, OSUC 214201 (deposited in SAMC). *Paratypes*: **SOUTH AFRICA**: 9 females, 16 males, OSUC 212450 (CNCI); OSUC 213634, 213699 (OSUC); OSUC 213538, 213574, 213695, 213697–213698, 213700, 214202, 214232, 214235, 244040, 250669–250670, 250713, 250716, 250742–250744, 250746–250748, 250983 (SAMC); OSUC 250668 (SANC).

#### Scelio simonolus

Yoder sp. n.

http://zoobank.org/5C0BE8E1-8348-4BA6-A705-365EAADF4245

urn:lsid:biosci.ohio-state.edu:osuc_concepts:244594

http://species-id.net/wiki/Scelio_simonolus

**Figures 317–322.**
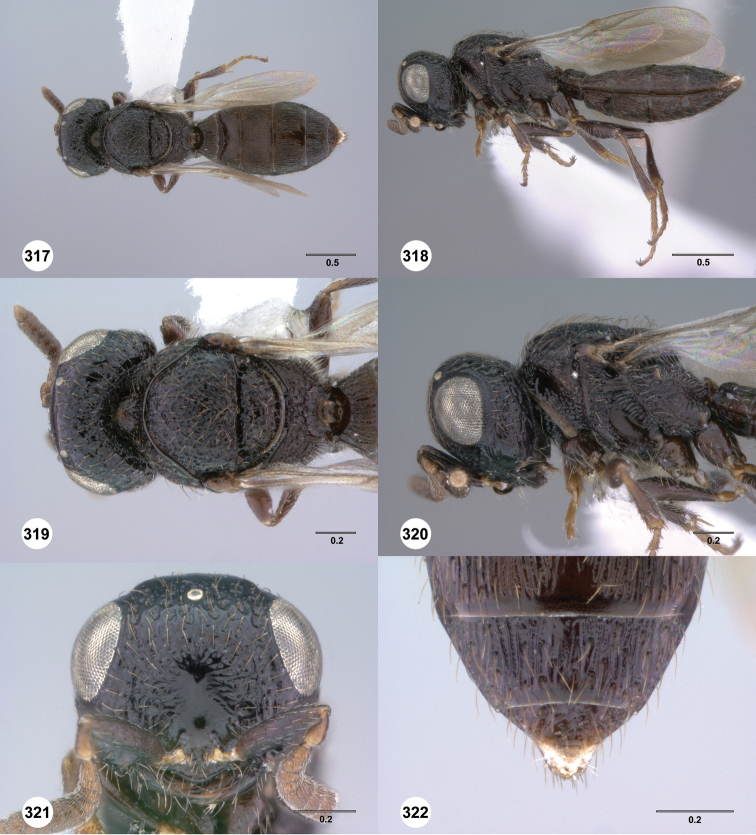
^167^ *Scelio simonolus* sp. n., paratype female (OSUC 250745). **317** Habitus, dorsal view **318** Habitus, lateral view **319** Head and mesosoma, dorsal view **320** Head and mesosoma, lateral view **321** Head, anterior view **322** apex of metasoma, dorsal view. Scale bars in millimeters.

Figures 317–322 ; Morphbank ^68^

##### Description.

Female body length: 3.06–3.08 mm (n=2). Body color in female: light brown to brown. Setae between ommatidia in female: absent. Surface of pronotal nucha in female: smooth (with only setigerous punctures) to slightly rugulose. Surface of mesopleural depression in female: almost completely smooth. Carinate division of posterior T6 in female: absent.

##### Diagnosis.

*Scelio simonolus* differs from both *Scelio vannoorti* and *Scelio simoni* by the nearly completely smooth mesopleural depression (Fig. 320) and the absence of a carina dividing T6 (Fig. 322, compare with Fig. 328).

##### Etymology.

The epithet is used as an adjective derived from the name of the sole collector of all known material, Simon van Noort, and referring to the diminutive size of this species.

##### Link to distribution map.

http://hol.osu.edu/map-large.html?id=244594

##### Material examined.

*Holotype*, female: **SOUTH AFRICA**: Western Cape Prov., Constantiaberg, Table Mountain N.P., site 3, Cape Town, 460m, no date, OSUC 250715 (deposited in SAMC). *Paratype*: **SOUTH AFRICA**: 1 female, OSUC 250745 (SAMC).

##### Comments.

*Scelio simonolus* is the least robustly sculptured of the three species in this group. The sculpture of the pronotal nucha is somewhat obliterated as compared to the transverse striae in the other two species.

#### Scelio vannoorti

Valerio & Yoder sp. n.

http://zoobank.org/5494CC6F-9FB2-465B-880A-115EF6DBBBC4

urn:lsid:biosci.ohio-state.edu:osuc_concepts:244595

http://species-id.net/wiki/Scelio_vannoorti

**Figures 323–328.**
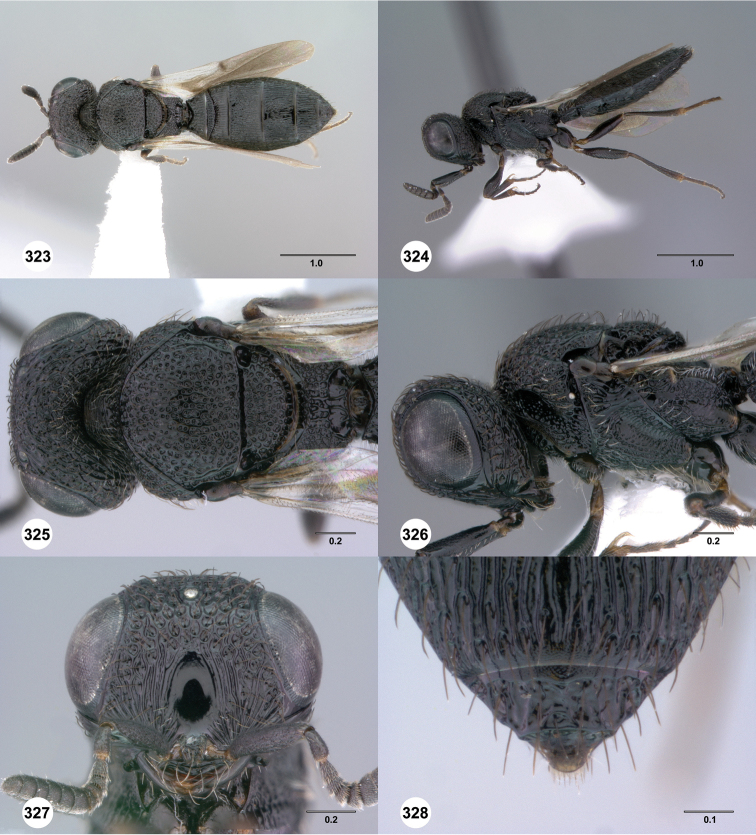
^168^ *Scelio vannoorti* sp. n., holotype female (OSUC 213199). **323** Habitus, dorsal view **324** Habitus, lateral view **325** Head and mesosoma, dorsal view **326** Head and mesosoma, lateral view **327** Head, anterior view **328** Apex of metasoma, dorsal view. Scale bars in millimeters.

Figures 323–328 ; Morphbank ^69^

##### Description.

Female body length: 3.82–3.94 mm (n=2). Male body length: 3.68 mm (n=1). Body color in female: dark brown to black. Setae between ommatidia in female: absent. Form of RSS on A5 in male: linear, a fine carina. Surface of pronotal nucha in female: partially to completely transversely striate, at most with slight obliterated patch. Notauli in males: absent. Surface of mesopleural depression in female: more or less sculptured throughout. Carinate division of posterior T6 in female: present.

##### Diagnosis.

This species differs from *Scelio simonolus* by the sculptured mesopleural depression (Fig. 326). It may be distinguished from the similar *Scelio simoni* by the absence of pilosity between the ommatidia and, in males, the linear RSS on A5 and the absence of discernible notauli.

##### Etymology.

The epithet is used as a genitive noun derived from the name of the sole collector of all known material, Simon van Noort.

##### Link to distribution map.

http://hol.osu.edu/map-large.html?id=244595

##### Material examined.

*Holotype*, female: **SOUTH AFRICA**: Western Cape Prov., Cape Town, Constantiaberg, 34°02'S, 18°23'E, 460m, 10.III–17.III.1995, malaise trap, S. van Noort, OSUC 214201 (deposited in SAMC). *Paratypes*: **SOUTH AFRICA**: 1 female, 1 male, OSUC 212543, 213198 (CNCI).

### *Scelio walkeri* species group

**Description.** *General*. Body size: small; moderate. Body length: 2.95–5.15 mm. Habitus: typical, mesosoma not dorsoventrally flattened. Body color: brown to dark brown. Fore leg color: concolorous with mid and hind legs. Sculpture: often fine or faint, reticulate to rugulose, at most with poorly developed parallel lineations. Wing type: macropterous.

*Pilosity*. General setation: moderate elongate and wide, variously vertically oriented. Thickened and truncate white pilosity: present. Interommatidial pilosity: present; absent. Genal pilosity density: sparse; moderate. Genal pilosity color: white. Number of anteclypeal setal pairs: 3, with fine and much smaller fourth laterally. Ventrolateral postgenal cluster of erect setae: absent. Antespiracular setal patch: very small, intersected by or immediately below lateral epomia. Netrion: glabrous. Propodeal shelf: glabrous except for anterolateral corner. Pilosity of laterotergites: absent.

*Head*. Sculpture of head: predominantly dorsoventrally striate on frons, often with obliterated or smooth patches dorsally, carinae usually fine. Ocelli size: small. Gap between antennal toruli and anteclypeus: absent, toruli reaching anteclypeus or anteclypeus more or less absent with clypeus concave; narrow to moderate width. Width of ventral head across mandibles: moderate, mandibles typically formed; broad, mandibles wide. Anteclypeus shape between outer teeth: broadly concave, or strip like and slightly projected, or projected and roundly rectangular. Malar sulcus: present. Medial portion of occipital carina: percurrent. Lateral portion of occipital carina: more or less linear throughout. Form of gena: broad, somewhat bulging, not evenly rounded from posterior margin of eye to occipital carina. Genal carina: absent. RSS on A5 in males: absent, or not visible at 60×. Microsculpture at base of mandible: absent. Basal tooth of mandible: present.

*Mesosoma*. Shape of mesoscutum in lateral view: moderately concave, not particularly bulging in anterior nor flattened throughout. Transverse pronotal carina in female: well developed, predominantly linear, anterolateral shoulder somewhat truncate from dorsal to anterior areas. Surface of mesoscutum: sculptured throughout, never with smooth patches, always reticulate at least in part, with at most slight longitudinal elements. Smooth or obliterated patches on mesoscutum: absent. Surface of the pronotal collar in females: finely sculptured throughout. Axillula: small, clearly discernible only in lateral view. Propodeal corners: rounded, without pointed vertices. Epomia: present. Surface of oxter: often smooth or with sculpture obliterated or very faint. Fore wing length: reaching or surpassing anterior margin of T5 but not surpassing apex of metasoma. Fore wing submarginal vein near curve towards costal margin: tubular. Pictation of fore wing in female: absent.

*Metasoma*. Anterior margin of T1: concave, with short rim.

**Diagnosis.** Most similar to species in the *ipomeae*, *pulchripennis* and *irwini* species groups, all of which share the rounded posterolateral corners of the propodeum. Females differing from species in these groups and all others (except one species of the *ipomeae*-group) by the presence of a basal tooth on the mandible (Figs 330, 375, *bt*). Most species in this group are further identifiable by the combination of the distinctly bent pilosity of the villus in lateral view (Fig. 374, *vil*); presence of pilosity above the hind coxa; fine dorsoventral striae of the head; thick, white, often truncate setae (females); the relatively broad head in dorsal view; absence of the RSS on male A5; and the truncate subapical margin of T6 (females). Note that individual exceptions to many of these characters exist. For example, the RSS of A5 in males is also absent in *pulchripennis*-group species. Unlike species of the *pulchripennis*-group in which the sculpture of the mesonotum is frequently obliterated or smooth, the mesonotum of *walkeri*-group species is always sculptured throughout.

**Comments.** This species of this group are very similar to one another, as noted by Nixon (1958). The delimitation of species is heavily based on characters of pilosity and the form of the anteclypeus. While interpretation of setal color may be difficult, it is possible and very useful. Interpretation is aided by using the setae of the lower gena as the standard of “white.” This allows for comparison to the color of mesonotal pilosity in particular, which can be very subtly darker. In the material we observed only very rarely was pilosity color positively misleading. In most of these cases the features were obviously artifacts, e.g. of collecting method, dirt, or extraneous films. The color patterns in older material (more than 25 years) appear to be stable. The thickened white pilosity may be broadly categorized as belonging to three categories, white to very slightly off-white and opaque, golden-brown to brown and opaque, and translucent white. The last state is only rarely seen in Afrotropical *walkeri*-group species, but was commonly observed in Australian species.

Several morphological trends are notable. No *walkeri*-group species are known with yellow, orange or metallic color. The antennae are typically unicolorous, with at most a slightly lighter base. Patterns of sculpture throughout the Afrotropical *walkeri*-group appear to be largely uniform and uninformative. Therefore, we have paid much less attention to their description. The sculpture of the head most often consists of fine dorsoventral striae with smooth interstices. This is particularly fine, while that on the mesosoma and metasomal is somewhat coarser. The anterior portion of the gena immediately below the eye and posterior to the malar sulcus always has a small glabrous patch (Fig. 350, *gp*). The cluster of erect straight setae on the ventrolateral postgena is coded as absent because the form of the cluster found in the *irwini* and *ipomeae*-groups is not present. There are some species, however, in which 1-3 setae are present that may be homologous. The tibia is always lighter in color (dark yellow at lightest) than the femur. The general pilosity is never long, thin, and distinctly erect as seen in many *pulchripennis*-group species. The thickened, truncate, white setae are particularly well represented on the pronotal shoulders and lateral portions of T2, though they are often found throughout the body. The propodeum is glabrous except for a patch of setae on the anterolateral corner. The subapical margin of T6 is poorly differentiated and usually is found very near to the apex of T6. As a result, the smoother apical area is very small.

Males of this group are additionally difficult to identify to species because the characters useful with respect to females, in particular the shape of the anteclypeus and the distribution of pilosity, are largely homogeneous. The general pilosity in males is much finer, not apically truncate, and sparse along the lateral metasoma. The basal tooth on the mandible is apparently absent in males: at most there is a very slight sinuate edge present. In general, the sculpture is coarser throughout in males.

The *walkeri*-group is not known from the New World, but is widespread through the Palearctic, Afrotropical, Oriental, and Australasian realms. We expect additional species to be discovered within the study area. We suspect their delimitation will require the addition of long series of new material, host data, or the use of molecular characters. The geographical limit of species treated here excludes several species with potentially overlapping geographic ranges from the Arabian Peninsula. Several names for these Arabian species are available and potentially applicable to the Afrotropical species. These include *Scelio popovi* Nixon and *Scelio aegyptiacus* Priesner.

**Key to *walkeri*-group species** (also available online at http://www.waspweb.org/Platygastroidea/Keys/index.htm)

**Table:** 

1	Metasomal tergites 2–5 in lateral profile covered with dense pilosity in anterior half, strongly contrasting with the glabrous or very sparsely setose posterior half (Fig. 396)	*Scelio striatus*
–	Metasomal tergites 2–5 in lateral profile with at least some setae present in posterior half, if sparse in posterior half then uniformly sparse throughout	2
2	Frons below and surrounding anterior ocellus with well-defined reticulate sculpture (Fig. 393, note that several other species from the Arabian Peninsula possess this character, and may overlap in distribution)	*Scelio retifrons*
–	Frons below and surrounding anterior ocellus with fine dorsoventral striae, rarely some slight reticulation among striae but in this case dorsoventral striae clearly predominate (e.g., Figs 339, 369, 387)	3
3	Gena strongly bulging (Fig. 385), in dorsal view usually extending laterad of eye, head very wide across mandibles (Fig. 387); medial anteclypeus very short and concave, with two acute triangular teeth at margins (Fig. 387, *lat*); pilosity of lateral metasoma sparse (Fig. 388); striae on frons dense (Fig. 388) (scutellum with fine longitudinal striae, Fig. 385; T5 often appearing slightly pinched, with a relatively well-developed median crease; anterolateral T3 with a large patch of appressed microtrichia; lateral S2–S5 predominantly sculptured)	*Scelio remaudierei*
–	Gena slightly bulging or simply rounded, not extending laterad of eye, head moderately wide across mandibles; medial anteclypeus variously developed though typically not with distinct acute triangular teeth at margins; pilosity of metasoma rarely sparse, typically moderate to dense (e.g., Figs 376, 382)	4
4	Anteclypeus medially projecting (Figs 351, 381); posteroventral quadrant of metapleuron densely setose (4 or more setae) (Fig. 350, 380)	5
–	Anteclypeus medially striplike (linear) to concave (e.g. Figs 339, 369, 405); posteroventral quadrant of metapleuron sparsely setose (1–3 originating setae)	6
5	Anteclypeus medially broadly lobed (Fig. 351), without sculpture (pilosity throughout often slightly wavy)	*Scelio clypeatus*
–	Anteclypeus strongly projecting, apical margin truncate to very slightly convex, with irregular sculpture (Fig. 381)	*Scelio quasiclypeatus*
6	Posterodorsal portion of head with some smooth patches formed by irregularly obliterated sculpture (Fig. 329); dorsal surface of anterolateral T3 with a patch of micropilosity (best seen in dorsolateral view, Fig. 340, *t3p*); pilosity of head and mesoscutum predominantly white, mesoscutellar setae brown (Fig. 337); pronotal nucha with some areas distinctly smooth (anteclypeus almost always concave and quite short, though in some appearing truncate at some angles, Fig. 339)	*Scelio afer*
–	Posterodorsal portion of head sculptured throughout, if with obliterated patches (rarely), then pronotal nucha well sculptured throughout; dorsal surface of anterolateral T3 with or without micropilosity; color of pilosity on head and dorsal mesonotum varying; sculpture of pronotal nucha varying, but in most sculptured throughout	7
7	Anteclypeus medially truncate (e.g. Figs 369, 375), if irregularly concave medially (typically smaller individuals), then medial teeth absent (Fig. 387)	8
–	Anteclypeus medially concave (e.g. Figs 339, 405)	10
8	Pilosity of lateral T3–T5 absent anteriorly such that no part of the anterior margin has nearby adjacent setae (Fig. 346); pilosity of posteroventral quadrant of metapleuron moderately dense, typically with 3 or more setae present (Fig. 344) (anteclypeus quite small, Fig. 345, sometimes with a hint of medial concavity; mesosoma typically with 7 or more brown setae)	*Scelio apospastos*
–	Pilosity of lateral T3–T5 reaching the anterior margin, at least along lateral margin; pilosity of posteroventral quadrant of metapleuron sparse, typically 1-2 setae present	9
9	Pilosity of mesoscutum completely white, or with at most a single pair of brown setae along posteromedial margin (Fig. 373); anteclypeus well developed, often projecting medially (Fig. 375); T1 laterally with two types of setae present, finer setae forming a complete patch from the anterior to the posterior; lateral T2–T5 densely setose, setae predominantly white	*Scelio pilosilatus*
–	Pilosity of mesoscutum often predominantly brown, with more than two brown setae medially (Fig. 367); anteclypeus small, striplike, sometimes with anterior margin slightly irregular; T1 laterally with predominantly thicker setae, if fine setae present then not forming a complete dense line from the anterior to the posterior; pilosity of lateral T2–T5 moderately dense to somewhat sparse; pilosity of T4–T5 at least partially (often completely) composed of fine brown to golden-brown setae (widespread and somewhat polymorphic, includes several series of tentatively included individuals, see comments)	*Scelio modulus*
10	Metasomal pilosity on T3–T5 white, thick, concolorous with pilosity of T2; oxter nearly always with a relatively large glabrous and smooth patch along dorsal margin; pilosity of the head and mesonotum with at least some white setae	11
–	Metasomal pilosity on T3–T5 very fine, brown, contrastingly lighter than that of T2; oxter sculptured throughout; pilosity of the head and mesonotum completely brown, concolorous (Kenya, Rwanda)	*Scelio concavus*
11	Pilosity of head, mesonotum not concolorous, distinctly multicolored on mesoscutum, and dark throughout mesoscutellum (Fig. 361); pronotal nucha predominantly smooth throughout; mesoscutum with relatively large reticulations; mesoscutum and mesoscutellum more transverse (Fig. 361, width of mesoscutum 1.5× length, width of mesoscutellum 2.3× length) (west Africa)	*Scelio erugatus*
–	Pilosity of dorsal head and mesonotum concolorous; pronotal nucha sculptured throughout; mesoscutum with moderately sized reticulations (Fig. 403); mesoscutum and mesoscutellum more elongate (Fig. 403, width of mesoscutum 1.1× length, width of mesoscutellum 1.1× length) (Madagascar)	*Scelio tritus*

#### Scelio afer

Kieffer

http://zoobank.org/75A6D327-2DBE-475F-A97F-E7E9B67DE436

urn:lsid:biosci.ohio-state.edu:osuc_concepts:5165

http://species-id.net/wiki/Scelio_afer

Figures 7 , 329–332 , 335–340 ; Morphbank ^70^

Scelio afer Kieffer, 1905: 130 (original description); Kieffer 1908: 131 (keyed); Kieffer 1926: 310, 323 (description, keyed); Ferrière 1952: 118 (diagnosis); Masner 1976: 17 (lectotype designation).
Scelio (Scelio) afer Kieffer: Kieffer 1910: 74 (subgeneric assignment).
Scelio afer Scelio clarus Fouts, 1934: 102 (original description); Ferrière 1952: 118 (diagnosis); Bin 1974: 463 (type information).
Scelio afer http://zoobank.org/7359AD56-ACA0-4526-922D-9A6F4A2743C9
Scelio afer urn:lsid:biosci.ohio-state.edu:osuc_concepts:5198
Scelio africanus Risbec, 1950: 586 (original description); Nixon 1958: 311, 317 (keyed); Masner 1976: 17 (type information). New synonymy.
Scelio afer http://zoobank.org/65EE302A-8AF3-4CF5-8530-0741502E1247
Scelio afer urn:lsid:biosci.ohio-state.edu:osuc_concepts:5198

##### Description.

Female body length: 3.06–4.42 mm (n=18). Form of sculpture of frons below anterior ocellus in female: fine dorsoventral striae with few to no reticulations. Distribution of sculpture of frons posterior to anterior ocellus in female: with at least some obliterated or reduced patches of sculpture posteriorly. Color of pilosity of dorsomedial head in female: white or predominantly white. Sculpture of ventrolateral corner of frons adjacent to malar sulcus in male: predominantly dorsoventral. Form of anteclypeus between medial teeth in female: striplike, broadly concave. Form of anteclypeus between medial teeth in males: produced, rounded to truncate medially. Form of lateral gena below eye in anterior view in female: evenly rounded towards mandible, not bulging laterally. Sculpture of anteclypeus: smooth throughout. Sculpture of pronotal nucha in female: absent (smooth) in parts. Color of pilosity on mesonotum in female: predominantly white on mesoscutum, predominantly brown on mesoscutellum. Sculpture of mesoscutellum in female: predominantly irregular rugulose to reticulate. Sculpture of oxter: with prominent smooth patch. Pilosity of metapleuron overlapping or arising within posteroventral quadrant in female: 2 setae; 3 setae. Color of fore wing in female: evenly colored throughout. Color of fore wing in male: completely without color except at extreme base. Color of pilosity on lateral T2–T5 in female: T2–T5 white to off-white. Fine pilosity of lateral T1 in female: absent; present, not reaching posterior margin. Distribution of pilosity on metasomal terga 3–5 in female: more or less uniformly present throughout. Form of setae on lateral T2–T5: predominantly thick throughout. Pilosity of anterolateral corner of dorsal T3 in female: with patch of short appressed micropilosity. Form of medial surface of S3–S5 in males: broadly concave, S3 posterior concavity extending into anterior half of sclerite.

##### Diagnosis.

*Scelio afer* is similar to other species with a narrow, concave anteclypeus (*Scelio tritus*, *Scelio remaudierei*, *Scelio erugatus*). It may be differentiated from *Scelio remaudierei* by the slightly narrower gena (very broad in *Scelio remaudierei*) and thicker and concolorous setae of the lateral metasoma (setae very thin in *Scelio remaudierei*, with those on T2 white and T3–T5 brown). *Scelio afer* differs from *Scelio tritus* by the presence of smooth patches on the pronotal nucha (robustly sculptured in all *Scelio tritus*), and the presence of microsetae in the anterolateral corner of T3 (glabrous to very sparse in *Scelio tritus*). Differing from *Scelio popovi* Nixon (described from Oman) by the pilosity of the lateral metasoma which is white and moderate to dense compared to brown and sparse.

**Figures 329–334.**
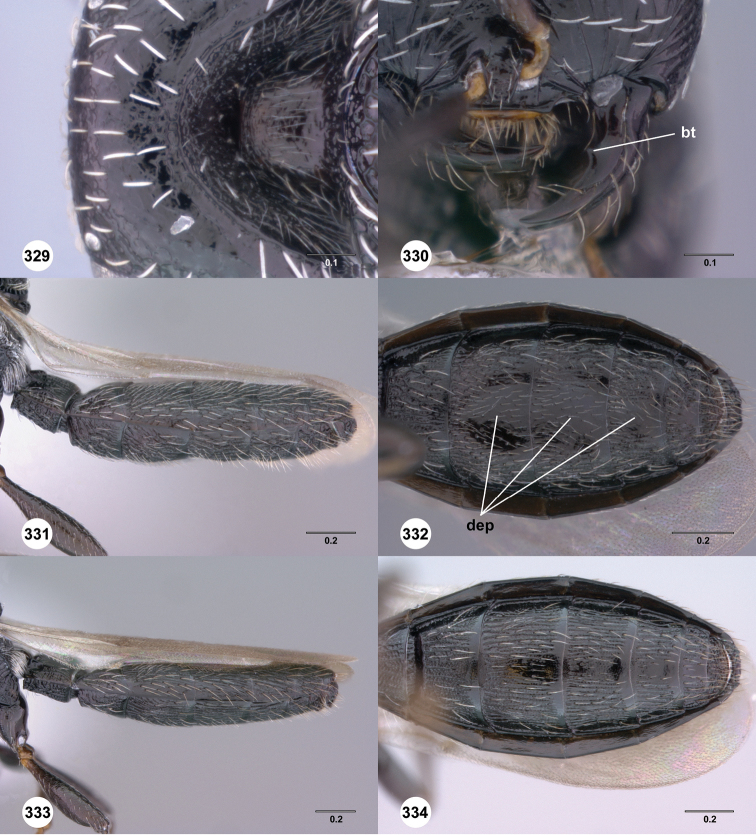
^169^ **329–332** *Scelio afer* (Kieffer) **333–334** *Scelio modulus* sp. n. **329** Head, dorsal view, female (OSUC 212366) **330** Mouthparts, anterior view, female (OSUC 250693) **331** Metasoma, lateral view, male (OSUC 250691) **332** Metasoma, ventral view, male (OSUC 250691) **333** Metasoma, lateral view, paratype male (OSUC 250918) **334** Metasoma, ventral view, paratype male (OSUC 250918). *bt*, basal tooth of mandible; *dep*, metasomal sternite depressions. Scale bars in millimeters.

##### Link to distribution map.

http://hol.osu.edu/map-large.html?id=5165

##### Associations.

Living in *Acacia nilotica* (Linnaeus) [Fabales: Fabaceae]; emerged from ootheca of *Acrida madecassa* (Brancsik) [Orthoptera: Acrididae].

##### Material examined.

*Lectotype*, male, *Scelio afer*: **GAMBIA**: no date, Mocquerys (deposited in MNHN). *Lectotype* (**present designation**), male, *Scelio africanus*: **CHAD**: Chari-Baguirmi Rég., shore, Mandjafa (Mandjaffa), 1904, J. Decorse, OSUC 244194 (deposited in MNHN). *Paralectotype*, *Scelio africanus*: **CHAD**: 1 female, OSUC 244190 (MNHN). *Holotype*, female, *Scelio clarus*: **SOMALIA** [Italian Somaliland]: V. Duca Abruzzi, iii.926, Miss. Enr. Paoli; Ex-coll Paoli; TYPE *Scelio clarus* Fouts, Det. R.M. Fouts; “La Specola” Firenze 289; Rientrato nel giugno 2008 nel Museo Civ. di Storia Naturale “G. Doria”di Genova; Esemplare rimasto per molto anni nel Museo Zool. de “La Specola” di Firenze (deposited in MCSN). *Other material*: (209 females, 78 males, 9 unknowns) **BENIN**: 2 females, 2 unknowns, BMNH(E)#790385–790386 (BMNH); OSUC 211369, 211373 (CNCI). **BOTSWANA**: 2 males, OSUC 211265 (CNCI); OSUC 160044 (EMEC). **BURKINA** **FASO**: 2 females, OSUC 213082, 213093 (CNCI). GABON: 1 female, OSUC 213125 (CNCI). **IVORY COAST**: 9 females, OSUC 213059, 213065, 213068, 213072, 213226, 213235–213236, 213239–213240 (CNCI). **KENYA**: 9 females, 2 males, OSUC 212820, 214018, 214157, 214177 (CNCI); OSUC 56046, 58976, 59016, 59018, 59035, 59076, 70701 (OSUC). **MADAGASCAR**: 17 females, 1 male, OSUC 261341–261358 (MNHN). **MALI**: 1 unknown, BMNH(E)#790383 (BMNH). **NAMIBIA**: 1 female, 1 unknown, BMNH(E)#790382 (BMNH); OSUC 213023 (CNCI). **NIGER**: 2 males, OSUC 251072–251073 (TAMU). **NIGERIA**: 3 females, 7 males, OSUC 250766, 250770, 250778, 250946 (CNCI); OSUC 142619–142622, 59132, 59135 (OSUC). **SOUTH AFRICA**: 110 females, 44 males, 3 unknowns, BMNH(E)#790379, 790381, 790384 (BMNH); OSUC 211269, 211279, 211282, 211287, 211297, 211355–211356, 212319, 212366–212367, 212433, 212518–212522, 212539–212541, 212545, 212547–212548, 212550–212552, 212554, 212556–212557, 212559–212562, 212566, 212739, 212768, 212834, 212864, 212870, 212877, 212886, 212993, 213000, 213020, 213120, 213255, 214084, 234668, 234670–234672, 250685, 250688–250695, 250697–250703 (CNCI); OSUC 254548 (MZLU); OSUC 211757, 211862–211865, 213534, 213536, 213539, 213541, 213543–213546, 213657–213660, 213674–213677, 213679–213680, 213683, 213929–213931, 213933, 214183–214186, 222324–222329, 223325, 254536–254539, 254618–254623 (SAMC); OSUC 174706–174707, 213326, 213329, 213331–213332, 213339–213342, 213344, 213348, 213350–213352, 213373–213374, 213380, 213386, 213392, 213398, 213405, 213407, 213419, 213421–213422, 213425, 213427–213428, 213430, 213437, 213442–213444, 213446, 213450, 213453, 213483 (SANC). **SUDAN**: 1 female, OSUC 244078 (USNM). **SWAZILAND**: 1 female, OSUC 254698 (CNCI). **TANZANIA**: 2 unknowns, BMNH(E)#790378, 790380 (BMNH). **YEMEN**: 20 females, 14 males, OSUC 212485, 212488, 212491, 212494, 212936–212937, 212941–212943, 212946, 250875–250878, 250880–250882, 250884–250887, 250889–250894, 250897, 250899, 250901–250904, 250941 (CNCI). **ZIMBABWE**: 33 females, 6 males, OSUC 211254, 211347–211349, 212099, 212113, 212128, 212131, 212133, 212135, 212150, 212152, 212206, 212208, 212226–212227, 212230, 212234, 212337, 212413, 212592, 212640, 212645, 212648, 212830–212831, 212839–212840, 212902, 212906, 213005, 213018, 213043, 213045, 213058, 213201, 213219, 213233, 213252 (CNCI).

##### Comments.

*Scelio afer* is the most widespread and perhaps most common species of the Afrotropical *walkeri*-group. Based on past determinations and the description of Nixon (1958), it largely corresponds to the past concept of *Scelio remaudierei*. As presently defined it is also the most morphologically variable, with variation roughly tracking longitude. Even given this variation, there is a core set of characters: 1) the pilosity of the dorsomedial head and mesoscutum is predominantly white, often with mesoscutum entirely white except for 1 (Fig. 337) or more pairs of brown pilosity posteromedially; 2) the metasoma has thick, white, somewhat sparse pilosity throughout the lateral margins of T2–T5; 3) the anteclypeus is concave medially; and 4) the sculpture of the dorsal head is obliterated in parts. The last state is difficult to discern in some individuals in which only a few sculptural lines are broken or obliterated, and very obvious in others in which the large majority of the sculpture is absent. The former case is largely seen in smaller individuals, the latter in larger.

Mesoscutal sculpture in this and other species seems to be relatively variable and largely uninformative. Within *Scelio afer* the size and density of the cells formed by reticulations on the mesoscutum is quite variable with those individuals in the south generally exhibiting much smaller more compact cells and those in the north larger cells and more frequent indication of longitudinal sculpture. In general the anteriormost cells are the largest.

**Figures 335–340.**
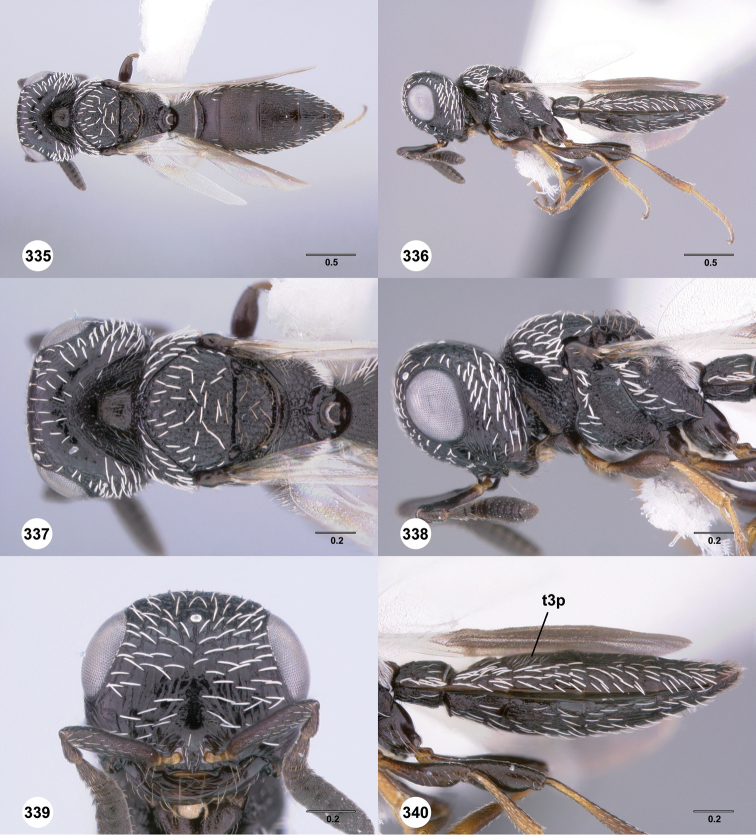
^170^ *Scelio afer* (Kieffer), female (OSUC 212366). **335** Habitus, dorsal view **336** Habitus, lateral view **337** Head and mesosoma, dorsal view **338** Head and mesosoma, lateral view **339** Head, anterior view **340** Metasoma, lateral view. *t3p*, micropilosity of anterolateral corner of T3. Scale bars in millimeters.

The anteclypeus width between the medial teeth is slightly variable, as is the degree of concavity. While most specimens are clearly concave a small number of individuals, primarily from South Africa, have a nearly truncate anteclypeus that has only a hint of concavity. The number of setae of the posteroventral quadrant of the metapleuron is typically 2-3, they are less commonly decreased by 1 or increased by 1–2. The color of the pilosity of the dorsomedial portion of the head is consistently white or off-white (the latter can be discerned by comparison to pilosity of the mesoscutellum, which will be distinctly darker). Males can be diagnosed by the relatively broad depressions medially on the metasoma sterna (Fig. 332).

Several reared series, previously determined as *Scelio remaudierei*, are known from Madagascar. In these specimens the sculpture of the mesonotum is slightly finer than typical, with a more prominent longitudinal trend, and the sculpture of the dorsal head is notable for the extreme reduction of sculpture. Within continental Africa this type of sculpture is typically seen in larger specimens. A single male is is present in the series, it matches well (broad deep sternal depression) with other males. The material from Madagascar may ultimately prove to be a separate species, though additional evidence is likely needed. Testing this hypothesis may prove to be difficult or impossible; despite the extensive recent collecting efforts in Madagascar no new material of *Scelio afer* has been collected since 1964.

#### Scelio apospastos

Yoder sp. n.

http://zoobank.org/F50C8C6D-A678-4AC1-9D8A-FEE2B14F4F1F

urn:lsid:biosci.ohio-state.edu:osuc_concepts:244616

http://species-id.net/wiki/Scelio_apospastos

**Figures 341–346.**
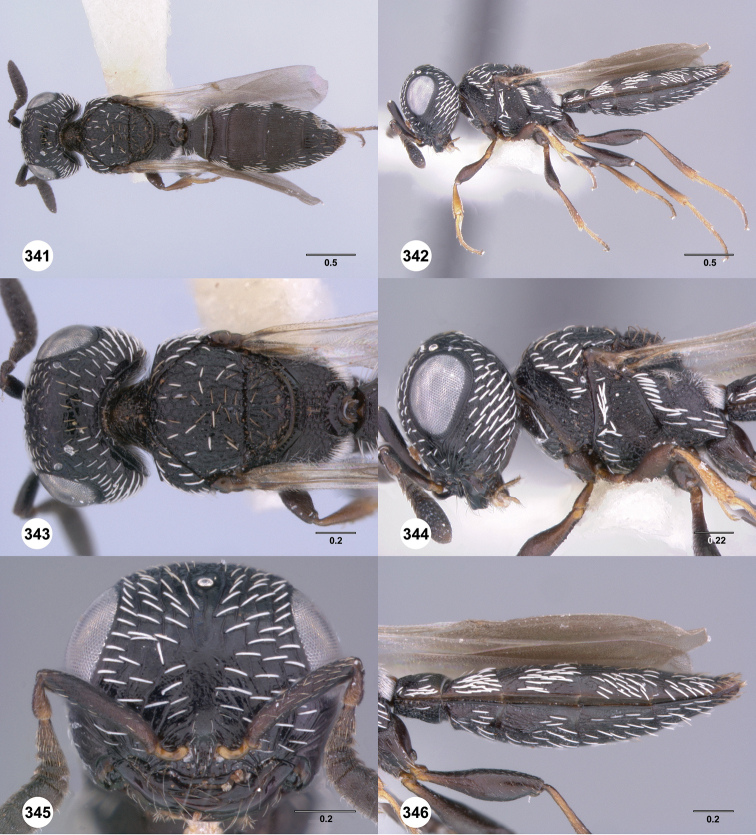
^171^ *Scelio apospastos* sp. n., holotype female (OSUC 213044). **341** Habitus, dorsal view **342** Habitus, lateral view **343** Head and mesosoma, dorsal view **344** Head and mesosoma, lateral view **345** Head, anterior view **346** Metasoma, lateral view. Scale bars in millimeters.

Figures 341–346 ; Morphbank ^71^

##### Description.

Female body length: 3.18–4.08 mm (n=19). Form of sculpture of frons below anterior ocellus in female: fine dorsoventral striae with few to no reticulations. Distribution of sculpture of frons posterior to anterior ocellus in female: more or less uniform throughout. Color of pilosity of dorsomedial head in female: white or predominantly white; brown or predominantly brown. Form of anteclypeus between medial teeth in female: striplike, truncate. Form of lateral gena below eye in anterior view in female: evenly rounded towards mandible, not bulging laterally. Sculpture of anteclypeus: smooth throughout. Sculpture of pronotal nucha in female: present throughout. Color of pilosity on mesonotum in female: predominantly brown throughout; predominantly white on mesoscutum, predominantly brown on mesoscutellum. Sculpture of mesoscutellum in female: predominantly irregular rugulose to reticulate. Sculpture of oxter: present throughout; with prominent smooth patch. Pilosity of metapleuron overlapping or arising within posteroventral quadrant in female: 4 or more setae. Color of fore wing in female: evenly colored throughout. Color of pilosity on lateral T2–T5 in female: T2–T5 white to off-white. Fine pilosity of lateral T1 in female: absent. Distribution of pilosity on metasomal terga 3–5 in female: densely present in posterior half of T3–T4, anterior half more or less glabrous, T5 setose throughout. Form of setae on lateral T2–T5: predominantly thick throughout. Pilosity of anterolateral corner of dorsal T3 in female: sparsely setose to glabrous.

##### Diagnosis.

*Scelio apospastos* is most similar to *Scelio modulus* which shares the small, striplike anteclypeus (Figs 369, 375). It differs from *Scelio modulus* in the distribution (not reaching the anterior margin, Fig. 346) and amount (relatively large patches, Fig. 346) of pilosity on the lateral metasoma. It is also very similar to *Scelio pilosilatus*, but may be separated by the absence of the fine line of pilosity along lateral T1 and the thicker more prominent patch of setae in the posteroventral quadrant of the metapleuron.

##### Etymology.

The epithet is used as a noun in apposition derived from the Greek word for separated, in reference to setae of the lateral metasoma tergites which are separated from the anterior margins by a glabrous area.

##### Link to distribution map.

http://hol.osu.edu/map-large.html?id=244616

##### Material examined.

*Holotype*, female: **ZIMBABWE**: Harare (Salisbury), Chishawasha, XII-1980, pan trap, A. Watsham, OSUC 213044 (deposited in CNCI). *Paratypes*:(55 females) **MALAWI**: 1 female, OSUC 212749 (CNCI). **SOUTH AFRICA**: 10 females, OSUC 212876, 212916, 213196 (CNCI); OSUC 213323, 213358, 213371, 213424, 213433, 213475–213476 (SANC). **SWAZILAND**: 1 female, OSUC 254697 (CNCI). **ZIMBABWE**: 43 females, OSUC 211230–211231, 211235, 211252–211253, 212104, 212111, 212129, 212132, 212137, 212143–212145, 212153, 212156–212157, 212213, 212228, 212338–212340, 212578–212579, 212642–212644, 212646, 212670, 212900, 212903, 212907, 212909, 212912, 212928, 213042, 213046, 213203, 213230, 213254 (CNCI); OSUC 212232, 212649, 212665, 213229 (OSUC).

##### Comments.

*Scelio apospastos* is very similar to *Scelio modulus*. The color of the pilosity on the lateral metasoma is also generally diagnostic between the two, with most individuals of *Scelio modulus* having finer brown pilosity on lateral T4–5, whereas in *Scelio apospastos* the pilosity is always white.

#### Scelio clypeatus

Yoder sp. n.

http://zoobank.org/2FA441A2-4634-4A7F-B694-FD1A9414B43A

urn:lsid:biosci.ohio-state.edu:osuc_concepts:244620

http://species-id.net/wiki/Scelio_clypeatus

**Figures 347–352.**
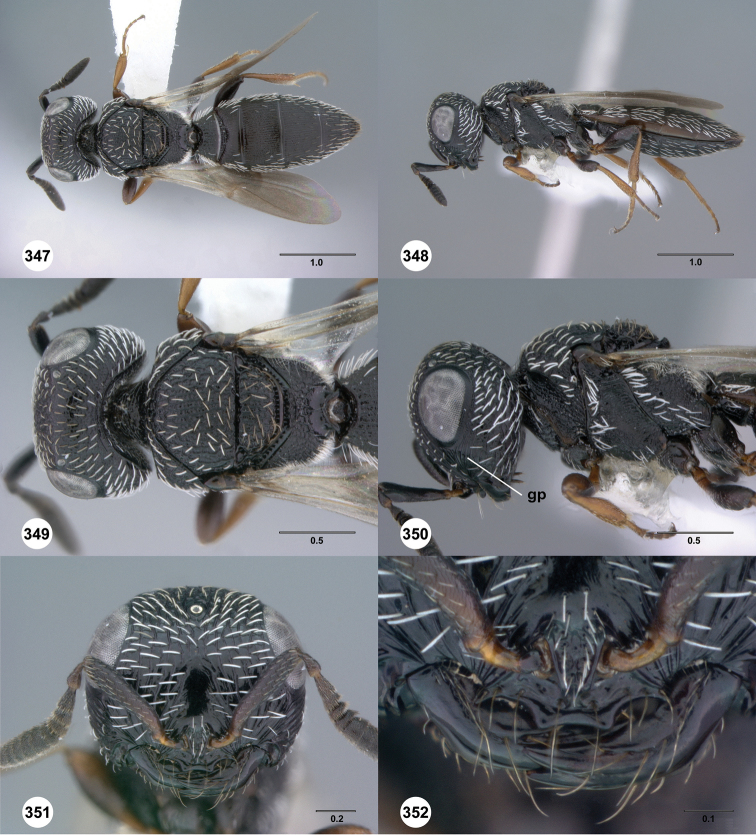
^172^ *Scelio clypeatus* sp. n., holotype female (OSUC 213552). **347** Habitus, dorsal view **348** Habitus, lateral view **349** Head and mesosoma, dorsal view **350** Head and mesosoma, lateral view **351** Head, anterior view **352** Clypeus and mouthparts, anterior view. *gp*, glabrous patch on anterior gena below eye and posterior to malar sulcus. Scale bars in millimeters.

Figures 347–352 ; Morphbank ^72^

##### Description.

Female body length: 4.20–4.50 mm (n=8). Form of sculpture of frons below anterior ocellus in female: fine dorsoventral striae with few to no reticulations. Distribution of sculpture of frons posterior to anterior ocellus in female: more or less uniform throughout. Color of pilosity of dorsomedial head in female: brown or predominantly brown. Form of anteclypeus between medial teeth in female: produced, concave medially, forming two broad lobes. Form of lateral gena below eye in anterior view in female: evenly rounded towards mandible, not bulging laterally. Sculpture of anteclypeus: smooth throughout. Sculpture of pronotal nucha in female: present throughout. Color of pilosity on mesonotum in female: predominantly white on mesoscutum, predominantly brown on mesoscutellum. Sculpture of mesoscutellum in female: predominantly irregular rugulose to reticulate. Sculpture of oxter: with prominent smooth patch. Pilosity of metapleuron overlapping or arising within posteroventral quadrant in female: 4 or more setae. Color of fore wing in female: evenly colored throughout. Color of pilosity on lateral T2–T5 in female: T2–T5 white to off-white. Fine pilosity of lateral T1 in female: present, not reaching posterior margin. Distribution of pilosity on metasomal terga 3–5 in female: more or less uniformly present throughout. Form of setae on lateral T2–T5: predominantly thick throughout. Pilosity of anterolateral corner of dorsal T3 in female: sparsely setose to glabrous.

##### Diagnosis.

This species is nearly identical to *Scelio quasiclypeatus* which shares the presence of a projected clypeus (Fig. 381) and densely setose posteroventral quadrant of the metapleuron (Figs 350, 380). It differs from *Scelio quasiclypeatus* by the absence of sculpture on the anteclypeus (Figs 351, 352) and the broadly bilobed anterior margin of the anteclypeus (truncate in *Scelio quasiclypeatus*). *Scelio apospastos* is also similar, though smaller and with the glabrous patch present along the anterior metasomal tergites T3–T5 (setose throughout in *Scelio clypeatus*).

##### Etymology.

The epithet is used as a noun in apposition derived from the Latin word for shield, in reference to the relatively large clypeus.

##### Link to distribution map.

http://hol.osu.edu/map-large.html?id=244620

##### Material examined.

*Holotype*, female: **GHANA**: camp 15 / research camp, Bia National Park, 06°32'34"N, 03°03'06"W, 190m, 22.VI–25.VI.2005, J. Gill & T. K. Philips, OSUC 213552 (deposited in OSUC). *Paratypes*: (9 females) **GABON**: 3 females, OSUC 212736, 212788, 212797 (CNCI). **GHANA**: 4 females, OSUC 213547, 213562, 213565, 213568 (OSUC). **ZIMBABWE**: 2 females, OSUC 211229, 211233 (CNCI).

##### Comments.

*Scelio clypeatus* and *Scelio quasiclypeatus* are very nearly identical, and specimens of both have been collected together. If additional intermediates are discovered they may need to be synonymized. The posterior margin of the mesopleuron (roughly the mesepimeron) is thicker and smoother than seen in most species of this group. Two individuals from Zimbabwe (OSUC 211229, 211233) are included here. They may represent an intermediate linking the two species, though their clypeus is more well developed and the glabrous patch on lateral T3–T5 is not evident.

#### Scelio concavus

Yoder sp. n.

http://zoobank.org/0465312C-8C64-465F-B498-EF6DA87F5334

urn:lsid:biosci.ohio-state.edu:osuc_concepts:244622

http://species-id.net/wiki/Scelio_concavus

**Figures 353–358.**
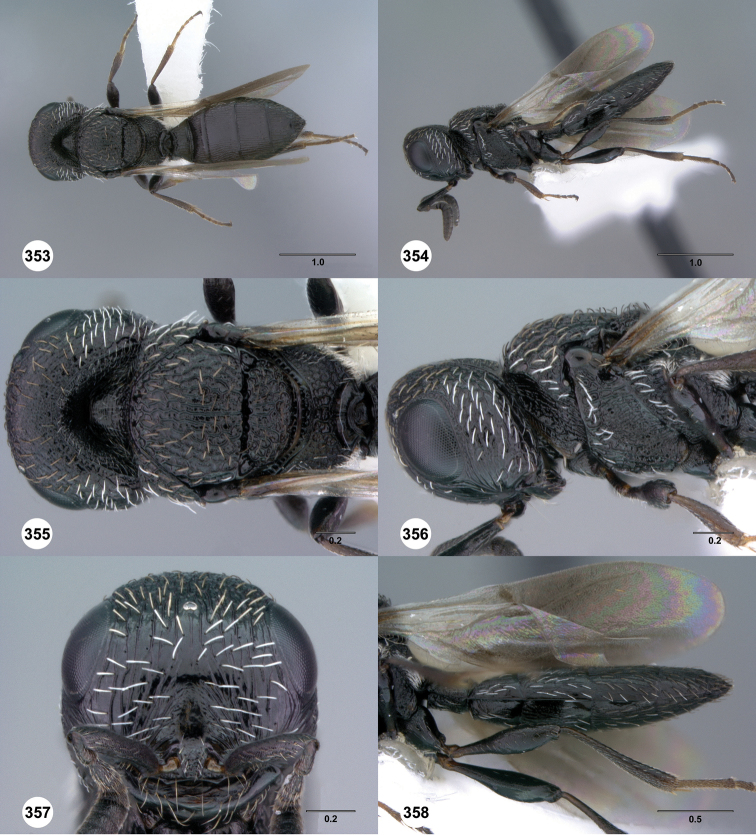
^173^ *Scelio concavus* sp. n., holotype female (OSUC 214035). **353** Habitus, dorsal view **354** Habitus, lateral view **355** Head and mesosoma, dorsal view **356** Head and mesosoma, lateral view **357** Head, anterior view **358** Metasoma, lateral view. Scale bars in millimeters.

Figures 353–358 ; Morphbank ^73^

##### Description.

Female body length: 3.59–3.85 mm (n=4). Form of sculpture of frons below anterior ocellus in female: fine dorsoventral striae with few to no reticulations. Distribution of sculpture of frons posterior to anterior ocellus in female: more or less uniform throughout. Color of pilosity of dorsomedial head in female: brown or predominantly brown. Form of anteclypeus between medial teeth in female: striplike, broadly concave. Form of lateral gena below eye in anterior view in female: evenly rounded towards mandible, not bulging laterally. Sculpture of anteclypeus: largely smooth, with few thick dorsoventral ridges. Sculpture of pronotal nucha in female: present throughout. Color of pilosity on mesonotum in female: predominantly brown throughout. Sculpture of mesoscutellum in female: predominantly irregular rugulose to reticulate. Sculpture of oxter: present throughout. Pilosity of metapleuron overlapping or arising within posteroventral quadrant in female: 1 seta; 2 setae. Color of fore wing in female: evenly colored throughout. Color of pilosity on lateral T2–T5 in female: T2 and much of T3 white, T4–T5 brown or predominantly brown. Fine pilosity of lateral T1 in female: present, extending to posterior margin. Distribution of pilosity on metasomal terga 3–5 in female: more or less uniformly present throughout. Form of setae on lateral T2–T5: uniformly thin throughout. Pilosity of anterolateral corner of dorsal T3 in female: sparsely setose to glabrous.

##### Diagnosis.

*Scelio concavus* is most similar to the other Afrotropical *walkeri*-group species with a broadly concave anteclypeus, in particular *Scelio remaudierei* which has similarly sparse and narrow pilosity on the lateral metasoma and no obliterated or smooth patches on the dorsal head. It differs from *Scelio remaudierei* by the narrower gena (strongly expanded in *Scelio remaudierei*), and less well developed medial clypeal teeth (very well developed in *Scelio remaudierei*). Sparsely setose specimens of *Scelio modulus* may be confused with *Scelio concavus*, but may be differentiated by the width and form of the clypeus (narrow and more or less truncate in *Scelio modulus*). The brown pilosity of the dorsomedial portion of the head and the mesoscutum differentiates this species from *Scelio afer*.

##### Etymology.

The epithet is used as a adjective derived from the Latin word for hollow or arched forward, in reference to the shape of the anteclypeus.

##### Link to distribution map.

http://hol.osu.edu/map-large.html?id=244622

##### Material examined.

*Holotype*, female: **KENYA**: Rift Valley Prov., Ol Pejeta Conservancy, 01°01.306'N, 36°54.818'E, 13.I–27.I.2006, malaise trap, R. Copeland, OSUC 214035 (deposited in NMKE). *Paratypes*: (3 females) **KENYA**: 2 females, OSUC 214101, 234661 (CNCI). **RWANDA**: 1 female, OSUC 212464 (CNCI).

##### Comments.

The brown pilosity of the dorsomedial part of the head and the mesonotum is striking (Fig. 355). *Scelio modulus* may be confused with the extralimital *Scelio aegyptiacus* which shares the presence of fine, brown, and sparsely distributed setae on the lateral metasoma. The fore wing of *Scelio modulus* is relatively darkly infuscate throughout, which contrasts the light/dark pattern seen in *Scelio aegyptiacus*.

#### Scelio erugatus

Yoder sp. n.

http://zoobank.org/04088880-8F52-44BD-8D33-75B0A15A5C91

urn:lsid:biosci.ohio-state.edu:osuc_concepts:244625

http://species-id.net/wiki/Scelio_erugatus

**Figures 359–364.**
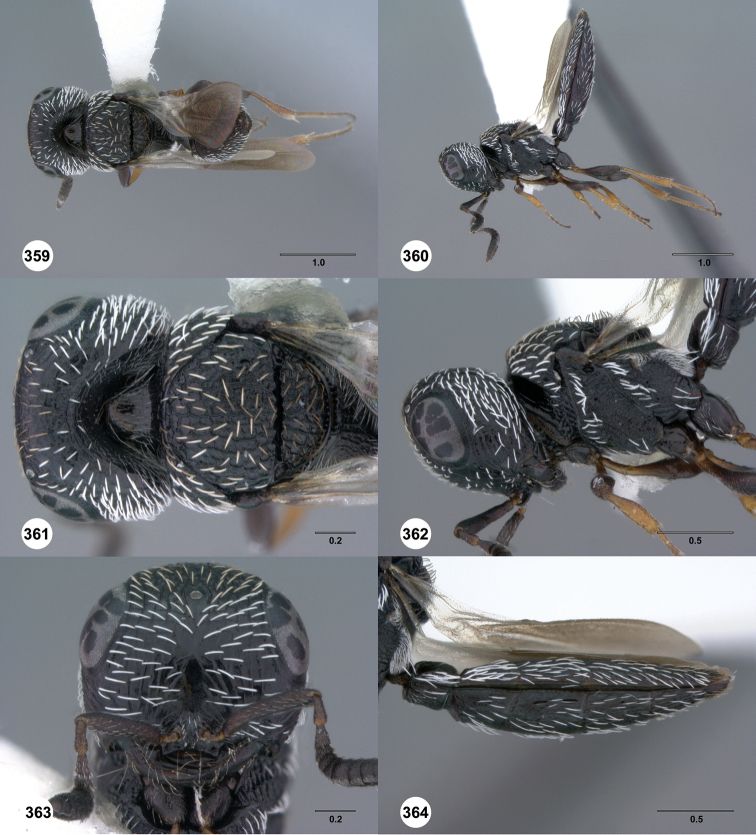
^174^ *Scelio erugatus* sp. n., holotype female (OSUC 213153). **359** Habitus, dorsal view **360** Habitus, lateral view **361** Head and mesosoma, dorsal view **362** Head and mesosoma, lateral view **363** Head, anterior view **364** Metasoma, lateral view. Scale bars in millimeters.

Figures 359–364 ; Morphbank ^74^

##### Description.

Female body length: 4.26–4.35 mm (n=3). Form of sculpture of frons below anterior ocellus in female: fine dorsoventral striae with few to no reticulations. Distribution of sculpture of frons posterior to anterior ocellus in female: more or less uniform throughout. Color of pilosity of dorsomedial head in female: brown or predominantly brown. Form of anteclypeus between medial teeth in female: striplike, broadly concave. Form of lateral gena below eye in anterior view in female: evenly rounded towards mandible, not bulging laterally. Sculpture of anteclypeus: smooth throughout. Sculpture of pronotal nucha in female: absent (smooth) in parts. Color of pilosity on mesonotum in female: predominantly brown throughout. Sculpture of mesoscutellum in female: predominantly longitudinally rugulose. Sculpture of oxter: with prominent smooth patch. Pilosity of metapleuron overlapping or arising within posteroventral quadrant in female: 4 or more setae. Color of fore wing in female: evenly colored throughout. Color of pilosity on lateral T2–T5 in female: T2–T5 white to off-white. Fine pilosity of lateral T1 in female: absent. Distribution of pilosity on metasomal terga 3–5 in female: more or less uniformly present throughout. Form of setae on lateral T2–T5: predominantly thick throughout. Pilosity of anterolateral corner of dorsal T3 in female: sparsely setose to glabrous.

##### Diagnosis.

This species is most similar to *Scelio tritus* and *Scelio afer* which share the concave anteclypeus (Figs 339, 363, 405). It differs from *Scelio tritus* by the completely smooth pronotal nucha (sculptured in *Scelio tritus*) and from *Scelio afer* by the absence of white micropilosity on the dorsal anterolateral corner of T3. *Scelio erugatus* is generally further distinguishable by the somewhat transverse pronotum and mesoscutum in combination with the somewhat scattered color pattern of pilosity of the mesoscutum (Fig. 361).

##### Etymology.

The epithet is used as an adjective derived from the Latin word for clear of wrinkles or smooth, in reference to the surface of the pronotal nucha.

##### Link to distribution map.

http://hol.osu.edu/map-large.html?id=244625

##### Material examined.

*Holotype*, female: **NIGERIA**: Oyo St., International Institute of Tropical Agriculture (IITA), Ibadan, XI-1987, screen sweeping, J. S. Noyes, OSUC 213153 (deposited in BMNH). *Paratypes*: (2 females) **GHANA**: 1 female, OSUC 213566 (OSUC). **NIGERIA**: 1 female, OSUC 213151 (CNCI).

##### Comments.

The pilosity of the mesoscutum, from anterior to posterior, consists of off-white, light brown and brown setae, the pattern and variation somewhat more notable due to the thickness of the setae. The dorsal metasoma is particularly glabrous, with almost no micropilosity. The setae on the lateral meso- and metanotum are white, moderately dense, and slightly wavy. The pronotal nucha is almost completely devoid of sculpture.

#### Scelio modulus

Yoder sp. n.

http://zoobank.org/F9695966-38BF-4B10-A6FE-681558F409EB

urn:lsid:biosci.ohio-state.edu:osuc_concepts:244621

http://species-id.net/wiki/Scelio_modulus

Figures 333 , 334 , 365–370 ; Morphbank ^75^

##### Description.

Female body length: 3.02–3.97 mm (n=20). Form of sculpture of frons below anterior ocellus in female: fine dorsoventral striae with few to no reticulations. Distribution of sculpture of frons posterior to anterior ocellus in female: more or less uniform throughout. Color of pilosity of dorsomedial head in female: brown or predominantly brown. Sculpture of ventrolateral corner of frons adjacent to malar sulcus in male: predominantly dorsoventral. Form of anteclypeus between medial teeth in female: striplike, truncate. Form of anteclypeus between medial teeth in males: striplike, truncate. Form of lateral gena below eye in anterior view in female: evenly rounded towards mandible, not bulging laterally. Sculpture of anteclypeus: smooth throughout. Sculpture of pronotal nucha in female: absent (smooth) in parts. Color of pilosity on mesonotum in female: predominantly brown throughout. Sculpture of mesoscutellum in female: predominantly irregular rugulose to reticulate. Sculpture of oxter: with prominent smooth patch. Pilosity of metapleuron overlapping or arising within posteroventral quadrant in female: 1 seta; 2 setae; 3 setae. Color of fore wing in female: evenly colored throughout. Color of fore wing in male: completely without color except at extreme base. Color of pilosity on lateral T2–T5 in female: T2 and much of T3 white, T4–T5 brown or predominantly brown. Fine pilosity of lateral T1 in female: absent; present, not reaching posterior margin; present, extending to posterior margin. Distribution of pilosity on metasomal terga 3–5 in female: more or less uniformly present throughout. Form of setae on lateral T2–T5: predominantly thick throughout. Pilosity of anterolateral corner of dorsal T3 in female: sparsely setose to glabrous. Form of medial surface of S3–S5 in males: evenly rounded throughout.

##### Diagnosis.

*Scelio modulus* is distinguished from all other species of the *walkeri*-group by the combination of the narrow, more or less truncate anteclypeus with the primarily brown pilosity of the mesonotum.

**Figures 365–370.**
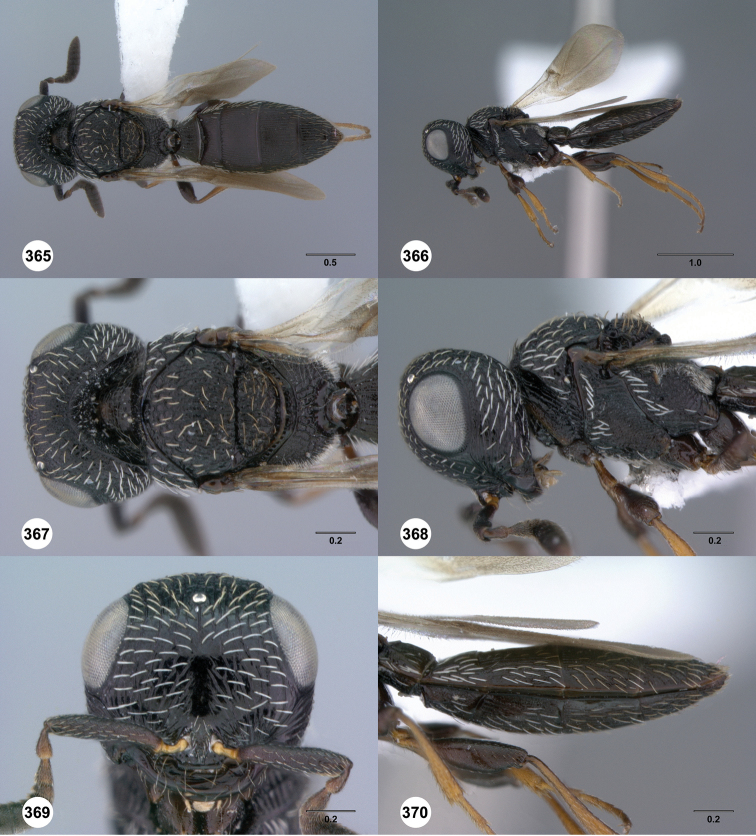
^175^ *Scelio modulus* sp. n., holotype female (OSUC 142574). **365** Habitus, dorsal view **366** Habitus, lateral view **367** Head and mesosoma, dorsal view **368** Head and mesosoma, lateral view **369** Head, anterior view **370** Metasoma, lateral view. Scale bars in millimeters.

##### Etymology.

The epithet is used as a noun in apposition derived from the Latin word for norm, model, or measure.

##### Link to distribution map.

http://hol.osu.edu/map-large.html?id=244621

##### Material examined.

*Holotype*, female: **IVORY COAST**: ~160km NW Abidjan, Lamto Research Station, 06°13'N, 05°02'W, XI-1988, malaise trap/yellow pan trap, J. S. Noyes, OSUC 142574 (deposited in BMNH). *Paratypes*: (193 females, 2 males) **BENIN**: 2 females, OSUC 211368, 212851 (CNCI). **BOTSWANA**: 1 female, OSUC 212452 (CNCI). **CAMEROON**: 5 females, OSUC 211224, 212116, 212118, 212124, 212258 (CNCI). **CENTRAL AFRICAN REPUBLIC**: 2 females, OSUC 211837, 211839 (SAMC). **GABON**: 1 female, OSUC 212796 (CNCI). **GHANA**: 5 females, OSUC 213550, 213555–213556, 213558, 213567 (OSUC). **GUINEA**: 1 female, OSUC 213121 (CNCI). **IVORY** **COAST**: 39 females, OSUC 211361, 212854, 212858, 212860, 213070, 213096 (CNCI); OSUC 142572–142573, 142575, 142577–142580, 142582–142585, 142587–142588, 142591–142592, 57142, 57144, 59112–59117, 59123–59124, 59126, 59128–59130, 64612, 64614, 64719, 64723 (OSUC). **KENYA**: 75 females, 2 males, OSUC 212950–212951, 214019–214020, 214022, 214026, 214039, 214044, 234607, 234612, 234616–234618, 234622–234624, 234627–234628, 234637, 234685–234686, 250918 (CNCI); OSUC 142672, 58973–58975, 58977, 59020–59021, 59024, 59026, 59029, 59031–59033, 59036–59037, 59041, 59045, 59047–59049, 59051–59052, 59056, 59060, 59062, 59064, 59067–59070, 59072–59075, 59079, 59081–59083, 59086, 59088–59089, 59092, 59094, 59099, 59101, 59103, 59106–59107, 59109, 70696, 70699, 70708–70709 (OSUC); OSUC 244081, 244086 (USNM). **NIGERIA**: 43 females, OSUC 202814 (AEIC); OSUC 211679, 212741–212742, 212802, 212807, 212822–212823, 212842, 212844–212845, 213139, 213141, 213176, 213178, 213182, 213184–213185, 213187–213188, 213191, 213263, 213267, 213269, 213272, 213274–213275, 250718, 250720–250721, 250723, 250726, 250728, 250773, 250792, 250987–250988, 250992, 250996, 250999, 251001–251002 (CNCI); OSUC 244206 (OSUC). **SOUTH AFRICA**: 6 females, OSUC 211206, 211208, 211211–211212, 212980 (CNCI); OSUC 254549 (MZLU). **SWAZILAND**: 1 female, OSUC 254542 (MZLU). **ZIMBABWE**: 12 females, OSUC 211249, 212202, 212216, 212575, 212638, 212816, 212826, 212832–212833, 212836, 212838, 212899 (CNCI).

##### Comments.

Like *Scelio afer*, *Scelio modulus* is widespread and polymorphic. As currently delimited this species includes several smaller series of tentatively included specimens. The core characters are the primarily brown pilosity of the mesonotum and the relatively small and more or less truncate anteclypeus. While variation occurs in characters of pilosity (see below), most specimens exhibit the following pattern: 1) mesonotum pilosity nearly all brown; 2) lateral metanotal pilosity white on T2–T3, then brown and slightly finer on T4–T5; 3) anterolateral T3 sparsely setose to glabrous; and 4) only 1–2 setae overlapping the posterolateral quadrant of the metapleuron. Several specimens with more or less white pilosity throughout the lateral metasoma are included because they are otherwise indistinguishable. Most specimens do not have prominent fine setae on lateral T2 although several small series have well-developed, completely setose lines as seen in *Scelio pilosilatus*. The sculpture of the mesoscutum is variable: most specimens are predominantly reticulate, and a minority exhibit stronger parallel longitudinal tendencies.

#### Scelio pilosilatus

Yoder sp. n.

http://zoobank.org/136D7068-F602-4AB6-8859-049F96D48C36

urn:lsid:biosci.ohio-state.edu:osuc_concepts:244617

http://species-id.net/wiki/Scelio_pilosilatus

**Figures 371–376.**
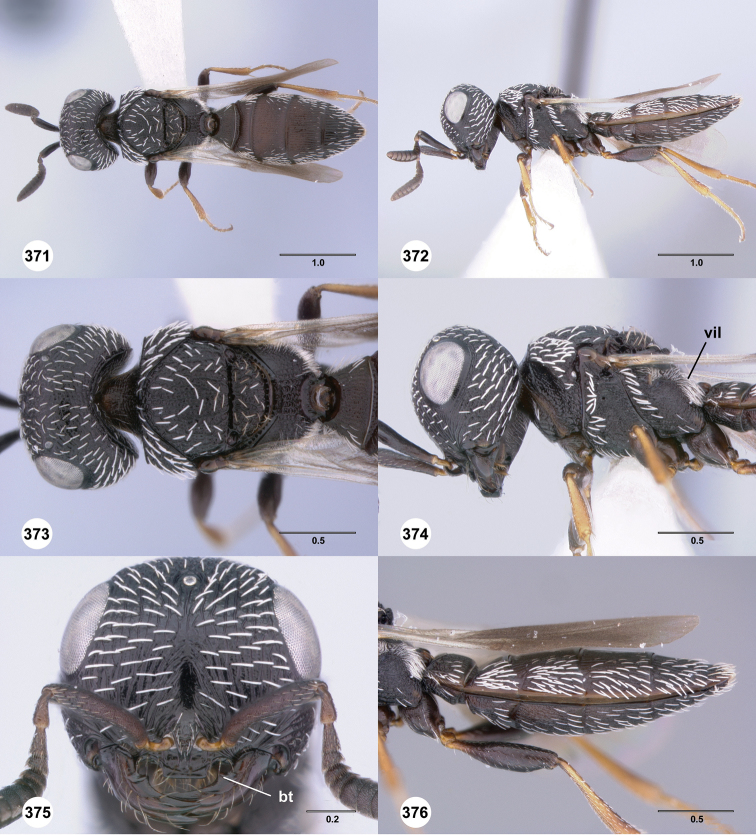
^176^ *Scelio pilosilatus* sp. n., holotype female (OSUC 211938). **371** Habitus, dorsal view **372** Habitus, lateral view **373** Head and mesosoma, dorsal view **374** Head and mesosoma, lateral view **375** Head, anterior view **376** Metasoma, lateral view. *bt*, basal tooth of mandible; *vil*, villus. Scale bars in millimeters.

Figures 371–376 ; Morphbank ^76^

##### Description.

Female body length: 3.14–4.56 mm (n=20). Form of sculpture of frons below anterior ocellus in female: fine dorsoventral striae with few to no reticulations. Distribution of sculpture of frons posterior to anterior ocellus in female: more or less uniform throughout. Color of pilosity of dorsomedial head in female: white or predominantly white. Form of anteclypeus between medial teeth in female: produced, truncate medially. Form of lateral gena below eye in anterior view in female: evenly rounded towards mandible, not bulging laterally. Sculpture of anteclypeus: smooth throughout. Sculpture of pronotal nucha in female: present throughout. Color of pilosity on mesonotum in female: predominantly white on mesoscutum, predominantly brown on mesoscutellum. Sculpture of mesoscutellum in female: predominantly irregular rugulose to reticulate. Sculpture of oxter: present throughout. Pilosity of metapleuron overlapping or arising within posteroventral quadrant in female: 1 seta; 2 setae; 3 setae; 4 or more setae. Color of fore wing in female: evenly colored throughout. Color of pilosity on lateral T2–T5 in female: T2–T5 white to off-white. Fine pilosity of lateral T1 in female: present, not reaching posterior margin; present, extending to posterior margin. Distribution of pilosity on metasomal terga 3–5 in female: more or less uniformly present throughout. Form of setae on lateral T2–T5: predominantly thick throughout. Pilosity of anterolateral corner of dorsal T3 in female: sparsely setose to glabrous.

##### Diagnosis.

*Scelio pilosilatus* differs from all Afrotropical *walkeri*-group species by the combination of the projecting anteclypeus, the dense, large patches of white pilosity that cover the lateral metasoma, the line of fine setae along the lateral margin of T1, and the relatively sparse (in appearance) pilosity of the posterolateral quadrant of the metapleuron.

##### Etymology.

The epithet is used as a noun in apposition derived from the Latin words for hair and side, in reference to the particularly well-developed patches of setae on the lateral metasoma.

##### Link to distribution map.

http://hol.osu.edu/map-large.html?id=244617

##### Material examined.

*Holotype*, female: **SOUTH AFRICA**: Limpopo Prov., 15km E Klaserie, Guernsey Farm, 19.XII–31.XII.1985, pan trap, M. Sanborne, OSUC 211938 (deposited in CNCI). *Paratypes*: (36 females) **SOUTH AFRICA**: 34 females, OSUC 211291, 211294, 211352, 211395, 211928, 211930, 211934, 212224–212225, 212242, 212252–212253, 212255, 212288, 212320, 212419, 212421–212422, 212426–212427, 212432, 212435, 212448–212449, 212743, 212868, 212873, 212983 (CNCI); OSUC 211354, 211398, 212284 (OSUC); OSUC 213346, 213434 (SANC); OSUC 244079 (USNM). **ZIMBABWE**: 2 females, OSUC 212101, 212217 (CNCI).

##### Comments.

There is a tendency in *Scelio pilosilatus* to have slightly reticulate sculpture on the frons. The fine pilosity of the lateral portion of T1 is particularly well-developed and prominent and of particular use in distinguishing the species from *Scelio apospastos*. The pilosity of the posteroventral quadrant of the metapleuron is often only 1–2 setae, one of which is particularly minute (instead of thickened, white, and large). However, we have seen specimens in which 3–5 setae are present, but these individual setae are not typically as thick and the area retains its sparsely setose appearance. While the patches of lateral metapleural pilosity are particularly large, there is a tendency for the extreme anterolateral corners to be glabrous.

#### Scelio quasiclypeatus

Yoder sp. n.

http://zoobank.org/844EEE99-9FF7-42C7-8567-50924BDF424E

urn:lsid:biosci.ohio-state.edu:osuc_concepts:244623

http://species-id.net/wiki/Scelio_quasiclypeatus

**Figures 377–382.**
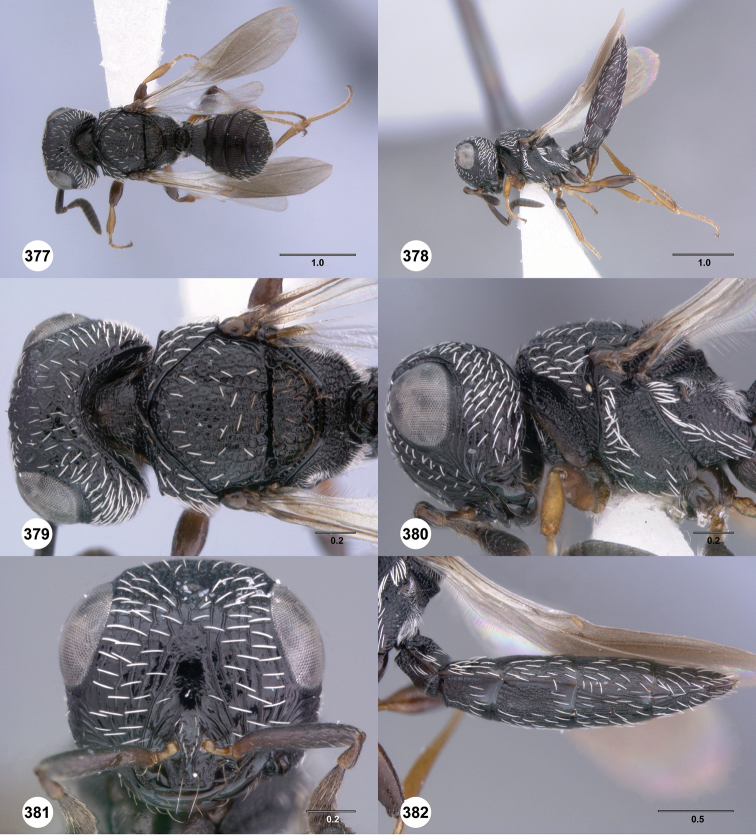
^177^ *Scelio quasiclypeatus* sp. n., paratype female (OSUC 211218). **377** Habitus, dorsal view **378** Habitus, lateral view **379** Head and mesosoma, dorsal view **380** Head and mesosoma, lateral view **381** Head, anterior view **382** Metasoma, lateral view. Scale bars in millimeters.

Figures 377–382 ; Morphbank ^77^

##### Description.

Female body length: 3.60–4.21 mm (n=16). Male body length: 3.20–3.63 mm (n=2). Form of sculpture of frons below anterior ocellus in female: fine dorsoventral striae with few to no reticulations. Distribution of sculpture of frons posterior to anterior ocellus in female: more or less uniform throughout. Color of pilosity of dorsomedial head in female: white or predominantly white. Sculpture of ventrolateral corner of frons adjacent to malar sulcus in male: predominantly dorsoventral. Form of anteclypeus between medial teeth in female: produced, truncate medially. Form of anteclypeus between medial teeth in males: produced, rounded to truncate medially. Form of lateral gena below eye in anterior view in female: evenly rounded towards mandible, not bulging laterally. Sculpture of anteclypeus: smooth throughout. Sculpture of pronotal nucha in female: present throughout. Color of pilosity on mesonotum in female: predominantly white on mesoscutum, predominantly brown on mesoscutellum. Sculpture of mesoscutellum in female: predominantly irregular rugulose to reticulate. Sculpture of oxter: present throughout. Pilosity of metapleuron overlapping or arising within posteroventral quadrant in female: 4 or more setae. Color of fore wing in female: base lighter until marginal vein, sometime strongly contrastingly so, apex darker, division relatively linear and abrupt. Color of fore wing in male: more or less evenly colored throughout. Color of pilosity on lateral T2–T5 in female: T2–T5 white to off-white. Fine pilosity of lateral T1 in female: absent. Distribution of pilosity on metasomal terga 3–5 in female: more or less uniformly present throughout. Form of setae on lateral T2–T5: predominantly thick throughout. Pilosity of anterolateral corner of dorsal T3 in female: sparsely setose to glabrous. Form of medial surface of S3–S5 in males: broadly concave, S3 posterior concavity extending into anterior half of sclerite.

##### Diagnosis.

As the name suggests, this species is most similar to *Scelio clypeatus* with which it shares the presence of a projected clypeus and densely setose posteroventral quadrant of the metapleuron. It differs by the presence of weak sculpture on the anteclypeus and the truncate anterior margin of the anteclypeus (broadly bilobed in *Scelio clypeatus*).

##### Etymology.

The epithet is used as an adjective derived from the Latin word for nearly or almost, in reference to the similarities with *Scelio clypeatus*.

##### Link to distribution map.

http://hol.osu.edu/map-large.html?id=244623

##### Material examined.

*Holotype*, female: **CAMEROON**: Nkoemvom, 30.XI.1979, malaise trap, D. Jackson, OSUC 212260 (deposited in BMNH). *Paratypes*: (15 females, 2 males) **BOTSWANA**: 1 female, OSUC 211264 (CNCI). **CAMEROON**: 4 females, OSUC 211218, 212257, 212262 (CNCI); OSUC 211223 (OSUC). **GABON**: 7 females, 2 males, OSUC 212792, 212798, 213159–213160, 213162–213164, 213169 (CNCI); OSUC 213158 (OSUC). **KENYA**: 1 female, OSUC 214144 (CNCI). **SOUTH AFRICA**: 1 female, OSUC 212458 (CNCI). **TANZANIA**: 1 female, OSUC 250958 (CNCI).

##### Comments.

The sculpture of the frons of this species is slightly more robust than typical and there are several reticulating elements, although it is still dominated by dorso-ventral elements. The fore wing of females appears only slightly lighter at the base. All specimens examined have the pilosity of the mesoscutum white except for 1 or 2 pairs of brown setae posteromedially. The gena is particularly broad, with almost no gap between the eye and the anterior of the head in lateral view. The core series from Gabon and Cameroon are nearly identical. A single specimen from Kenya (OSUC 214144) has the sculpture of the dorsal head somewhat obliterated as often seen in *Scelio afer*. The specimens from Botswana and South Africa (OSUC 211264, 212458) are slightly less robust, with pilosity less dense. The two males are tentatively included but excluded from the type material.

#### Scelio remaudierei

Ferrière

http://zoobank.org/5EDD6DE0-CDB4-46D6-A76C-C07D2A3CFD53

urn:lsid:biosci.ohio-state.edu:osuc_concepts:5314

http://species-id.net/wiki/Scelio_remaudierei

**Figures 383–388.**
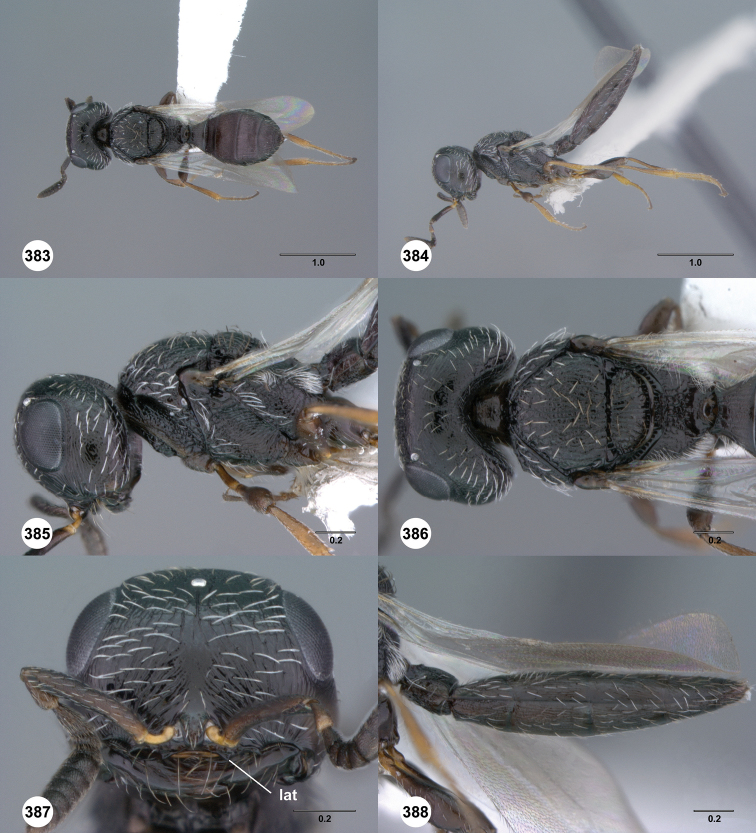
^178^ *Scelio remaudierei* Ferrière, female (OSUC 212538). **383** Habitus, dorsal view **384** Habitus, lateral view **385** Head and mesosoma, dorsal view **386** Head and mesosoma, lateral view **387** Head, anterior view **388** Metasoma, lateral view. *lat*, lateral anteclypeal tooth. Scale bars in millimeters.

Figures 383–388 ; Morphbank ^78^

Scelio remaudierei Ferrière, 1952: 116 (original description).
Scelio remaudieri : Nixon 1958: 309, 317 (keyed, spelling error).

##### Description.

Female body length: 3.14–3.71 mm (n=12). Form of sculpture of frons below anterior ocellus in female: fine dorsoventral striae with few to no reticulations. Distribution of sculpture of frons posterior to anterior ocellus in female: with at least some obliterated or reduced patches of sculpture posteriorly. Color of pilosity of dorsomedial head in female: brown or predominantly brown. Form of anteclypeus between medial teeth in female: striplike, broadly concave. Form of lateral gena below eye in anterior view in female: bulging, often surpassing eye laterally. Sculpture of anteclypeus: smooth throughout. Sculpture of pronotal nucha in female: absent (smooth) in parts. Color of pilosity on mesonotum in female: predominantly brown throughout. Sculpture of mesoscutellum in female: predominantly longitudinally rugulose. Sculpture of oxter: with prominent smooth patch. Pilosity of metapleuron overlapping or arising within posteroventral quadrant in female: none; 1 seta. Color of fore wing in female: evenly colored throughout. Color of pilosity on lateral T2–T5 in female: T2 white, T3–T5 brown. Fine pilosity of lateral T1 in female: absent. Distribution of pilosity on metasomal terga 3–5 in female: more or less uniformly present throughout. Form of setae on lateral T2–T5: uniformly thin throughout. Pilosity of anterolateral corner of dorsal T3 in female: with patch of short appressed micropilosity.

##### Diagnosis.

Within the study region this species is most similar to *Scelio afer* with which it shares a broadly concave anteclypeus and smooth sculptureless patches on the dorsal portion of the head. It differs from *Scelio afer* and all other Afrotropical species by the distinctly bulging gena. It may further be distinguished from other Afrotropical species by the fine, sparse pilosity throughout, particularly on the metasoma, a state which should only be confused with *Scelio concavus*.

##### Link to distribution map.

http://hol.osu.edu/map-large.html?id=5314

##### Associations.

Emerged from egg of *Acrida turrita* (Linnaeus) [Orthoptera: Acrididae]; emerged from egg of Acrididae [Orthoptera: Acrididae]; emerged from egg of *Eyprepocnemis plorans plorans* (Charpentier) [Orthoptera: Acrididae, as *Euprepocnemis senegalensis* Bolívar)]; emerged from egg of *Locusta cinerascens migratorioides* (Reiche & Fairmaire) [Orthoptera: Acrididae, as *Locusta migratoria migratorioides*]; emerged from egg of *Hilethera* nigerica (Uvarov) [Orthoptera: Acrididae, as *Paracinema tricolor nigerica* (Uvarov)].

##### Material examined.

*Lectotype* (**present designation**), **MALI**: Dogo, II-1950, Remaudière, BMNH(E)#790429 (deposited in BMNH). *Paralectoype*, female, **MALI**: Dogo, II-1950, Remaudière, OSUC 173945 (deposited in MNHN). *Other material*: (12 females, 5 unknowns) **EGYPT**: 3 females, OSUC 244121–244123 (USNM). **KENYA**: 1 female, OSUC 59038 (OSUC). **MALI**: 5 unknowns, BMNH(E)#790424–790428 (BMNH). **NIGER**: 1 female, OSUC 212203 (CNCI). **SOUTH** **AFRICA**: 4 females, OSUC 212538, 212542, 250686 (CNCI); OSUC 213385 (SANC). **UNITED ARAB EMIRATES**: 2 females, OSUC 214056, 214073 (CNCI). **YEMEN**: 1 female, OSUC 254783 (CNCI).

##### Comments.

We initially interpreted specimens of *Scelio remaudierei* as a monstrous form of *Scelio afer*. However, specimens with nearly identical morphology from a broad distribution suggests that this morphotype warrants species status. Our material examined matches well with the type (see http://www.waspweb.org/Platygastroidea/Platygastridae/Scelioninae/Scelio/Scelio_remaudierei.htm).

*Scelio aegyptiacus* shares the relatively sparse and fine pilosity of the metasoma, the predominantly longitudinally striate sculpture of the mesoscutellum, the partially obliterated sculpture of the dorsum of the head (also shared with *Scelio afer*), and a relatively broad, but not as bulging gena. Given the available material the two species *Scelio remaudierei* and *Scelio aegyptiacus* are reliably separated by the presence (*Scelio remaudierei*) of sculpture on the lateral sternites (smooth in *Scelio aegyptiacus*) and by the broader gena in *Scelio remaudierei*. Nixon’s (1958) concept of *Scelio remaudierei* appears to be broader and combines of the concepts of *Scelio remaudierei* and *Scelio afer* presented here. *Scelio popovi* Nixon (1958) may be a synonym of *Scelio aegyptiacus*. We do not formally proposed this at this point, but the issue should be reviewed in a future revision of the North African and middle Eastern *Scelio*.

The dorsoventral striae of the frons are somewhat more closely packed together in *Scelio remaudierei* than in most other species. The patches of obliterated sculpture on the dorsal head are smaller than those observed in *Scelio afer*, and the sculpture is also somewhat finer and more dense in *Scelio remaudierei*. The mandibles are very broad. It is difficult to discern if there are two setal types on the lateral portion of T2. T5 in females has a relatively well-developed median crease, and the lateral surface appears to be slightly pinched.

#### Scelio retifrons

Yoder sp. n.

http://zoobank.org/1322728C-1C1A-4AB5-BBCE-47BAAD7D588C

urn:lsid:biosci.ohio-state.edu:osuc_concepts:244624

http://species-id.net/wiki/Scelio_retifrons

**Figures 389–394.**
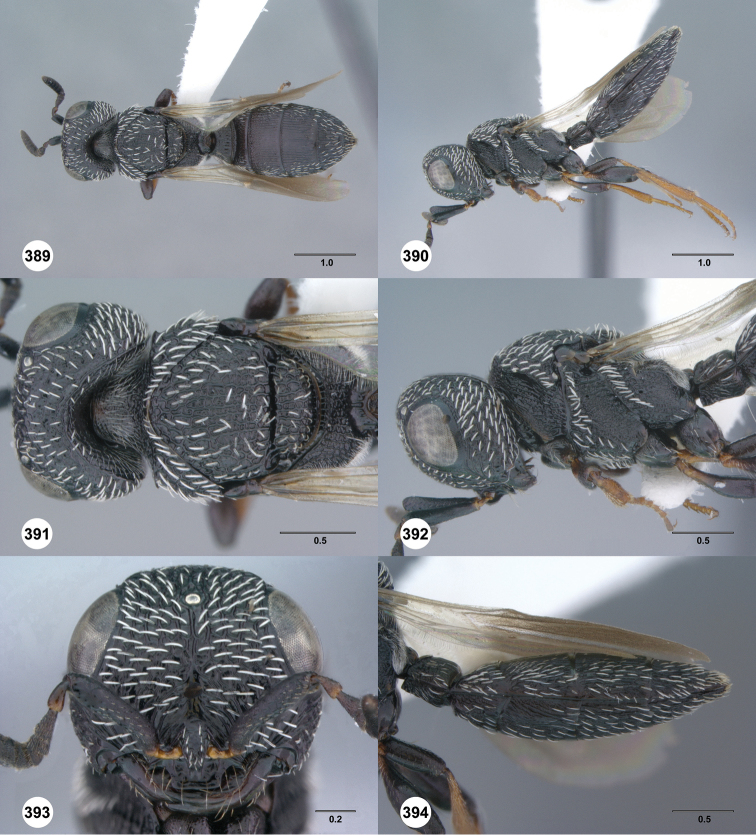
^179^ *Scelio retifrons* sp. n., holotype female (OSUC 213170). **389** Habitus, dorsal view **390** Habitus, lateral view **391** Head and mesosoma, dorsal view **392** Head and mesosoma, lateral view **393** Head, anterior view **394** Metasoma, lateral view. Scale bars in millimeters.

Figures 389–394 ; Morphbank ^79^

##### Description.

Female body length: 5.15 mm (n=1). Form of sculpture of frons below anterior ocellus in female: robustly reticulate. Distribution of sculpture of frons posterior to anterior ocellus in female: more or less uniform throughout. Color of pilosity of dorsomedial head in female: white or predominantly white. Form of anteclypeus between medial teeth in female: produced, truncate medially. Form of lateral gena below eye in anterior view in female: evenly rounded towards mandible, not bulging laterally. Sculpture of anteclypeus: smooth throughout. Sculpture of pronotal nucha in female: present throughout. Color of pilosity on mesonotum in female: predominantly white throughout. Sculpture of mesoscutellum in female: predominantly irregular rugulose to reticulate. Sculpture of oxter: present throughout. Pilosity of metapleuron overlapping or arising within posteroventral quadrant in female: 2 setae. Color of fore wing in female: evenly colored throughout. Color of pilosity on lateral T2–T5 in female: T2–T5 white to off-white. Fine pilosity of lateral T1 in female: present, not reaching posterior margin. Distribution of pilosity on metasomal terga 3–5 in female: more or less uniformly present throughout. Form of setae on lateral T2–T5: predominantly thick throughout. Pilosity of anterolateral corner of dorsal T3 in female: with patch of short appressed micropilosity.

##### Diagnosis.

*Scelio retifrons* is most similar to *Scelio pilosilatus* with which it share the dense pilosity of the lateral metasoma and the projecting anteclypeus (Fig. 393). It differs from *Scelio pilosilatus* and all other Afrotropical *walkeri*-group species by the reticulate sculpture of the frons (Fig. 393).

##### Etymology.

The epithet is used as a noun in apposition derived from the Latin words for net and front, in reference to the sculpture of the frons.

##### Link to distribution map.

http://hol.osu.edu/map-large.html?id=244624

##### Material examined.

*Holotype*, female: **IVORY COAST**: Lamto Research Station, 06°13'N, 05°02'W, 5.IV.1988, malaise trap, OSUC 213170 (deposited in CNCI).

##### Comments.

*Scelio retifrons* is presently known from only a single specimen. However, among Afrotropical *walkeri*-group species it is unique for its size (the only species > 5 mm long) and sculpture of the frons. The frons has strong dorsoventral elements, but there are also relatively well-developed reticulations among these elements. Similar reticulate sculpture of the frons is known in species from the Arabian peninsula. The T3 anterolateral setal patch (Fig. 394) is particularly well developed and the posteroventral quadrant of the metapleuron has only 2 setae (Fig. 392).

#### Scelio striatus

Priesner

http://zoobank.org/688B21CF-5849-4F4F-B958-D9F0A417119E

urn:lsid:biosci.ohio-state.edu:osuc_concepts:5338

http://species-id.net/wiki/Scelio_striatus

**Figures 395–400.**
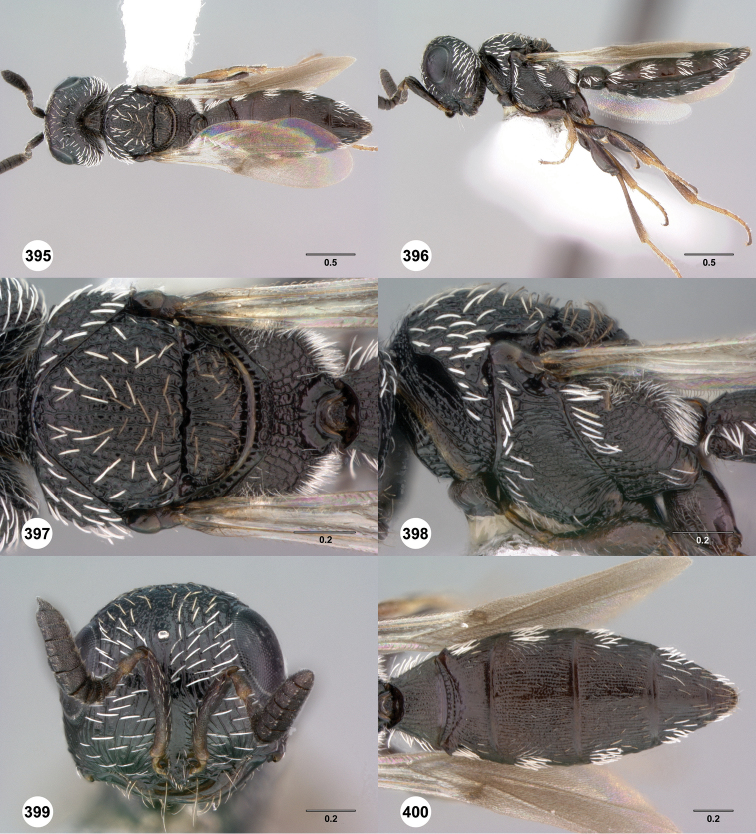
^180^ *Scelio striatus* Priesner, female (OSUC 212944). **395** Habitus, dorsal view **396** Habitus, lateral view **397** Mesosoma, dorsal view **398** Mesosoma, lateral view **399** Head, anterior view **400** Metasoma, dorsal view. Scale bars in millimeters.

Figures 395–400 ; Morphbank ^80^

Scelio striatus Priesner, 1951: 144 (original description); Kononova and Kozlov 2008: 139, 153 (description, keyed).
Scelio gaudens Nixon, 1958: 309, 317 (original description, keyed); Masner 1965: 93 (type information), syn. n.
Scelio striatus http://zoobank.org/4FF01A88-A8EF-4464-BFD3-1E836328215F
Scelio striatus urn:lsid:biosci.ohio-state.edu:osuc_concepts:5241

##### Description.

Female body length: 2.95–4.09 mm (n=20). Male body length: 2.88–3.31 mm (n=11). Form of sculpture of frons below anterior ocellus in female: fine dorsoventral striae with few to no reticulations. Distribution of sculpture of frons posterior to anterior ocellus in female: more or less uniform throughout. Color of pilosity of dorsomedial head in female: brown or predominantly brown. Sculpture of ventrolateral corner of frons adjacent to malar sulcus in male: predominantly dorsoventral. Form of anteclypeus between medial teeth in female: striplike, broadly concave. Form of anteclypeus between medial teeth in males: deeply concave medially, bounded by two projecting rounded lobes. Form of lateral gena below eye in anterior view in female: evenly rounded towards mandible, not bulging laterally. Sculpture of anteclypeus: smooth throughout. Sculpture of pronotal nucha in female: present throughout. Color of pilosity on mesonotum in female: predominantly brown throughout; predominantly white on mesoscutum, predominantly brown on mesoscutellum. Sculpture of mesoscutellum in female: predominantly longitudinally rugulose. Sculpture of oxter: with prominent smooth patch. Pilosity of metapleuron overlapping or arising within posteroventral quadrant in female: 1 seta; 2 setae; 3 setae; 4 or more setae. Color of fore wing in female: evenly colored throughout. Color of fore wing in male: more or less evenly colored throughout. Color of pilosity on lateral T2–T5 in female: T2–T5 white to off-white. Fine pilosity of lateral T1 in female: present, not reaching posterior margin. Distribution of pilosity on metasomal terga 3–5 in female: pilosity densely present in anterior half, posterior half more or less glabrous. Form of setae on lateral T2–T5: predominantly thick throughout. Pilosity of anterolateral corner of dorsal T3 in female: sparsely setose to glabrous. Form of medial surface of S3–S5 in males: flat to slightly concave, S3 only slightly concave posteriorly.

##### Diagnosis.

*Scelio striatus* is easily distinguished from all other Afrotropical *walkeri*-group species by the setal pattern on the lateral tergites in which the pilosity is absent in the posterior half and dense in anterior half (Figs 395, 396, 400). Males are recognized among all Afrotropical *walkeri*-group species by the strongly concave medial anteclypeus bound by strong rounded projections (see Fig. 17 in Nixon 1958).

##### Link to distribution map.

http://hol.osu.edu/map-large.html?id=5338

##### Material examined.

*Holotype* *Scelio striatus*, female: **EGYPT**: Al Qahirah Gov., Al Ma’adi (Meadi), 10.VII.1933, H. Priesner, USNM Type No. 70883 (deposited in USNM). *Paratypes*, *Scelio gaudens*: **MALI**: 5 unrecorded sex, BMNH(E)#790433–790437 (BMNH). *Other material*: (104 females, 11 males, 13 unknowns) **BOTSWANA**: 1 female, OSUC 212416 (CNCI). **ERITREA**: 1 female, OSUC 210358 (MCSN). **ETHIOPIA**: 1 unknown, BMNH(E)#790449 (BMNH). **IVORY** **COAST**: 8 females, OSUC 213015–213016, 213061, 213063, 213067, 213225, 213238 (CNCI); OSUC 213013 (OSUC). **KENYA**: 20 females, 1 male, OSUC 212359–212360, 212362, 214095, 214112, 214143, 214145, 214155–214156, 214158, 234650, 234657, 234660, 234662, 234696, 234700 (CNCI); OSUC 214097, 59022, 59028, 59096 (OSUC); OSUC 248099 (USNM). **NAMIBIA**: 1 unknown, BMNH(E)#790441 (BMNH). **NIGERIA**: 2 females, OSUC 211377, 250998 (CNCI). **SOMALIA**: 5 females, OSUC 211272, 212130, 212606–212607, 212904 (CNCI). **SOUTH AFRICA**: 7 females, 1 male, 10 unknowns, BMNH(E)#790438–790440, 790442–790448 (BMNH); OSUC 211273, 211292, 212352, 212669 (CNCI); OSUC 203963 (OSUC); OSUC 213365, 213381, 213463 (SANC). **TANZANIA**: 3 females, 1 male, OSUC 212891, 212966–212967, 212974 (CNCI). **UGANDA**: 1 unknown, BMNH(E)#790450 (BMNH). **UNITED ARAB EMIRATES**: 4 females, OSUC 214059, 214072, 214074–214075 (CNCI). **YEMEN**: 22 females, 8 males, OSUC 212471, 212473–212474, 212490, 212498, 212501, 212940, 212944, 250678, 250879, 250942–250943, 251033, 251039, 251044, 251051–251052, 251054, 251058, 254664, 254667, 254671, 254685, 254691, 254777, 254779, 254801 (CNCI); OSUC 212499, 251056 (OSUC); UCRC ENT 171006 (UCRC). **ZIMBABWE**: 31 females, OSUC 211234, 211237–211238, 211245, 211248, 212097–212098, 212139–212140, 212403, 212409, 212570–212571, 212573, 212577, 212580–212581, 212585–212587, 212589, 212595, 212597, 212600, 213003, 213009, 213011, 213055–213056, 213251 (CNCI); OSUC 211236 (OSUC).

##### Comments.

*Scelio striatus* is the most easily diagnosed member of the *walkeri*-group based on the distinctive metasomal pilosity. The association of males with females is largely based on observations of Nixon (1958), who treated the species as *Scelio gaudens*. A small series of females (OSUC 203963, 212669, 214143, 214156, 214158, 234650, 234696, 234700) is tentatively included here. They differ slightly in that pilosity of the lateral metasoma is slightly thinner. There are usually 2–3 setae in the posteroventral quadrant of the metapleuron of females. The sculpture of the dorsum of the head is more densely reticulate than in most species.

#### Scelio tritus

Yoder sp. n.

http://zoobank.org/615C35B7-06C5-4E1F-B6D4-6EBE93305745

urn:lsid:biosci.ohio-state.edu:osuc_concepts:244618

http://species-id.net/wiki/Scelio_tritus

**Figures 401–406.**
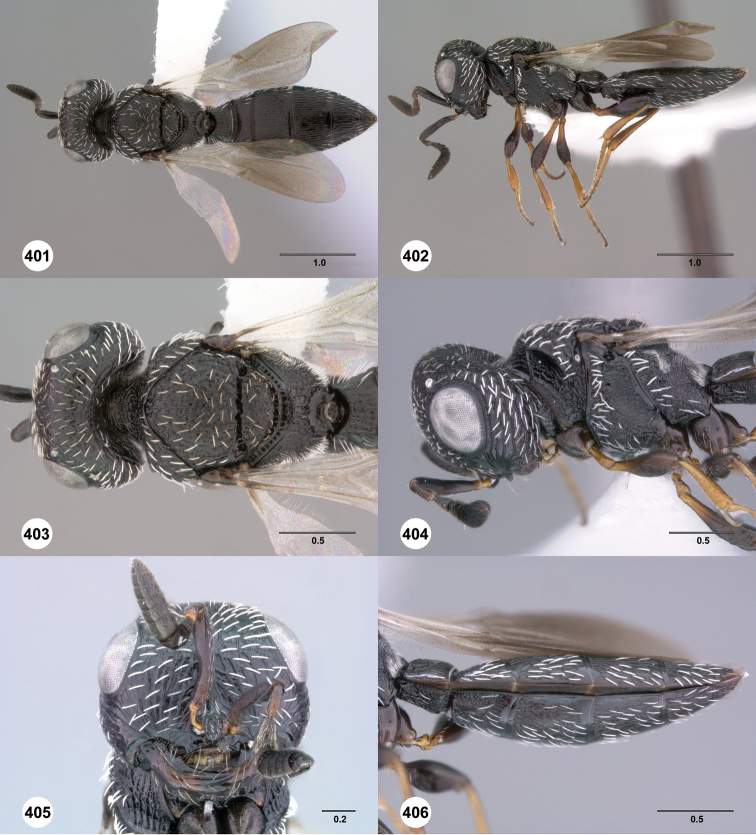
^181^ *Scelio tritus* sp. n. **401–402** paratype female (OSUC 212466) **403–406** holotype female (CASENT 8106001) **401** Habitus, dorsal view **402** Habitus, lateral view **403** Head and mesosoma, dorsal view **404** Head and mesosoma, lateral view **405** Head, anterior view **406** Metasoma, lateral view. Scale bars in millimeters.

Figures 5 , 401–406 ; Morphbank ^81^

##### Description.

Female body length: 3.30–4.36 mm (n=19). Male body length: 3.20–4.15 mm (n=20). Form of sculpture of frons below anterior ocellus in female: fine dorsoventral striae with few to no reticulations. Distribution of sculpture of frons posterior to anterior ocellus in female: more or less uniform throughout; with at least some obliterated or reduced patches of sculpture posteriorly. Color of pilosity of dorsomedial head in female: white or predominantly white; brown or predominantly brown. Sculpture of ventrolateral corner of frons adjacent to malar sulcus in male: predominantly dorsoventral. Form of anteclypeus between medial teeth in female: striplike, broadly concave. Form of anteclypeus between medial teeth in males: produced, rounded to truncate medially. Form of lateral gena below eye in anterior view in female: evenly rounded towards mandible, not bulging laterally. Sculpture of anteclypeus: largely smooth, with few thick dorsoventral ridges. Sculpture of pronotal nucha in female: present throughout. Color of pilosity on mesonotum in female: predominantly white throughout; predominantly brown throughout; predominantly white on mesoscutum, predominantly brown on mesoscutellum. Sculpture of mesoscutellum in female: predominantly irregular rugulose to reticulate. Sculpture of oxter: with prominent smooth patch. Pilosity of metapleuron overlapping or arising within posteroventral quadrant in female: 2 setae; 3 setae; 4 or more setae. Color of fore wing in female: evenly colored throughout. Color of fore wing in male: more or less evenly colored throughout. Color of pilosity on lateral T2–T5 in female: T2–T5 white to off-white. Fine pilosity of lateral T1 in female: absent. Distribution of pilosity on metasomal terga 3–5 in female: more or less uniformly present throughout. Form of setae on lateral T2–T5: predominantly thick throughout. Pilosity of anterolateral corner of dorsal T3 in female: sparsely setose to glabrous. Form of medial surface of S3–S5 in males: broadly concave, S3 posterior concavity extending into anterior half of sclerite.

##### Diagnosis.

This species is most similar to *Scelio afer* with which it shares the broadly concave anteclypeus (Fig. 405) and, in some specimens, the slightly obliterated sculpture of the dorsal head (as in Fig. 337). It differs from *Scelio afer* by the sculptured dorsal pronotal nucha (vs. clearly evident smooth patches), the concolorous pilosity of the dorsomedial head and mesonotum (mesoscutellar pilosity distinctly darker in *Scelio afer*), and the absence of micropilosity on the anterolateral portion of T3.

##### Etymology.

The epithet is used as a adjective derived from the Latin word for familiar or commonplace, in reference to its relatively abundance within Madagascar.

##### Link to distribution map.

http://hol.osu.edu/map-large.html?id=244618

##### Associations.

Emerged from egg of Acrididae [Orthoptera: Acrididae]; emerged from ootheca of *Heteracris zolotarevskyi* Dirsh [Orthoptera: Acrididae]; emerged from leaf of *Kalanchoe delagoensis* Eckl. & Zeyh. [Rosales: Crassulaceae].

##### Material examined.

*Holotype*, female: **MADAGASCAR**: Mahajanga Auto. Prov., 3.4km (93°) E Bekopaka, Vazimba Tomb, tropical dry forest, BLF4233, Tsingy de Bemaraha National Park, 19°08'31"S, 44°49'41"E, 50m, 6.XI–10.XI.2001, malaise trap, Fisher, Griswold et al., CASENT 8106001 (deposited in CASC). *Paratypes*: **MADAGASCAR**: 47 females, 62 males, CASENT 2042629–2042634, 2042891, 2043301, 2043333, 2043344, 2043588, 2043590, 2043592, 2043953, 2043994, 2043996, 2132180, 2132216, 2132285, 2132310, 2132322, 2132343, 2132579, 2132634, 2132703, 2132764, 2132827, 2132851, 2132874, 2133074, 2133083, 2133125, 2133183, 2133192, 2133204, 2133370, 2133492, 2133495, 2133497–2133498, 2133505–2133506, 2133523, 2133532, 2133599, 2133601, 2133656, 2133660, 2133681, 2133707, 2133773, 2133783, 2133802, 2133859, 2133861, 2133955, 2133985, 2134007, 2134011, 2134035, 2134179, 2134236, 2134249, 2134353, 2134405, 2134587, 2134745, 2134778, 2134846, 8097474, 8106003, 8106062, 8106076, 8106176, 8106263, 8106332, 8106342, 8106508, 8106521, 8106532, 8106535, 8106545, 8106562, 8106568, 8106638, 8106734, 8106774, 8106818, 8106852, 8106896, OSUC 212014, OSUC 212017, OSUC 212025, OSUC 212028, OSUC 212036, OSUC 212037 (CASC); OSUC 212466, 212468, 212530, 212532, 234770 (CNCI); CASENT 2043586, 2133234, 2134010, 2134715, OSUC 142625, OSUC 211868, OSUC 60591 (OSUC); OSUC 213378 (SANC). *Other material*: **MADAGASCAR**: 7 females, 18 males, OSUC 244181a, 244181b–244184a, 244184b–244185, 244187–244188b, 244188c–244189c, 244191–244193a, 244193c, 261194, 261196–261198, 261381–261386 (MNHN).

##### Comments.

*Scelio tritus* is not known from mainland Africa and is only one of two *walkeri*-group species in Madagascar (see also Comments on *Scelio afer*). *Scelio tritus* is very similar to the widespread *Scelio afer*, however, congruence in several subtle characters (see Diagnosis) can be used to separate the two. While a few specimens have similarly obliterated sculpture of the dorsal head as seen in *Scelio afer*, the majority do not. The color of pilosity on the dorsal head and mesonotum is more or less concolorous for a given specimen, but varies among individuals. This general pattern contrasts that of individuals of *Scelio afer* which always have the mesoscutellar pilosity darker than that of the mesoscutum. The pronotal nucha is relatively robustly sculptured as compared to other species in this group. The pilosity of the lateral metasoma tends to be less dense or absent towards the anterior and posterior of each tergite (Fig. 406). A small number of specimens (CASENT 2134007, 2134010, 2134011, and OSUC 142625, 213378) are notable for their dense, completely white and somewhat thicker pilosity. These otherwise fit well with the remaining material.

The five specimens from the Muséum National d’Histoire Naturelle with letters following the identifiers are mounted with other specimens on the same pins. They are excluded from the type series in order to try to avoid future confusions.

## Supplementary Material

XML Treatment for Scelio albatus

XML Treatment for Scelio aphares

XML Treatment for Scelio ardelio

XML Treatment for Scelio bayanga

XML Treatment for Scelio chapmani

XML Treatment for Scelio copelandi

XML Treatment for Scelio dupondi

XML Treatment for Scelio exophthalmus

XML Treatment for Scelio janseni

XML Treatment for Scelio mauritanicus

XML Treatment for Scelio phaeoprora

XML Treatment for Scelio taylori

XML Treatment for Scelio fremo

XML Treatment for Scelio howardi

XML Treatment for Scelio igland

XML Treatment for Scelio ructo

XML Treatment for Scelio scomma

XML Treatment for Scelio ululo

XML Treatment for Scelio zolotarevskyi

XML Treatment for Scelio balo

XML Treatment for Scelio bubulo

XML Treatment for Scelio cano

XML Treatment for Scelio crepo

XML Treatment for Scelio gemo

XML Treatment for Scelio grunnio

XML Treatment for Scelio latro

XML Treatment for Scelio mutio

XML Treatment for Scelio susurro

XML Treatment for Scelio tono

XML Treatment for Scelio tristis

XML Treatment for Scelio effervesco

XML Treatment for Scelio destico

XML Treatment for Scelio memorabilis

XML Treatment for Scelio philippinensis

XML Treatment for Scelio pipilo

XML Treatment for Scelio aurantium

XML Treatment for Scelio impostor

XML Treatment for Scelio ipomeae

XML Treatment for Scelio ntchisii

XML Treatment for Scelio somaliensis

XML Treatment for Scelio transtrum

XML Treatment for Scelio harinhalai

XML Treatment for Scelio irwini

XML Treatment for Scelio obscuripennis

XML Treatment for Scelio parkeri

XML Treatment for Scelio simoni

XML Treatment for Scelio simonolus

XML Treatment for Scelio vannoorti

XML Treatment for Scelio afer

XML Treatment for Scelio apospastos

XML Treatment for Scelio clypeatus

XML Treatment for Scelio concavus

XML Treatment for Scelio erugatus

XML Treatment for Scelio modulus

XML Treatment for Scelio pilosilatus

XML Treatment for Scelio quasiclypeatus

XML Treatment for Scelio remaudierei

XML Treatment for Scelio retifrons

XML Treatment for Scelio striatus

XML Treatment for Scelio tritus

## Appendix 1

Uri Table of HAO morphological terms (doi: 10.3897/zookeys.380.5755.app1) File format: Open Office text file (odt).

## Appendix 2

Darwin Core Archive file of Afrotropical *Scelio* taxonomic names. (doi: 10.3897/zookeys.380.5755.app2) File format: Win Zip archive (zip).

## Appendix 3

Darwin Core Archive file of occurrences of Afrotropical *Scelio*. (doi: 10.3897/zookeys.380.5755.app3) File format: Win Zip archive (zip).

## Endnotes

1 http://biocol.org/urn:lsid:biocol.org:col:1008

2 http://biocol.org/urn:lsid:biocol.org:col:32972

3 http://biocol.org/urn:lsid:biocol.org:col:1009

4 http://biocol.org/urn:lsid:biocol.org:col:1010

5 http://biocol.org/urn:lsid:biocol.org:col:33071

6 http://biocol.org/urn:lsid:biocol.org:col:33138

7 http://biocol.org/urn:lsid:biocol.org:col:1013

8 http://biocol.org/urn:lsid:biocol.org:col:33355

9 http://biocol.org/urn:lsid:biocol.org:col:33527

10 http://biocol.org/urn:lsid:biocol.org:col:33900

11 http://biocol.org/urn:lsid:biocol.org:col:33864

12 http://biocol.org/urn:lsid:biocol.org:col:33943

13 http://biocol.org/urn:lsid:biocol.org:col:34022

14 http://biocol.org/urn:lsid:biocol.org:col:1014

15 http://biocol.org/urn:lsid:biocol.org:col:34210

16 http://biocol.org/urn:lsid:biocol.org:col:1018

17 http://biocol.org/urn:lsid:biocol.org:col:34248

18 http://biocol.org/urn:lsid:biocol.org:col:34336

19 http://biocol.org/urn:lsid:biocol.org:col:34411

20 http://biocol.org/urn:lsid:biocol.org:col:34424

21 http://biocol.org/urn:lsid:biocol.org:col:34026

22 http://morphbank.net/?id=787857

23 http://morphbank.net/?id=787861

24 http://morphbank.net/?id=787860

25 http://morphbank.net/?id=787863

26 http://morphbank.net/?id=787879

27 http://morphbank.net/?id=787855

28 http://morphbank.net/?id=787859

29 http://morphbank.net/?id=787862

30 http://morphbank.net/?id=787856

31 http://morphbank.net/?id=787858

32 http://morphbank.net/?id=787890

33 http://morphbank.net/?id=792630

34 http://morphbank.net/?id=787846

35 http://morphbank.net/?id=787850

36 http://morphbank.net/?id=787829

37 http://morphbank.net/?id=787833

38 http://morphbank.net/?id=792631

39 http://morphbank.net/?id=787836

40 http://morphbank.net/?id=792632

41 http://morphbank.net/?id=787848

42 http://morphbank.net/?id=787843

43 http://morphbank.net/?id=787845

44 http://morphbank.net/?id=787847

45 http://morphbank.net/?id=787844

46 http://morphbank.net/?id=787842

47 http://morphbank.net/?id=787835

48 http://morphbank.net/?id=787837

49 http://morphbank.net/?id=787840

50 http://morphbank.net/?id=787849

51 http://morphbank.net/?id=829257

52 http://morphbank.net/?id=792633

53 http://morphbank.net/?id=787839

54 http://morphbank.net/?id=787827

55 http://morphbank.net/?id=787841

56 http://morphbank.net/?id=787838

57 http://morphbank.net/?id=787824

58 http://morphbank.net/?id=787821

59 http://morphbank.net/?id=787822

60 http://morphbank.net/?id=787825

61 http://morphbank.net/?id=787826

62 http://morphbank.net/?id=787823

63 http://morphbank.net/?id=787817

64 http://morphbank.net/?id=787819

65 http://morphbank.net/?id=787816

66 http://morphbank.net/?id=787818

67 http://morphbank.net/?id=787812

68 http://morphbank.net/?id=787813

69 http://morphbank.net/?id=787814

70 http://morphbank.net/?id=792637

71 http://morphbank.net/?id=787808

72 http://morphbank.net/?id=787805

73 http://morphbank.net/?id=787804

74 http://morphbank.net/?id=787807

75 http://morphbank.net/?id=787802

76 http://morphbank.net/?id=787801

77 http://morphbank.net/?id=787811

78 http://morphbank.net/?id=787810

79 http://morphbank.net/?id=787806

80 http://morphbank.net/?id=787809

81 http://morphbank.net/?id=792638

82 http://lsid.tdwg.org/urn:lsid:biosci.ohio-state.edu:osuc_pubs:68

83 http://lsid.tdwg.org/urn:lsid:biosci.ohio-state.edu:osuc_pubs:104

84 http://lsid.tdwg.org/urn:lsid:biosci.ohio-state.edu:osuc_pubs:127

85 http://lsid.tdwg.org/urn:lsid:biosci.ohio-state.edu:osuc_pubs:624

86 http://lsid.tdwg.org/urn:lsid:biosci.ohio-state.edu:osuc_pubs:9560

87 http://lsid.tdwg.org/urn:lsid:biosci.ohio-state.edu:osuc_pubs:955

88 http://lsid.tdwg.org/urn:lsid:biosci.ohio-state.edu:osuc_pubs:960

89 http://lsid.tdwg.org/urn:lsid:biosci.ohio-state.edu:osuc_pubs:23466

90 http://lsid.tdwg.org/urn:lsid:biosci.ohio-state.edu:osuc_pubs:21725

91 http://lsid.tdwg.org/urn:lsid:biosci.ohio-state.edu:osuc_pubs:842

92 http://lsid.tdwg.org/urn:lsid:biosci.ohio-state.edu:osuc_pubs:308

93 http://lsid.tdwg.org/urn:lsid:biosci.ohio-state.edu:osuc_pubs:309

94 http://lsid.tdwg.org/urn:lsid:biosci.ohio-state.edu:osuc_pubs:459

95 http://lsid.tdwg.org/urn:lsid:biosci.ohio-state.edu:osuc_pubs:361

96 http://lsid.tdwg.org/urn:lsid:biosci.ohio-state.edu:osuc_pubs:310

97 http://lsid.tdwg.org/urn:lsid:biosci.ohio-state.edu:osuc_pubs:22163

98 http://lsid.tdwg.org/urn:lsid:biosci.ohio-state.edu:osuc_pubs:342

99 http://lsid.tdwg.org/urn:lsid:biosci.ohio-state.edu:osuc_pubs:311

100 http://lsid.tdwg.org/urn:lsid:biosci.ohio-state.edu:osuc_pubs:474

101 http://lsid.tdwg.org/urn:lsid:biosci.ohio-state.edu:osuc_pubs:312

102 http://lsid.tdwg.org/urn:lsid:biosci.ohio-state.edu:osuc_pubs:21300

103 http://lsid.tdwg.org/urn:lsid:biosci.ohio-state.edu:osuc_pubs:585

104 doi: 10.1038/437477a

105 http://lsid.tdwg.org/urn:lsid:biosci.ohio-state.edu:osuc_pubs:345

106 http://lsid.tdwg.org/urn:lsid:biosci.ohio-state.edu:osuc_pubs:21214

107 http://lsid.tdwg.org/urn:lsid:biosci.ohio-state.edu:osuc_pubs:1079

108 http://lsid.tdwg.org/urn:lsid:biosci.ohio-state.edu:osuc_pubs:348

109 doi: 10.1098/rspb.2004.2758

110 http://lsid.tdwg.org/urn:lsid:biosci.ohio-state.edu:osuc_pubs:1057

111 doi: 10.1371/journal.pone.0015991

112 http://lsid.tdwg.org/urn:lsid:biosci.ohio-state.edu:osuc_pubs:22482

113 doi: 10.3897/zookeys.20.205

114 http://morphbank.net/?id=787864

115 http://morphbank.net/?id=787865

116 http://morphbank.net/?id=787866

117 http://morphbank.net/?id=787867

118 http://morphbank.net/?id=787868

119 http://morphbank.net/?id=787877

120 http://morphbank.net/?id=787857

121 http://morphbank.net/?id=787860

122 http://morphbank.net/?id=787863

123 http://morphbank.net/?id=787879

124 http://morphbank.net/?id=787855

125 http://morphbank.net/?id=787859

126 http://morphbank.net/?id=787862

127 http://morphbank.net/?id=787856

128 http://morphbank.net/?id=787858

129 http://morphbank.net/?id=786890

130 http://morphbank.net/?id=792606

131 http://morphbank.net/?id=787880

132 http://morphbank.net/?id=787881

133 http://morphbank.net/?id=787846

134 http://morphbank.net/?id=792628

135 http://morphbank.net/?id=792629

136 http://morphbank.net/?id=787833

137 http://morphbank.net/?id=787884

138 http://morphbank.net/?id=787836

139 http://morphbank.net/?id=787886

140 http://morphbank.net/?id=787848

141 http://morphbank.net/?id=787843

142 http://morphbank.net/?id=787845

143 http://morphbank.net/?id=787847

144 http://morphbank.net/?id=796366

145 http://morphbank.net/?id=792613

146 http://morphbank.net/?id=787883

147 http://morphbank.net/?id=787837

148 http://morphbank.net/?id=792612

149 http://morphbank.net/?id=787849

150 http://morphbank.net/?id=829257

151 http://morphbank.net/?id=792611

152 http://morphbank.net/?id=787882

153 http://morphbank.net/?id=792609

154 http://morphbank.net/?id=792608

155 http://morphbank.net/?id=792607

156 http://morphbank.net/?id=787824

157 http://morphbank.net/?id=787821

158 http://morphbank.net/?id=787876

159 http://morphbank.net/?id=787825

160 http://morphbank.net/?id=787826

161 http://morphbank.net/?id=787823

162 http://morphbank.net/?id=787816

163 http://morphbank.net/?id=787817

164 http://morphbank.net/?id=787819

165 http://morphbank.net/?id=787870

166 http://morphbank.net/?id=792636

167 http://morphbank.net/?id=787813

168 http://morphbank.net/?id=787814

169 http://morphbank.net/?id=787869

170 http://morphbank.net/?id=787873

171 http://morphbank.net/?id=787808

172 http://morphbank.net/?id=787805

173 http://morphbank.net/?id=787804

174 http://morphbank.net/?id=787807

175 http://morphbank.net/?id=787874

176 http://morphbank.net/?id=787801

177 http://morphbank.net/?id=787811

178 http://morphbank.net/?id=787810

179 http://morphbank.net/?id=787806

180 http://morphbank.net/?id=787809

181 http://morphbank.net/?id=787875
