# Supplementary material for: Monograph of the Afrotropical species of Scelio Latreille (Hymenoptera, Platygastridae), egg parasitoids of acridid grasshoppers (Orthoptera, Acrididae)
Source: Zookeys. 2014 Feb 17;(380):1–188. doi: 10.3897/zookeys.380.5755 (PMC3950483; doi:10.3897/zookeys.380.5755)
Supplement: Supplementary file 1 [file ZooKeys-380-001-s001.odt]

URI Table of HAO morphological terms.

The table was generated by entering all text occurring in the descriptive portions of the text into the analyze function at the Hymenoptera Anatomy Ontology (HAO) portal (http://api.hymao.org/projects/32/public/ontology/analyze ). The definitions associated with each class can be retrieved by using the URI to navigate to the relevant page.

| Label Used in Text | Unique Identifier of HAO Class |
| --- | --- |
| anteclypeus | http://purl.obolibrary.org/obo/HAO_0000097 |
| axillular carina | http://purl.obolibrary.org/obo/HAO_0000161 |
| bar | http://purl.obolibrary.org/obo/HAO_0000164 |
| body | http://purl.obolibrary.org/obo/HAO_0000182 |
| carina | http://purl.obolibrary.org/obo/HAO_0000188 |
| clypeus | http://purl.obolibrary.org/obo/HAO_0000212 |
| corner | http://purl.obolibrary.org/obo/HAO_0000223 |
| depression | http://purl.obolibrary.org/obo/HAO_0000241 |
| edge | http://purl.obolibrary.org/obo/HAO_0000285 |
| eye | http://purl.obolibrary.org/obo/HAO_0000217 |
| fore leg | http://purl.obolibrary.org/obo/HAO_0000349 |
| fore wing | http://purl.obolibrary.org/obo/HAO_0000351 |
| frons | http://purl.obolibrary.org/obo/HAO_0001044 |
| gena | http://purl.obolibrary.org/obo/HAO_0000371 |
| genal carina | http://purl.obolibrary.org/obo/HAO_0001755 |
| genal pilosity | http://purl.obolibrary.org/obo/HAO_0001987 |
| head | http://purl.obolibrary.org/obo/HAO_0000397 |
| leg | http://purl.obolibrary.org/obo/HAO_0000494 |
| malar sulcus | http://purl.obolibrary.org/obo/HAO_0000504 |
| mandible | http://purl.obolibrary.org/obo/HAO_0000506 |
| margin | http://purl.obolibrary.org/obo/HAO_0001981 |
| mesonotum | http://purl.obolibrary.org/obo/HAO_0000556 |
| mesopleural depression | http://purl.obolibrary.org/obo/HAO_0000326 |
| mesoscutellum | http://purl.obolibrary.org/obo/HAO_0000574 |
| mesoscutum | http://purl.obolibrary.org/obo/HAO_0000575 |
| mesosoma | http://purl.obolibrary.org/obo/HAO_0000576 |
| metapleuron | http://purl.obolibrary.org/obo/HAO_0000621 |
| metasoma | http://purl.obolibrary.org/obo/HAO_0000626 |
| notaulus | http://purl.obolibrary.org/obo/HAO_0000647 |
| nucha | http://purl.obolibrary.org/obo/HAO_0000651 |
| occipital carina | http://purl.obolibrary.org/obo/HAO_0000653 |
| ocellus | http://purl.obolibrary.org/obo/HAO_0000661 |
| patch | http://purl.obolibrary.org/obo/HAO_0000704 |
| pilosity | http://purl.obolibrary.org/obo/HAO_0001990 |
| projection | http://purl.obolibrary.org/obo/HAO_0000829 |
| pronotal collar | http://purl.obolibrary.org/obo/HAO_0000832 |
| pronotum | http://purl.obolibrary.org/obo/HAO_0000853 |
| propodeum | http://purl.obolibrary.org/obo/HAO_0001249 |
| row | http://purl.obolibrary.org/obo/HAO_0000901 |
| scape | http://purl.obolibrary.org/obo/HAO_0000908 |
| sculpture | http://purl.obolibrary.org/obo/HAO_0000913 |
| submarginal vein | http://purl.obolibrary.org/obo/HAO_0000972 |
| sulcus | http://purl.obolibrary.org/obo/HAO_0000978 |
| tooth | http://purl.obolibrary.org/obo/HAO_0001019 |
| transverse pronotal carina | http://purl.obolibrary.org/obo/HAO_0001031 |
| vertex | http://purl.obolibrary.org/obo/HAO_0001077 |
| villus | http://purl.obolibrary.org/obo/HAO_0001083 |
| wing | http://purl.obolibrary.org/obo/HAO_0001089 |
